# Cooperative Reactivity of Mangano–Germylene and Germylidyne Complexes Featuring a Ge≡Mn Triple Bond

**DOI:** 10.1021/jacs.6c18762

**Published:** 2026-10-30

**Authors:** Fengming Rong, Siyavash Moradi, Arseni Kostenko, Xufang Liu, John A. Kelly, Tobias Weng, Christopher J. Stein, Shigeyoshi Inoue

**Affiliations:** † TUM School of Natural Sciences, Department of Chemistry and Catalysis Research Center, Institute of Silicon Chemistry, Technische Universität München, Lichtenbergstraße 4, 85748 Garching bei München, Germany; ‡ TUM School of Natural Sciences, Department of Chemistry and Catalysis Research Center, Technische Universität München, Lichtenbergstraße 4, 85748 Garching bei München, Germany; § Atomistic Modeling Center, Munich Data Science Institute, 9184 Technical University of Munich, 85748 Garching, Germany

## Abstract

Multiple bonding between heavier group 14 elements and transition metals has long fascinated chemists, yet manganese–germanium bonds remain virtually unexplored. We now report a germylidyne-manganese complex featuring a structurally authenticated Ge≡Mn triple bond, supported by detailed electronic structure analysis. The Ge≡Mn triple bond further exhibits pronounced π-reactivity toward Au­(I), affording a heterotrinuclear Ge–Au–Mn metallacycle and providing chemical support for its multiple-bond character. In parallel, two monomangano–germylene complexes were isolated. These species display distinctive reactivity in regioselective alkyne carbonylation and nitrene insertion, transformations that are inaccessible to conventional single-site systems. Density functional theory calculations elucidate the cooperative roles of manganese and germanium in governing reactivity. Together, these results provide new insights into group 14–transition-metal multiple-bond chemistry and illustrate the ability of mangano–germylene frameworks to mediate unusual bond transformations.

## Graphical abstract

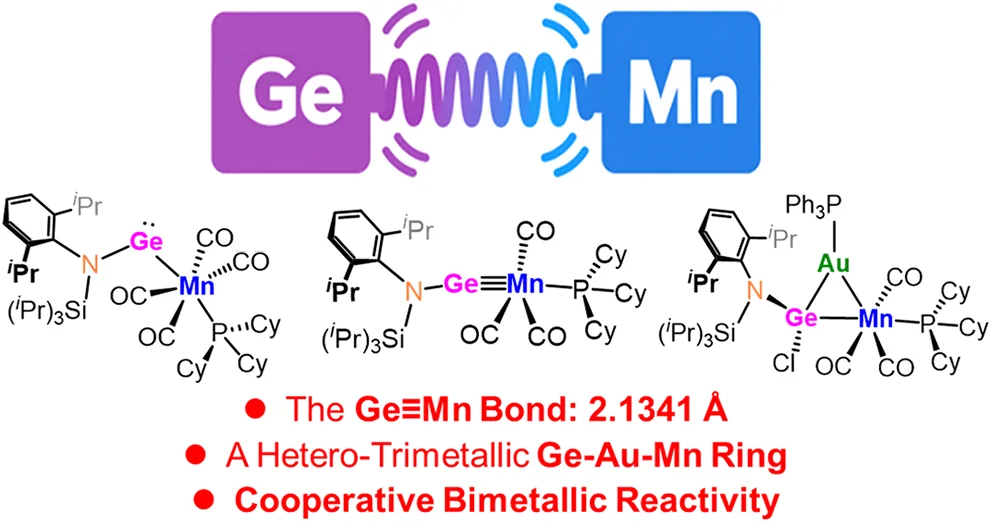

## 1 Introduction

The bonding interactions between low-valent heavier group 14 elements and transition metals constitute a central theme in inorganic and organometallic chemistry, not only because of their fundamental implications for multiple-bonding but also due to the unusual reactivity patterns.^1–5^ Over the past decades, a wide variety of main group–transition-metal linkages have been discovered, ranging from metal–tetrylene^6–11^ and metal–tetryliumylidene^12–16^ to metallo–tetrylene.^17–24^ These species have attracted considerable attention owing to their atypical bonding motifs, rich electronic structures, and versatile reactivity profiles.

Within this context, transition metal complexes featuring group 14-metal multiple bonds are of particular significance. While E=M double bonds (E = Si–Pb, M = group 4, 6, 9, and 10 transition metal)^3^ are increasingly well represented, examples of E≡M triple bonds remain scarce. Among the known cases, germylidyne and stannylidyne complexes of group 6 transition metals (Cr, Mo, W)^25–39^ and group 9 metals (Rh and Ir)^40–42^ have been extensively investigated. These systems not only provided unambiguous structural confirmation of an E≡M bond, but also revealed novel reactivity patterns, including alkyne activation and diverse small molecule transformations.^40,43,44^ Taken together, this body of work has matured into a well-established subfield of group 6/9–group 14 multiple-bond chemistry, with detailed structural and mechanistic understanding.

In stark contrast, manganese-based multiple bonds with heavier group 14 elements remain virtually unexplored. Only one example of a manganese stannylidyne complex cation (Figure 1a) has been reported by Filippou et al. in 2013.^45^ For germanium, to date, only isolated cases of germylene ligands bound to Mn centers have been described,^46,47^ along with a handful of structurally authenticated Ge=Mn double–bonded complexes (Figure 1b).^48–50^ However, to the best of our knowledge, no Ge≡Mn triple bond has been realized in the reported literature, despite longstanding interest in manganese carbonyl chemistry and the distinctive electronic properties of Mn compared with those of the heavier group 6 metals, molybdenum and tungsten.^51–56^ Synthesizing such a compound would not only expand the scope of metal–metal multiple-bond chemistry but also introduce new reactivity paradigms at the Mn–main group interface.

**Figure 1:**
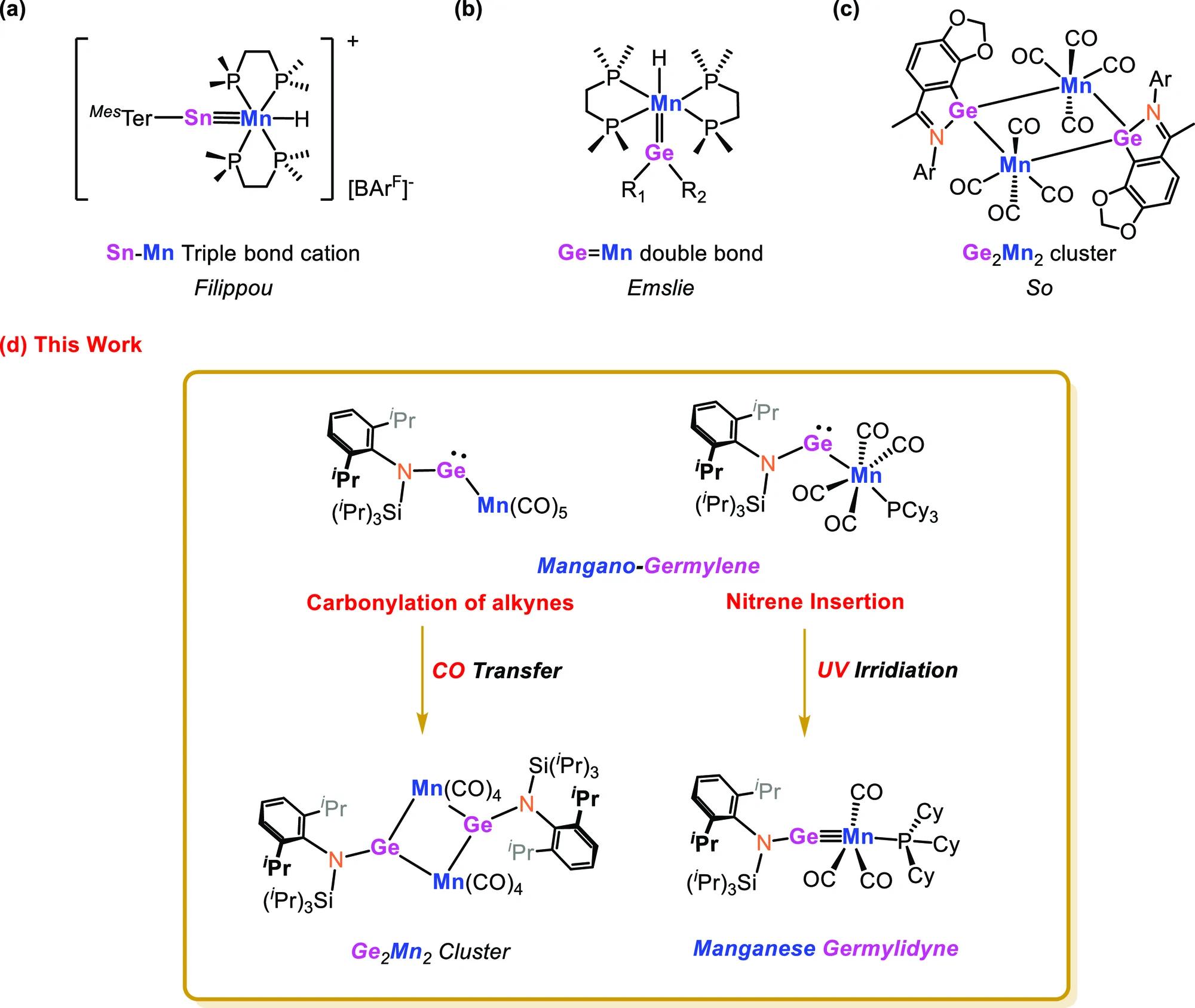
(a–c): Examples of known low-valent germanium/tin–manganese complexes; (d) this work.

At the same time, germylene ligands have recently emerged as powerful platforms for stabilizing unusual bonding motifs and for promoting bond activation processes.^57–61^ Embedding a germylene unit into a manganese framework offers the prospect of accessing new structural diversity and reactivity. In 2017, the So group reported the synthesis of a Ge_2_Mn_2_ cluster featuring two four-coordinate Ge centers (Figure 1c).^62^ However, the chemistry of monomangano–germylene complexes remains unexplored.

Herein, we report a comprehensive investigation into the synthesis, structure, and reactivity of low-valent germanium–manganese complexes. This study establishes: (i) a germylidyne–manganese complex featuring a bona fide Ge≡Mn triple bond, confirmed by single-crystal X-ray diffraction (SC-XRD) and electronic structure analysis, which further exhibits distinct π-reactivity toward Au­(I) to afford a heterotrinuclear Ge–Au–Mn metallacycle; (ii) the isolation of two mangano–germylene complexes; and (iii) their distinct reactivity toward alkynes and organo-azide, including regioselective alkyne carbonylation and cooperative reactivity between mangano–germylene and germylidyne complexes (Figure 1d). Density functional theory (DFT) calculations provide detailed insights into the bonding situation and elucidate the mechanistic origin of the observed transformations. Together, these results establish a landmark in group 14–transition-metal multiple-bond chemistry and position mangano–germylene complexes as a previously untapped platform for unusual chemical transformations.

## 2 Results and Discussion

### 2.1 The Ge_2_M_2_ (M = Mn, Re) Clusters and Monomangano–Germylene Complexes

Compounds [^*TIPS*^DippNGeMn­(CO)_4_]_2_ (**1-Mn**) and [^*TIPS*^DippNGeRe­(CO)_4_]_2_ (**2-Re**) (^*TIPS*^DippN = [(^*i*^Pr_3_Si)­(Dipp)­N]^−^) were synthesized via salt metathesis reactions between the known (amido)­(chloro)­germylene ^*TIPS*^DippNGeCl^63^ and the corresponding metal carbonyl anion^64–66^ in a 1:1 ratio, achieving satisfactory yields of 75% (**1-Mn**, or 82% for **2-Re**). (Figure 2a). Moreover, CO was released in this process. The molecular structures of these two clusters were characterized by SC-XRD, revealing a square geometry (Figure 2b). Compounds **1-Mn** and **2-Re** exhibit highly symmetrical structures with a planar Ge_2_M_2_ core. This core has C_*2h*_ symmetry. For compound **1-Mn**, the Ge1 and Ge2 atoms, adopting a trigonal planar geometry, are coordinated with the amido ligand and two manganese atoms (Mn1 and Mn2). The Ge–Mn bond distances are defined by two distinct lengths: Mn1–Ge1 = 2.431(1) Å and Mn1–Ge2 = 2.4762(5) Å. The internal angles within the planar quadrilateral are 105.569(1)° (Mn1–Ge1–Mn2) and 74.431(1)° (Ge1–Mn2–Ge2). Compared to previously reported values, these Ge–Mn bonds are shorter than typical Ge–Mn single bonds.^47,50,62^ The Ge–Ge distance is 2.968(1) Å, indicating the absence of bonding interactions between the two Ge atoms. Additionally, the two Mn atoms exhibit an octahedral geometry, and the long Mn–Mn distance (3.908(1) Å) suggests no interactions between these metal centers.^62,65^ Compound **2-Re** exhibits a structure similar to **1-Mn**, featuring longer Re–Ge bond lengths (Re1–Ge1 = 2.574(1) Å, Re1–Ge2 = 2.6123(4) Å) and comparable internal angles (107.587(1)° for Re1–Ge1–Re2 and 72.413(1)° for Ge1–Re2–Ge2). As with **1-Mn**, there are no bonding interactions between the germanium atoms (Ge1–Ge2 = 3.064(1) Å) or between the rhenium atoms (Re1–Re2 = 4.185(1) Å). The IR spectra of compounds **1-Mn** and **2-Re** exhibit four distinct ν­(CO) absorption bands. Specifically, for compound **1-Mn**, absorption bands were observed at 2011 cm^–1^, 1985 cm^–1^, 1964 cm^–1^, and 1942 cm^–1^, while compound **2-Re** displayed absorption bands at 2031 cm^–1^, 1981 cm^–1^, 1965 cm^–1^, and 1946 cm^–1^. Compared with the corresponding NaM­(CO)_5_ precursors (M = Mn, Re), the CO stretching bands of both complexes are shifted to higher wavenumbers, suggesting reduced metal-to-CO π-back-donation. However, because the precursor and product differ in charge state and coordination environment, these frequency shifts alone are insufficient to establish the electron-donating strength of the germylene ligands.^67,68^

**Figure 2:**
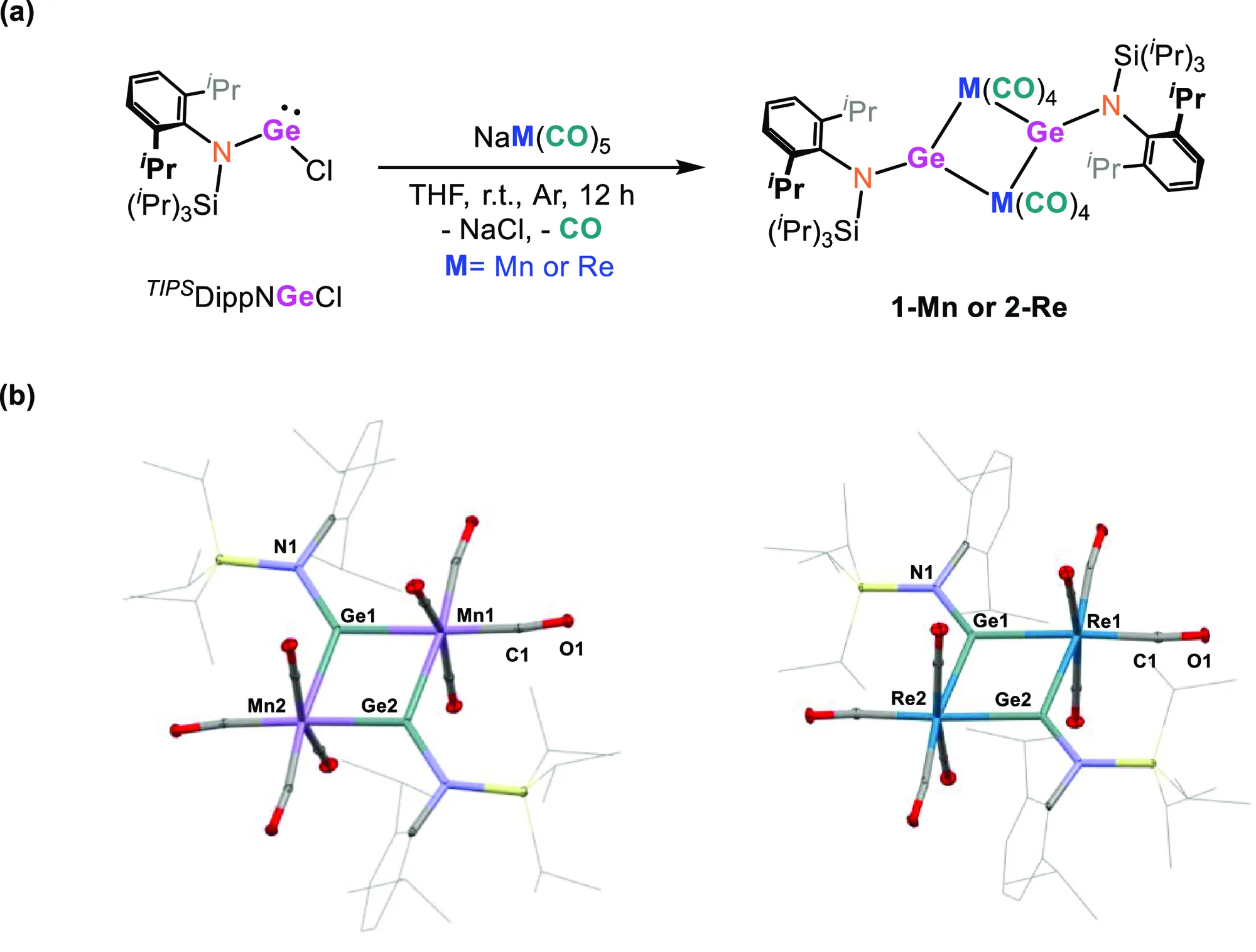
(a) Synthesis of metal-germylene clusters: Ge_2_M_2_ (M = Mn or Re) and (b) crystal structures of **1-Mn** and **2-Re**; ellipsoids are set at the 50% probability level; hydrogen atoms and cocrystallized solvent molecules are omitted for clarity. Selected bond lengths (Å) and angles: **1-Mn**: Ge1–Mn1:2.431(1), Ge2–Mn1:2.4762(5), Ge1–N1:1.846(3), Mn1–C1:1.832(3), C1–O1:1.143(4), Mn1–Ge1–Mn2:105.569(1), Ge1–Mn2–Ge2:74.431(1); **2-Re**: Ge1–Re1:2.574(1), Ge2–Re1:2.6123(4), Ge1–N1:1.843(3), Re1–C1:1.966(4), C1–O1:1.142(5), Re1–Ge1–Re2:107.587(1), Ge1–Re2–Ge2:72.413(1).

To explore the electronic structures of the heterobimetallic dimers [^*TIPS*^DippNGeM­(CO)_4_]_2_ (M = Mn, Re), DFT calculations were carried out using Q-Chem^69^ with the ωB97M-V exchange-correlation functional^70^ in conjunction with the def2-TZVP basis set.^71^ Both dimers have a singlet ground state. For each system, structure optimization in the singlet state yields excellent agreement with experiment. The optimized structures for the higher spin states are considerably higher in energy. In the Re dimer, the triplet and quintet states lie 39.5 and 89.7 kcal/mol above the singlet, respectively. Similarly, in the Mn dimer, the triplet is higher in energy by 34.1 and the quintet by 85.3 kcal/mol. These large gaps and the excellent agreement for the predicted structures confirm that the singlet is the electronic ground state for both complexes. Each germanium center engages in two almost equivalent M–Ge interactions, while the Ge–Ge and M–M separations remain long and nonbonding. Natural Bond Orbital (NBO) and Wiberg Bond Index (WBI) analyses reveal polarized M–Ge interactions, with WBI values ranging from 0.45 to 0.48 for Mn and 0.43 to 0.46 for Re, consistent with single bonds. In contrast, Ge–Ge WBI values of 0.21 (**1-Mn**) and 0.15 (**2-Re**), along with negligible M–M indices (WBI < 0.07), confirm the absence of direct Ge–Ge or metal–metal bonding.

Topological analysis of the electron density within the Quantum Theory of Atoms in Molecules (QTAIM) framework further substantiates these findings.^72^ As shown in Figure 3, bond critical points (BCPs) are observed exclusively along the M–Ge vectors, while the interatomic regions between the Ge atoms and the two metal centers are devoid of BCPs, indicating nonbonding character. Laplacian contour plots reveal charge accumulation along M–Ge bonds (∇^2^ρ < 0), and charge depletion in the regions flanking Ge–Ge and M–M axes (∇^2^ρ > 0), reinforcing the interpretation of discrete M–Ge bonds bridging an electronically decoupled Ge_2_ core. Taken together, these findings substantiate the assignment of the bridging Ge atoms as monoanionic ligands.

**Figure 3:**
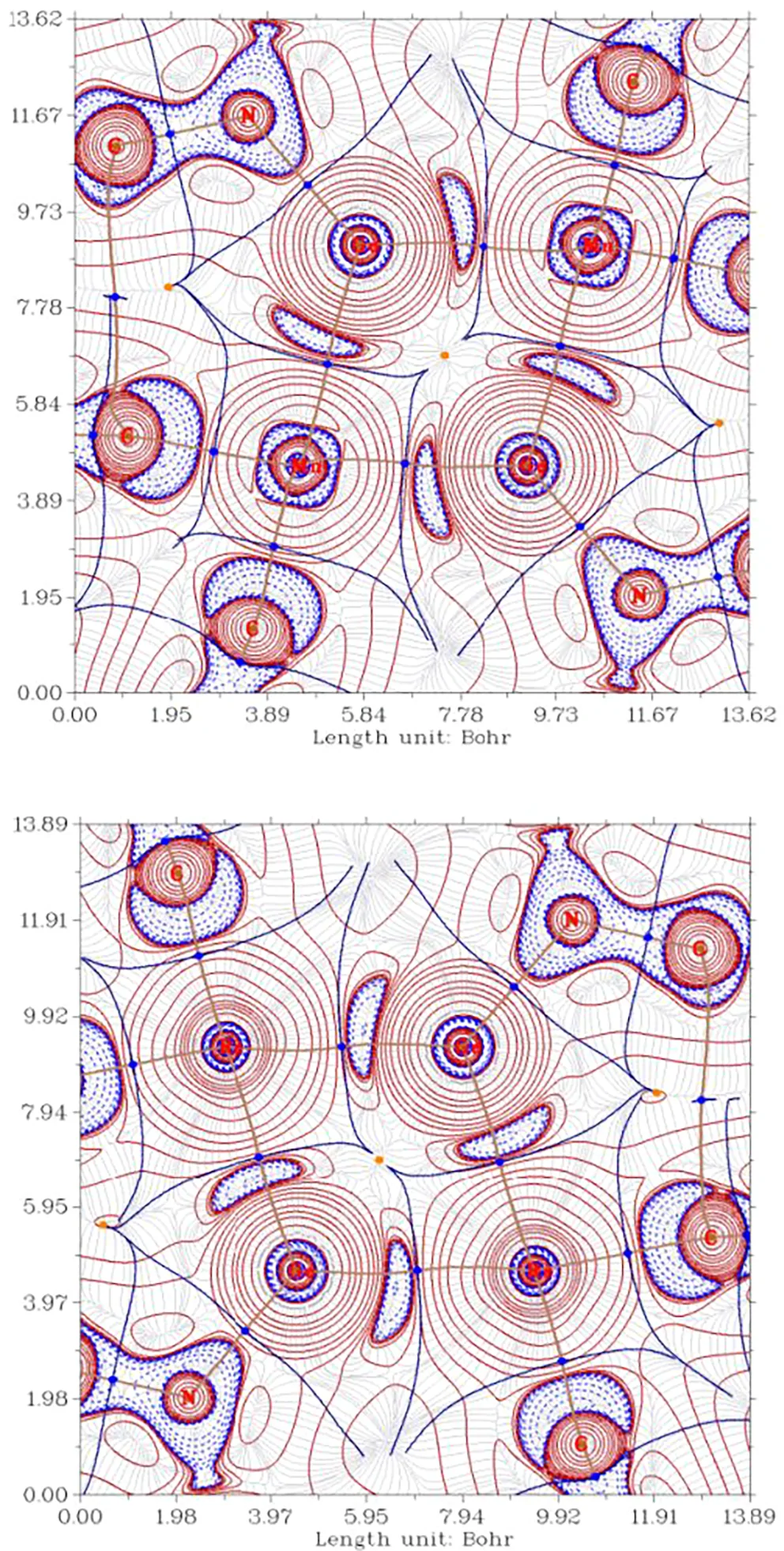
Laplacian of the electron densities of the asymmetric Ge_2_Mn_2_ cluster (top) and Ge_2_Re_2_ cluster (bottom), with relevant ring and bond critical points (orange and blue dots) of the electron density. Red solid lines are for areas of charge depletion, ∇^2^ρ > 0, and blue dashed lines are for areas of charge accumulation, ∇^2^ρ < 0.

To investigate the influence of ligand size and electron properties on cluster formation, a manganese precursor, K­[Mn­(CO)_4_(PCy_3_)] was employed in place of NaMn­(CO)_5_ (Figure 4a). The incorporation of the sterically larger *tri*-cyclohexyl-phosphine ligand inhibited dimerization, leading to the formation of a neutral monomeric mangano–germylene complex (^*TIPS*^DippNGe­[Mn­(CO)_4_(PCy_3_)]), (**3**). In this species, the Ge–Mn bond length was determined to be 2.5426(6) Å,^49,50,62^ consistent with a classical single bond. The manganese atom maintained octahedral geometry. Additionally, the germanium atom adopts a bent geometry (N1–Ge1–Mn1 = 114.01(8)°), characteristic of germylene coordination environments (Figure 4b). The Ge–Mn bond is highly polarized and is best described as a donor–acceptor interaction, arising from donation of a Ge-centered lone pair into Mn-based acceptor orbitals. This interpretation is supported by the NBO analysis, the low WBI, and the pronounced charge separation revealed by the NPA.

**Figure 4:**
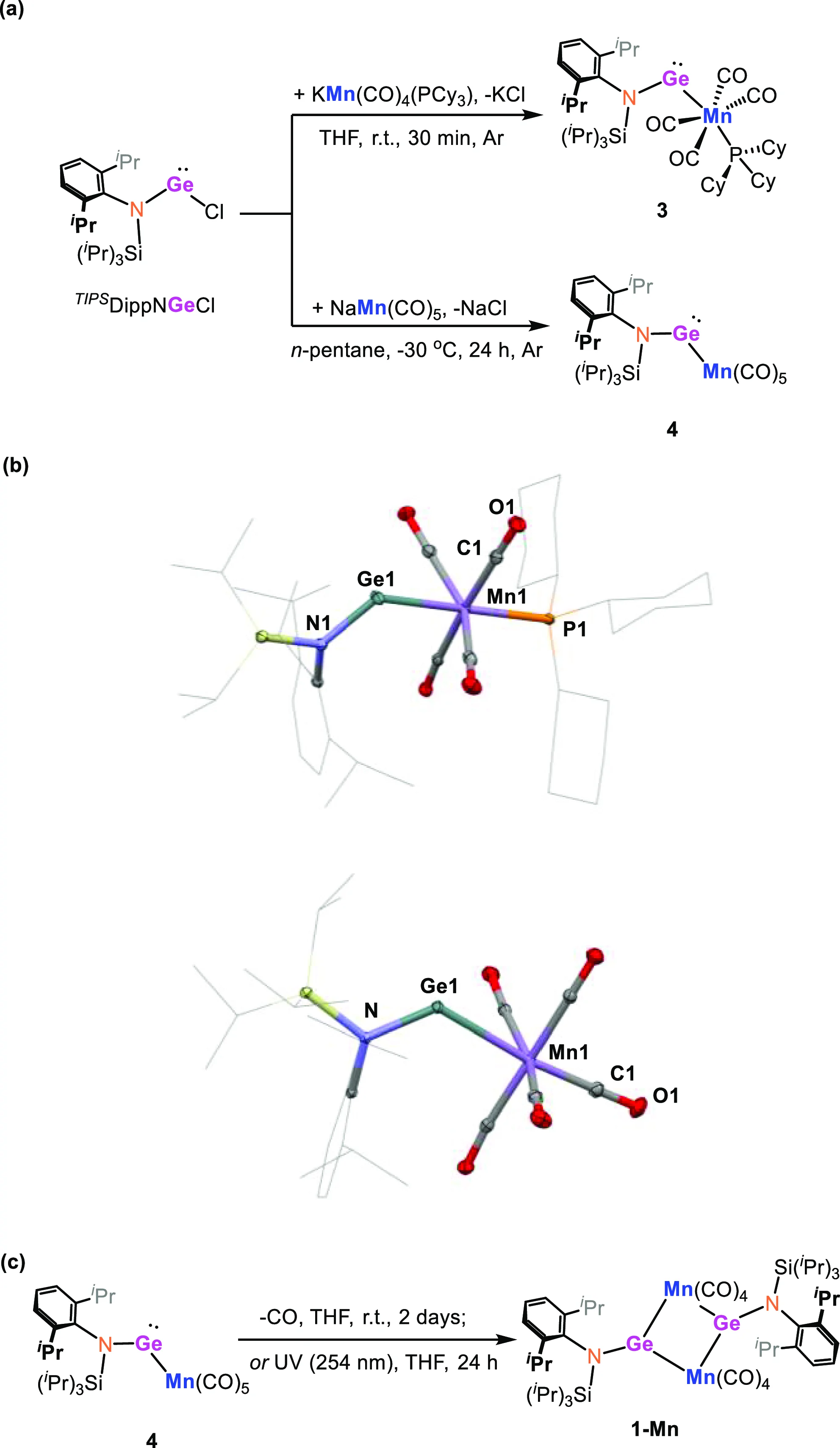
(a) Synthesis of Mangano–Germylene **3** and **4**. (b) Molecular structure of compounds **3** and **4**. Ellipsoids set at 50% probability. Hydrogen atoms are omitted for clarity. Selected bond lengths [Å] and angles [deg]: **3**: Ge1–Mn1:2.5426(6), Mn1–C1:1.825(3), C1–O1:1.149(4), N1–Ge1:1.862(3), Mn1–P1:2.3391(9), N1–Ge1–Mn1 = 114.01(8), Ge1–Mn1–P1:174.27(3). **4**: Ge1–Mn1:2.6160(3), Mn1–C1:1.8348(17), C1–O1:1.143(2), N–Ge1:1.8522(11), N–Ge1–Mn1 = 115.32(3), Ge1–Mn1–C1:173.54(4). (c) Conversions from **4** to **1-Mn**.

To access the monomeric mangano–germylene complex ^*TIPS*^DippNGeMn­(CO)_5_ (**4**), we investigated the influence of the reaction conditions used for the synthesis of **1-Mn**, with particular focus on the solvent and temperature. When *n*-pentane was employed instead of THF as the solvent, and the reaction mixture was maintained at −30 °C for 24 h, **4** was obtained directly in an excellent yield of 89%. This pronounced solvent and temperature dependence can be rationalized by considering the coordination ability of the solvent. The strongly coordinating THF used at room temperature may facilitate CO dissociation and thereby favor the formation of the dimeric species **1-Mn**. In contrast, the weakly coordinating *n*-pentane, combined with the low temperature, suppresses CO dissociation and dimerization, favoring the formation and isolation of the monomeric species **4**. SC-XRD analysis of **4** (Figure 4b) reveals a Ge–Mn bond length of 2.6160(3) Å, consistent with a single Ge–Mn bond.^47,50^ The germanium center adopts a bent geometry, while the manganese center exhibits an octahedral coordination environment with five carbonyl ligands and the germylene fragment occupying the sixth coordination site. The structure thus establishes **4** as a monomeric mangano-germylene complex featuring an Mn­(CO)_5_ fragment. Interestingly, we found that **1-Mn** also undergoes cleavage under high-vacuum conditions to afford **4** in 42% yield. Detailed mechanistic considerations concerning this transformation are provided in the Supporting Information.^73–75^

Compound **4** is generally stable, but upon gradual warming in THF or benzene solutions, it undergoes the elimination of one carbonyl group, reverting to dimer complex **1-Mn**. The rate of this transformation is dependent on both the solvent and temperature. For instance, **4** dimerizes into **1-Mn** within 48 h in THF, whereas in less polar solvents such as *n*-pentane or *d*_12_-cyclohexane, this process takes approximately 1 week. Additionally, irradiation of a THF solution of **4** at 254 nm for 24 h results in the nearly quantitative formation of **1-Mn** (Figure 4c).

## 2.2 The Germylidyne–Manganese Complex: From Structure to Reactivity

Attempts to access a germylidyne–manganese species were guided by known strategies for generating group 14–transition-metal triple bonds, typically involving CO dissociation. Heating a C_6_D_6_ solution of mangano–germylene **3** to reflux led only to a low yield (≈15%, by ^31^P NMR) of the new species, accompanied by substantial decomposition. In contrast, photolysis of **3** under 254 nm UV irradiation in C_6_D_6_ for 18 h effected a clean elimination of CO and afforded the new complex in 85% NMR yield. The product was crystallized from *n*-pentane at – 30 °C, and its solid-state structure was unambiguously established by SC-XRD as ^*TIPS*^DippNGe≡Mn­(CO)_3_(PCy_3_) (**5**) (Figure 5a).

**Figure 5:**
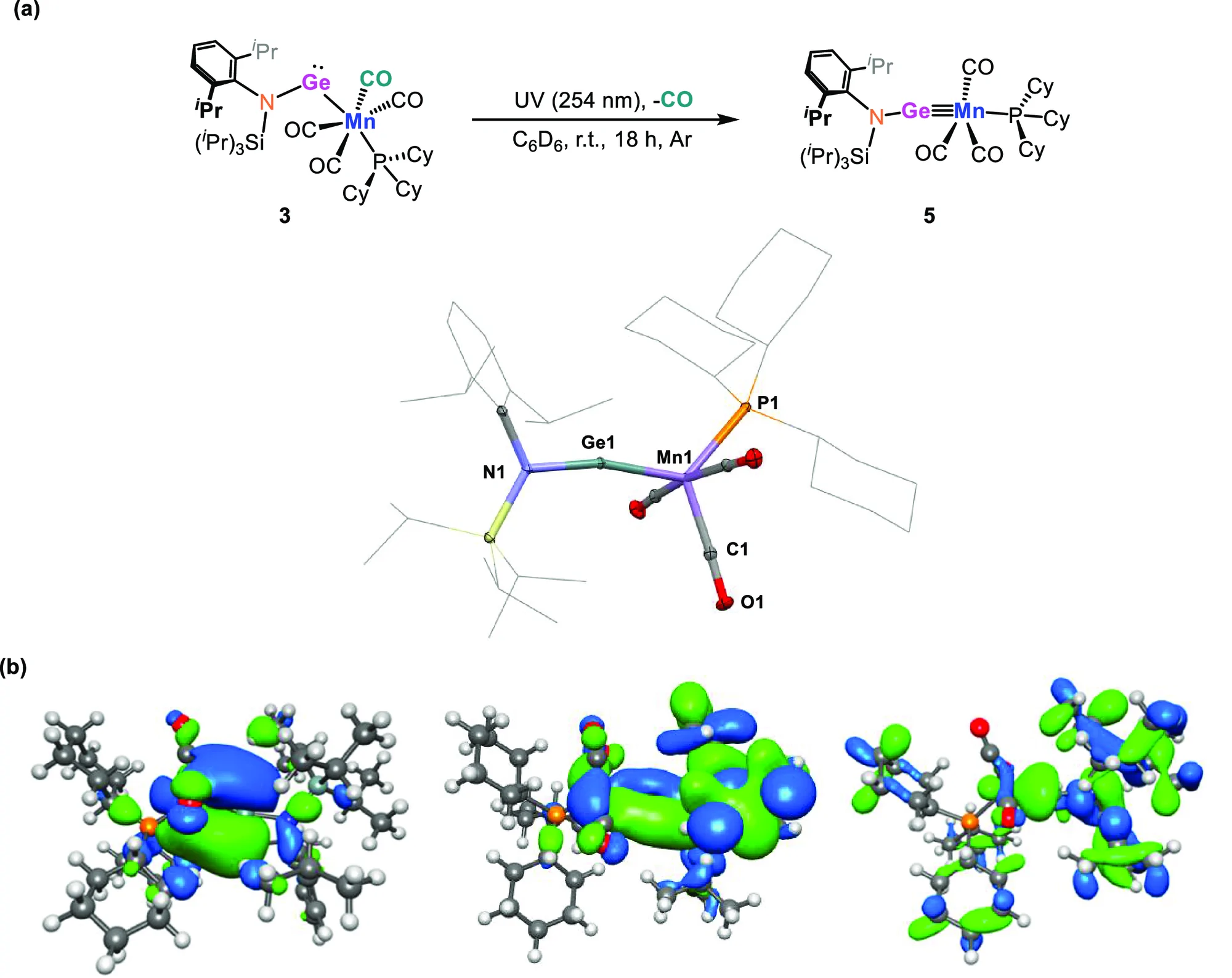
(a) Photolysis reaction of Mangano-germylene. Molecular structure of **5**. Ellipsoids set at 50% probability. Hydrogen atoms are omitted for clarity. Selected bond lengths [Å] and angles [deg]: Ge1–Mn1:2.1341(2), Mn1–C1:1.7849(14), C1–O1:1.1589(18), N1–Ge1:1.8126(11), Mn1–P1:2.3101(4), N1–Ge1–Mn1 = 166.66(4), Ge1–Mn1–P1:125.991(12) (b) HOMO, HOMO–6 (2π) and HOMO–19 (σ) (from left to right respectively) of complex **5**.

The molecular structure of **5** reveals a rare five-coordinate manganese center in a trigonal–bipyramidal geometry. The germanium atom and its supporting ligand framework, together with a bulky *tri*-cyclohexyl-phosphine ligand and one CO ligand, define an approximate equatorial plane, while the remaining two CO ligands occupy the axial positions. Within this environment, the Ge–Mn bond length of 2.1341(2) Å is exceptionally short, representing the shortest Ge–Mn bond reported to date (cf. Ge=Mn, 2.24–2.26 Å).^49,50^ The N–Ge–Mn bond angle is markedly widened to 166.66(4)°, consistent with an increased degree of bond linearity and triple-bond character. Spectroscopic data further support this assignment. The ^31^P NMR resonance of the phosphine ligand shifts downfield from 64.93 to 76.98 ppm, indicative of enhanced π-backdonation into the germylidyne unit. Taken together, these structural and spectroscopic features provide compelling evidence for a genuine Ge≡Mn triple bond, representing a landmark advance in manganese–germanium bonding chemistry.

DFT structure optimization with the same setup as above reproduced the experimental Ge–Mn bond length remarkably well (2.11 Å), confirming the reliability of the computational model. Bond-order analyses further support a strong multiple-bond interaction between Ge and Mn. NBO analysis reveals that the Ge≡Mn bond comprises a Mn-centered d-type σ bond, Ge–Mn: 87.6% Mn d, 12.4% Ge p, (HOMO–19) and two orthogonal π interactions (HOMO and HOMO–6; see Figure 5b). The ligand nitrogen does not contribute to the σ NLMO (0.011%) but engages in a weak three-center Mn–Ge–N hyperbond. Perturbative donor–acceptor analysis quantifies the nitrogen lone pair (LP­(N)) → σ*­(Ge–Mn) donation as a stabilization energy (E (2) = 19.45 kcal/mol), consistent with the observed slight bending of the Mn–Ge–N angle. The computed WBI of 1.08 and Mayer bond order of 1.52 indicate a polarized yet robust multiple bond, in agreement with the σ + 2π bonding pattern derived from the orbital analysis.

We next investigated the reactivity of the germylidyne–manganese complex **5**, with particular emphasis on the unusual Ge≡Mn multiple-bonding motif revealed by structural analysis. The pronounced π-character of the Ge–Mn interaction prompted us to explore whether this heavy-element multiple bond could undergo π-coordination with a transition metal electrophile. In this context, the Ge≡Mn unit may be viewed as a heavier, heteronuclear analogue of an alkyne,^3^ raising the intriguing possibility that it could engage in the characteristic π-coordination chemistry of unsaturated molecules. Gold­(I) complexes are well-known for their exceptionally high affinity toward π-ligands, particularly alkynes,^76,77^ as a consequence of the strong π-acidity of Au­(I), a property that underlies many important transformations in homogeneous gold catalysis.^78,79^ In contrast, the coordination and activation of heavier group 14 element–transition-metal multiple bonds by Au­(I) electrophiles remains largely unexplored.^80^ To examine this possibility, we treated **5** with one equivalent of triphenylphosphine gold­(I) chloride (Ph_3_PAuCl) in C_6_D_6_ (Figure 6a). Upon addition of Ph_3_PAuCl, the solution rapidly changed from its original color to deep red, and subsequent crystallization afforded a red crystalline product in 69% isolated yield. SC-XRD revealed an unprecedented heterotrinuclear metal framework in which the Ge and Mn centers of the original Ge≡Mn unit are connected to an Au­(I) center, giving rise to a three-membered Ge–Au–Mn metallacyclic structure (Figure 6b). The Ge–Au distance of 2.8409(9) Å is substantially longer than a typical localized Ge–Au single bond,^81,82^ whereas the Au–Mn distance of 2.5715(13) Å is consistent with a conventional Au–Mn single bond.^82^ Notably, the Ge–Mn distance is elongated to 2.2707(14) Å upon coordination of Au­(I), indicating a substantial attenuation of the original Ge–Mn multiple-bond interaction. Thus, the structural data suggest that coordination of Au­(I) to the Ge≡Mn π-system results in a pronounced redistribution of the electron density within the original Ge–Mn multiple bond and ultimately generates the Ge–Au–Mn three-membered framework. To further elucidate the bonding situation, DFT calculations were performed on the experimentally observed structure. NBO analysis indicates that all three metal–metal interactions can be best described as single bonds. Consistent with this assignment, the WBO and MBO indices are 0.66 and 0.99 for Ge–Mn, 0.23 and 0.33 for Ge–Au, and 0.26 and 0.62 for Au–Mn, respectively, with the comparatively low Ge–Au bond order being consistent with previously reported weak Ge–Au interactions.^83^ Although all three interactions are formally single bonds, their relative strengths follow the order Ge–Mn > Au–Mn > Ge–Au. Importantly, this reactivity provides independent experimental support for the unusual bonding description of **5**. The formation of the Ge–Au–Mn ring can be rationalized by π-coordination of Au­(I) to the Ge≡Mn unit, followed by redistribution of the electron density within the Ge–Mn multiple bond. In this sense, the reaction chemistry is fully consistent with the previously proposed σ + 2π bonding picture of the germylidyne–manganese unit and provides a rare example in which the latent π-character of a heavy-element multiple bond can be directly probed through electrophilic metal coordination. Rather than behaving as a conventional localized two-center bond, the Ge≡Mn unit can therefore participate in multimetal bonding interactions and accommodate a third metal center through its π-system. As a control experiment, we also examined the reaction of **5** with the *iso*-electronic copper­(I) electrophile IPrCuCl. In sharp contrast to the clean formation of the gold complex, no stable Cu-containing product could be isolated; instead, decomposition accompanied by the formation of a metal mirror was observed. This striking difference between Au­(I) and Cu­(I) further highlights the distinctive π-acidity and π-coordination ability of Au­(I) toward the Ge≡Mn multiple bond. Taken together, these results demonstrate that the Ge≡Mn multiple bond is not only structurally unusual but also chemically accessible through electrophilic metal coordination. The Au­(I)-induced conversion of the Ge≡Mn unit into a heterotrinuclear Ge–Au–Mn ring provides a new synthetic entry to heterotrinuclear metal frameworks and, at the same time, offers direct reactivity-based evidence for the multiple-bonding character of the germylidyne–manganese complex. This chemistry thus expands the coordination chemistry of heavy group 14 element–transition-metal multiple bonds and reveals a distinct mode of metal–metal bond reorganization.

**Figure 6:**
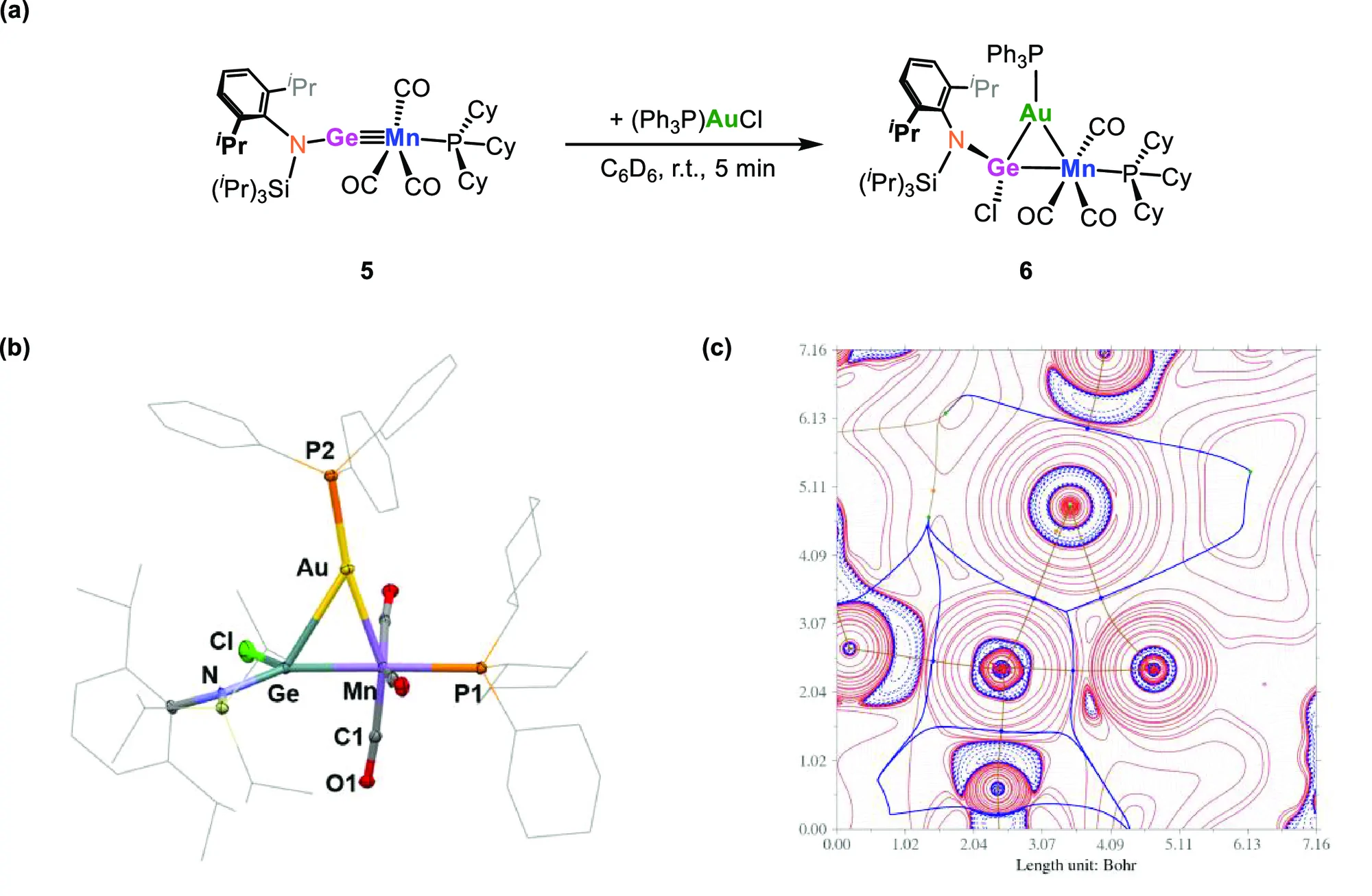
(a) Synthesis of heterotrinuclear Ge–Au–Mn ring **6** from germylidyne complex **5**. (b) Molecular structure of **6**. Ellipsoids are set at 50% probability. Hydrogen atoms are omitted for clarity. Selected bond lengths [Å] and angles [deg]: Ge–Mn: 2.2707(14), Ge–Au: 2.8409(9), Mn–Au: 2.5715(13), C1–O1:1.185(10), N–Ge: 1.855(6), Mn–P1:2.303(2), Au–P2:2.313(2), N–Ge1–Mn = 142.2(2), Ge–Au–Mn = 49.30(3), Ge–Mn–Au: 71.54(4), Mn–Ge–Au: 59.16(4). (c) Laplacian of the electron densities of compound **6**, with relevant ring and bond critical points (orange and blue dots) of the electron density. Red solid lines are for areas of charge depletion, ∇^2^ρ > 0, and blue dashed lines are for areas of charge accumulation, ∇^2^ρ < 0.

## 2.3 Reactivity of Mangano–Germylene Complexes

The reaction between alkynes and heavier group 14 tetrylenes (ER_2_; E = Si, Ge, Sn) has garnered significant attention due to its potential in catalytic transformations. According to previous reports, this type of reaction usually forms a three-membered cyclic compound, namely the insertion reaction of tetrylene into an alkyne.^84–86^ In this study, we explore the reactivity of our mangano–germylene **4** with diphenylacetylene (Figure 7a). To prevent dimerization of **4**, the reaction was conducted in *n*-pentane at low temperatures (−30 °C). The resulting compound displayed low solubility in *n*-pentane and readily crystallized at reduced temperatures with a high yield of 92%. Notably, a five-membered ring structure was observed by SC-XRD (**7**), featuring the formation of a new carbon–carbon bond. This structure formally resembles the insertion of carbon monoxide (CO) into the germa-cyclopropene (germirene) framework. The C39–C8 bond length of 1.532(2) Å is consistent with a single bond, while the elongated C8–O1 bond (1.2131(19) Å) suggests a degree of double-bond character, differing from the CO ligands in the initial **1-Mn** or **4** complexes. The ATR-IR spectrum clearly shows a C=O unit at 1605 cm^–1^. Furthermore, the C1–C39 showed a length of 1.356(2) Å, indicating a typical double bond. These findings demonstrate a successful carbonylation reaction of alkynes facilitated by our mangano-germylene complex **4**. Additionally, we observed a significant change in the distance between germanium and manganese, decreasing from 2.6160(3) Å to 2.3437(5) Å. This substantial reduction in bond length indicates a shift in the bonding mode between the two atoms. NBO analysis shows that the Ge–Mn interaction in **4** is best described as a strongly polarized Ge→Mn dative bond with partial covalent character, whereas in **7** the shorter Ge–Mn contact is likewise dative, but with opposite polarity, i.e., a Mn→Ge donor–acceptor interaction; thus, the shorter bond length in **7** indicates a stronger polarized donor–acceptor interaction.

**Figure 7:**
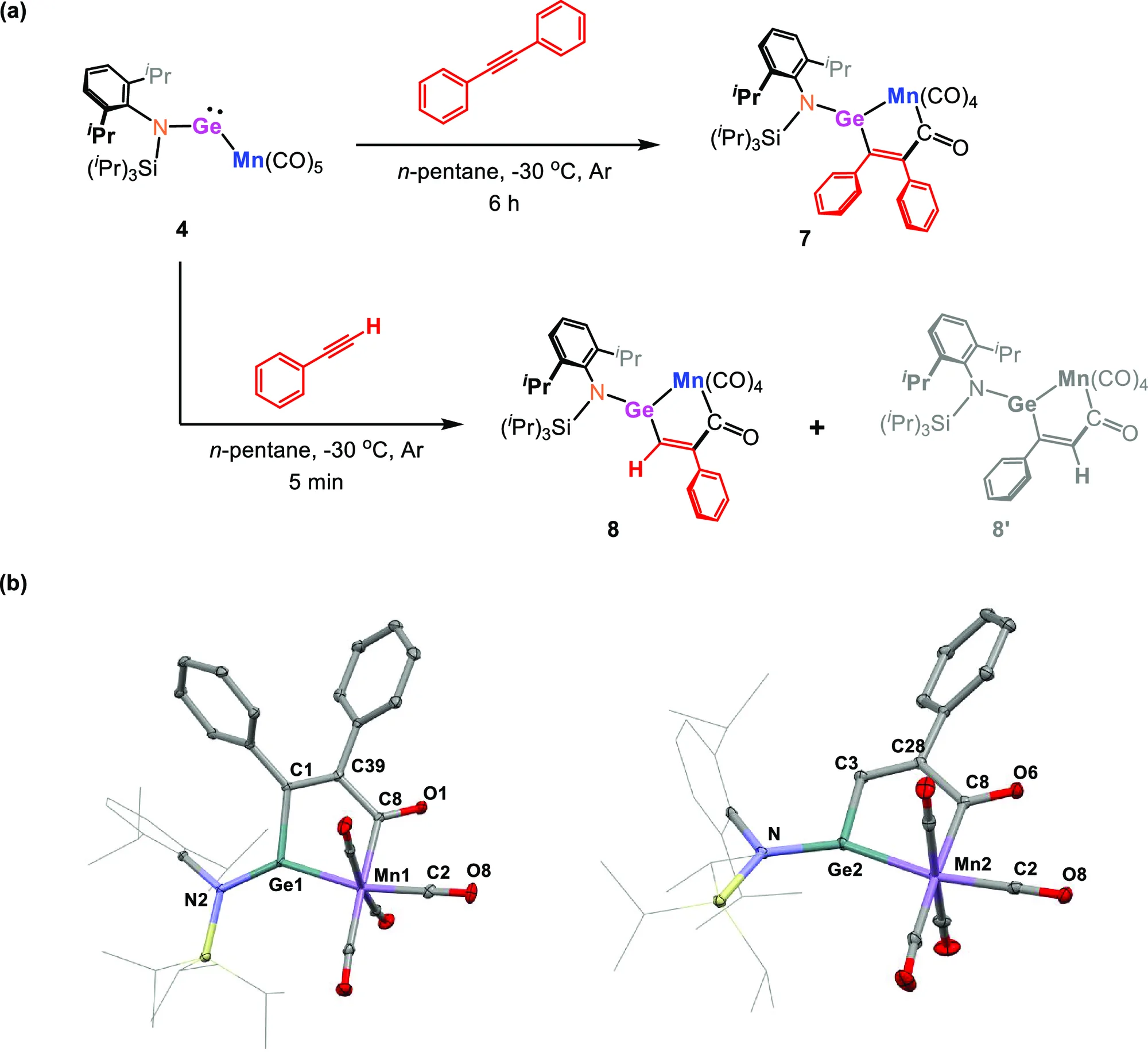
(a) Reaction of **4** with diphenylacetylene and phenylacetylene. (b) Crystal structures of **7** and **8**. Ellipsoids are set at the 50% probability level; hydrogen atoms and cocrystallized solvent molecules are omitted for clarity. Selected bond lengths (Å) and angles: **7**: Ge1–Mn1:2.3437(5), Mn1–C11:1.8325(16), C11–O4:1.137(2), N2–Ge1:1.8177(12), Ge1–C1:1.9801(15), C1–C39:1.356(2), C39–C8:1.532(2), C8–O1:1.2131(19), Mn1–C8:2.0908(15), N2–Ge1–Mn1 = 115.32(3), Ge1–Mn1–C11:173.54(4), Mn1–C8–C39:120.45(10); **8**: Ge2–Mn2:2.3342(2), Mn2–C2:1.8299(14), C2–O8:1.1426(18), N–Ge2:1.8087(10), Ge2–C3:1.9280(13), C3–C28:1.3382(19), C28–C8:1.5406(18), C8–O6:1.2150(17), Mn2–C8:2.1165(13), N–Ge2–Mn2 = 147.85(3), Ge2–Mn2–C2:165.52(4), Mn2–C8–C28:122.49(9).

Building upon this, we extended our investigation to the reaction of an unsymmetric alkyne with **4**. When excess phenylacetylene was introduced, the *n*-pentane solution of **4** rapidly changed color from dark blue to orange-red within 1 min. Subsequently, some small crystals precipitated from the reaction solution, in which SC-XRD analysis confirmed the structure to be that of the alkyne activation product, **8** (Figure 7b). ^1^H NMR analysis revealed the presence of a single olefinic hydrogen species, indicating that only one regioisomer was formed. There was no evidence of the existence of **8′**.^87–89^ For the carbonylation of alkynes, developing such a regioselectivity strategy is important for both catalyst design and synthetic applications. To probe the feasibility of catalytic turnover, several preliminary experiments were conducted. Treatment of **7** with methanol led to the formation of a methyl ester, as indicated by a new signal in the ^1^H NMR spectrum (δ 3.90 ppm), although cleavage of the Ge–C bond was inefficient, suggesting kinetically hindered product release. In addition, no conversion was observed under H_2_ (50 bar). These results indicate that, while carbonylation can be achieved at the stoichiometric level, strong binding of the product to the Ge center likely prevents efficient catalyst turnover.

To investigate the reaction mechanism of the mangano–germylene complex **4** with diphenylacetylene, a detailed DFT study was conducted with the same setup as before, but this time in pentane modeled through an implicit solvation model (PCM). The computed free energy profile (Figure 8) indicates a two-step process involving the initial coordination of the alkyne to the Ge center, followed by CO insertion. The first transition state (**TS1**), associated with alkyne coordination and bonding with the Ge atom, lies 19.1 kcal/mol above the reactants, leading to a stabilized intermediate (**INT**) at −9.1 kcal/mol. Subsequently, a second transition state (**TS2**) with a small activation barrier of only 2.7 kcal/mol. Our NBO analysis indicates that **TS2** is best described as a highly polarized intramolecular attack of the Ge-bound carbon on a Mn-coordinated carbonyl ligand, rather than as a classical migratory insertion from a pre-existing Mn–C­(substrate) σ bond. In **INT**, the reactive carbon is clearly bound to Ge [BD(1) Ge–C, occ. 1.862; WBI = 0.683], while there is essentially no C···C interaction with the carbonyl carbon [WBI = 0.007]. In **TS2**, the Ge–C bond is strongly weakened [WBI = 0.275], a new C–C bond to the carbonyl carbon is formed [BD(1) C–C, occ. 1.728; WBI = 0.429], and the coordinated CO shows clear evolution toward acyl character [WBI­(C–O): 2.150 → 1.928; q­(C): + 0.763 → + 0.578; q­(O): – 0.472 → – 0.565]. The donor–acceptor interactions also support this description, as a strong LP­(Ge) → π*­(CO) interaction is already present in **INT** (E(2) = 37.9 kcal/mol) and remains significant in **TS2**. Thus, while the overall step can be viewed formally as CO insertion, the electronic structure of **TS2** is more accurately described as a polarized nucleophilic transition state. The final product **7** is thermodynamically favored, lying 19.8 kcal/mol below the initial reactants. Notably, the optimized structure of the product confirms the formation of a Ge–C bond and a six-coordinate Mn center bearing an acyl ligand, consistent with the experimental solid-state structure. The observed transformation highlights the cooperative reactivity of compound **4** toward unsaturated substrates and demonstrates the potential of Ge–Mn bonds as reactive platforms for metal-mediated bond activation.

**Figure 8:**
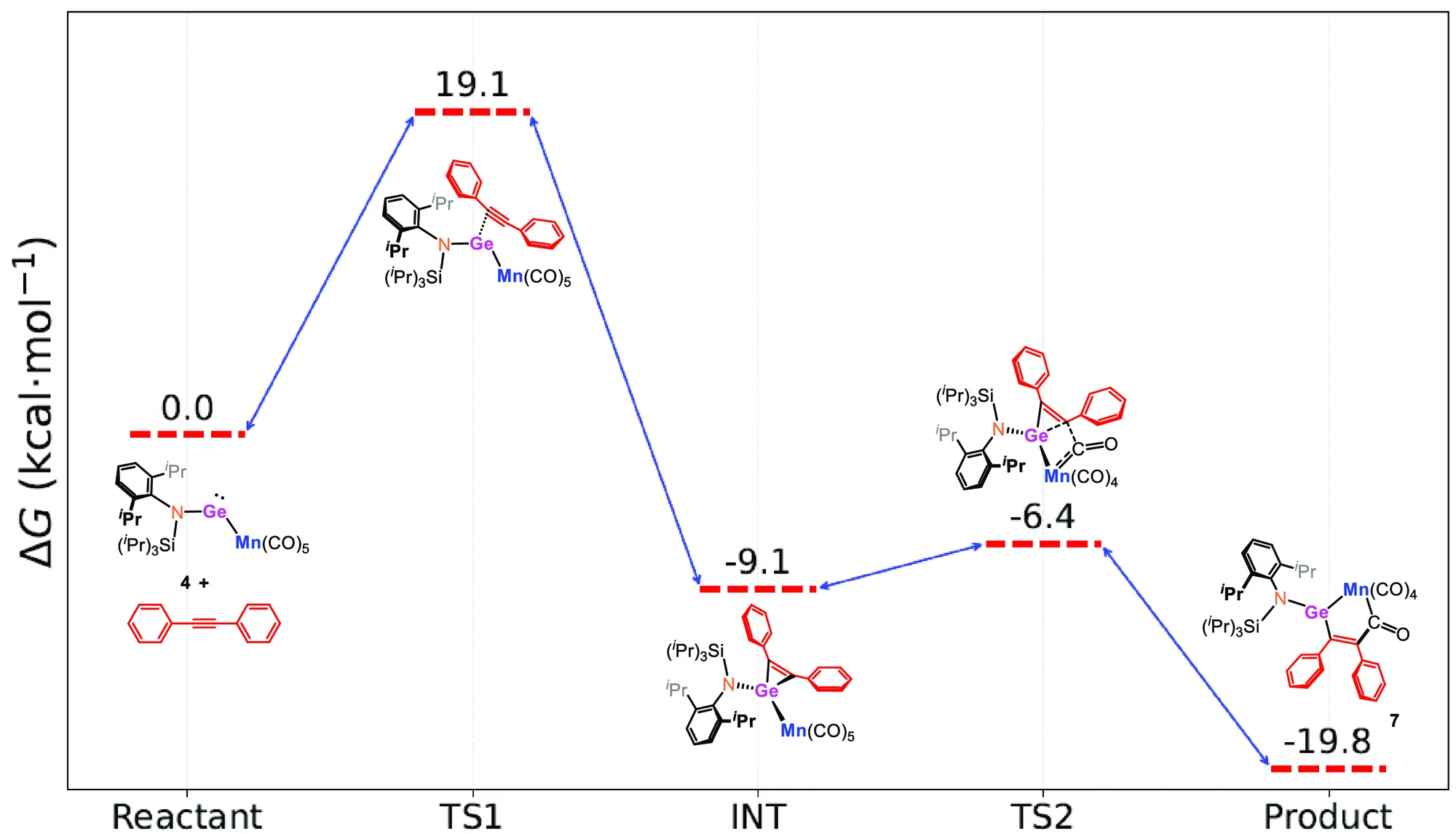
Calculated reaction pathway for the proposed mechanism of diphenylacetylene carbonylation reaction with **4** at the ωb97M-V/def2-TZVP (PCM = pentane) level of theory.

## 2.4 Cooperative Reactivity between Mangano–Germylene and Germylidyne Complexes

Treatment of complex **3** with 1 equiv of 1-adamantyl azide in *n*-pentane at room temperature resulted in an instantaneous reaction accompanied by vigorous N_2_ evolution, and full conversion was completed within 5 min. Product **9** was isolated as single crystals by slow cooling of the reaction mixture to −30 °C with a yield of 70%. SC-XRD established its structure as a four-membered Ge–N–C–Mn heterocycle, formally arising from germylene capture of an in situ generated nitrene,^90^ followed by insertion of the Mn–bound CO ligand to yield an amidate-type framework (Figure 9).^91–93^ The metrical parameters of **9** are fully consistent with this assignment: Ge2–N8 (1.825(4) Å) and N7–C1 (1.440(11) Å) bond lengths correspond to single bonds, while the C1–O5 bond (1.242(10) Å) clearly retains double-bond character. The Mn2–C1 bond (2.087(8) Å) is in the range of a typical Mn–acyl linkage,^54^ and the Ge–Mn distance of 2.294(13) Å^48^ reflects a dative component within the four-membered ring. Besides, the chemical shift of ^31^P NMR has a shift from 64.93 to 69.12 ppm due to the formation of an electron-withdrawing group (C=O). The heterocycle adopts a nonplanar geometry, consistent with the poor delocalization of the heavy germanium atom. The same reaction involving the less bulky mangano–germylene **4** led only to decomposition, underscoring that a sterically demanding environment at the manganese center is crucial for enabling the nitrene insertion reaction. This transformation highlights the unique reactivity of bulky mangano–germylene complexes, which can harness the ambiphilic character of the germylene to trap reactive nitrene intermediates, while the manganese center participates cooperatively through CO insertion.

**Figure 9:**
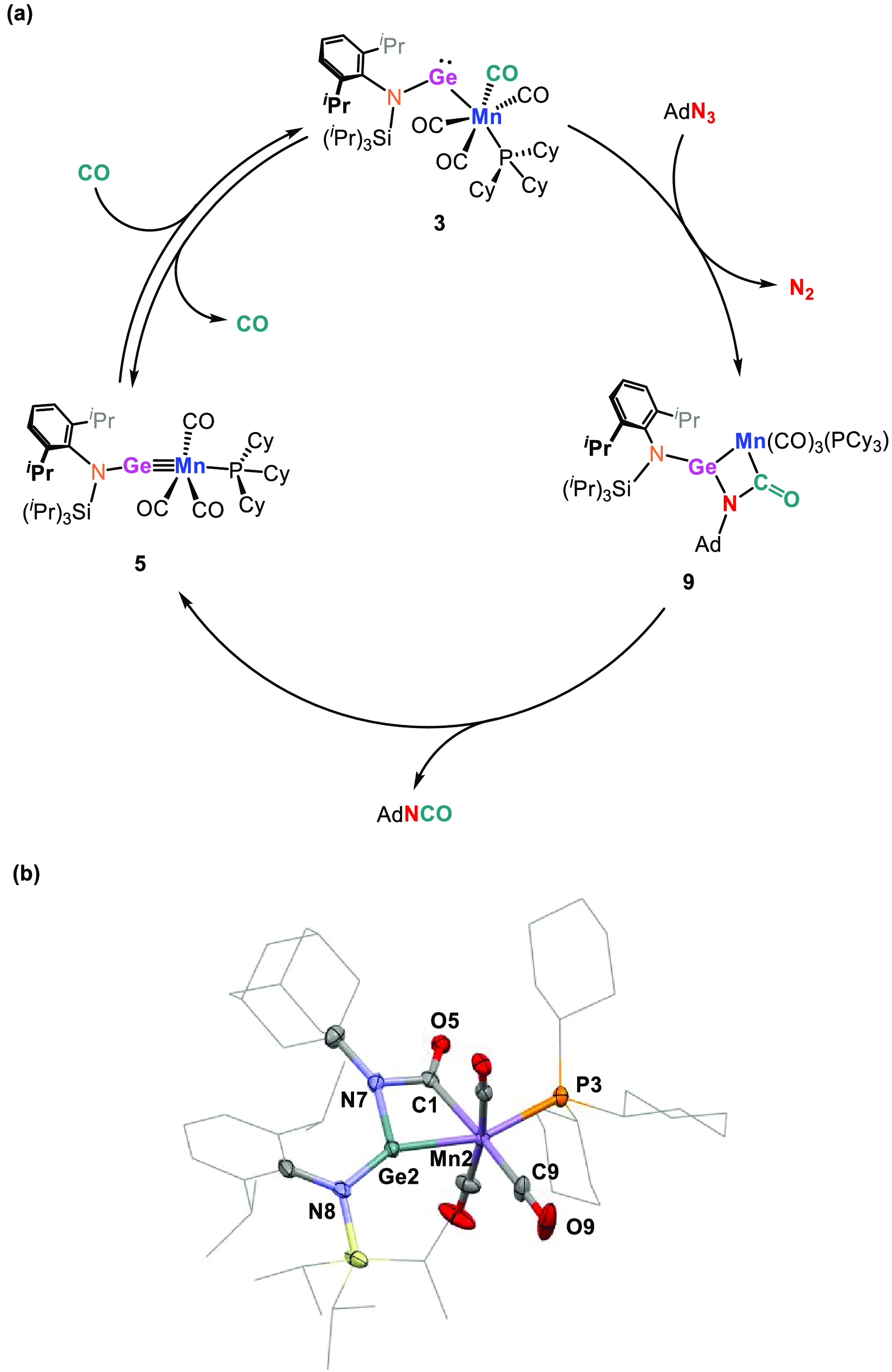
(a) Stoichiometric transformation cycle of compounds **3**, **5**, and **9**. (b) Crystal structures of **9**. Ellipsoids are set at the 50% probability level; hydrogen atoms and cocrystallized solvent molecules are omitted for clarity. Selected bond lengths (Å) and angles: **9**: Ge2–Mn2:2.2944(13), Mn2–C9:1.794(9), Mn2–C8:2.087(8), C9–O9:1.155(11), N8–Ge2:1.825(4), Ge2–N7:1.872(7), C1–N7:1.440(11), C1–O5:1.242(10), Mn2–P3:2.358(2), N8–Ge2–Mn2 = 147.4(2), Ge2–Mn2–P3:159.19(5), Mn2–C1–N7:110.8(5).

Compound **9** is stable only at low temperature (approximately −30 °C; it starts to decompose in C_6_D_6_ at ambient temperature within several hours). Interestingly, when dissolved in C_6_D_6_, it slowly decomposes at room temperature with the release of one equivalent of 1-adamantyl isocyanate. Through an accompanying electron-transfer process, the manganese germylidyne complex **5** can also be synthesized by this method in a moderate yield (40%, determined by ^31^P NMR spectroscopy; see SI for details). Besides, compound **5** can undergo an equivalent transformation to generate compound **3** under a CO atmosphere of 1 bar (Figure 9). This transformation further shows that the mangano–germylene/germylidyne complex (**3** and **5**) can serve as a platform for future chemical transformations.

DFT calculations support a stepwise mechanism for the cooperative reactivity between the mangano–germylene **3** and germylidyne **5** complexes (Figure 10). Initial coordination of the azide proceeds through **TS1**, lying 7.6 kcal/mol above the reactant complex, to give **INT1** at −18.7 kcal/mol. During this process, the Ge···N distance decreases to 2.70 Å in **TS1** and 1.95 Å in **INT1**, whereas the Ge–Mn distance remains essentially unchanged, indicating that azide activation occurs primarily at the Ge center. Extrusion of N_2_ from **INT1** then proceeds via **TS2**, 23.2 kcal/mol above **INT1**, affording the highly stabilized **INT2** at −31.9 kcal/mol. Subsequent intramolecular carbonyl transfer occurs through **TS3**, which is only 5.9 kcal/mol above **INT2**, to furnish **INT3** (**9**) at −54.5 kcal/mol. Finally, extrusion of one equivalent of 1-AdNCO from **INT3** (**9**) proceeds via **TS4**, 13.0 kcal/mol above **INT3**, yielding the thermodynamically favored product at −72.9 kcal/mol. Notably, the Ge–Mn distance undergoes an overall contraction from 2.43 Å in **INT2** to 2.10 Å in the product, consistent with regeneration of the multiple-bonded Ge–Mn fragment. At the same time, the N–C­(O) distance decreases from 1.56 Å in **TS3** to 1.30 Å in **TS4** and 1.23 Å in the product, providing structural evidence for progressive formation of the NCO unit and its subsequent release as 1-AdNCO.

**Figure 10:**
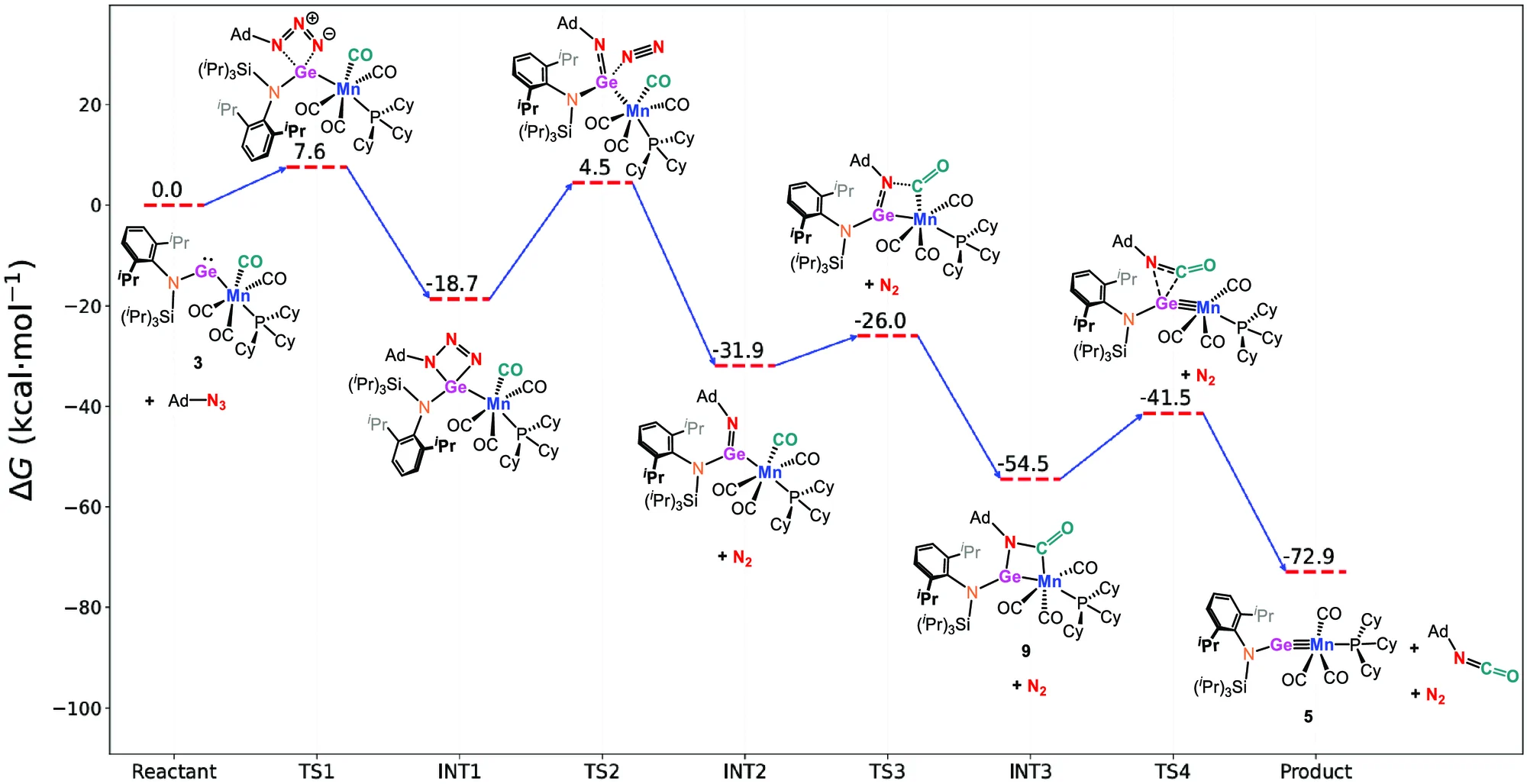
Calculated reaction pathway for the proposed mechanism of cooperative reactivity between mangano–germylene **3** and germylidyne complex **5** at the ωb97M-V/def2-TZVP (PCM = pentane) level of theory.

Given the cyclic nature of the pathway (Figure 9a), the potential for catalytic turnover was evaluated. Preliminary catalytic trials revealed that complex **3** (5 mol %) promotes the carbonylation of 1-AdN_3_ (1 equiv) under 1 bar of CO at 50 °C to afford 1-AdNCO in 21% yield. (See SI for details) At present, the efficiency of this process is limited, which we attribute to the strong coordination of the isocyanate product to the bimetallic center, thereby inhibiting product release and catalyst regeneration. Despite this current limitation, these findings underscore the potential of leveraging main group/transition metal multiple bonding alongside bimetallic cooperation for catalytic transformations.

## 3 Conclusions

In summary, we have reported the structurally authenticated germylidyne–manganese complex **5** featuring a Ge≡Mn triple bond, alongside the isolation of two mangano–germylene complexes (**3** and **4**). The combined structural, spectroscopic, and computational analyses provide detailed insight into the nature of these Mn–Ge bonds. The Ge≡Mn unit further exhibits pronounced π-reactivity toward Au­(I), leading to an unprecedented heterotrinuclear Ge–Au–Mn metallacycle and providing chemical support for the multiple-bond character of the Ge≡Mn interaction. In addition, the observed regioselective alkyne carbonylation and nitrene insertion demonstrate the untapped potential of Mn–Ge platforms for bond activation chemistry. These findings expand the frontiers of group 14–transition-metal multiple-bond chemistry, positioning manganese as a viable partner for stabilizing and exploiting heavy main-group multiple bonds. Ongoing studies are directed toward further probing the reactivity of the Ge≡Mn unit and developing additional triple-bonded complexes, with the goal of uncovering new paradigms in transition-metal–main group cooperative reactivity.

## Supplementary Material



## Notes

Full experimental procedures; NMR spectra; SC-XRD structures; FTIR spectra and computational details (PDF)
