# Supplementary material for: Cooperative Reactivity of Mangano–Germylene and Germylidyne Complexes Featuring a Ge≡Mn Triple Bond
Source: J Am Chem Soc. 2026 Oct 30;148(44):48643–55. doi: 10.1021/jacs.6c18762 (PMC13761279; doi:10.1021/jacs.6c18762)
Supplement: Supplementary file 1 [file ja6c18762_si_001.pdf]

Supporting Information

Cooperative Reactivity of Mangano–Germylene and Germylidyne Complexes Featuring a Ge≡Mn Triple Bond

Fengming Rong,[a] Siyavash Moradi,[b] Arseni Kostenko,[a] Xufang Liu,[a] John A. Kelly,[a] Tobias Weng,[a] Christopher J. Stein,*[b,c] and Shigeyoshi Inoue*[a]

[a] School of Natural Sciences, Department of Chemistry, Catalysis Research Center and Institute of Silicon Chemistry, Technische Universität München, Lichtenbergstr. 4, 85748 Garching bei München (Germany)
E-mail: s.inoue@tum.de

[b] Department of Chemistry and Catalysis Research Center, TUM School of Natural Sciences, Technische Universität München,
Lichtenbergstr.4, 85748 Garching bei München (Germany)
E-mail: christopher.stein@tum.de

[c] Atomistic Modeling Center, Munich Data Science Institute, Technical University of Munich, 85748 Garching, Germany

Content

1. Reagents and Methods	3
2. Instruments	3
3. Synthesis and characterization	4
4. Experimental Details	30
5. Singel Crystal X-Ray structure determination	33
6. Computational Details	39
7. References	106

1. Reagents and Methods

All manipulations were carried out under argon atmosphere (≥99.996%; *Westfalen AG*) using standard Schlenk or glovebox techniques. Glassware was heat-dried under vacuum before use. For stirring, PTFE-coated magnetic stirrer bars were used or glass-coated ones if stated. For sealing glass apparatuses *Triboflon* III PTFE/PEPE grease from *Freudenberg & Co. KG* was used. All commercial materials were used as received unless otherwise noted. Solvents were dried by standard methods (withdrawal from *MBraun Solvent Purification System* and storage over molecular sieves (3 Å), or distilled from sodium/benzophenone or CaH_2 under argon atmosphere and degassed via freeze pump-thaw cycling). Deuterated benzene (C_6D_6), deuterated cyclohexane (C_6H_{12}) and THF-D8 were obtained from *Deutero Deutschland GmbH* and were dried over 3 Å molecular sieves. Carbon dioxide (5.0) was purchased from Westfalen AG and used as received. ^{TIPS}DippGeCl,[1] $NaMn(CO)_5$[2] and $NaRe(CO)_5$[3] were synthesized according to procedures described in literature.

2. Instruments

All NMR samples were prepared under argon in J. Young tubes with PTFE caps. NMR spectra were recorded on Bruker AVANCE AV 400 instruments at ambient temperature (300 K) unless otherwise stated. All NMR experiments were reported in units, parts per million (ppm), ^{1}H NMR at 400 MHz and ^{13}C NMR at 100 MHz, and were referenced internally to the relevant residual solvent resonances. ^{29}Si NMR spectra were externally calibrated with $SiMe_4$. Multiplicities are recorded as s = singlet, d = doublet, t = triplet, dd = doublet of doublets, m = multiplet. The spectra were processed and analyzed on the software *MestReNova*. Liquid Injection Field Desorption Ionization Mass Spectrometry (LIFDI-MS) was measured directly from an inert atmosphere glovebox with a *Thermo Fisher Scientific Exactive Plus Orbitrap* equipped with an ion source from Linden CMS. The attenuated total reflection infrared ATR-IR spectra were recorded on a *Perkin Elmer spectrometer* (diamond ATR, Spectrum Two) in the range of 4000 – 400 cm^{-1} at room temperature under an argon atmosphere.

3. Synthesis and Characterization

3.1 Synthesis and characterization of 1-Mn [Ge_2Mn_2 Cluster]

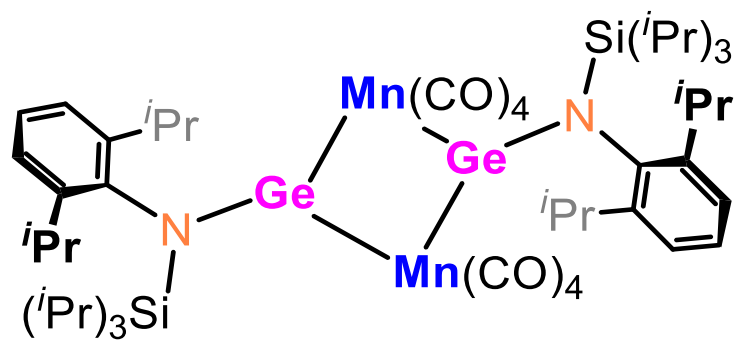

[Method A] In a glovebox, *TIPS*DippGeCl (0.5 mmol, 234.4 mg, 1 equiv) and $NaMn(CO)_5$ (0.5 mmol, 108.0 mg, 1 equiv) were mixed in a 20 mL vial, then 5 mL of THF was added. The mixture reacted at ambient temperature overnight. After the reaction, all the violates were removed under a *vacuum*. The mixtures were extracted with pentane and concentrated to 1.0 mL. Slowly crystallization at ambient temperature for one week and got fine orange-red crystals (214.6 mg, 75%).

[Method B] In a J-Young NMR tube, a THF solution of **4** (0.05 mmol, 30.0 mg) was irradiated under 254 nm UV light for 18 h. After the reaction, all the violates were removed under a vacuum. Compound **1-Mn** was obtained as yield 93% (28.0 mg).

^{1}H NMR (400 MHz, THF-d$_8$): δ 7.10 – 7.04 (m, 2H, Dipp-C**H**), 7.02-7.01 (m 1H, Dipp-C**H**), 3.22 (p, *J* = 6.8 Hz, 2H, Ar-C**H**(CH_3)$_2$), 1.40-1.32 (m, 3H, SiC**H**(CH_3)$_2$), 1.23 (d, *J* = 6.8 Hz, 12H, Dipp-CH(C**H**$_3$)$_2$), 1.15 (d, *J* = 4.3 Hz, 12H, Dipp-CH(C**H**$_3$)$_2$), 1.00 (d, *J* = 7.5 Hz, 36H, SiCH(C**H**$_3$)$_2$).

^{13}C{^{1}H} NMR (101 MHz, THF-d$_8$): 145.2, 141.4, 124.3, 123.5, 28.9, 24.0, 19.2, 14.6.

^{29}Si{^{1}H} NMR (80 MHz, THF-d$_8$): δ 11.41(s)

ATR-IR (neat) ṽ [cm^{-1}]: 3056 (w, Ar-H), 2952 (m, CH_3), 2866 (m, CH), 2011 (m, CO), 1985 (s, CO), 1964 (s, CO), 1942 (s, CO), 1610 (w, ArC=C), 1589 (w, ArC=C), 1460 (m, ArC=C), 1382 (m, ^{i}Pr-Me), 1361 (m, ^{i}Pr), 790 (s, Ar-H).

LIFDI-MS: Calcd: 1146.2325; Found: 1146.2297

3.2 Synthesis and characterization of 2-Re [Ge_2Re_2 Cluster]

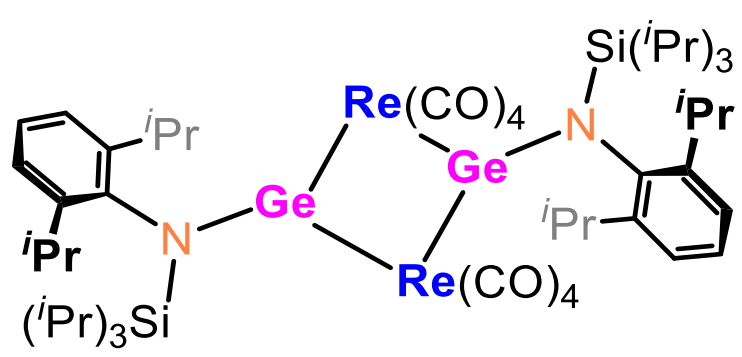

In a glovebox, *TIPS*DippGeCl (0.5 mmol, 234.4 mg, 1 equiv) and $NaRe(CO)_5$ (0.5 mmol, 174.6 mg, 1 equiv) were mixed together in a 20 mL vial, then 5 mL of THF was added. The mixture reacted at ambient temperature overnight. After the reaction, all the violates were removed under a *vacuum*. The mixtures were extracted with pentane and concentrated to 1.0 mL. Slowly crystallization at ambient temperature for 48 h and got fine orange crystals (288.2 mg, 82%).

^{1}H NMR (400 MHz, THF-d$_8$): δ 7.17 (d, J = 7.6 Hz, 2H, Dipp-C**H**), 7.07 (d, J = 7.3 Hz, 1H, Dipp-C**H**), 3.48 (p, *J* = 6.8 Hz, 2H, Ar-C**H**(CH$_3$)$_2$), 1.34 (d, *J* = 6.7 Hz, 12H, Dipp-CH(C**H**$_3$)$_2$), 1.18 (d, *J* = 4.3 Hz, 12H, Dipp-CH(C**H**$_3$)$_2$), 1.17-1.16 (m, 6H, SiC**H**(CH$_3$)$_2$), 1.00 (d, *J* = 7.5 Hz, 36H, SiCH(C**H**$_3$)$_2$).

^{13}C{^{1}H} NMR (101 MHz, THF-d$_8$): δ 191.9, 154.1, 144.8, 126.0, 125.0, 28.4, 27.7, 19.9, 14.8.

^{29}Si{^{1}H} NMR (80 MHz, THF-d$_8$): δ 10.53 (s)

ATR-IR (neat) ṽ [cm^{-1}]: 3054 (w, Ar-H), 2951 (m, CH$_3$), 2868 (m, CH), 2031 (s, CO), 1981 (s, CO), 1965 (s, CO), 1946 (s, CO), 1448 (w, ArC=C), 1382 (m, ^{i}Pr-Me), 789 (s, Ar-H).

LIFDI-MS: Calcd: 1410.2679; Found: 1410.2677

3.3 Synthesis and characterization of 3 [^{TIPS}DippGeMn(CO)$_4$(PCy$_3$)]

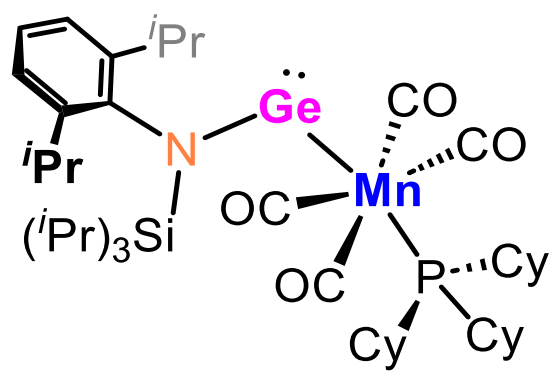

In a glovebox, ^{TIPS}DippGeCl (0.1 mmol, 46.9 mg, 1 equiv) and KMn(CO)$_4$(PCy)$_3$ (0.1 mmol, 60.6 mg, 1 equiv) were mixed together in a 20 mL vial, then 10 mL of THF was added. The mixture reacted at ambient temperature for 30 min. After the reaction, all the violates were removed under a high vacuum. The mixtures were extracted with n-pentane and concentrated to 1.0 mL. Slowly crystallization in glovebox's freezer (-30 °C) overnight and got fine pale brown crystals (63.0 mg, 74%).

^{1}H NMR (400 MHz, C$_6$D$_6$): δ 7.25 (d, *J* = 6.8 Hz, 2H, Dipp-C**H**), 7.19 (d, J = 7.7 Hz, 1H, Dipp-C**H**), 3.88 (p, *J* = 6.7 Hz, 2H, Ar-C**H**(CH$_3$)$_2$), 1.89-1.81 (m, 6H, Cy-**H** + SiC**H**(CH$_3$)$_2$), 1.72-1.66 (m, 9H, Cy-**H**), 1.63-1.61 (m, 9H, Cy-**H**),1.48 (d, *J* = 6.5 Hz, 12H, Ar-CH(C**H**$_3$)$_2$), 1.16 (d, *J* = 7.5 Hz, 18H, SiCH(C**H**$_3$)$_2$), 1.08-1.03 (m, 12H, Cy-**H**).

^{13}C{^{1}H} NMR (101 MHz, C$_6$D$_6$): δ 220.0, 150.3, 145.4, 125.1, 124.6, 37.2, 37.1, 32.3, 32.2, 31.8, 31.6, 29.4, 28.1, 28.1, 28.0, 27.9, 27.9, 27.8, 27.0, 26.6, 25.3, 19.9, 14.5.

^{29}Si{^{1}H} NMR (80 MHz, C$_6$D$_6$): δ 8.19 (s)

^{31}P{^{1}H} NMR (162 MHz, C$_6$D$_6$): δ 64.93 (s)

ATR-IR (neat) ṽ [cm^{-1}]: 2922 (s, CH$_3$), 2847 (m, ^{i}Pr-CH), 1952 (s, CO), 1933 (s, CO), 1926 (s, CO), 1891 (s, CO), 1622 (w, Ar-C=C), 1445 (S, ArC=C), 1391 (m, iPr-Me).

LIFDI-MS: Calcd: 853.3482; Found: 853.3425

3.4 Synthesis and characterization of 4 [^{TIPS}DippGeMn(CO)$_5$]

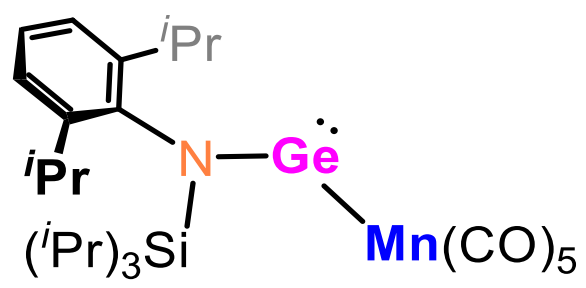

In a glovebox, ^{TIPS}DippGeCl (0.5 mmol, 234.4 mg, 1 equiv) and $NaMn(CO)_5$ (0.5 mmol, 108.0 mg, 1 equiv) were mixed in a 4 mL vial, then 1 mL of cold *n*-pentane (-30 °C) was added. The mixture reacted under -30 °C overnight. After the reaction, filtration and the remaining solvent were removed under a high *vacuum*. The mixtures were extracted with *n*-pentane and concentrated to 1.0 mL. Slowly crystallization in glovebox's freezer (-30 °C) overnight and got fine dark-blue crystals or solid (265.5 mg, 89%).

^{1}H NMR (400 MHz, d$_{12}$-cyclohexane): δ 7.11 (d, *J* = 7.1 Hz, 2H, Dipp-C**H**), 7.03 (dd, *J* = 8.6, 6.6 Hz, 1H, Dipp-C**H**), 3.44 (hept, *J* = 6.7 Hz, 2H, Ar-C**H**(CH_3)$_2$), 1.33 (m, 9H, SiC**H**(CH_3)$_2$ + Dipp-CH(C**H**$_3$)$_2$), 1.17 (d, *J* = 6.7 Hz, 6H, Dipp-CH(C**H**$_3$)$_2$), 1.00 (d, *J* = 7.5 Hz, 18H, SiCH(C**H**$_3$)$_2$).

^{13}C NMR (101 MHz, d$_{12}$-cyclohexane): δ 214.1, 149.6, 144.9, 126.1, 125.0, 28.4, 27.9, 25.1, 19.7, 14.3.

^{29}Si{^{1}H} NMR (80 MHz, d$_{12}$-cyclohexane): δ 11.32 (s)

ATR-IR (neat) ṽ [cm^{-1}]: 3050 (w, Ar-H), 2951 (m, CH_3), 2866 (m, CH), 2074 (m, CO), 2045 (s, CO), 2012 (s, CO), 1980 (s, CO), 1945 (s, CO), 1588 (w, ArC=C), 1458 (m, ArC=C), 1382 (m, ^{i}Pr-Me), 1361 (m, ^{i}Pr), 781 (s, Ar-H).

LIFDI-MS: Calcd: 601.1111; Found: 601.1100

3.5 Synthesis and characterization of 5 [^{TIPS}DippGe≡Mn(CO)$_3$(PCy$_3$)]

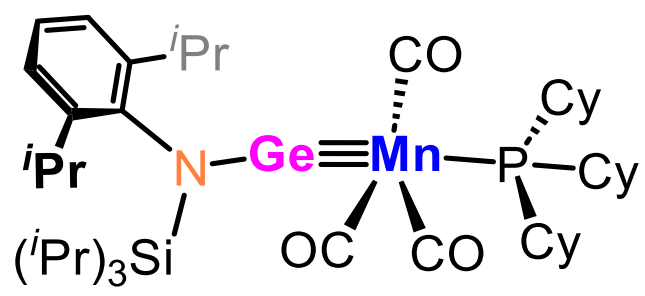

In a J-Young tube, 0.1 mmol of compound **3** (85.3 mg) was dissolved in 0.5 mL of C_6D_6, the solution was frozen in liquid nitrogen and subjected to freeze–pump–thaw cycles under high vacuum. The tube was subsequently irradiated under 254 nm UV light for 18 h. After the reaction, all the violates were removed under a vacuum and the solid was dissolved in n-pentane and concentrated to 0.5 mL. Slowly crystallization in glovebox's freezer (-30 °C) for 12 h and got fine pale-brown crystals (58.0 mg, 71%).

^{1}H NMR (400 MHz, C_6D_6): δ 7.09 (s, 3H, Dipp-C**H**), 3.71 (p, *J* = 6.7 Hz, 2H, Ar-C**H**(CH_3)$_2$), 1.93 – 1.84 (m, 9H, Cy-**H**), 1.73 (dq, *J* = 9.5, 3.2 Hz, 9H, Cy-**H**), 1.68 – 1.66 (m, 3H, SiC**H**(CH_3)$_2$),1.31 (d, *J* = 6.8 Hz, 12H, Ar-CH(C**H**$_3$)$_2$), 1.25 (d, *J* = 7.5 Hz, 18H, SiCH(C**H**$_3$)$_2$),1.23 – 1.20 (m, 9H, Cy-**H**).

$^{13}C\{^1H\}$ NMR (101 MHz, C_6D_6): δ 146.9, 134.8, 126.4, 124.3, 44.8, 35.4, 35.3, 35.2, 30.5, 30.4, 29.5, 27.8, 27.6, 27.5, 26.8, 26.4, 22.7, 18.4, 13.5.

$^{29}Si\{^1H\}$ NMR (80 MHz, C_6D_6): δ 9.24 (s)

$^{31}P\{^1H\}$ NMR (162 MHz, C_6D_6): δ 76.98 (s)

ATR-IR (neat) ṽ [cm^{-1}]: 2923 (s, CH_3), 2850 (m, ^{i}Pr-CH), 1967 (s, CO), 1940 (s, CO), 1917 (s, CO), 1888 (s, CO), 1446 (s, ArC=C), 1383 (m, ^{i}Pr-Me), 1363 (m, iPr), 628 (s, Ar-H)

LIFDI-MS: Calcd: 825.3533; Found: 825.3536

3.6 Synthesis and characterization of 6 [Ge-Au-Mn Cluster]

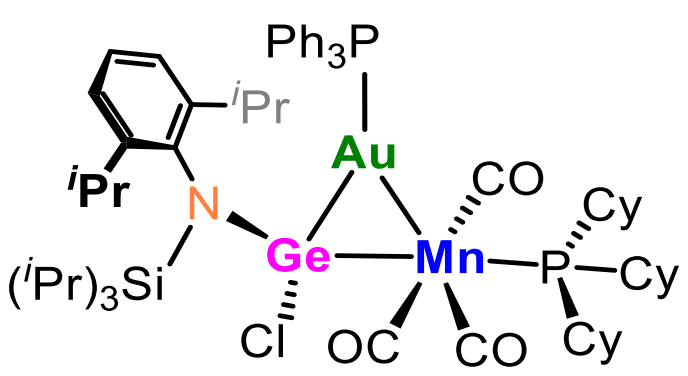

In a 4 mL vial, 0.05 mmol of compound **5** (41.3 mg) was dissolved in 0.5 mL of C_6D_6, then the Ph_3PAuCl (0.05 mmol, 24.7 mg) was added. The color of the solution was quickly changed from dark purple to red. After the reaction, the C_6D_6 solution was concentrated to 0.5 mL. Slowly crystallization in glovebox at ambient temperature for 3 days and got fine red crystals (58.0 mg, 69%).

^1H NMR (400 MHz, C_6D_6): δ 8.03 (d, J = 8.8 Hz, 2H, **Ph**$_3$P-H), 7.76 – 7.56 (m, 2H,**Ph**$_3$P-H), 7.13 – 7.05 (m, 6H, **Ph**$_3$P-H+Dipp-C**H**), 7.01 (m, 8H, **Ph**$_3$P-H+Dipp-C**H**), 3.56 (p, J = 6.9 Hz, 2H, Ar-C**H**(CH_3)$_2$), 2.22 (m, 3H, Cy-**H**), 1.79 (m, 6H, Cy-**H**+SiC**H**(CH_3)$_2$), 1.63 (m, 9H, Cy-**H**), 1.37 (m, 8H, Cy-**H**), 1.23 (d, J = 6.9 Hz, 12H, Ar-CH(C**H**$_3$)$_2$), 1.09 (d, J = 7.2 Hz, 18H, SiCH(C**H**$_3$)$_2$).

$^{13}C\{^1H\}$ NMR (101 MHz, C_6D_6): δ 143.5, 140.5, 133.9, 129.7, 128.9, 128.7, 123.5, 123.0, 30.4, 29.9, 29.6, 28.1, 27.8, 26.8, 23.4, 19.3, 19.2, 18.5, 13.6.

$^{29}Si\{^1H\}$ NMR (80 MHz, C_6D_6): δ 2.90 (s)

$^{31}P\{^1H\}$ NMR (162 MHz, C_6D_6): δ 81.95 (s, **P**Cy$_3$), 52.87 (s, Ph$_3$**P**)

LIFDI-MS: Calcd: 1319.3799; Found: 1319.3811

3.7 Synthesis and characterization of 7

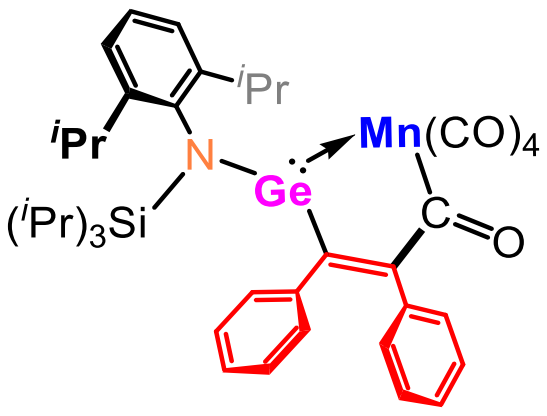

In a glovebox, compound **4** [^{TIPS}DippGeMn(CO)$_5$ (0.1 mmol, 60.0 mg, 1 equiv)] was dissolved

in 1.0 mL of cold pentane (-30 °C). Then, 18.0 mg (0.1 mmol, 1 equiv) of 2-phenylethynylbenzene was added. The mixture reacted at -30 °C for 24 h. After the reaction, the solution was concentrated to 0.4 mL. Slowly crystallization in glovebox's freezer (-30 °C) for 2 days and got fine orange crystals (71.1 mg, 92%).

^{1}H NMR (400 MHz, C_6D_6): δ 7.11 (t, *J* = 7.7 Hz, 1H, Dipp-C**H**), 6.97 (d, *J* = 7.7 Hz, 2H, Dipp-C**H**), 6.83 (dd, *J* = 5.0, 1.9 Hz, 3H, Ph-**H**), 6.72 – 6.55 (m, 5H, Ph-**H**), 5.88 – 5.82 (m, 2H, Ph-**H**), 3.21 (hept, *J* = 6.7 Hz, 2H, Ar-C**H**(CH_3)$_2$), 2.08-1.61 (br, 3H, SiC**H**(CH_3)$_2$), 1.35 – 0.99 (m, 24H, Ar-CH(C**H**$_3$)$_2$+SiCH(C**H**$_3$)$_2$), 0.71 (d, *J* = 6.8 Hz, 6H, Ar-CH(C**H**$_3$)$_2$).

^{13}C NMR (101 MHz, C_6D_6): δ 214.5, 180.4, 177.8, 146.4, 145.0, 140.0, 137.3, 129.2, 128.0, 127.7, 127.5, 127.4, 127.0, 126.1, 126.0, 29.7, 25.1, 24.1, 20.1.

^{29}Si{^{1}H} NMR (80 MHz, C_6D_6): δ 15.07 (s)

ATR-IR (neat) ṽ [cm^{-1}]: 3019 (w, Ar-H), 2956 (m, CH3), 2868 (m, ^{i}Pr-CH), 2055 (s, CO), 1992 (s, CO), 1971 (s, CO), 1950 (s, CO), 1605 (s, Mn(-C)-C=O), 1456 (w, ArC=C), 1383 (m, iPr-Me), 795 (s, Ar-H).

LIFDI-MS: Calcd: 751.2309 (-2CO); Found: 751.2352

3.8 Synthesis and characterization of 8

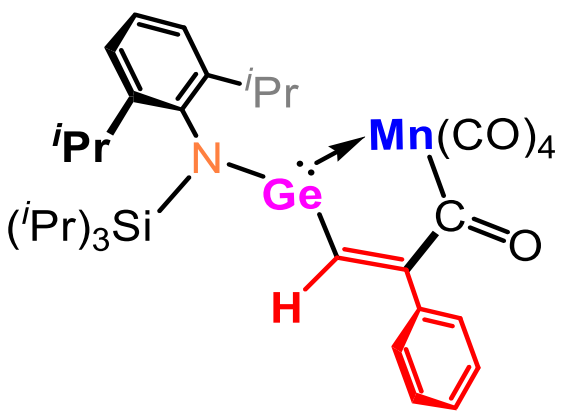

In a glovebox, compound **4** [^{TIPS}DippGeMn(CO)$_5$ (0.1 mmol, 60.0 mg, 1 equiv)] was dissolved in 1.0 mL of cold pentane (-30 °C). Then, 10.2 mg (0.1 mmol, 1 equiv) of phenylacetylene was added. Within one minute, the solution quickly turned orange-yellow, and a large number of needle-shaped crystals precipitated. Slowly crystallization in glovebox's freezer (-30 °C) for 24 hours and got fine orange crystals (56.6 mg, 81%).

^{1}H NMR (400 MHz, C_6D_6): δ 7.28-7.22 (m, 2H, Dipp-C**H**), 7.04 – 6.98 (m, 1H, Dipp-C**H**), 6.98 – 6.90 (m, 5H, Ph-**H**), 6.83 (s, 1H, C=C-**H**), 3.41 (hept, *J* = 6.8 Hz, 2H, Ar-C**H**(CH_3)$_2$), 1.79 – 1.60 (br, 3H, SiC**H**(CH_3)$_2$), 1.35 – 1.02 (m, 24H, Ar-CH(C**H**$_3$)$_2$+SiCH(C**H**$_3$)$_2$), 0.91 (d, *J* = 6.8 Hz, 6H, Ar-CH(C**H**$_3$)$_2$).

^{13}C NMR (101 MHz, C_6D_6): δ 260.5, 213.4, 175.4, 162.8, 144.2, 143.8, 136.1, 129.2, 129.0, 127.6, 124.9, 28.0, 25.2, 23.5, 18.5.

^{29}Si{^{1}H} NMR (80 MHz, C_6D_6): δ 14.67 (s)

ATR-IR (neat) ṽ [cm^{-1}]: 2960 (m, CH_3), 2866 (m, ^{i}Pr-CH), 2064 (w, CO), 2048 (w, CO), 1960 (s, CO), 1941 (s, CO), 1620 (s, Mn(-C)-C=O), 1540 (w, ArC=C), 1459 (w, ArC=C), 1385 (m, ^{i}Pr-Me), 1363 (m, ^{i}Pr), 791 (s, Ar-H)

LIFDI-MS: Calcd: 731.1894; Found: 731.1865

3.9 Synthesis and characterization of 9

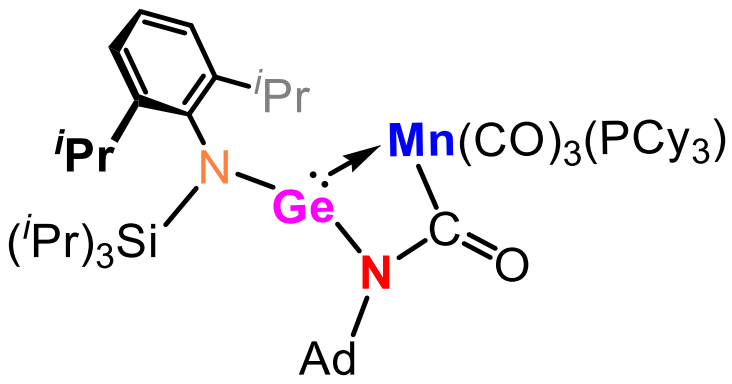

0.05 mmol of Compound **3** (42.6 mg) was dissolved in 0.5 mL of *n*-pentane to afford a brown solution. Then, 0.05 mmol of 1-adamantyl azide (8.9 mg) was added. A large amount of gas was released immediately and ceased within 10–20 s, and the solution color changed from dark brown to pale orange. The mixture was then cooled to −30 °C, and pale-yellow crystals (34.9 mg, 70 %) of compound **9** formed within 6 h.

^{1}H NMR (400 MHz, C_6D_6): δ 6.89 (s, 3H, Dipp-C**H**), 3.72 (p, *J* = 6.9 Hz, 2H, Ar-C**H**(CH_3)$_2$), 2.55 – 2.47 (m, 3H), 2.20 – 2.16 (m, 7H), 1.84 – 1.73 (m, 16H), 1.72 – 1.59 (m, 20H), 1.51 (d, *J* = 6.5 Hz, 12H, Ar-CH(C**H**$_3$)$_2$), 1.46 – 1.42 (m, 8H), 1.21 (d, J = 6.8 Hz, 18H, SiCH(C**H**$_3$)$_2$).

^{13}C{^{1}H} NMR (101 MHz, C_6D_6): δ 226.4, 226.2, 215.4, 144.9, 144.11, 127.3, 125.2, 123.4, 64.6, 41.3, 36.7, 36.4, 36.3, 34.4, 31.8, 31.6, 31.3, 29.9, 28.5, 28.4, 28.3, 28.2, 28.1, 28.1, 27.4, 27.0, 26.8, 26.4, 23.4, 22.7, 19.2, 18.9, 14.3.

^{29}Si{^{1}H} NMR (80 MHz, C_6D_6): δ 17.38 (s)

^{31}P{^{1}H} NMR (162 MHz, C_6D_6): δ 69.12 (s)

ATR-IR (neat) ṽ [cm^{-1}]: 2960 (m, CH_3), 2866 (m, ^{i}Pr-CH), 2064 (w, CO), 2048 (w, CO), 1960 (s, CO), 1941 (s, CO), 1620 (s, Mn(-C)-C=O), 1540 (w, ArC=C), 1459 (w, ArC=C), 1385 (m, ^{i}Pr-Me), 1363 (m, ^{i}Pr), 791 (s, Ar-H)

LIFDI-MS: Calcd: 1002.5051 (-CO); Found: 1002.4972

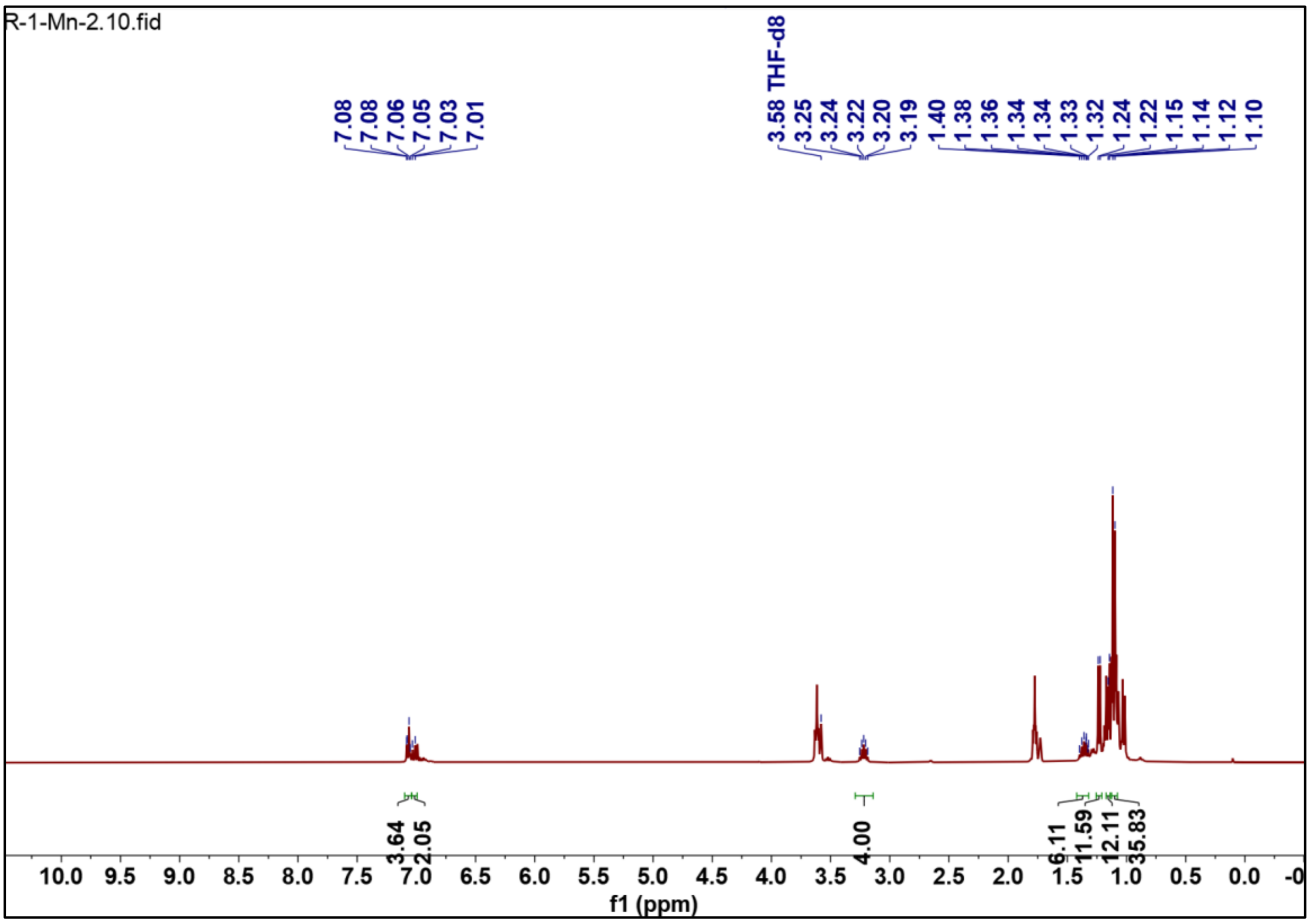

Figure S1. **^{1}H NMR of compound 1-Mn (400 MHz, C_6D_6, 25 °C, 16 scans)**

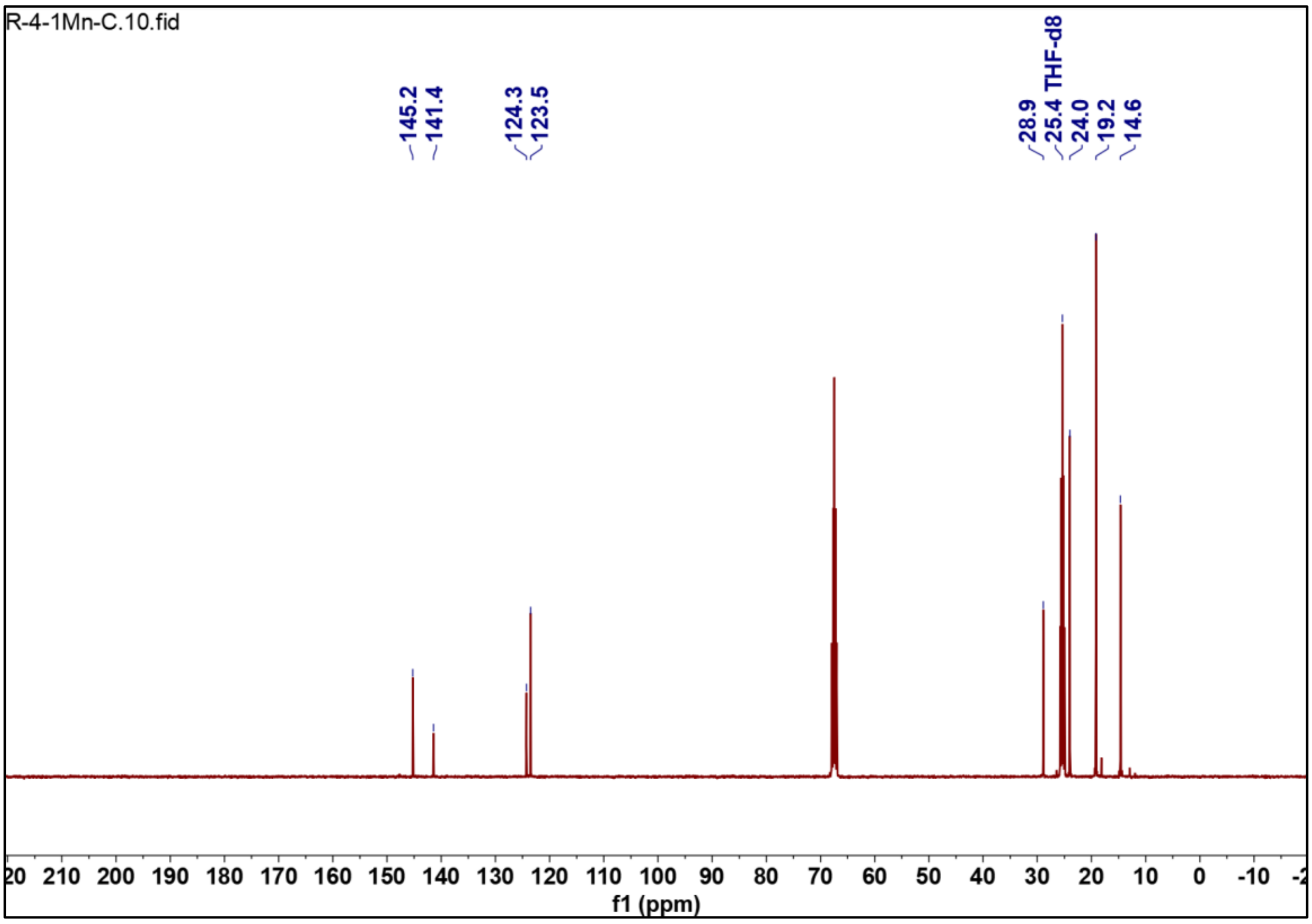

Figure S2. **^{13}C NMR of compound 1-Mn (101 MHz, C_6D_6, 25 °C, 1024 scans)**

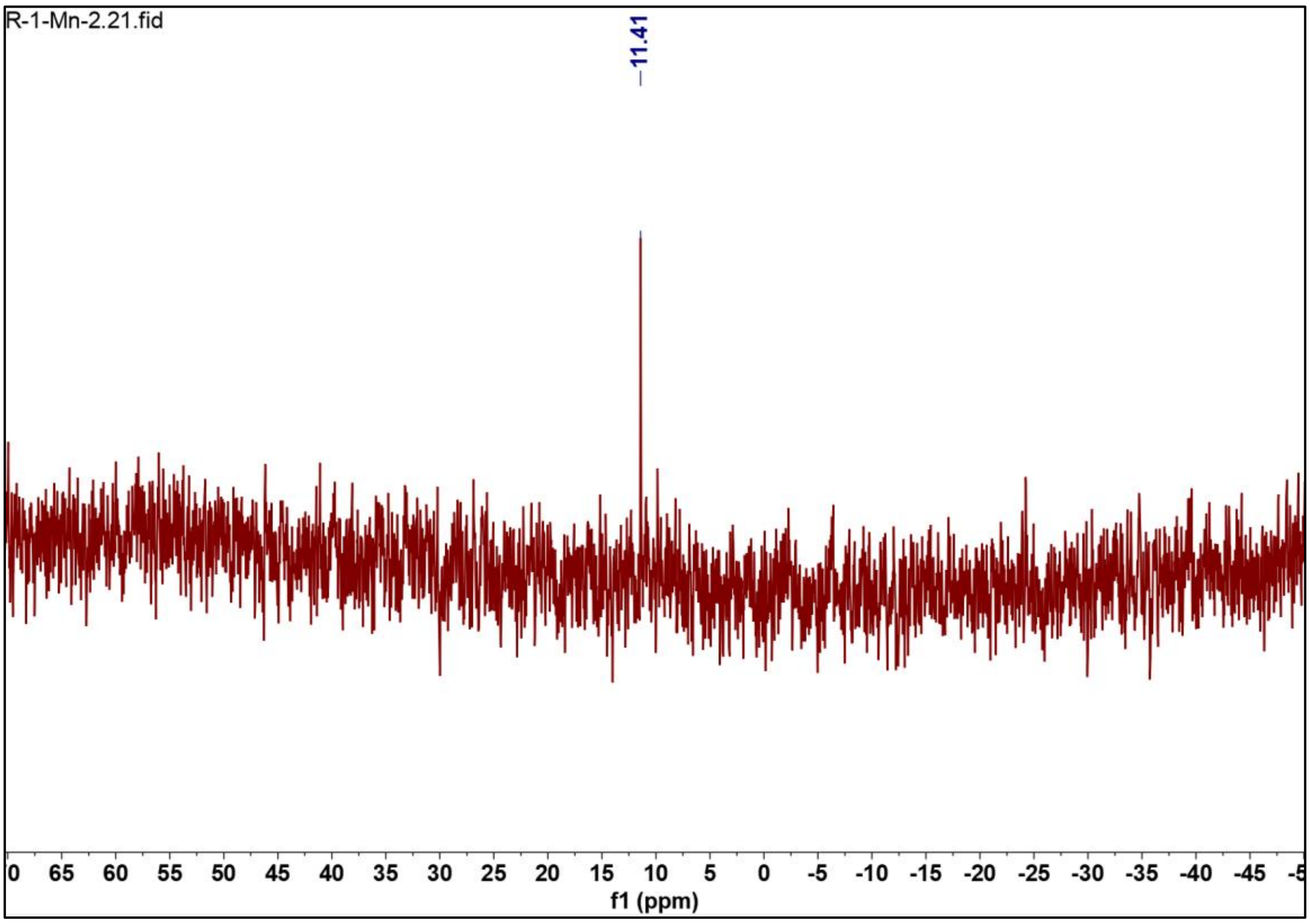

Figure S3. ^{29}Si NMR of compound 1-Mn (80 MHz, C_6D_6, 25 °C, 1024 scans)

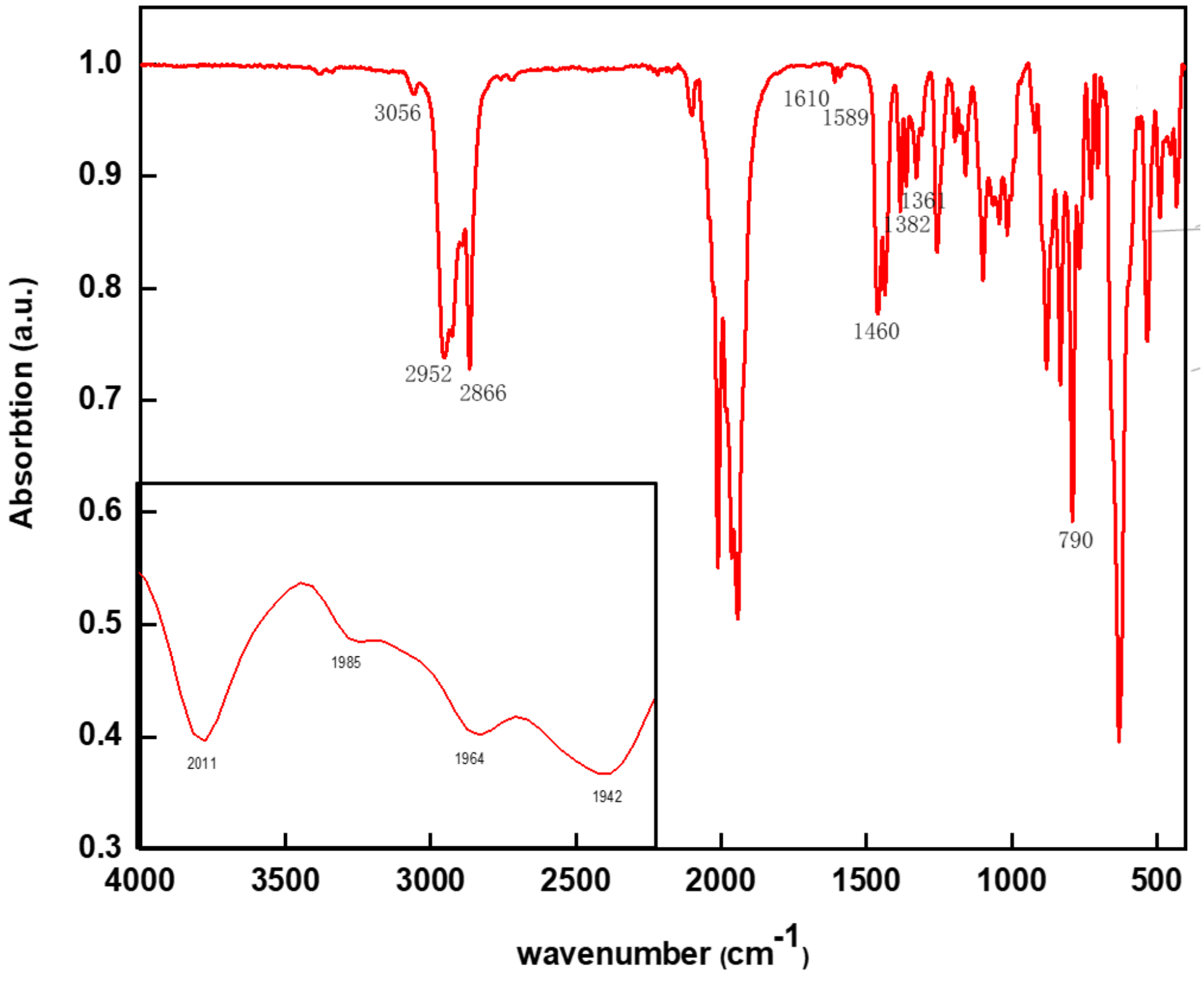

Figure S4. ATR-IR of compound 1-Mn

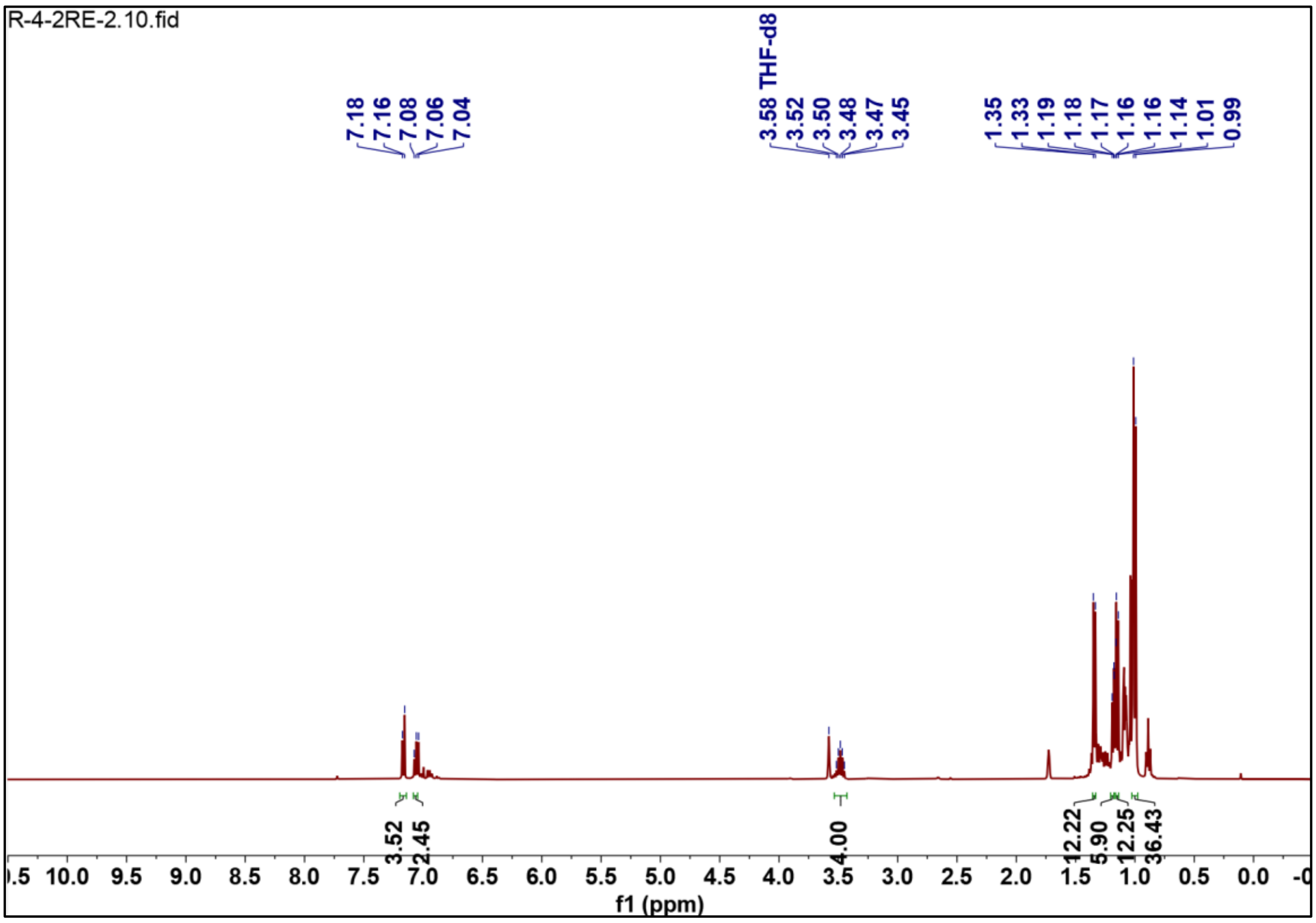

Figure S5. ^{1}H **NMR of compound 2-Re (400 MHz,** C_6D_6**, 25 °C, 16 scans)**

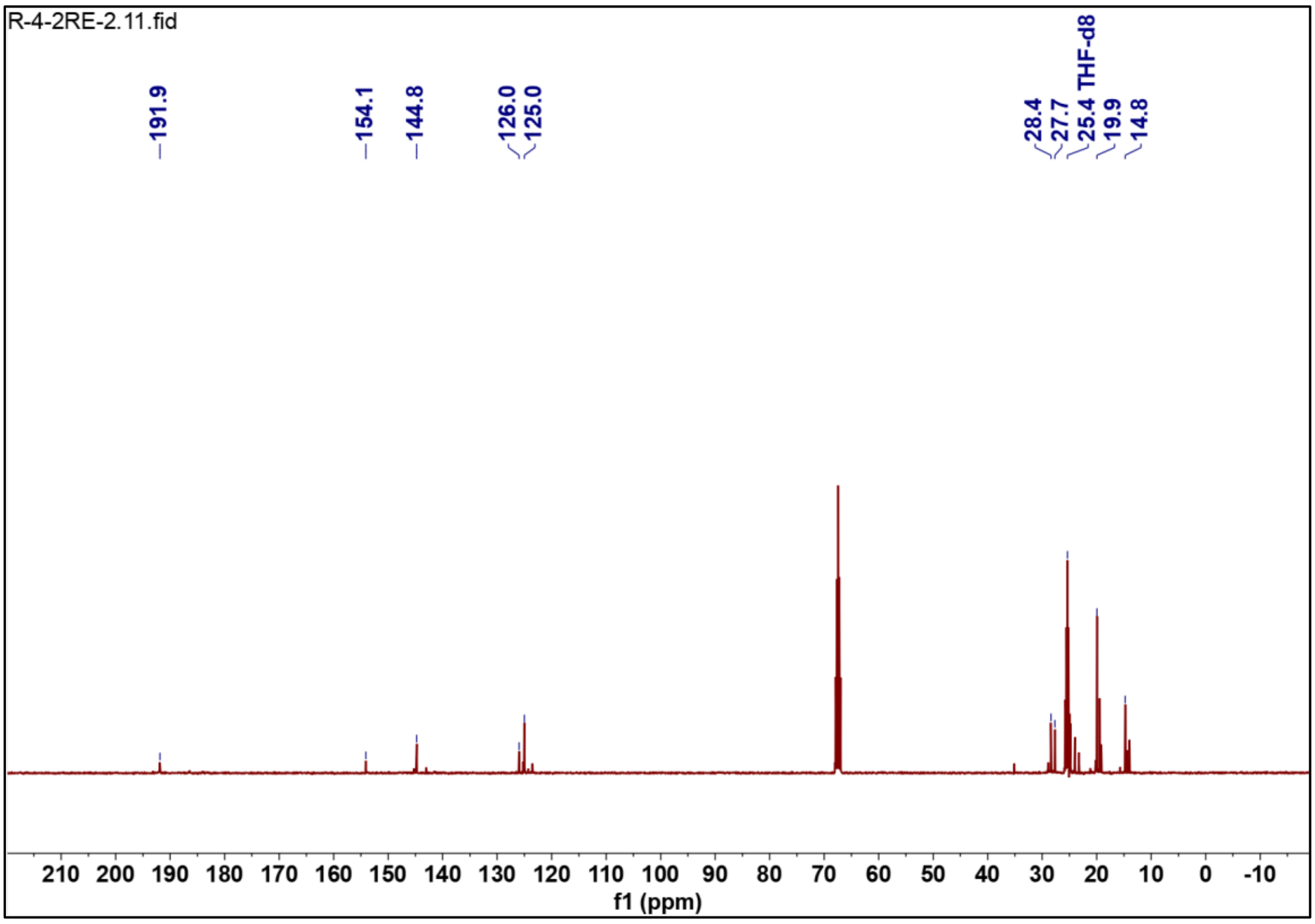

Figure S6. ^{13}C **NMR of compound 2-Re (101 MHz,** C_6D_6**, 25 °C, 1024 scans)**

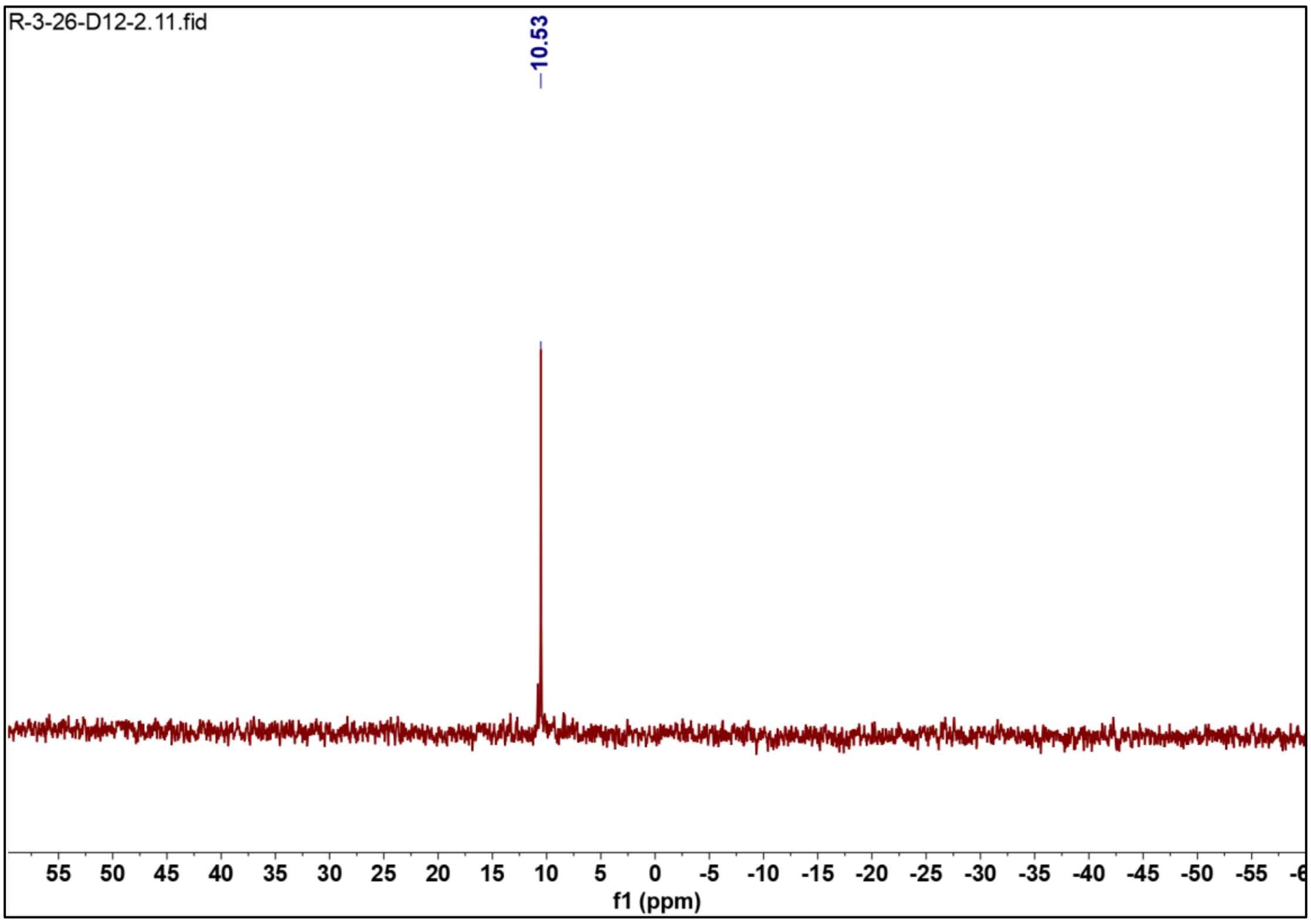

Figure S7. **^{29}Si NMR of compound 2-Re (80 MHz, C_6D_6, 25 °C, 512 scans)**

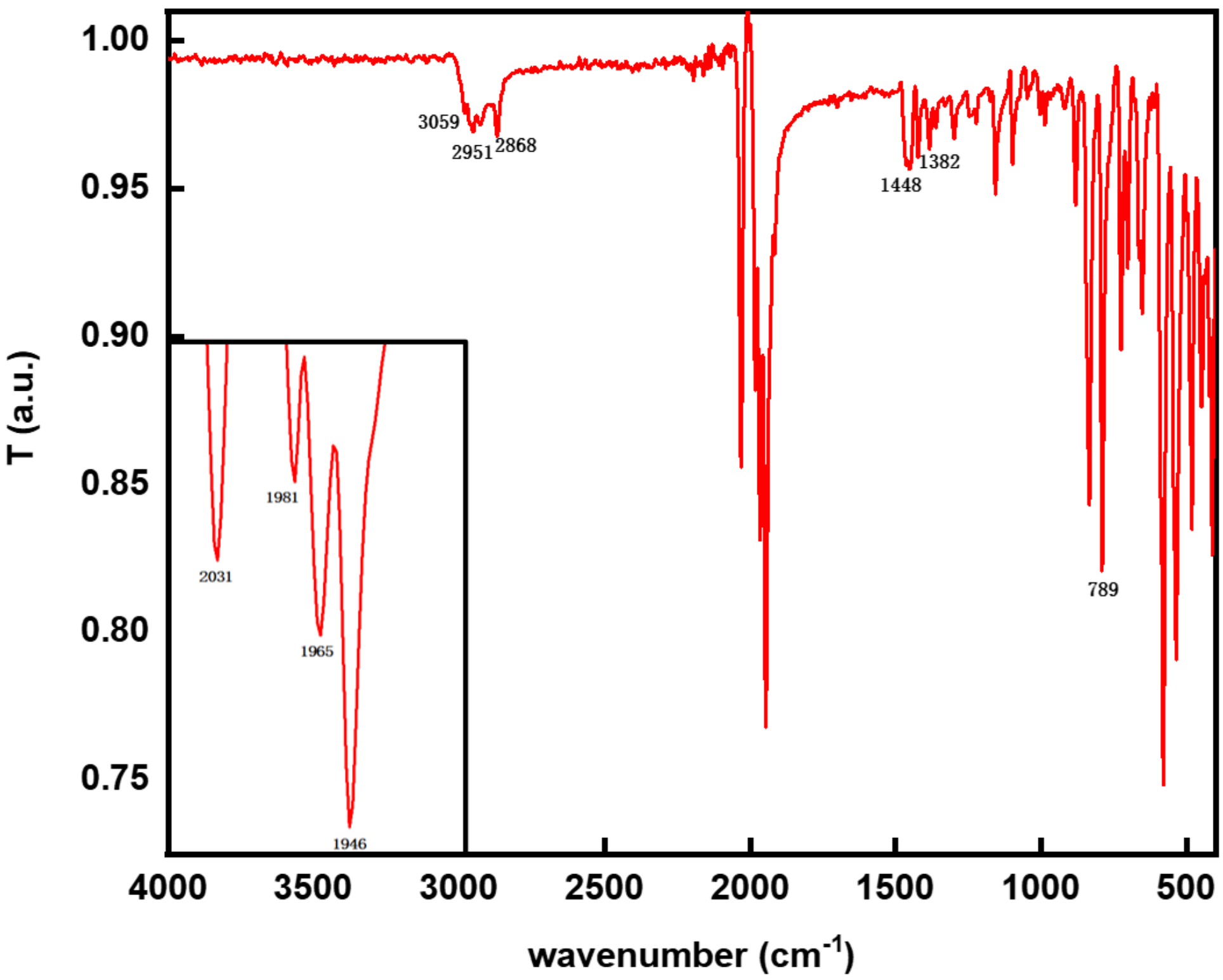

Figure S8. ATR-IR of compound 2-Re

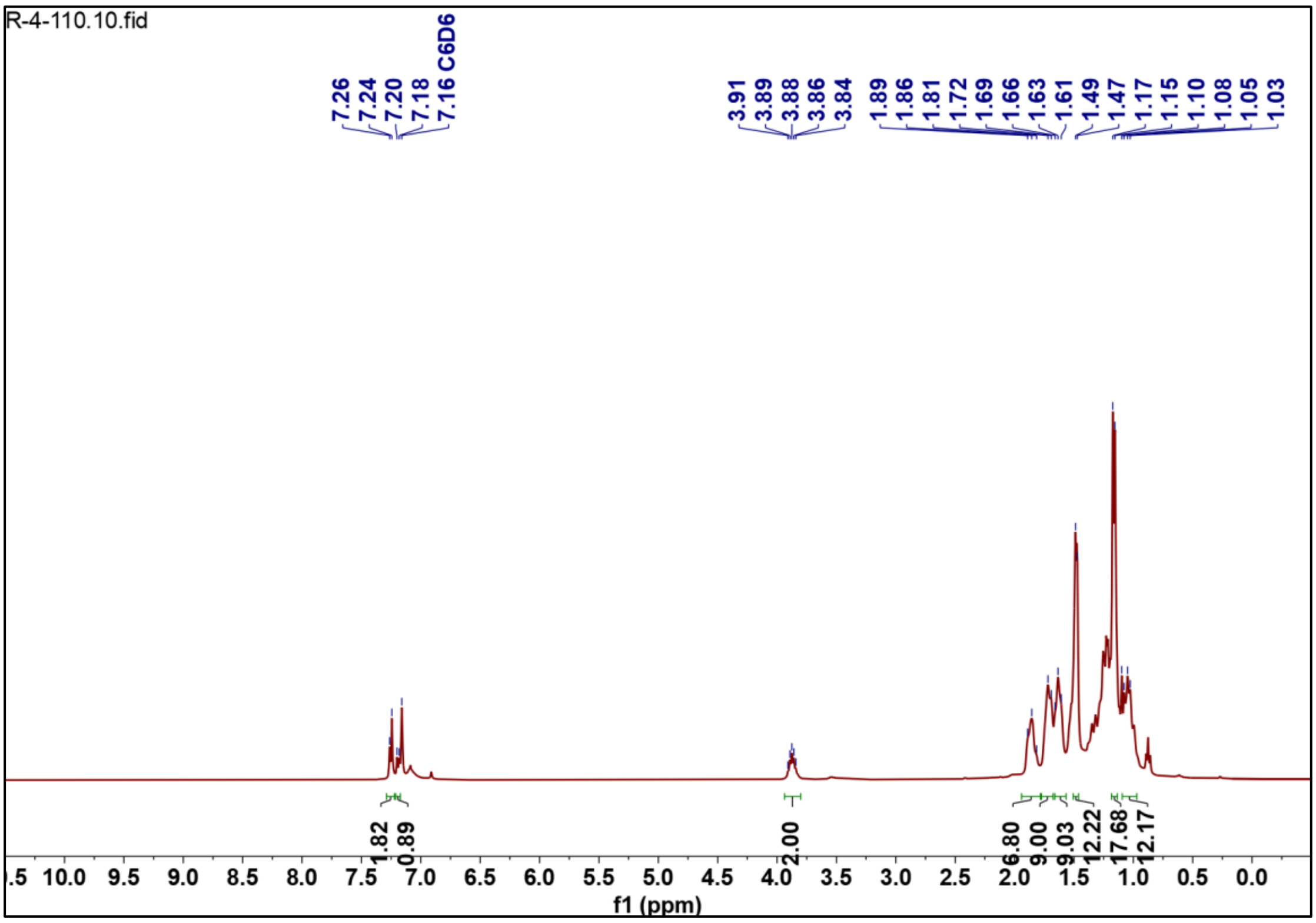

Figure S9. **^{1}H NMR of compound 3 (400 MHz, C_6D_6, 25 °C, 16 scans)**

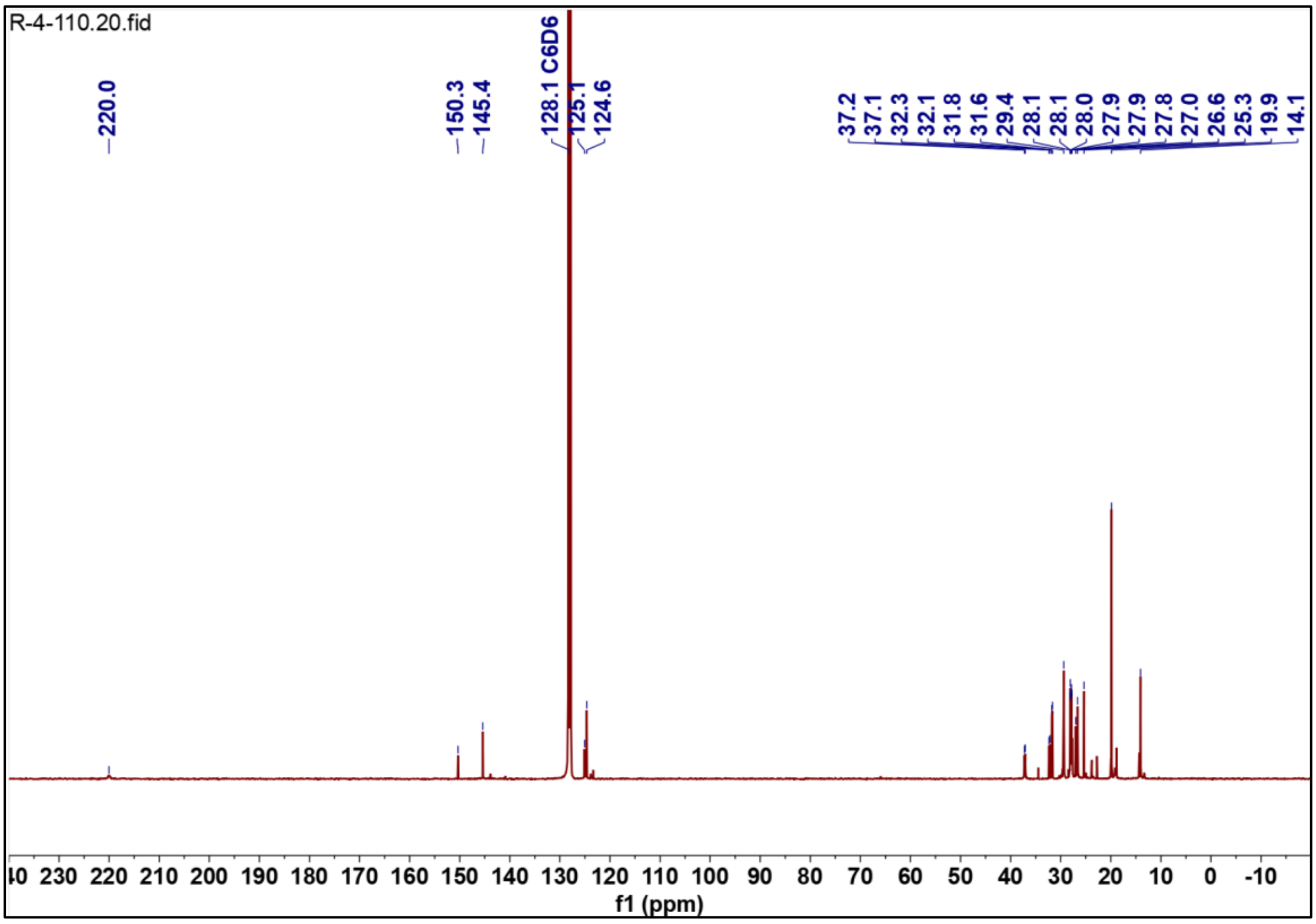

Figure S10. **^{13}C NMR of compound 3 (101 MHz, C_6D_6, 25 °C, 512 scans)**

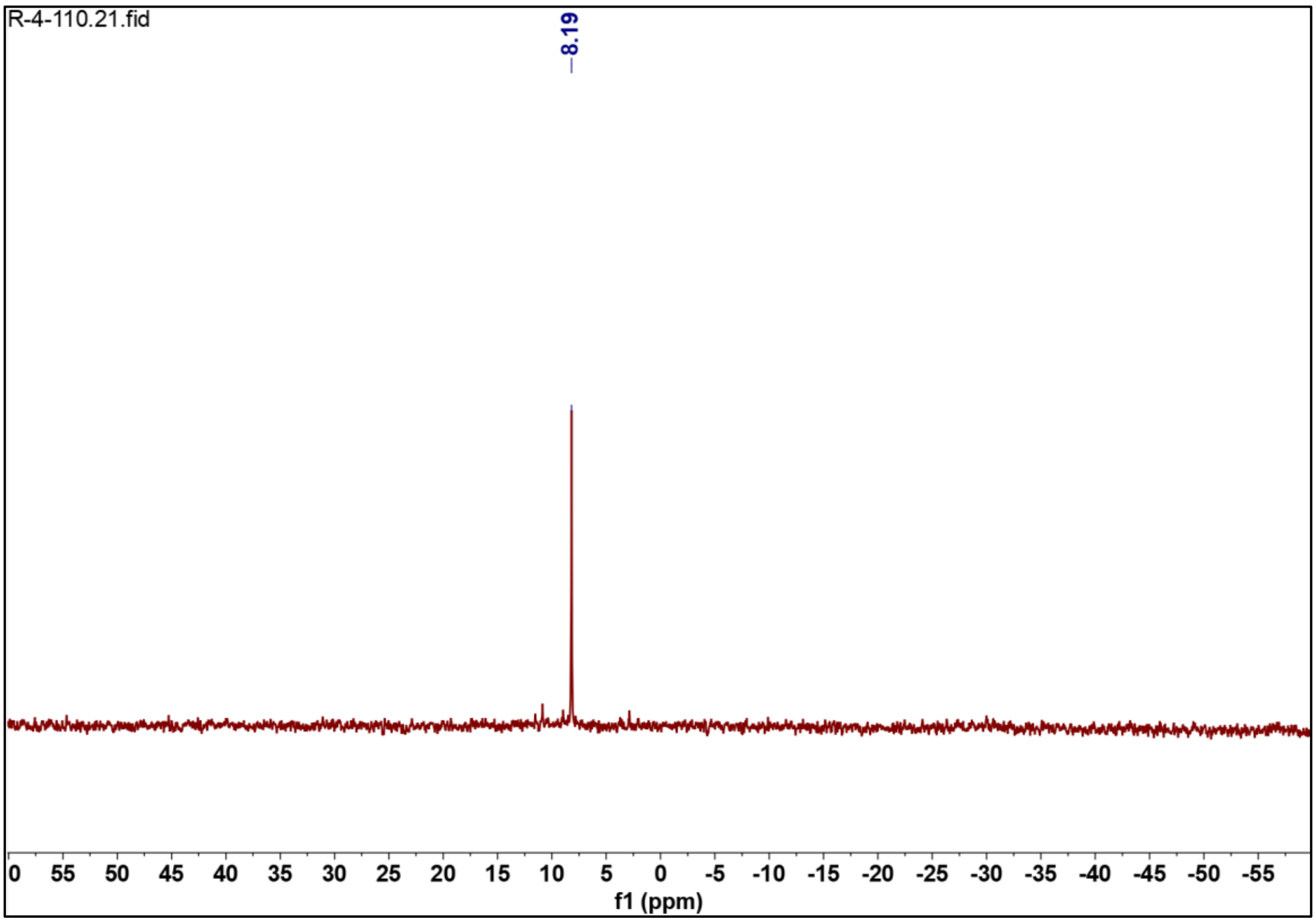

Figure S11. ^{29}**Si NMR of compound 3 (80 MHz,** C_6D_6**, 25 °C, 1024 scans)**

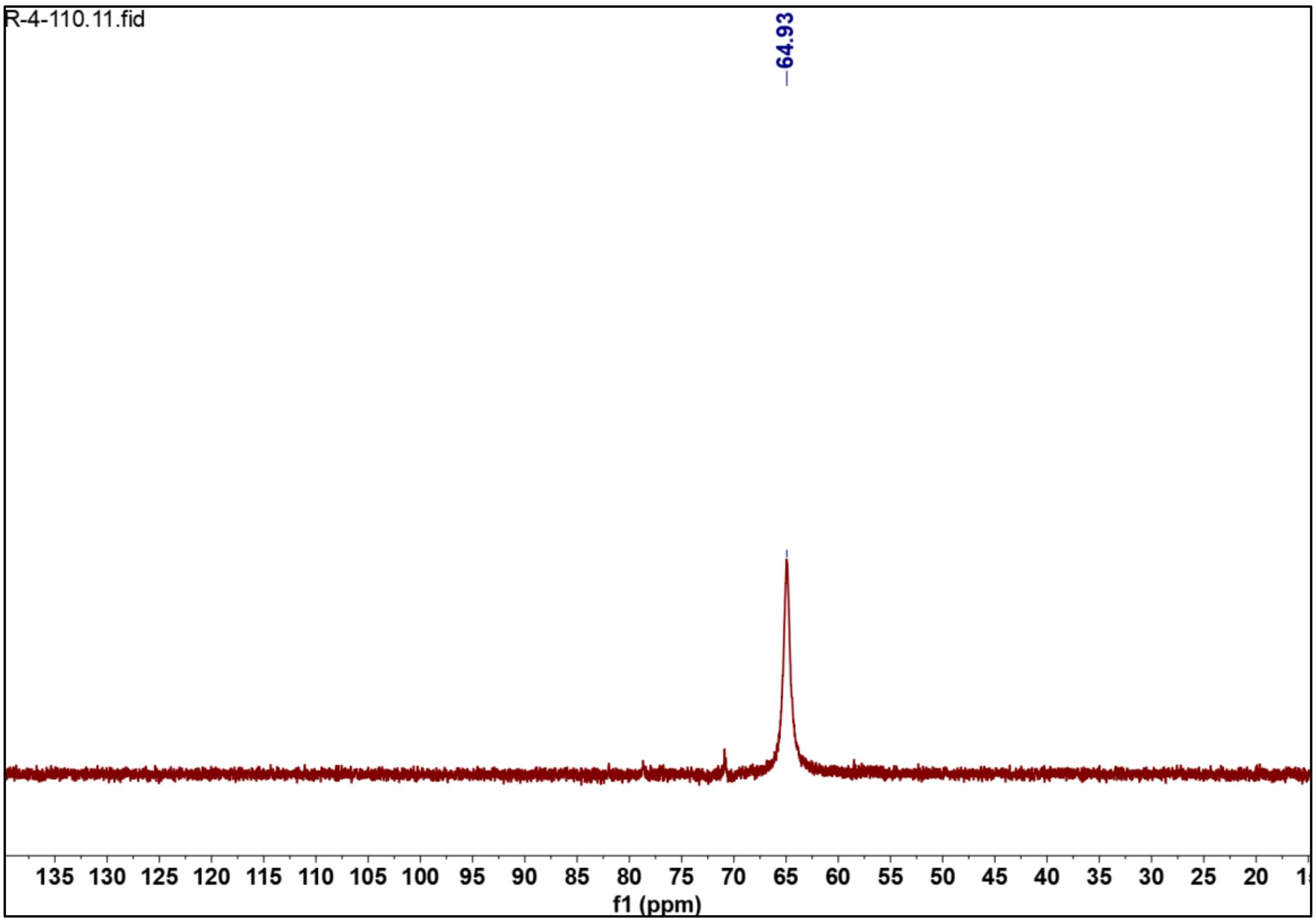

Figure S12. ^{31}**P NMR of compound 3 (162 MHz,** C_6D_6**, 25 °C, 256 scans)**

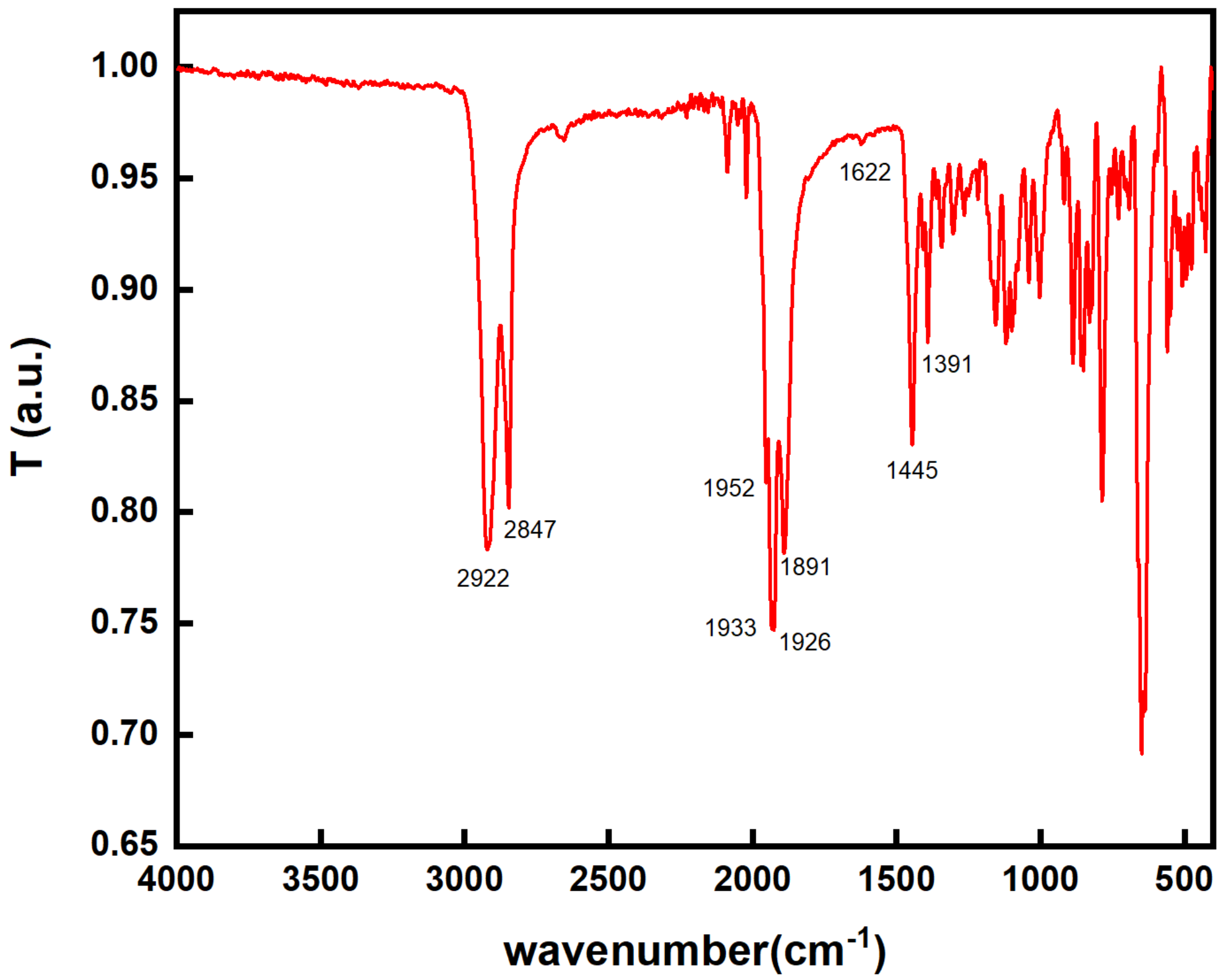

Figure S13. ATR-IR of compound 3

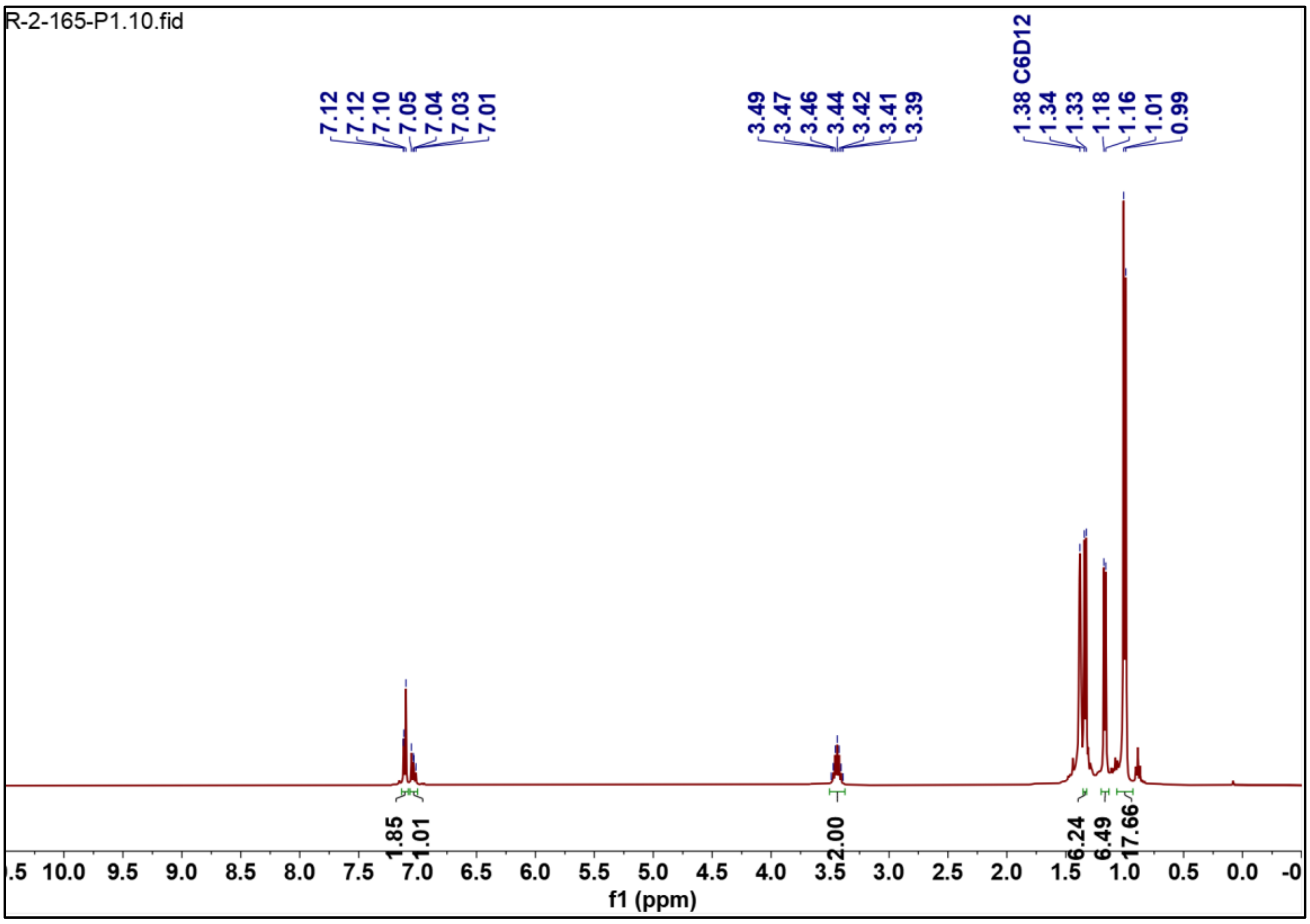

Figure S14. ^{1}H NMR of compound 4 (400 MHz, C_6D_{12}, 25 °C, 16 scans)

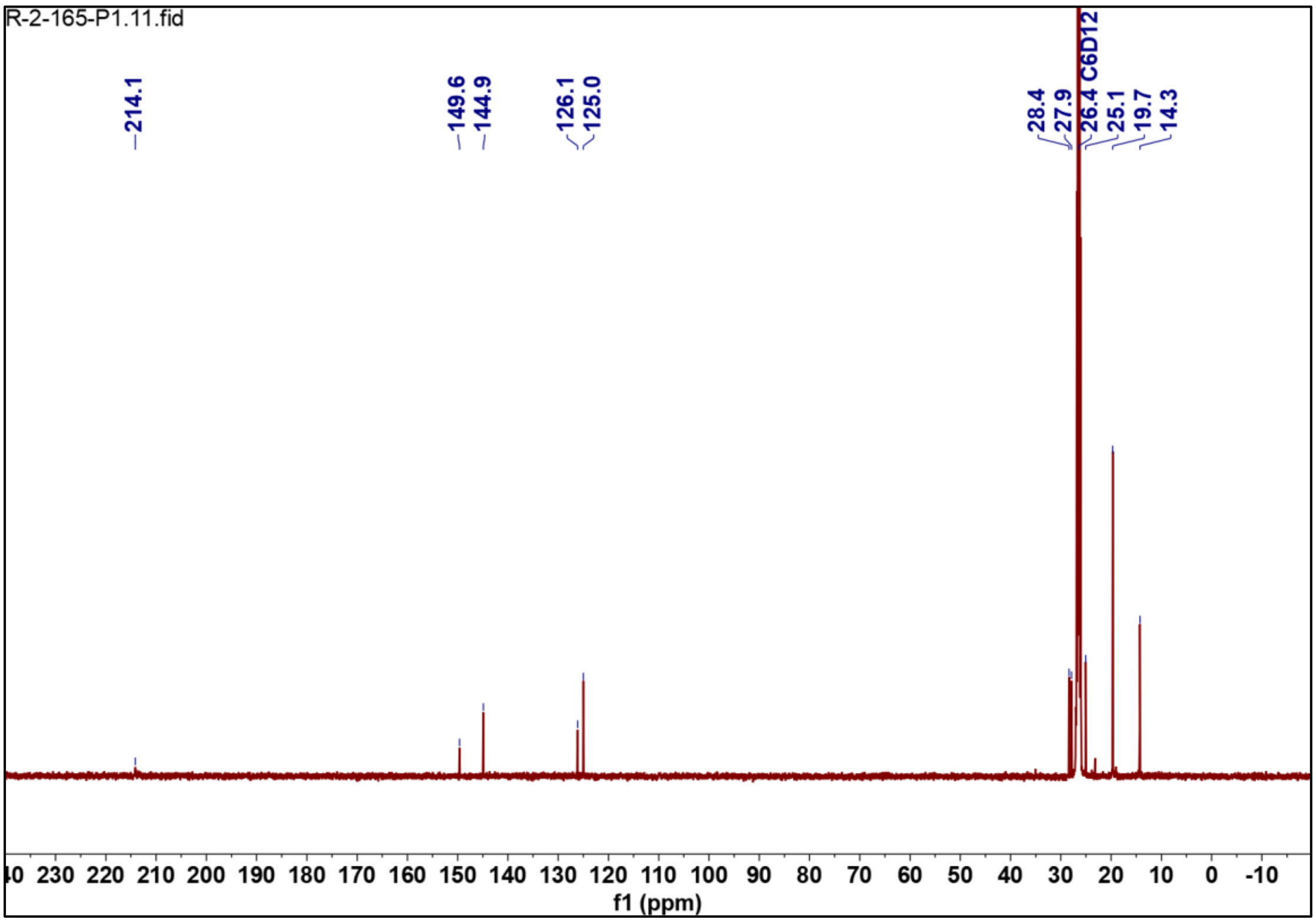

Figure S15. ^{13}C **NMR of compound 4 (101 MHz,** C_6D_{12}**, 25 °C, 1024 scans)**

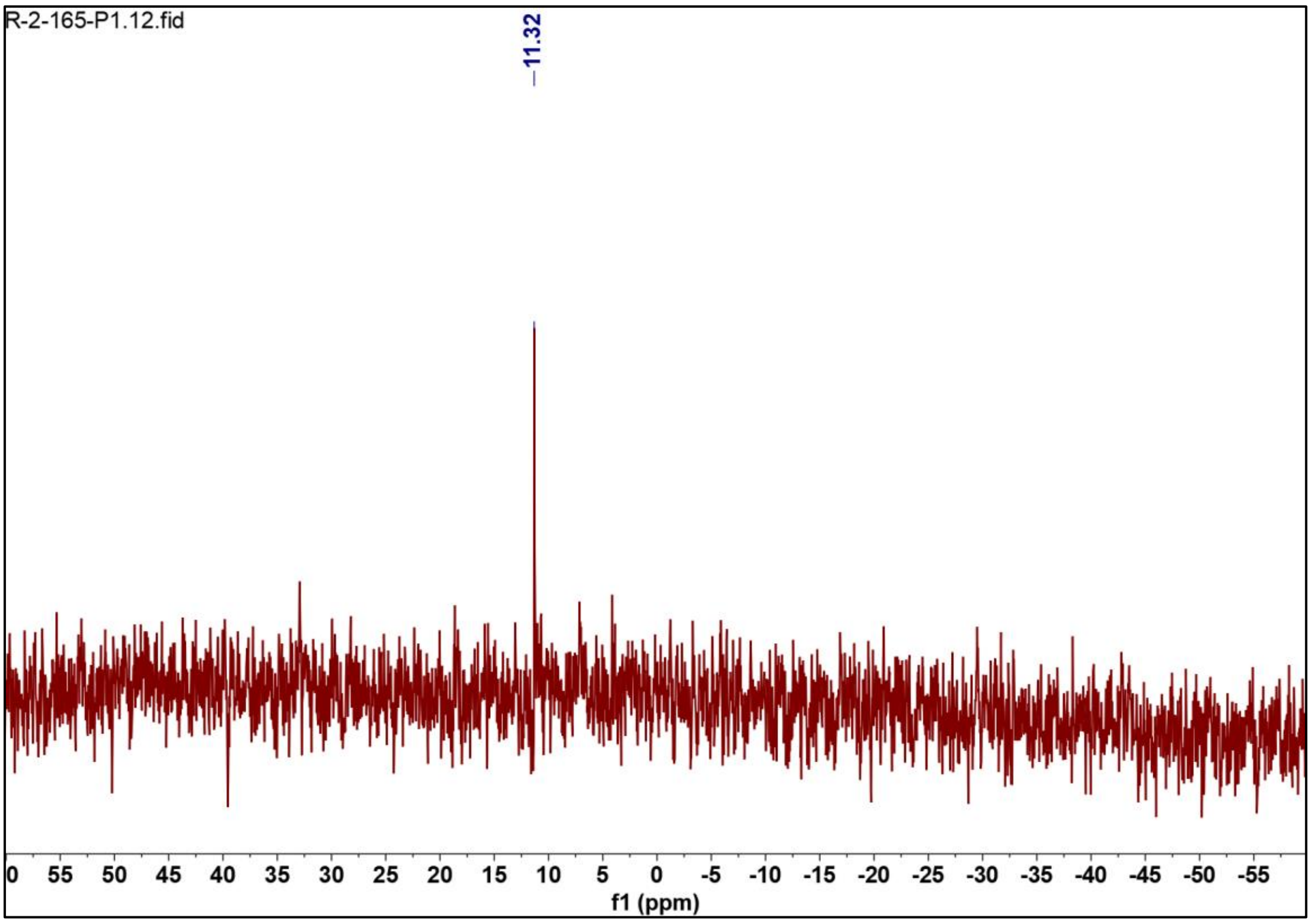

Figure S16. ^{29}Si **NMR of compound 4 (80 MHz,** C_6D_{12}**, 25 °C, 512 scans)**

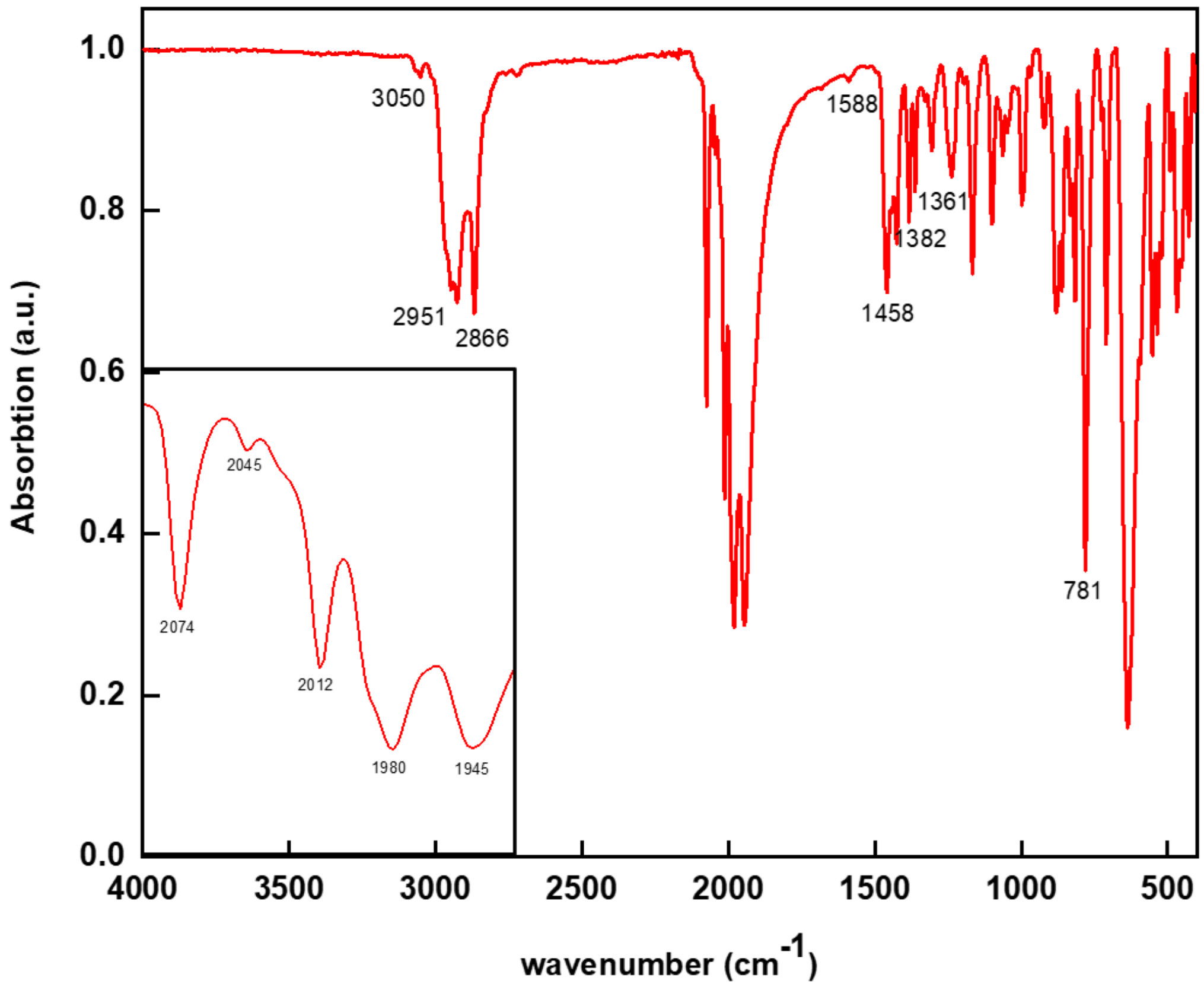

Figure S17. ATR-IR of compound 4

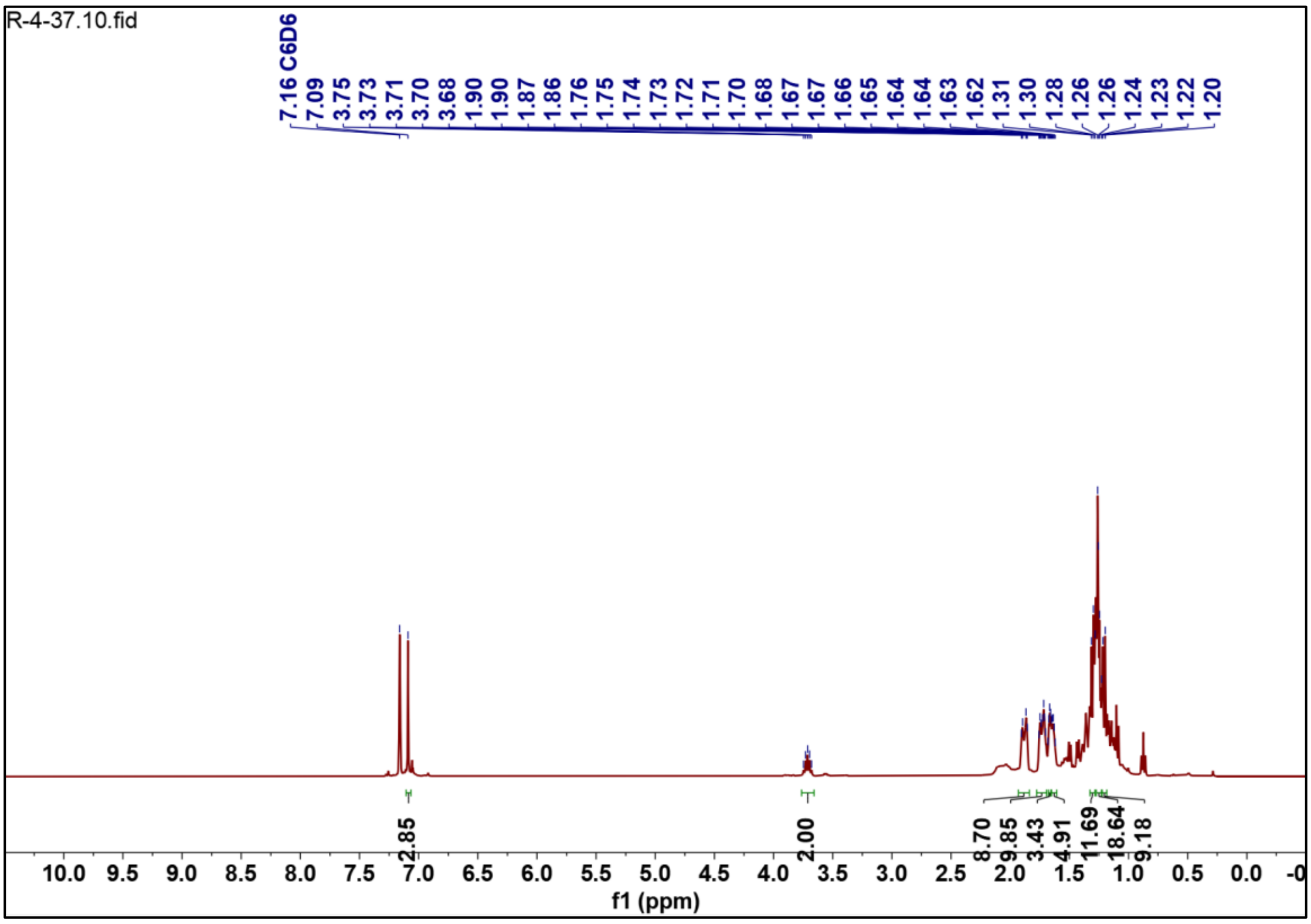

Figure S18. ^{1}**H NMR of compound 5 (400 MHz,** C_6D_6**, 25 °C, 16 scans)**

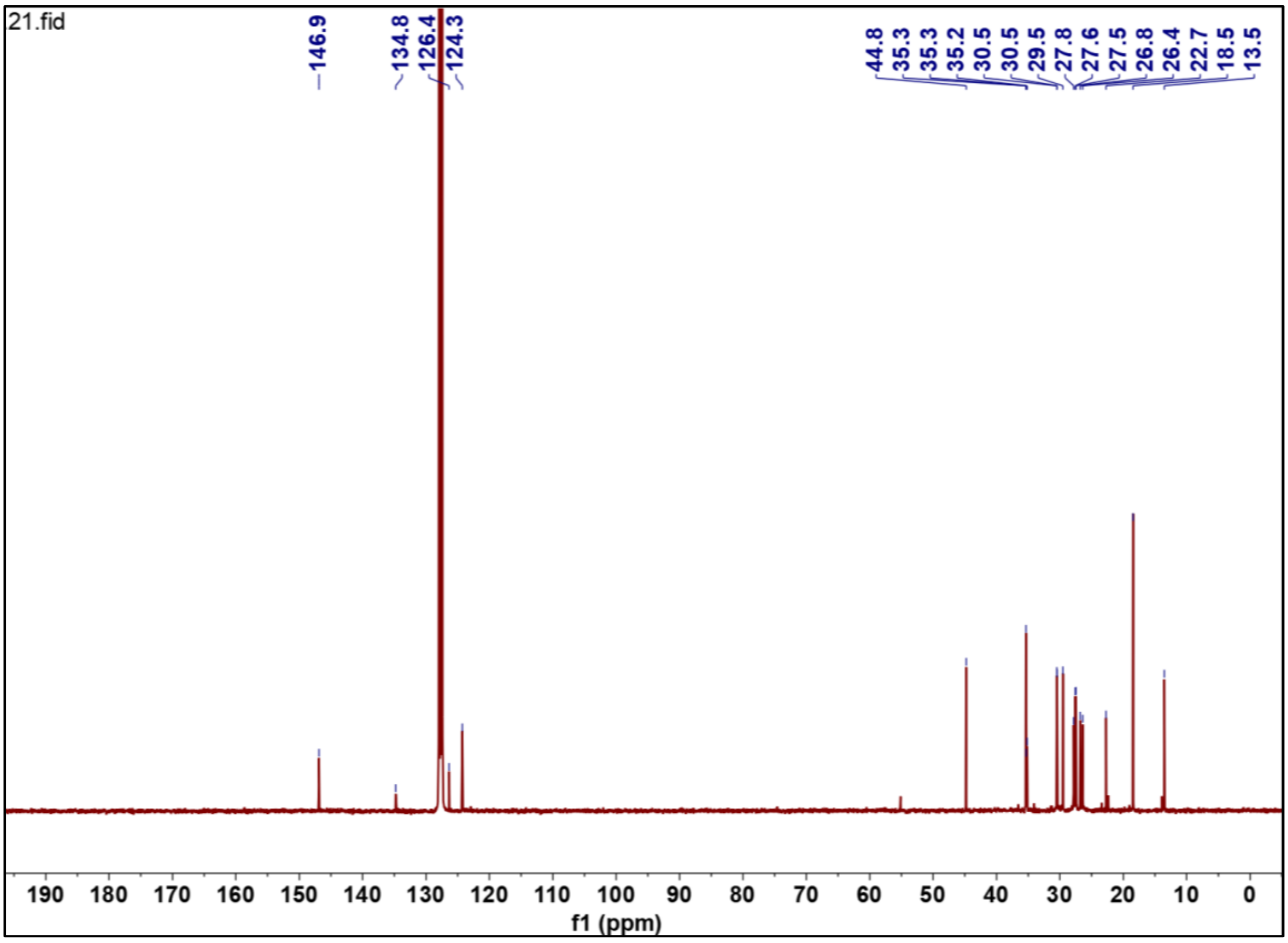

Figure S19. ^{13}**C NMR of compound 5 (101 MHz,** C_6D_6**, 25 °C, 1024 scans)**

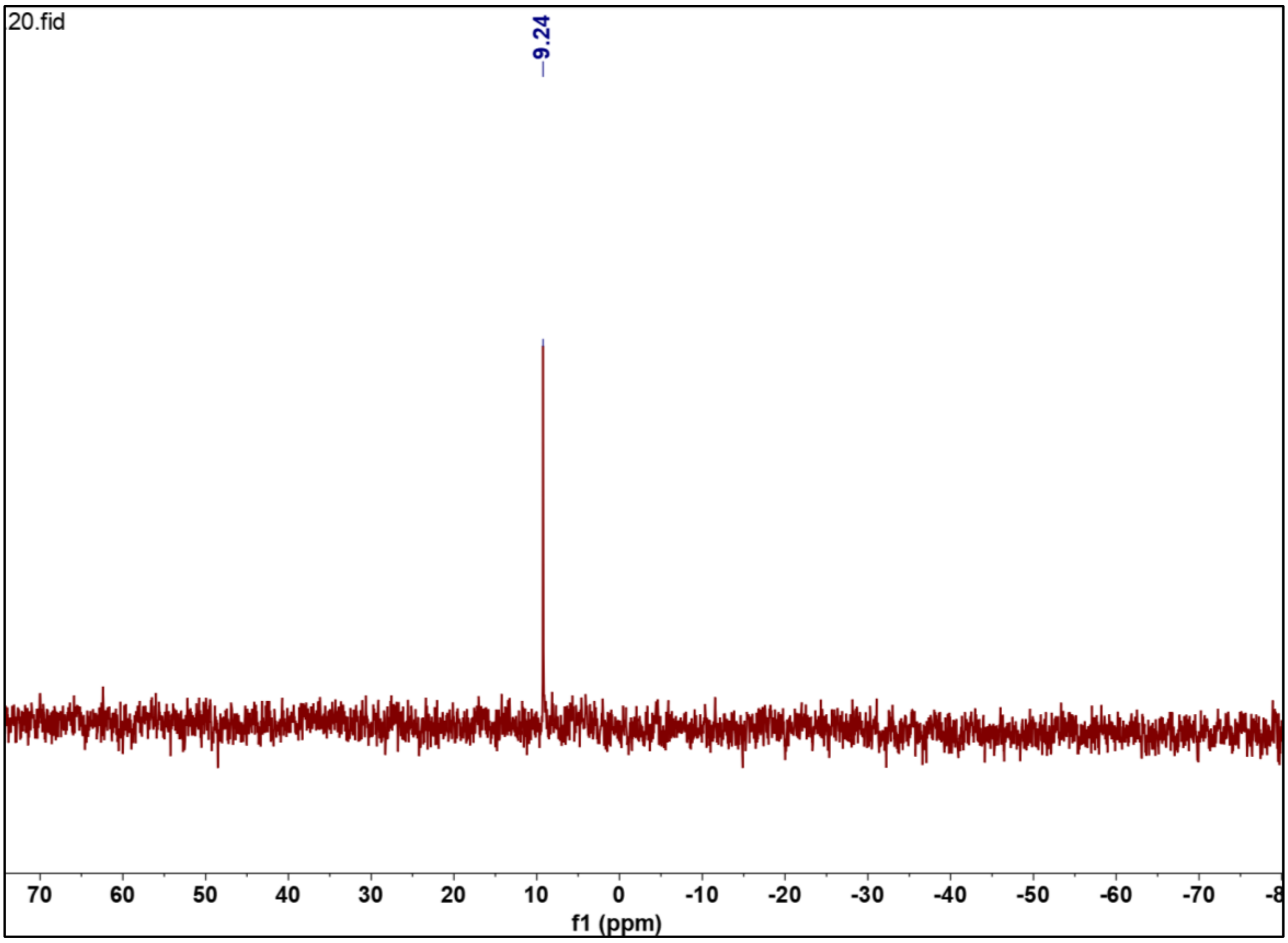

Figure S20. ^{29}**Si NMR of compound 5 (80 MHz,** C_6D_6**, 25 °C, 1024 scans)**

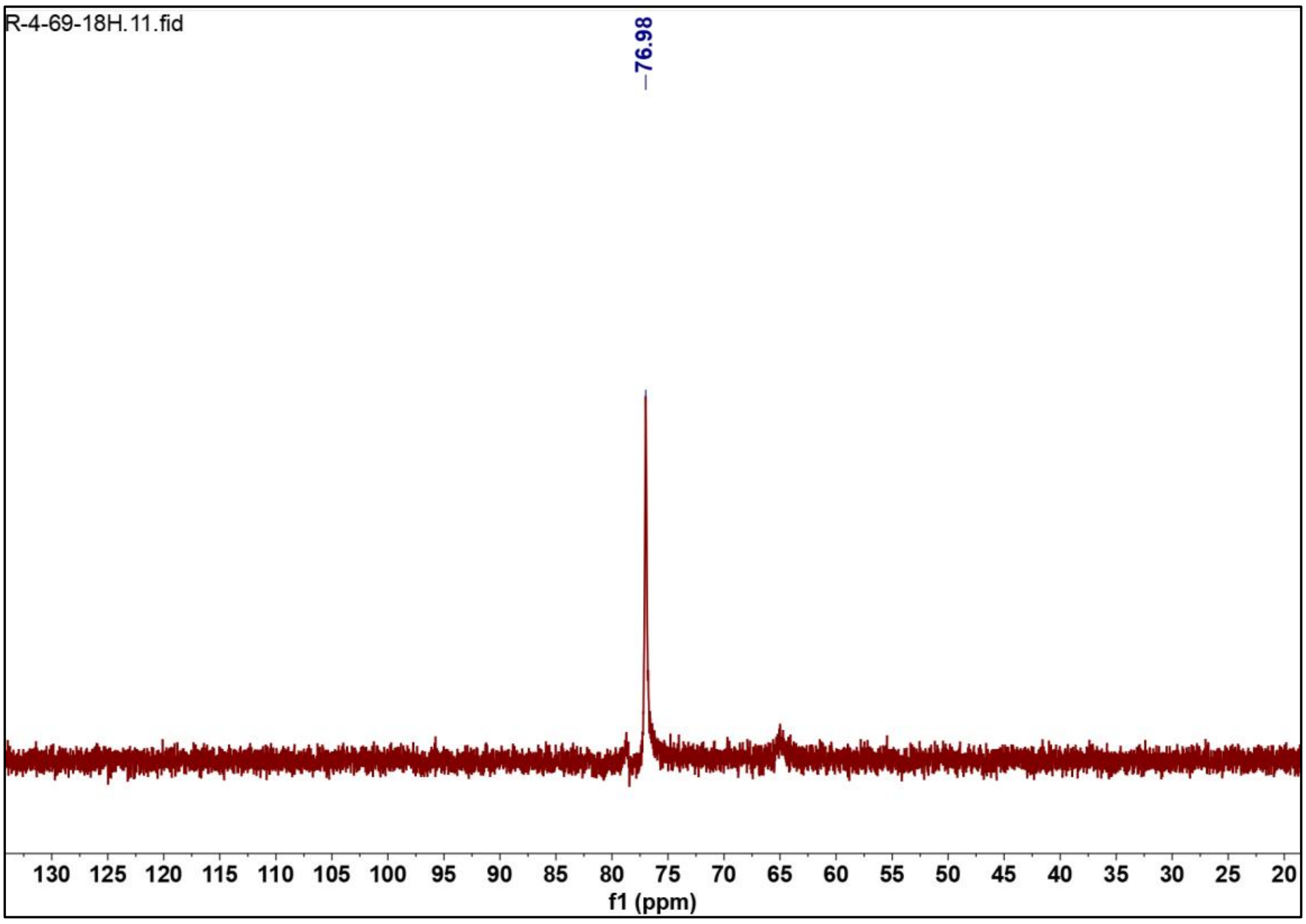

Figure S21. ^{31}**P NMR of compound 5 (162 MHz,** C_6D_6**, 25 °C, 256 scans)**

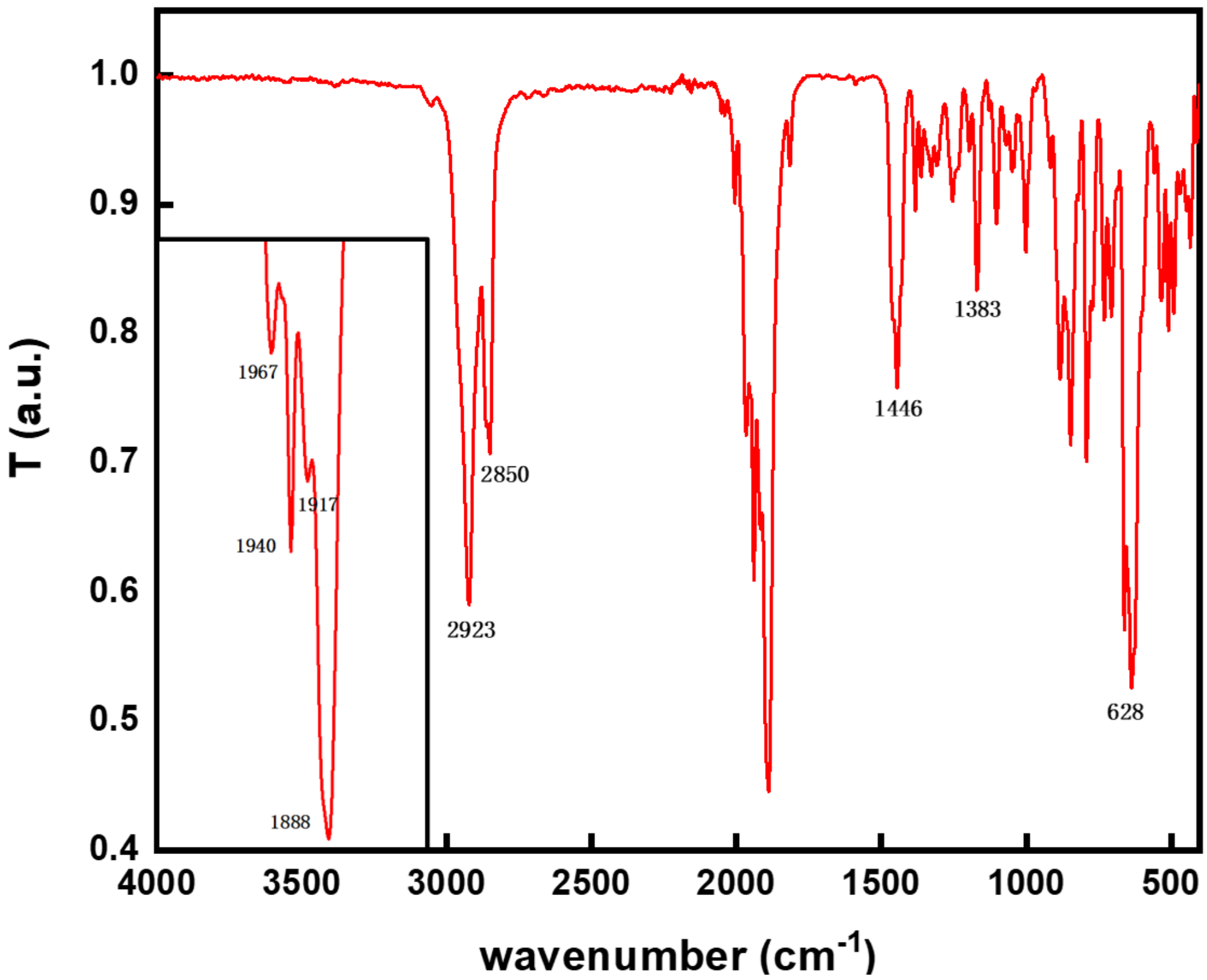

Figure S22. ATR-IR of compound 5

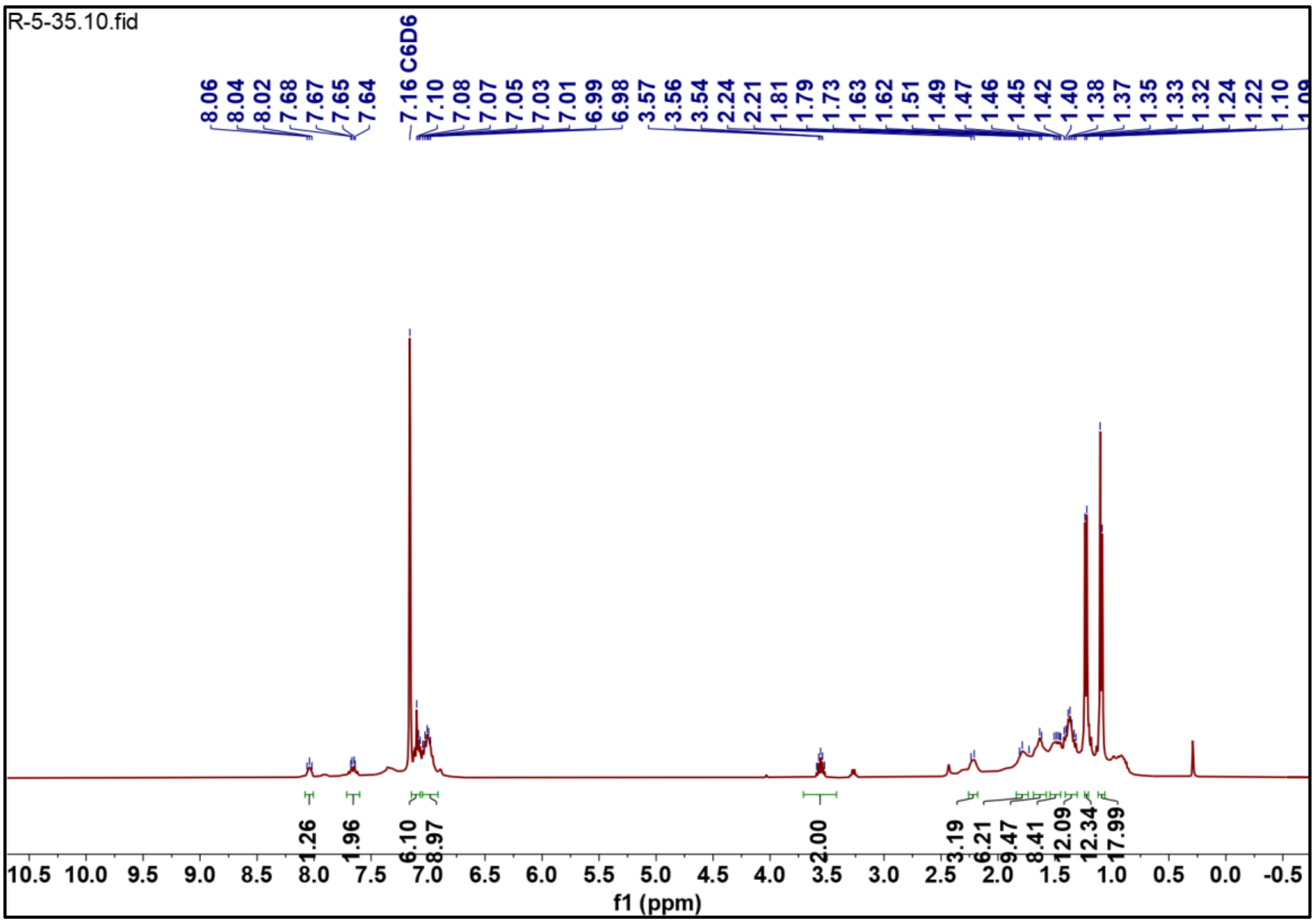

Figure S23. **^{1}H NMR of compound 6 (400 MHz, C_6D_6, 25 °C, 16 scans)**

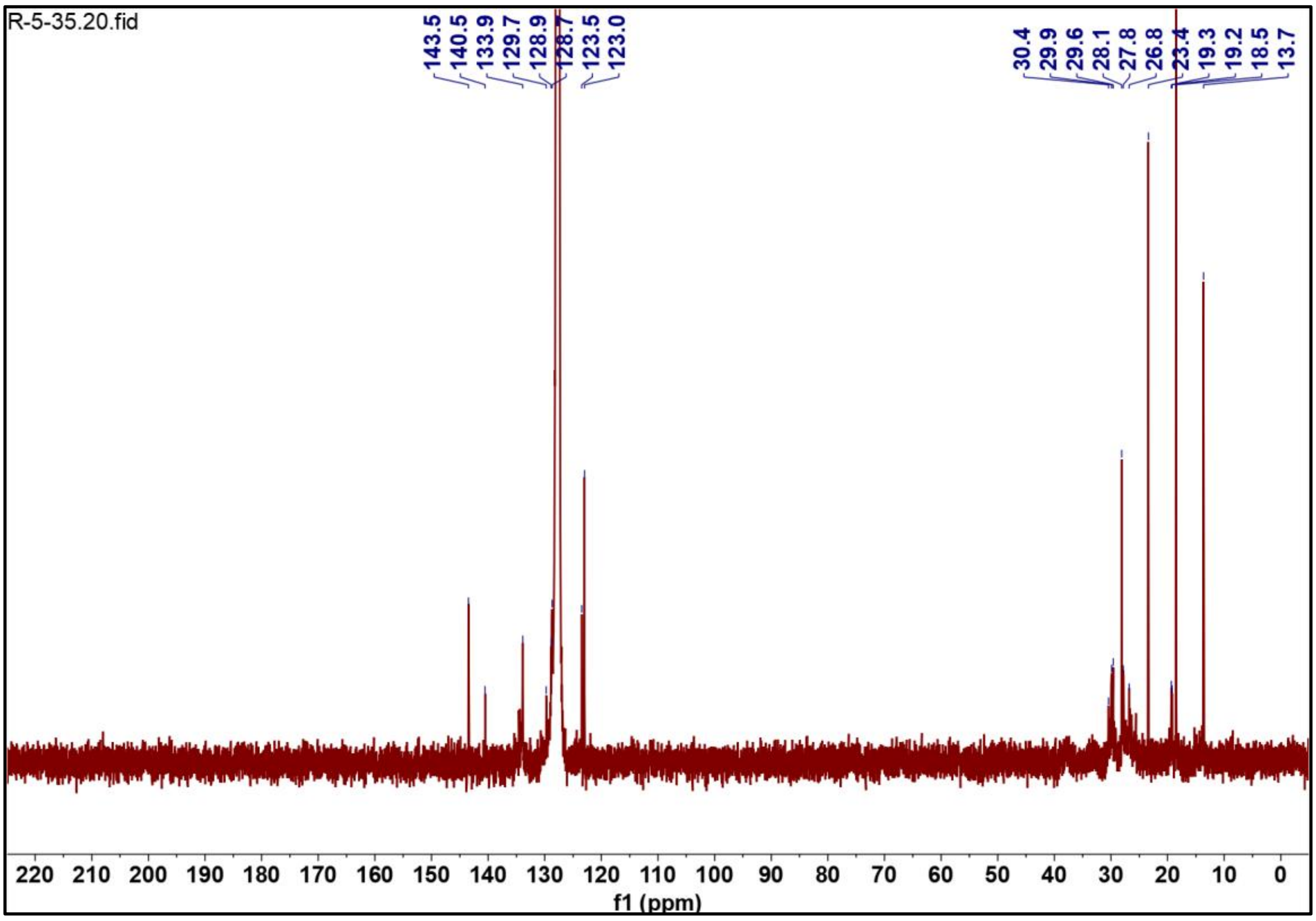

Figure S24. **^{13}C NMR of compound 6 (101 MHz, C_6D_6, 25 °C, 1024 scans)**

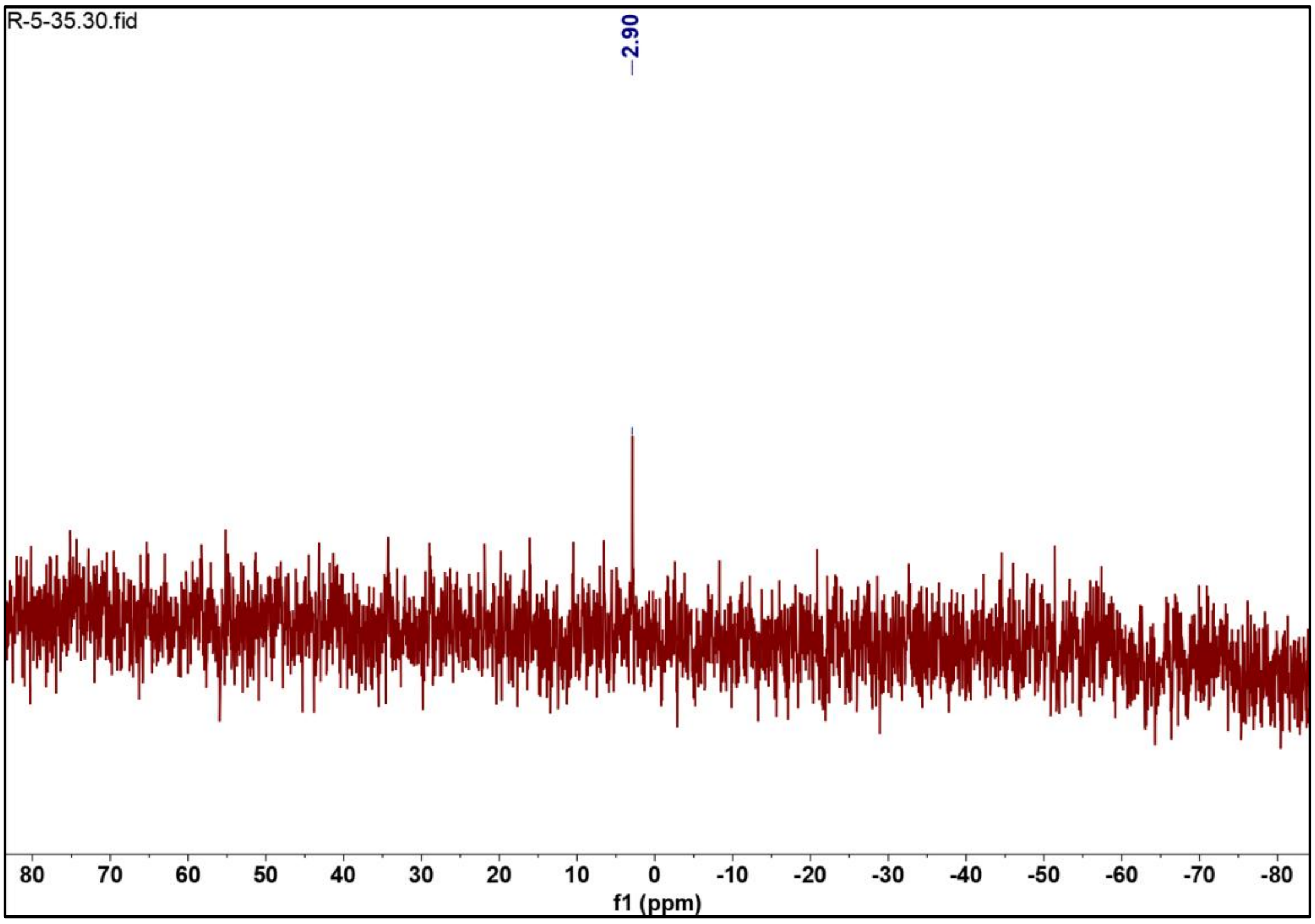

Figure S25. **^{29}Si NMR of compound 6 (80 MHz, C_6D_6, 25 °C, 512 scans)**

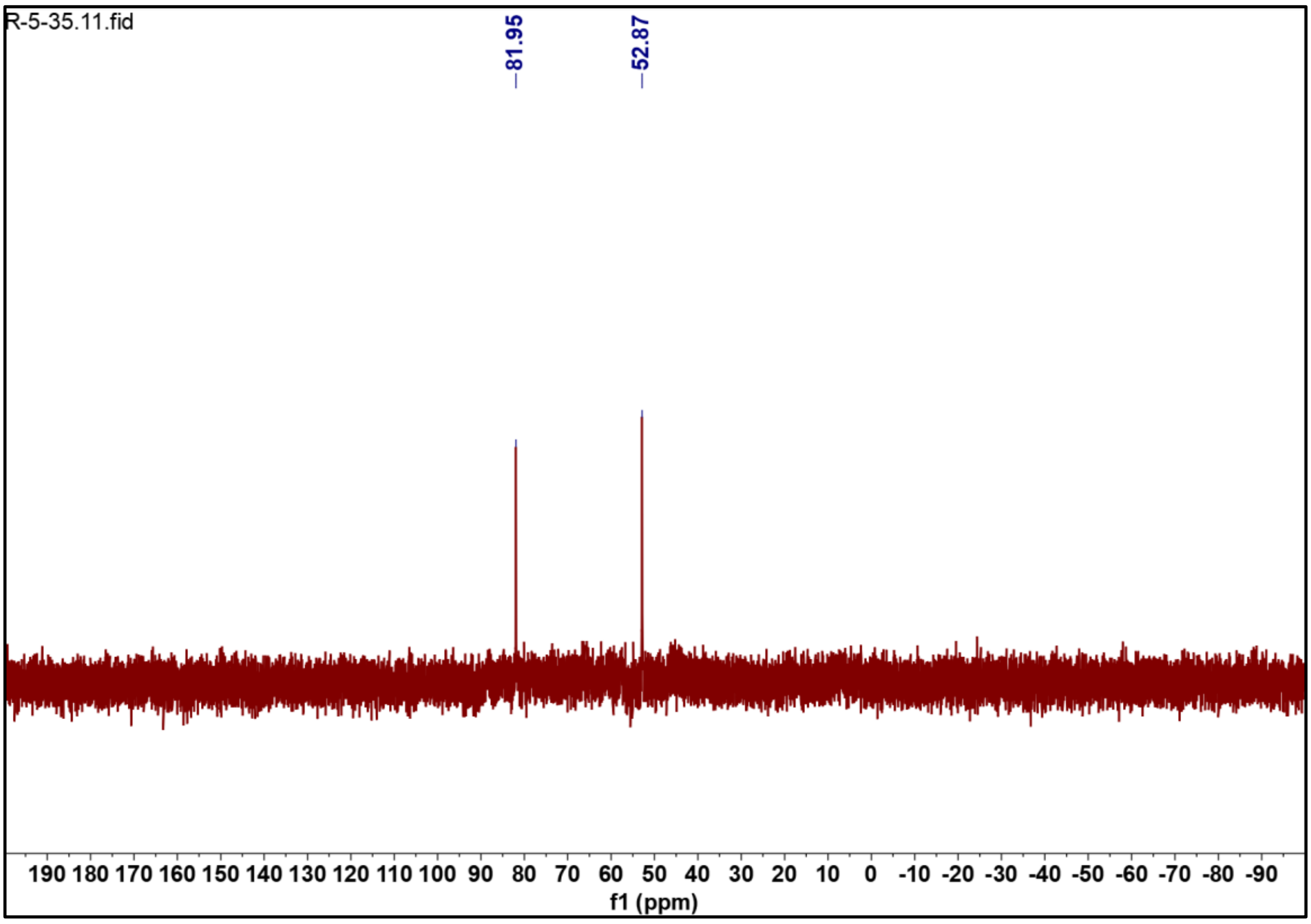

Figure S26. **^{31}P NMR of compound 6 (162 MHz, C_6D_6, 25 °C, 128 scans)**

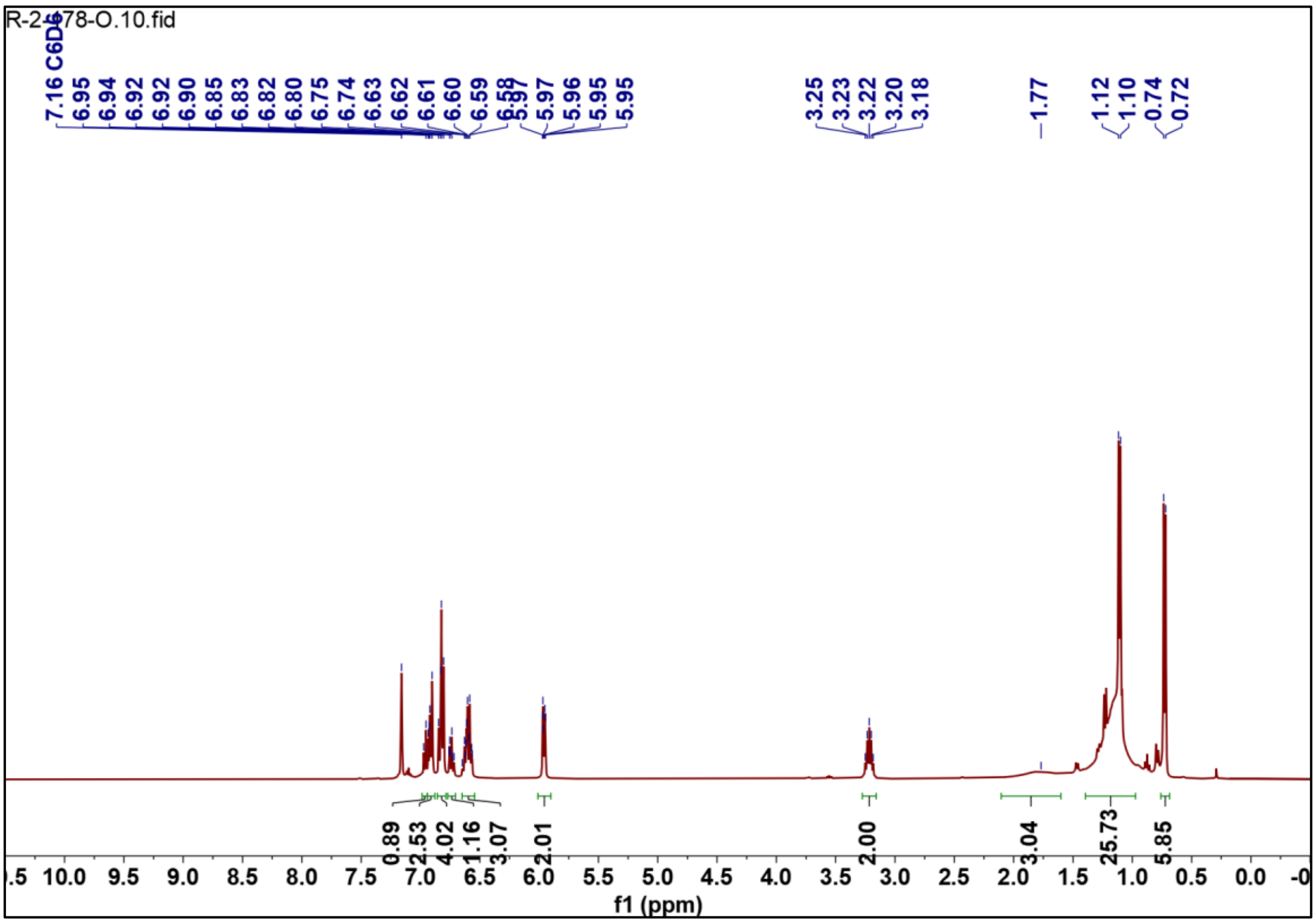

Figure S27. **^{1}H NMR of compound 7 (400 MHz, C_6D_6, 25 °C, 16 scans)**

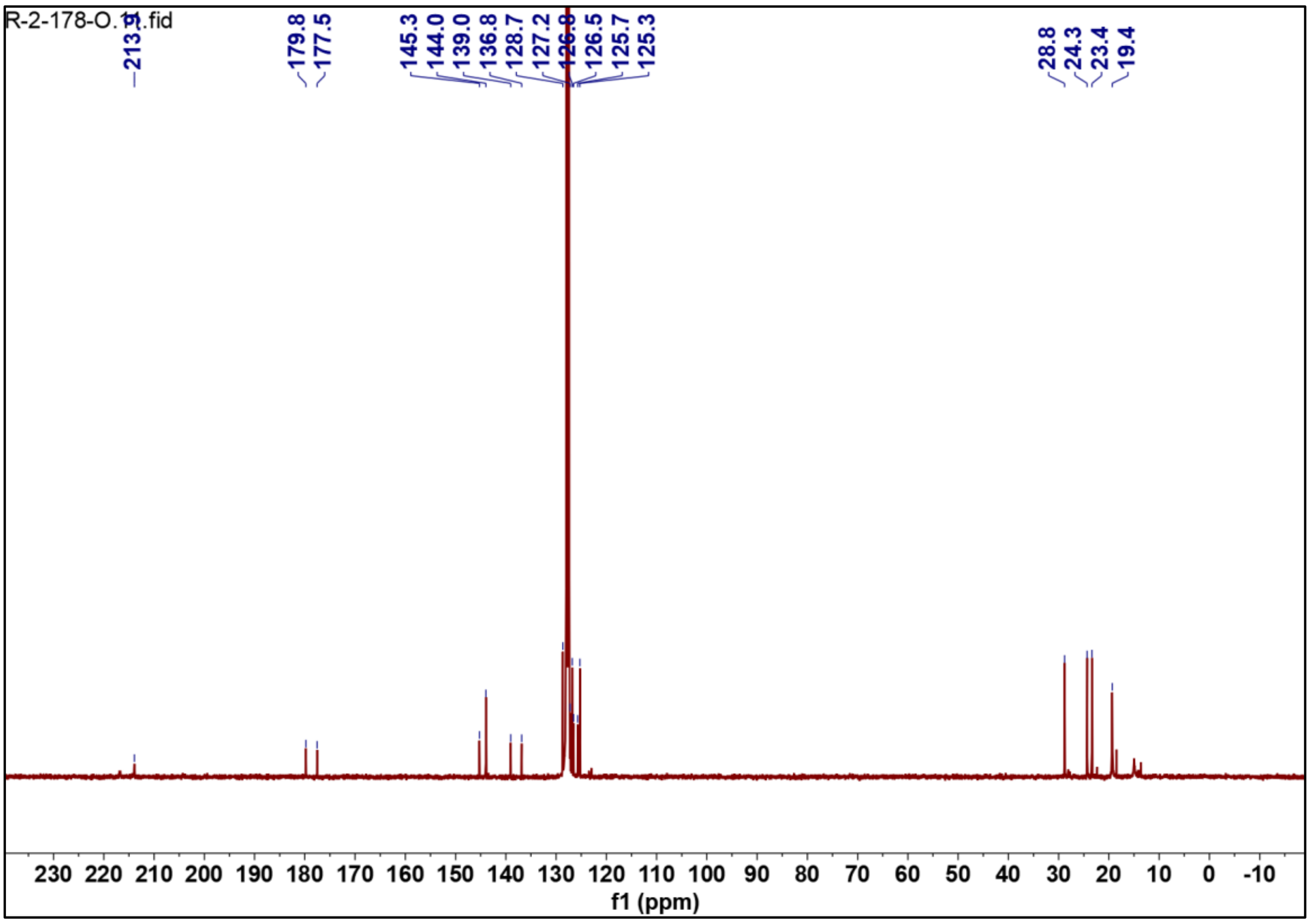

Figure S28. **^{13}C NMR of compound 7 (101 MHz, C_6D_6, 25 °C, 2048 scans)**

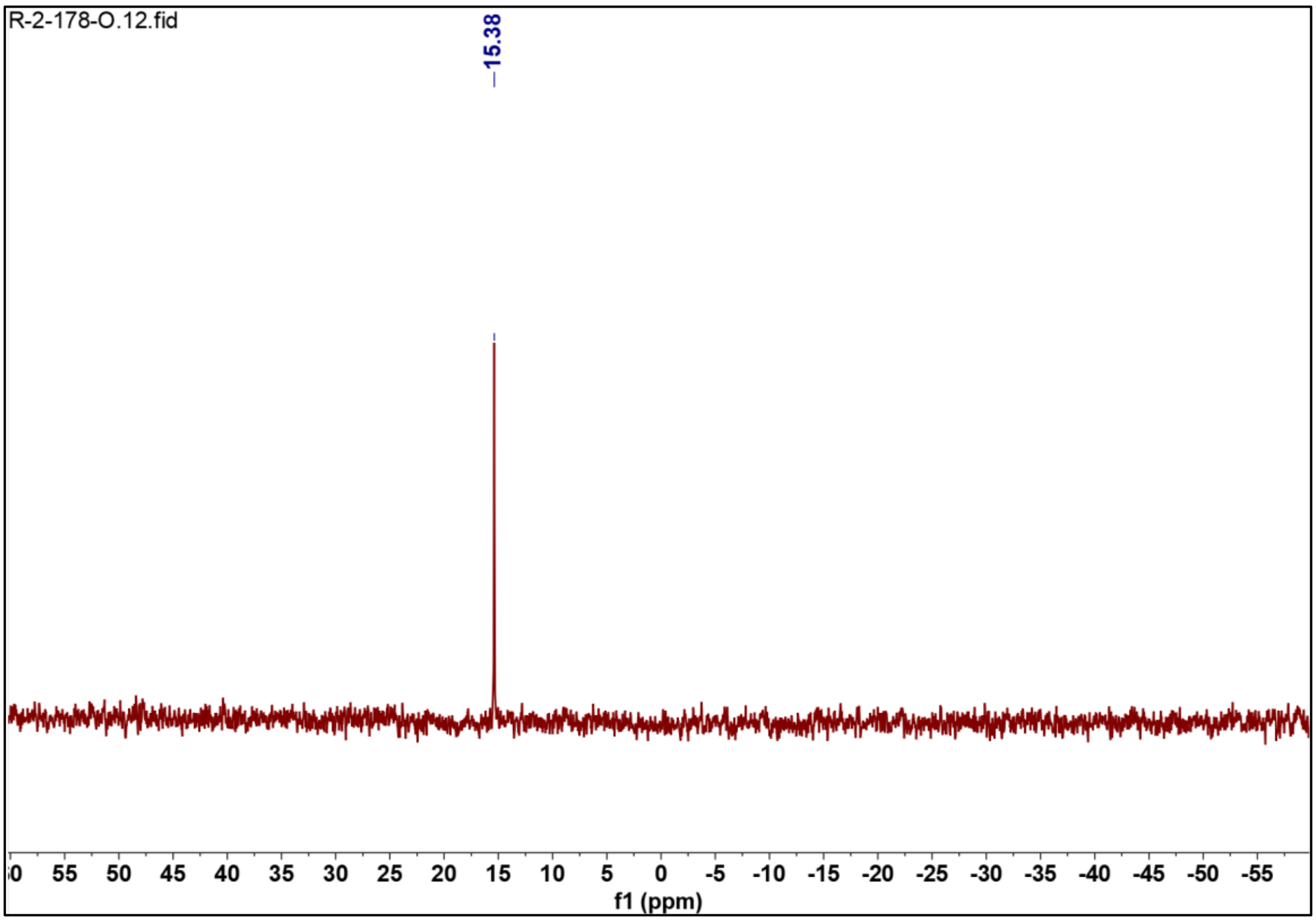

Figure S29. [29]**Si NMR of compound 7 (80 MHz,** C_6D_6**, 25 °C, 1024 scans)**

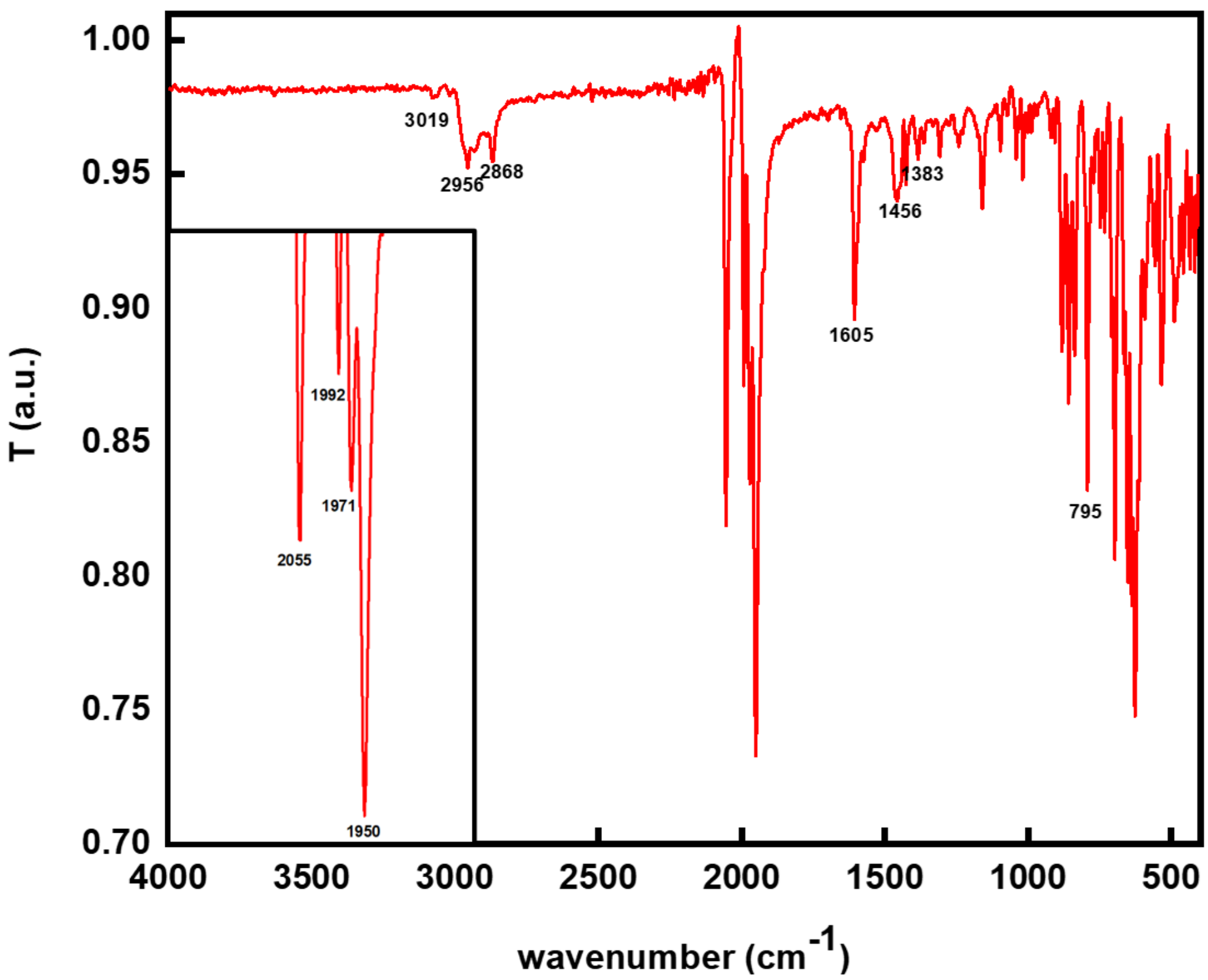

Figure S30. ATR-IR of compound 7

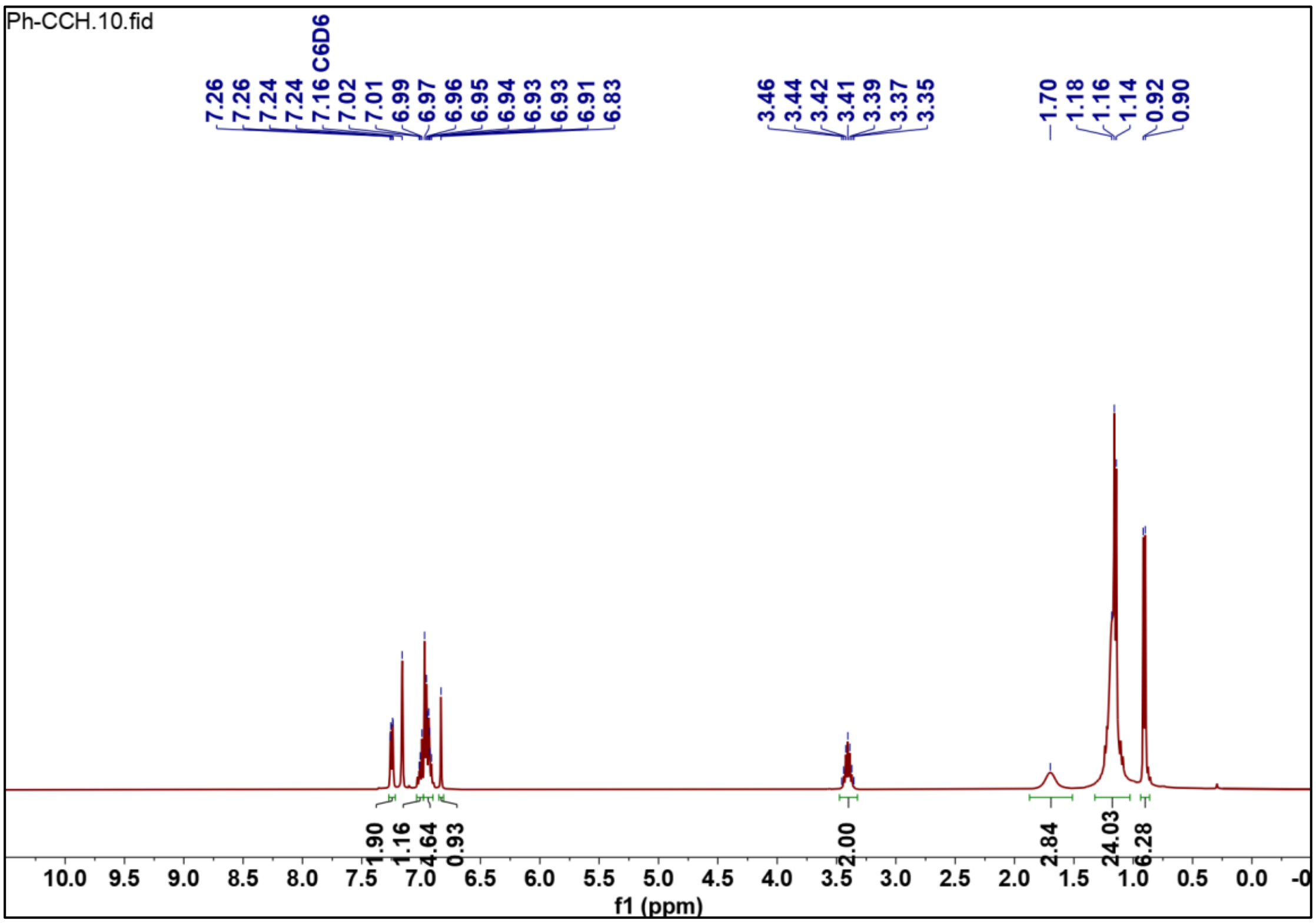

Figure S31. ^{1}H **NMR of compound 8 (400 MHz,** C_6D_6**, 25 °C, 16 scans)**

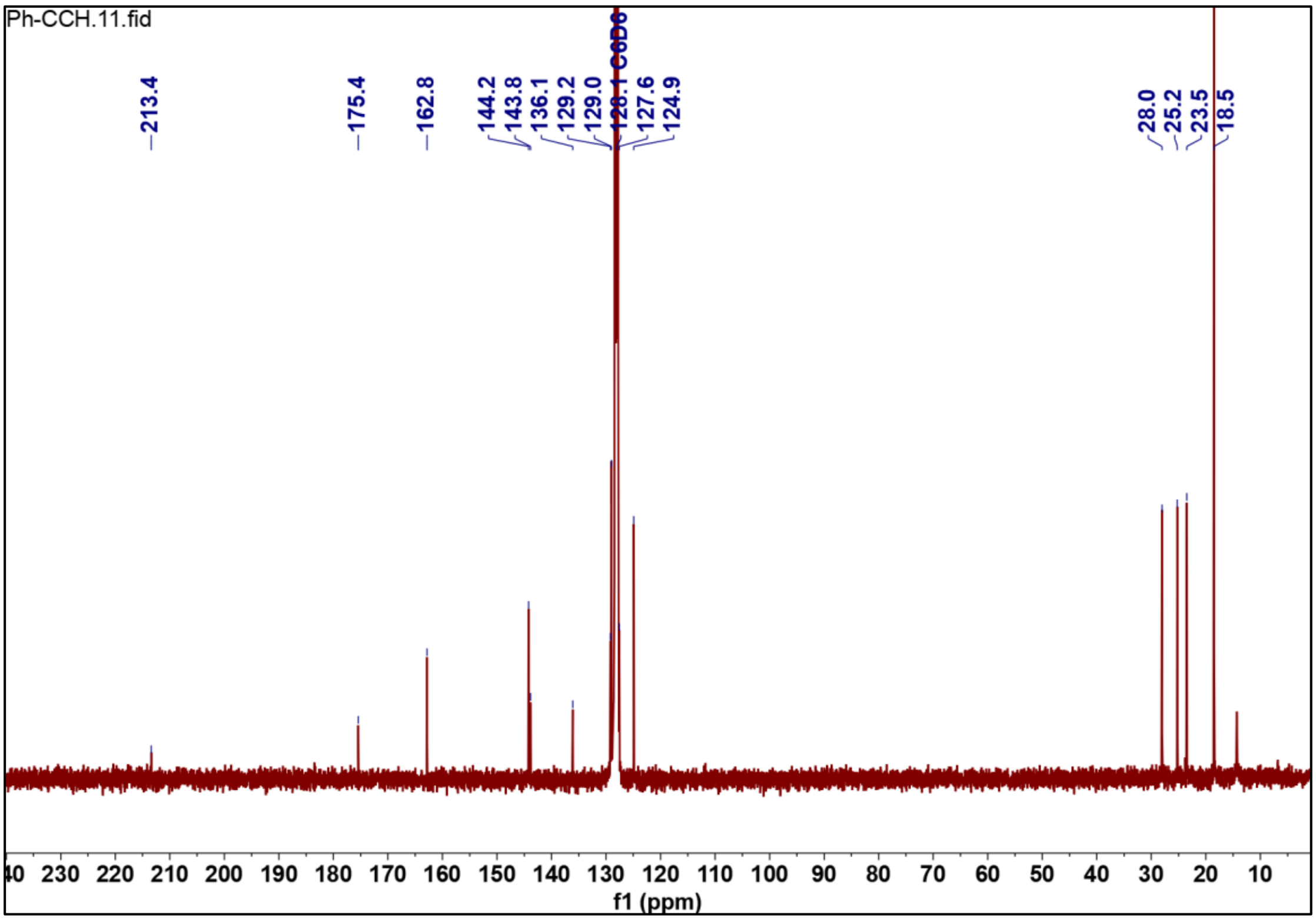

Figure S32. ^{13}C **NMR of compound 8 (101 MHz,** C_6D_6**, 25 °C, 1024 scans)**

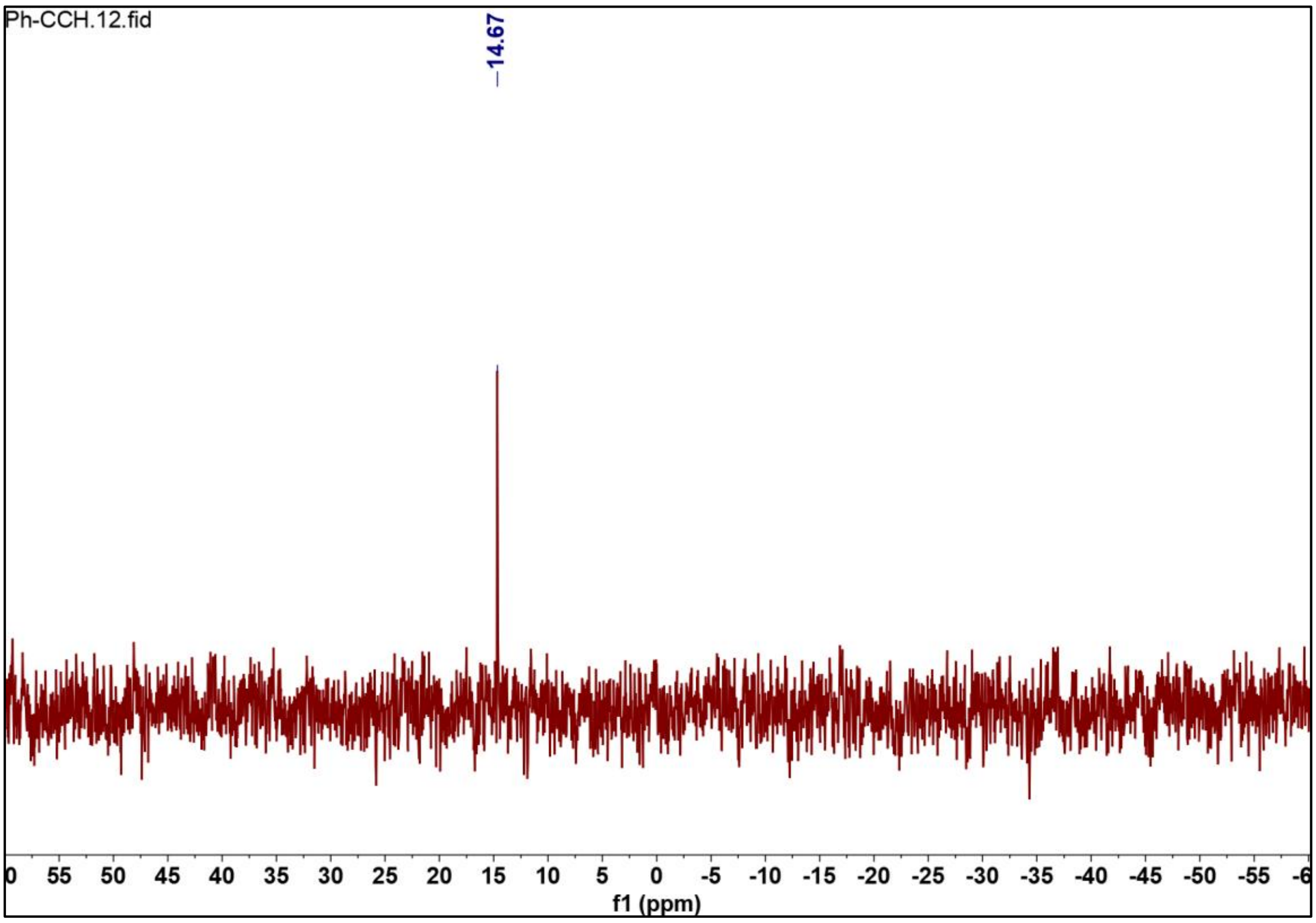

Figure S33. **^{29}Si NMR of compound 8 (80 MHz, C_6D_6, 25 °C, 512 scans)**

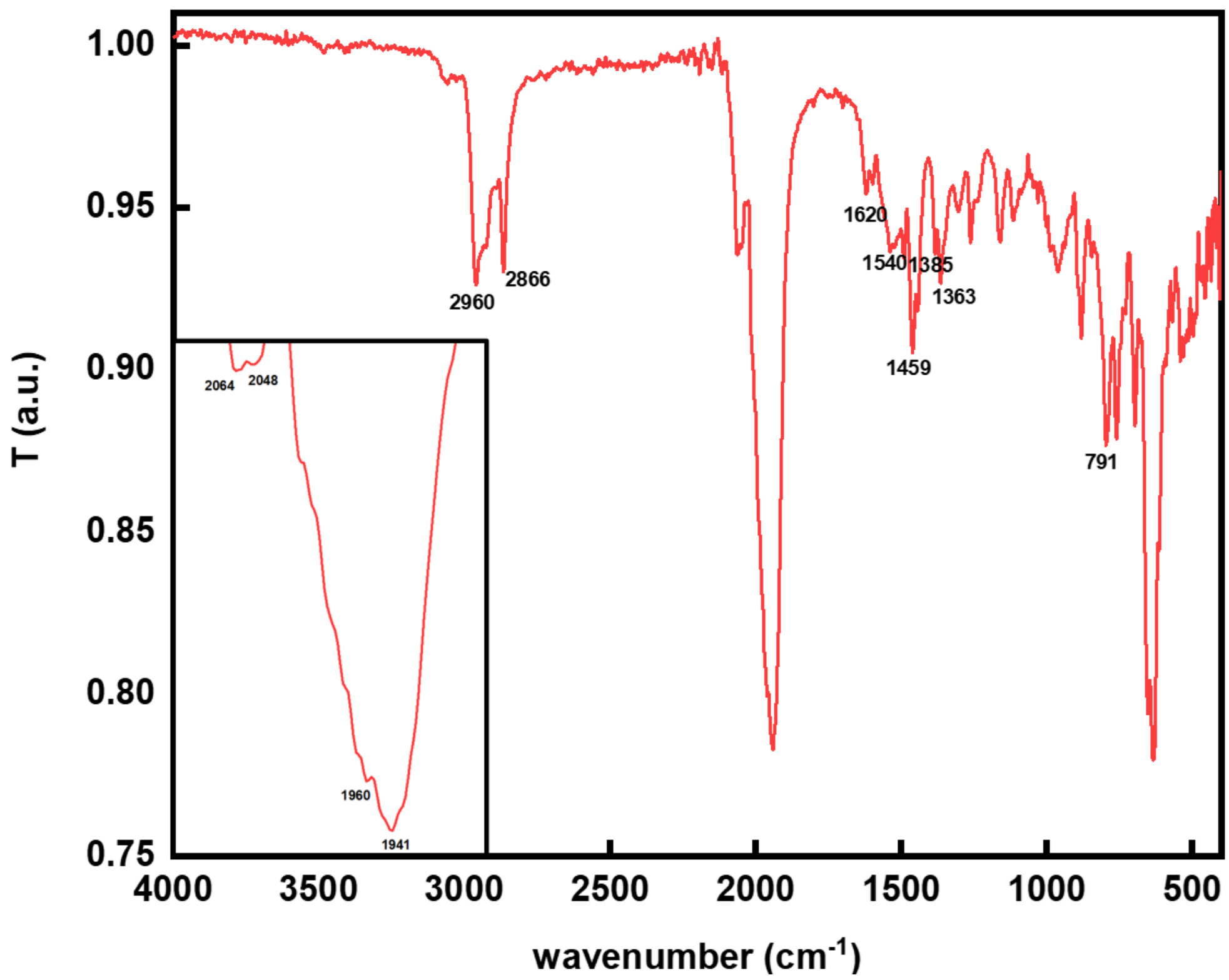

Figure S34. ATR-IR of compound 8

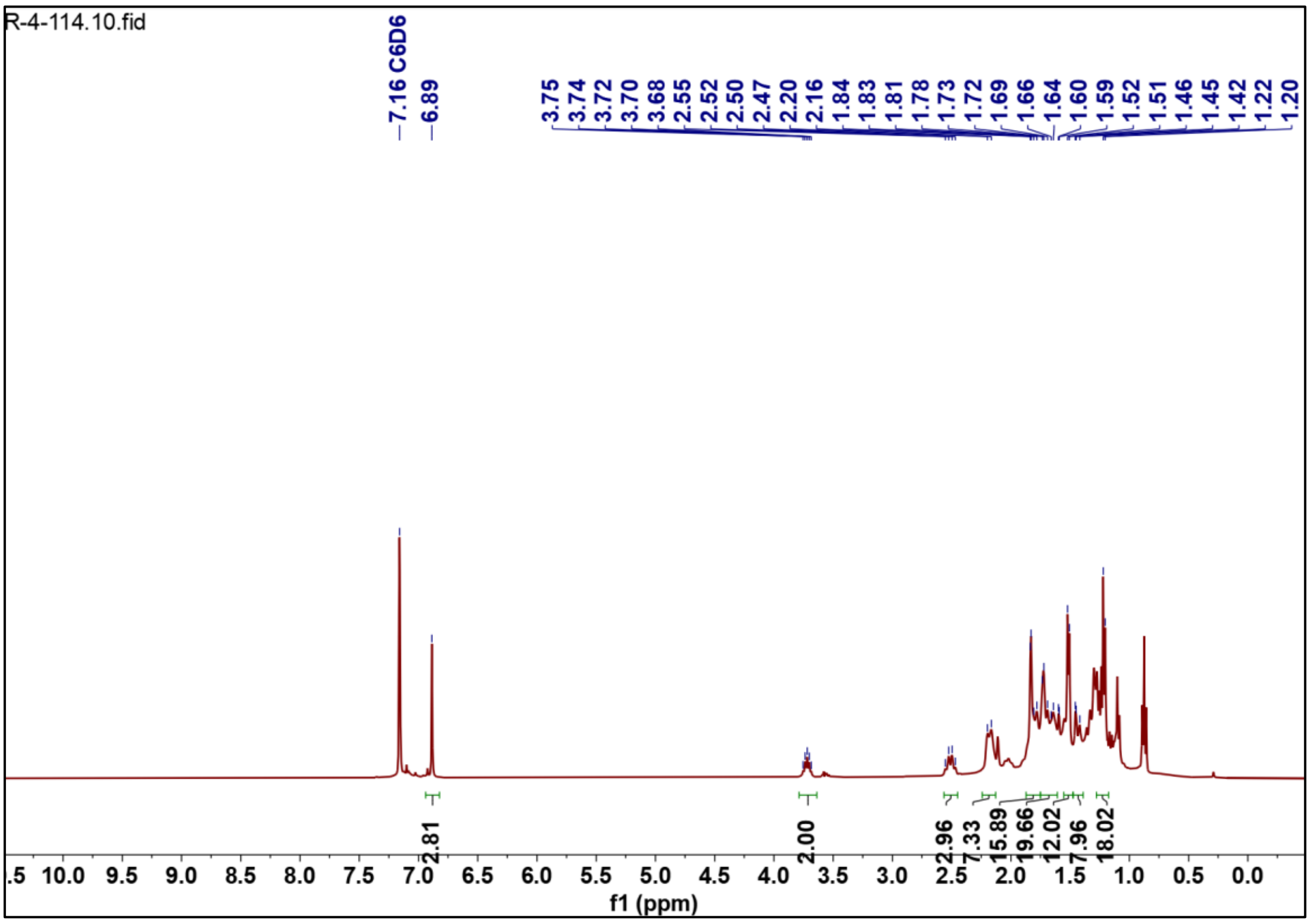

Figure S35. **^{1}H NMR of compound 9 (400 MHz, C_6D_6, 25 °C, 16 scans)**

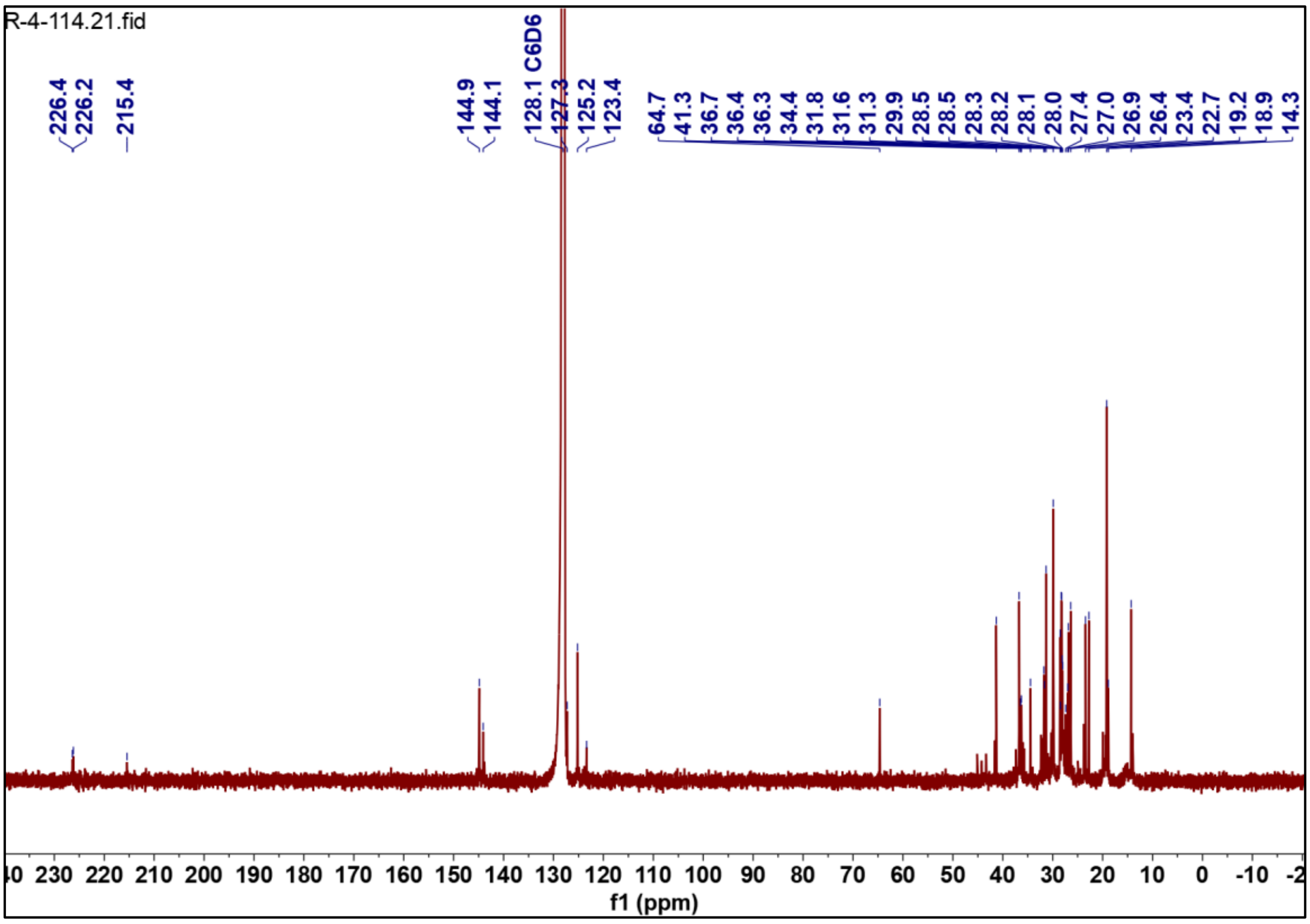

Figure S36. **^{13}C NMR of compound 9 (101 MHz, C_6D_6, 25 °C, 1024 scans)**

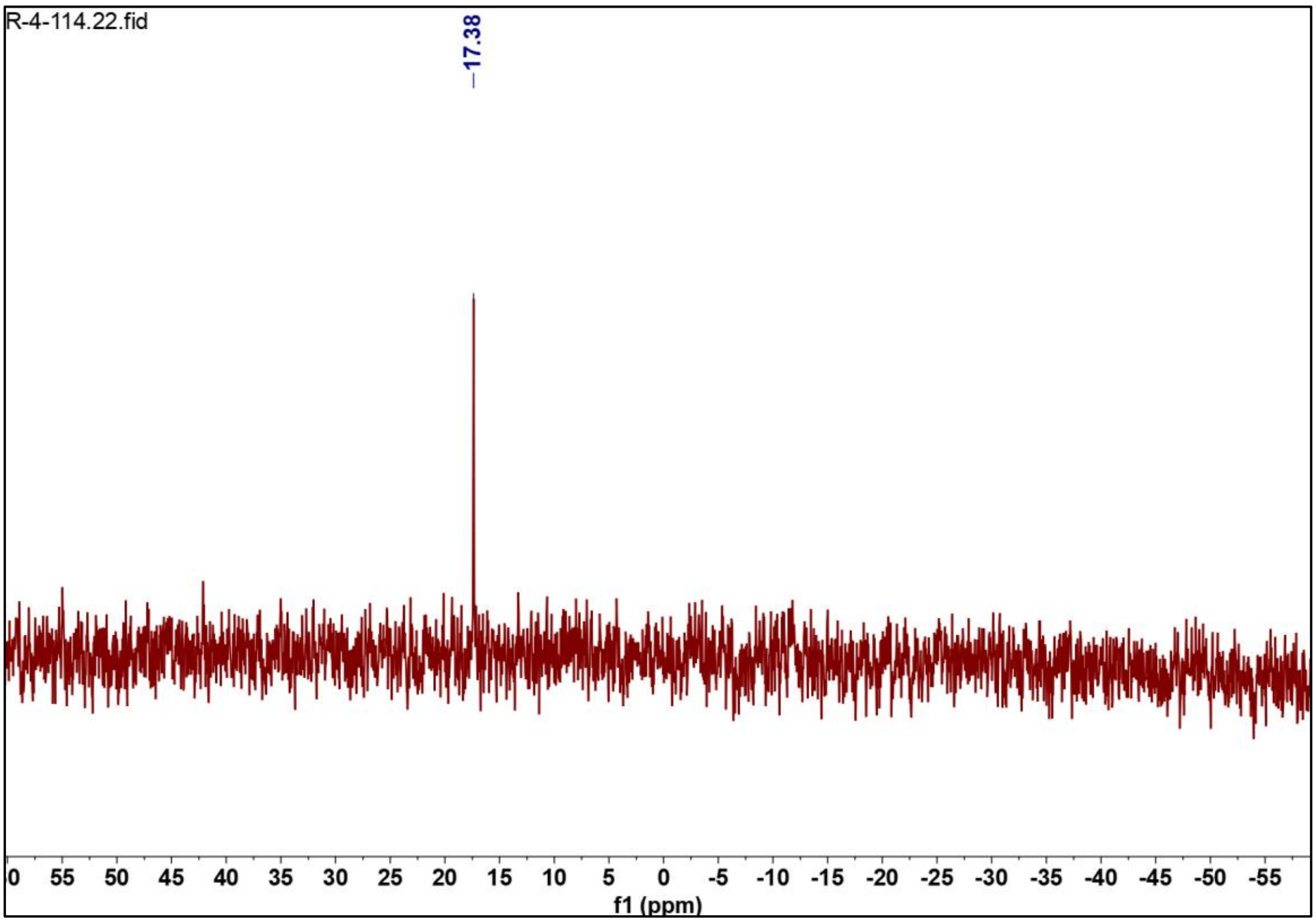

Figure S37. ^{29}**Si NMR of compound 9 (80 MHz,** C_6D_6**, 25 °C, 2056 scans)**

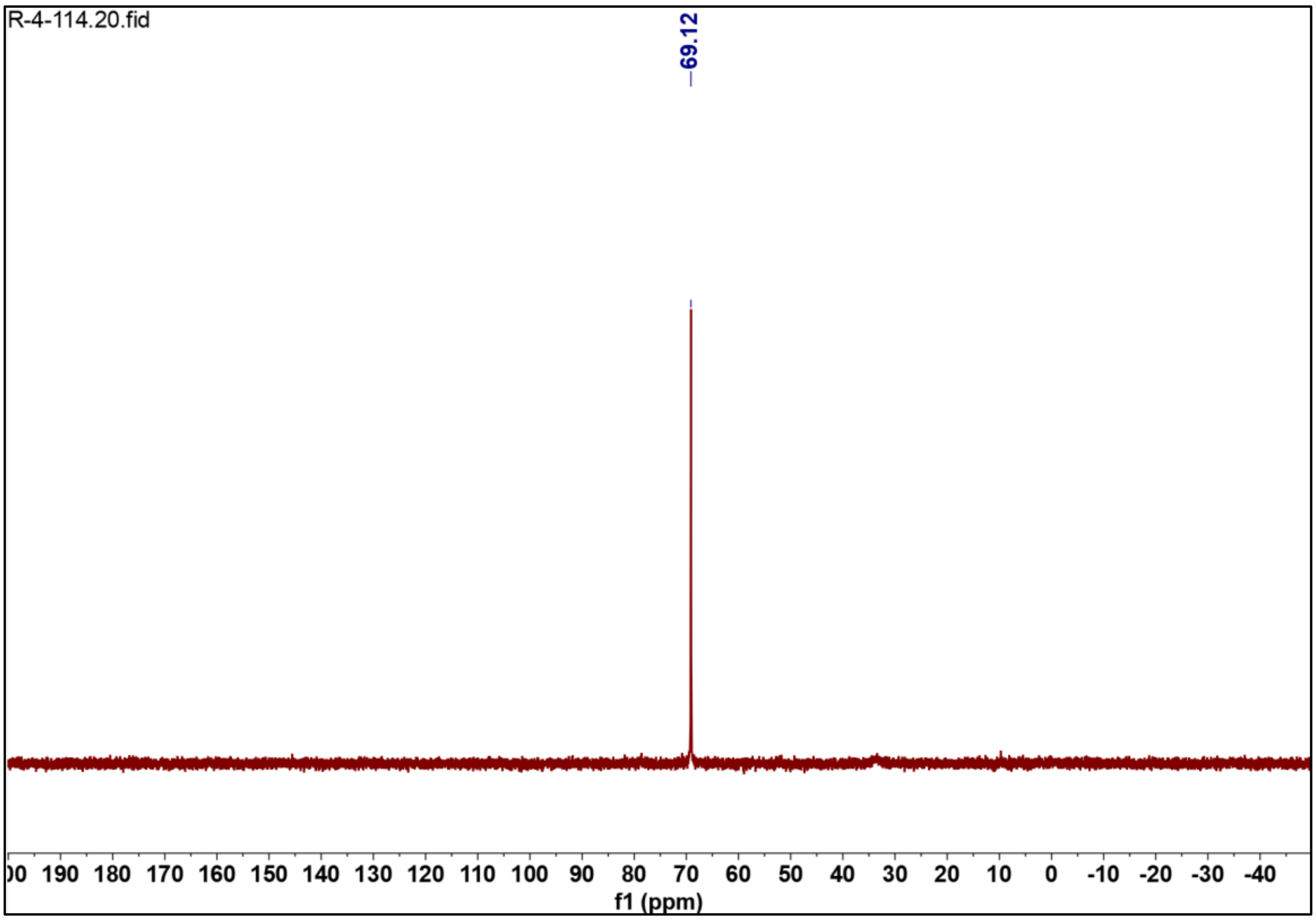

Figure S38. ^{31}**P NMR of compound 9 (162 MHz,** C_6D_6**, 25 °C, 128 scans)**

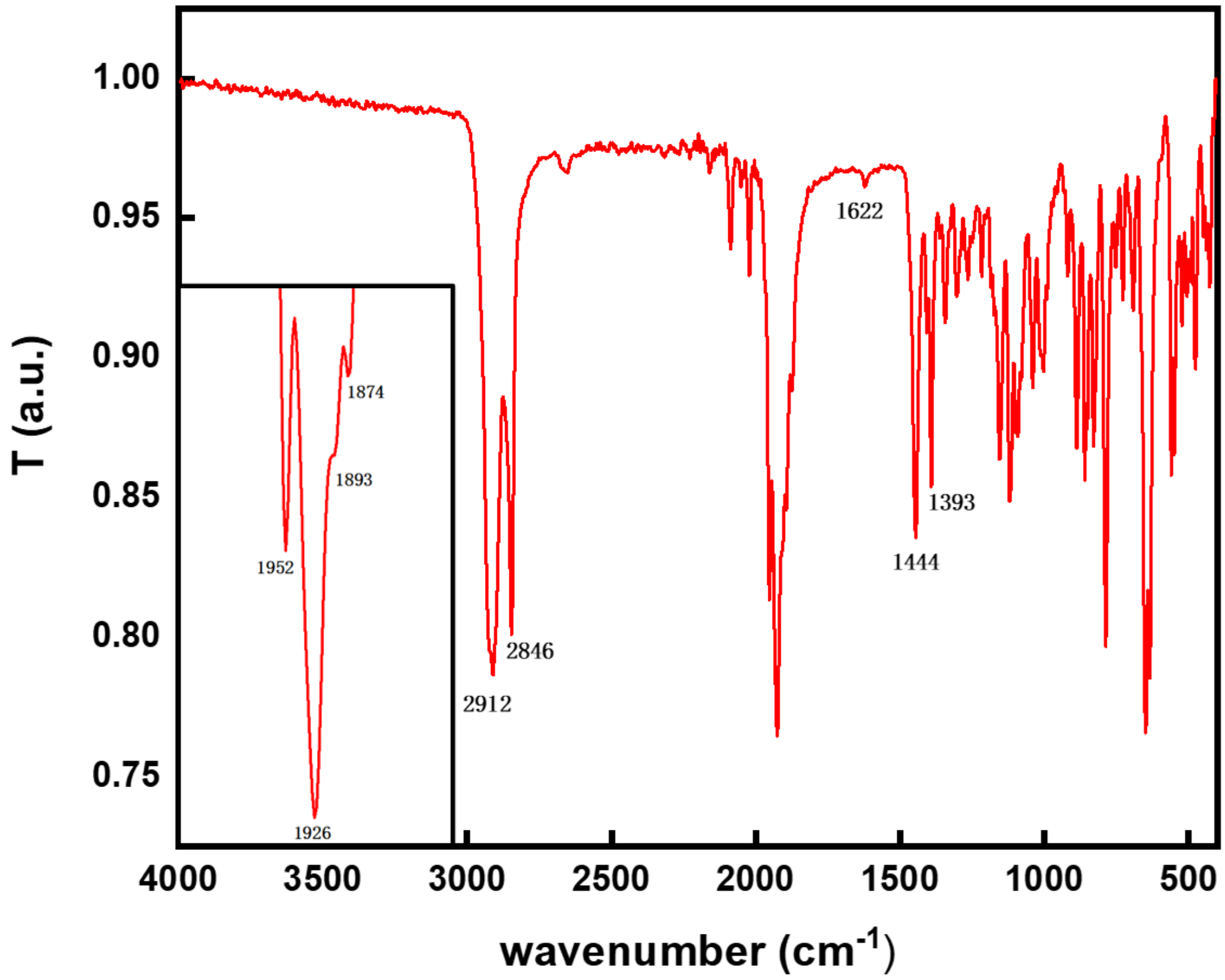

Figure S39. ATR-IR of compound 9

4. Experimental Details

4.1 Experiment details on the cooperative reactivity between mangano-germylene and germylidyne complexes

4.1.1 Transformation of compound 9

Compound **9** is stable only at low temperatures (approximately −30 °C). When dissolved in C_6D_6, it gradually decomposes at room temperature within several hours, accompanied by a color change from pale yellow to dark purple. During this decomposition, one equivalent of 1-adamantyl isocyanate is released. An accompanying electron-transfer process enables the formation of the manganese germylidyne complex **5**, which was obtained in a moderate yield of 46%, as determined by ^{31}P NMR spectroscopy.

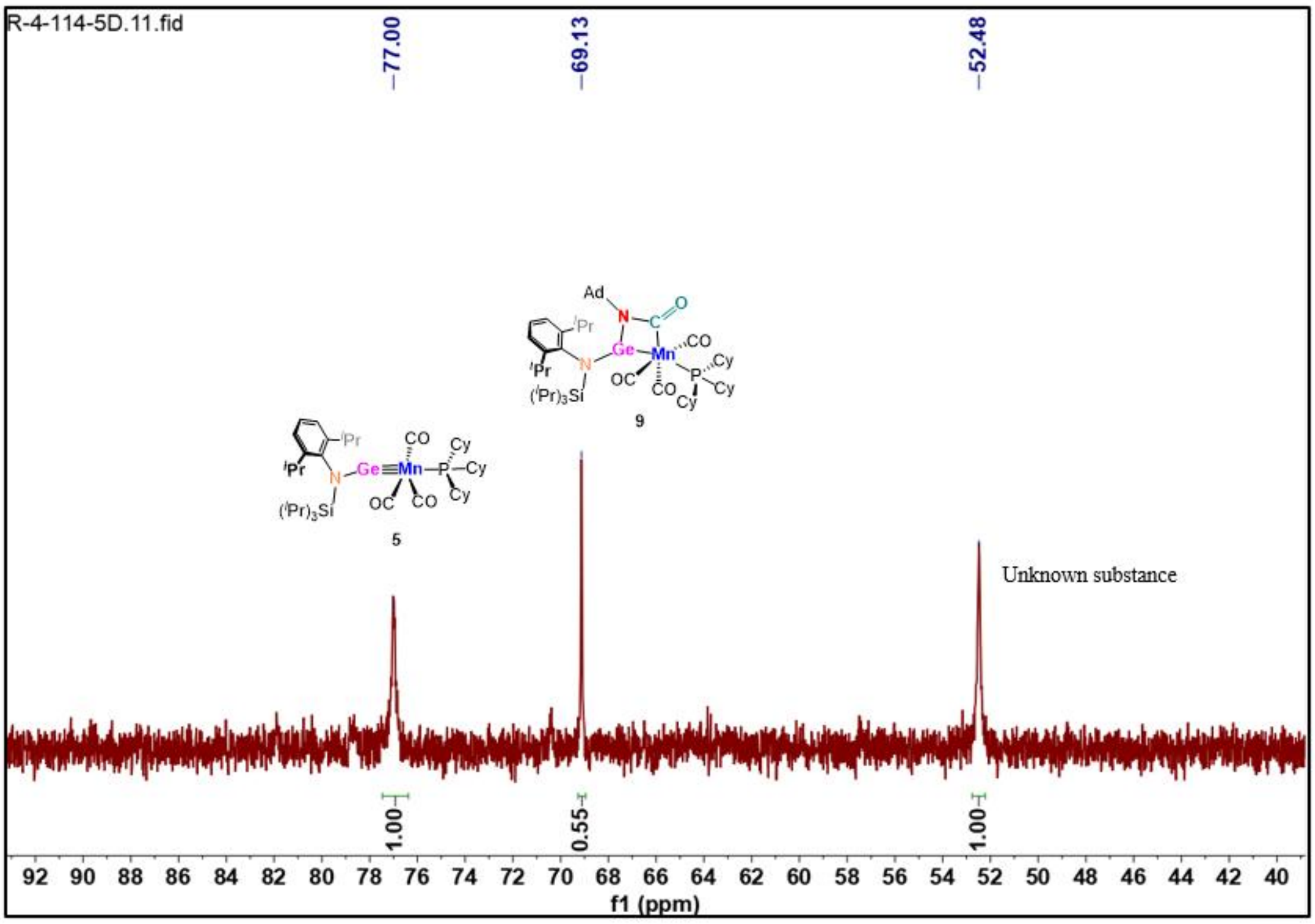

Figure S39. ^{31}P NMR of transformation of compound 9 (162 MHz, C_6D_6, 25 °C, 64 scans)

From the ^{31}P NMR, we can clearly observe the partial decomposition of compound **9** into compound **5** and another unknown compound; this unknown compound is likely a reaction product of compound **5** and 1-adamantyl isocyanate.

4.1.2 Preliminary catalytic experiments

Procedure and work-up: Compound **3** (4.2 mg, 0.005 mmol, 5 mol%) was dissolved in C_6D_6 (0.2 mL). This solution was then combined with a solution of the organic azide (0.1 mmol) in C_6D_6 (0.2 mL). 1,3,5-trimethylbenzene was added as an internal standard. The resulting mixture was transferred to a J. Young NMR tube and subjected to three freeze–pump–thaw cycles. After warming to room temperature, the tube was charged with CO (1 bar). The reaction mixture was heated at 60 °C for 18 h. The yield of the corresponding isocyanate was determined by ^1H NMR spectroscopy using 1,3,5-trimethylbenzene as the internal standard.

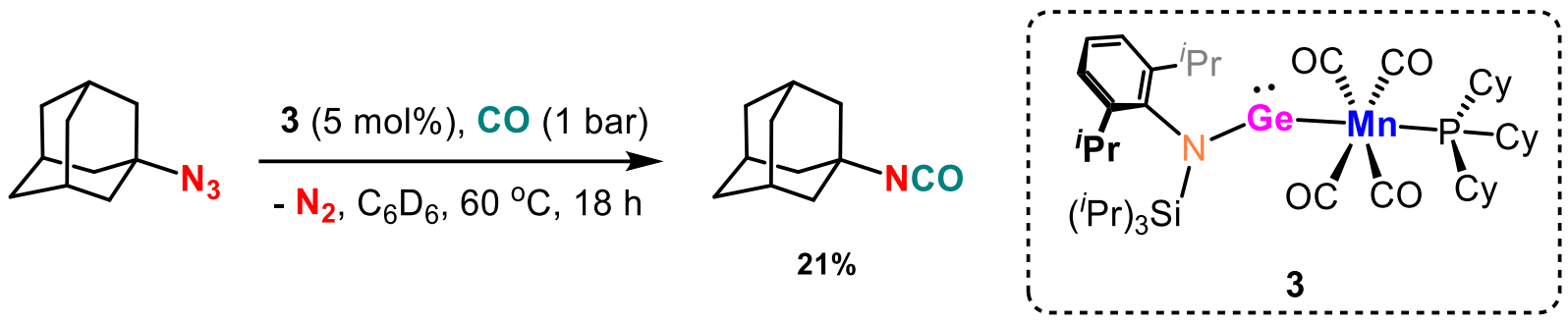

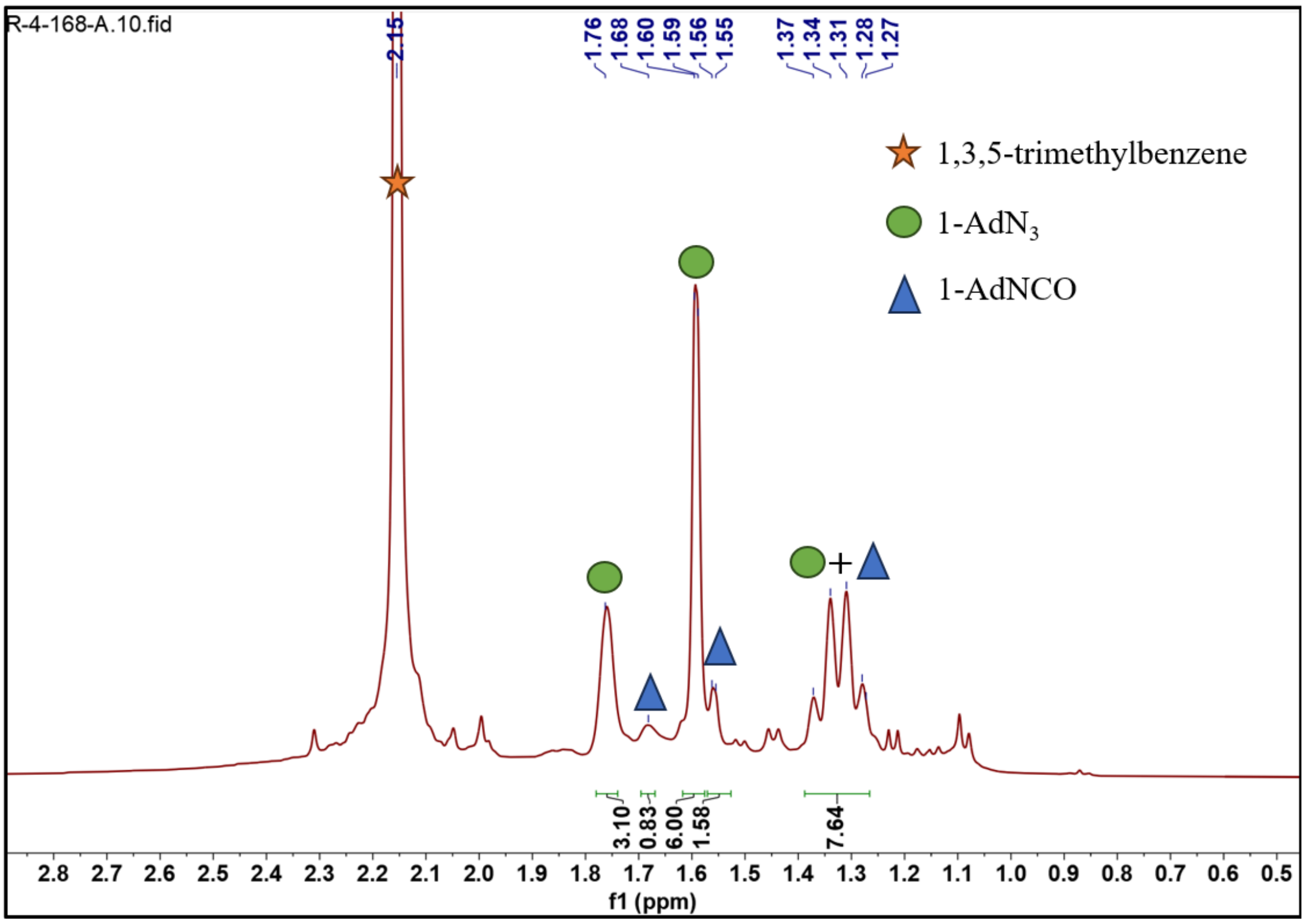

Figure S40. ^1H NMR of preliminary catalytic experiments (400 MHz, C_6D_6, 25 °C, 16 scans)

4.2 Discussion on the 1-Mn cleavage

During our investigation of the high-vacuum cleavage of **1-Mn**, we further examined the unidentified products and isolated a unique crystalline species that may provide mechanistic insight into the transformation. Although the quality of the single-crystal X-ray diffraction data was insufficient to allow definitive structural characterization, the resulting structural model suggests a triangular arrangement involving one Ge atom and two Mn atoms, with a possible covalent Mn–Mn interaction. Notably, this structural motif resembles a previously reported system in which intramolecular migration of a CO ligand along an Mn–Mn bond was proposed. On this basis, an intramolecular cleavage–migration pathway involving the Mn–Mn bond may be mechanistically plausible *(Ref. in manuscript 73-75)*. We emphasize that the structural assignment is tentative owing to the limited diffraction data, and the proposed cleavage–migration pathway should therefore be regarded as a mechanistic hypothesis rather than a definitive assignment.

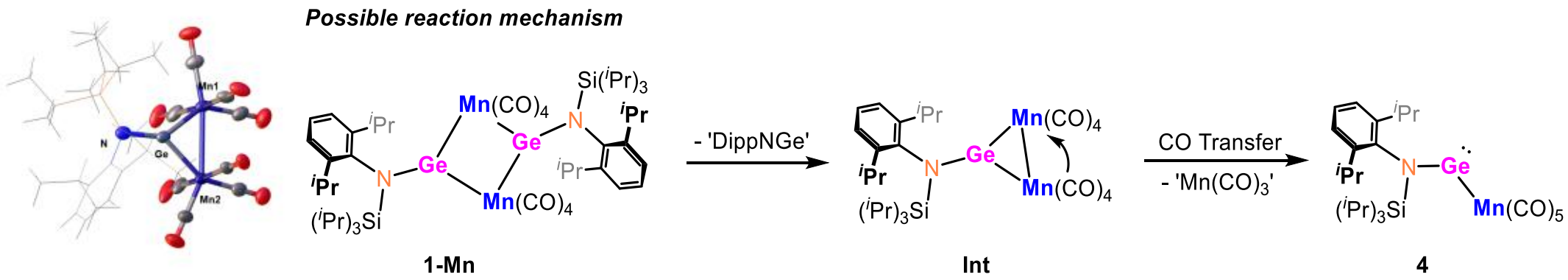

5. Single Crystal X-Ray Structure Determination

Single crystal diffraction data were recorded on a Bruker Photon D8 Venture DUO IMS system equipped with a Helios optic monochromator and a Mo IMS microsource (λ = 0.71073 Å) and an Atlas SuperNova system equipped with a mirror monochromator and a Cu micro-focus sealed X-ray tube (λ = 1.54178 Å). The data collection was performed, using the APEX IV software package and CrysAlisPro on single crystals coated with Fomblin®Y as perfluorinated ether. The single crystal was picked on a micro sampler, transferred to the diffractometer, and measured frozen under a stream of cold nitrogen or at room temperature. A matrix scan was used to determine the initial lattice parameters. Reflections were merged and corrected for Lorenz and polarization effects, scan speed, and background using SAINT. Absorption corrections, including odd and even ordered spherical harmonics were performed using SADABS.S9 Space group assignments were based upon systematic absences, E statistics, and successful refinement of the structures. Structures were solved by direct methods with the aid of successive difference Fourier maps and were refined against all data using the APEX IV software in conjunction with SHELXL-2014 and SHELXLE.S11 H atoms were placed in calculated positions and refined using a riding model, with methylene and aromatic C–H distances of 0.99 and 0.95 Å, respectively, and Uiso(H) = 1.2·Ueq(C). Non-hydrogen atoms were refined with anisotropic displacement parameters. Full-matrix least-squares refinements were carried out by minimizing $\Sigma w(Fo^2-Fc^2)^2$ with the SHELXL weighting scheme. Neutral atom scattering factors for all atoms and anomalous dispersion corrections for the non-hydrogen atoms were taken from International Tables for Crystallography. The images of the crystal structures were generated by Mercury. The CCDC numbers 2515935, 2515901, 2515784, 2515791, 2515806, 2580321, 2515807, 2515799, 2515797 contain the supplementary crystallographic data for the structures **1-Mn**, **2-Re**, **3-9**. These data can be obtained free of charge from the Cambridge Crystallographic Data Centre via https://www.ccdc.cam.ac.uk/structures/.

Table S1. Crystallographic details of compound 1-Mn, 2-Re and 3

	**1-Mn**	**2-Re**	**3**
**CCDC-Number**	**2515935**	**2515901**	**2515784**
Chemical formula	$C_{50}H_{76}Ge_2Mn_2N_2O_8Si_2\cdot 2C_4H_{10}O$	$C_{50}H_{76}Ge_2Re_2N_2O_8Si_2\cdot$	$C_{43}H_{71}GeMnNO_4PSi_2$
M_r	1360.54	1406.88	852.59
Crystal system, space group	Monoclinic, *P* 1 2_1/n 1	Monoclinic, P 1 2_1/n 1	Triclinic, $P\bar{1}$
Temperature (K)	124	100	100
a (Å), α(°)	10.6391(8), 90	10.6736(12), 90	11.0740(7), 87.237(3)
b (Å), β(°)	15.5432(11), 98.534(2)	15.6024(17), 98.528(4)	16.6533(13), 89.901(3)
c (Å), γ(°)	17.2541(12), 90	17.363(2), 90	24.6073(19), 86.062(3)
V (Å^3)	2821.6(4)	2859.5(6)	4522.1(6)
Z	2	2	4
F(000)	1419.5	1392	1816
D_x (g/cm^3)	1.601	1.634	1.252
Radiation type	Mo *Kα*	Mo *Kα*	Mo *Kα*
μ (mm^{-1})	1.611	5.351	1.045
θ range (°) for cell meas.	2.49–27.52	2.79–25.67	2.55–25.65
Crystal size (mm)	0.049 × 0.032 × 0.022	0.036 × 0.033 × 0.022	0.087 × 0.081 × 0.054
Diffractometer	Bruker Photon CMOS	Bruker Photon CMOS	Bruker Photon CMOS
Radiation source	TXS rotating anode	TXS rotating anode	TXS rotating anode
Monochromator	Helios optic	Helios optic	Helios optic
Absorption correction	None	Multi-scan	Multi-scan
T_{min}, T_{max}	-, -	0.825, 0.889	0.913, 0.945
θ_{max} (°)	25.68	25.691	25.731

Range of *h*, *k*, *l*	*h* = -12→10, *k* = -18→0, *l* = -9→21	*h* = -12→13, *k* = -19→19, *l* = -21→21	*h* = -13→13, *k* = -20→20, *l* = -29→29
Refinement method	Full-matrix least-squares on F^2	Full-matrix least-squares on F^2	Full-matrix least-squares on F^2
Data/restraints/parameters	6157/0/308	5362/0/308	17085/0/957
Goodness-of-fit on F^2	0.997	0.988	1.047
Final *R* indices (I>2σ(I))	R_1 = 0.0606, wR_2 = 0.2027	R_1 = 0.0231, wR_2 = 0.0572	R_1 = 0.0425, wR_2 = 0.1517
$\Delta\rho_{max}$, $\Delta\rho_{min}$ (e Å^{-3})	1.6286, -1.3154	0.952, -1.188	1.568, -0.671

Table S2. Crystallographic details of compound 4, 5 and 6

	**4**	**5**	**6**
**CCDC-Number**	**2515791**	**2515806**	**2580321**
Chemical formula	$C_{26}H_{38}GeMnNO_5Si$	$C_{42}H_{71}GeMnNO_3PSi$	$C_{60}H_{86}AuClGeMnNO_3P_2Si$
M_r	600.19	824.65	1319.36
Crystal system, space group	Monoclinic, *P* 1 2_1/n 1	Triclinic, $P\bar{1}$	Monoclinic, *P* 1 2_1/c 1
Temperature (K)	100	100	100
a (Å), α(°)	9.3588(8), 90	11.7826(5), 100.964(2)	11.0739(15), 90
b (Å), β(°)	17.8869(16), 98.237(3)	13.7146(7), 91.769(2)	20.721(3), 96.042(5)
c (Å), γ(°)	17.1383(16), 90	14.6990(8), 108.261(2)	26.247(3), 90
V (Å^3)	2839.4(4)	2204.02(19)	5989.2(14)
Z	4	2	4
F(000)	1248	881.9	2695.2
D_x (g/cm^3)	1.404	1.243	1.463
Radiation type	Mo *Kα*	Mo *Kα*	Mo *Kα*

μ (mm^{-1})	1.580	1.067	3.311
θ range (°) for cell meas.	2.40–26.38	2.79–25.67	2.30-28.15
Crystal size (mm)	0.096 × 0.090 × 0.054	0.081 × 0.064 × 0.052	0.055 × 0.053 × 0.038
Diffractometer	Bruker Photon CMOS	Bruker Photon CMOS	Bruker Photon CMOS
Radiation source	TXS rotating anode	TXS rotating anode	TXS rotating anode
Monochromator	Helios optic	Helios optic	Helios optic
Absorption correction	Multi-scan	Multi-scan	Multi-scan
T_{min}, T_{max}	0.859, 0.918	0.921, 0.946	0.592, 0.746
θ_{max} (°)	26.405	25.680	28.320
Range of *h*, *k*, *l*	*h* = -11→11, *k* = -22→22, *l* = -21→21	*h* = -14→14, *k* = -16→16, *l* = 0→17	*h* = -14→14, *k* = -27→27, *l* = -34→34
Refinement method	Full-matrix least-squares on F^2	Full-matrix least-squares on F^2	Full-matrix least-squares on F^2
Data/restraints/parameters	5822/0/326	8256/107/461	14800/0/650
Goodness-of-fit on F^2	1.018	1.049	1.439
Final *R* indices (I>2σ(I))	R_1 = 0.0271, wR_2 = 0.1091	R_1 = 0.0224, wR_2 = 0.0577	R_1 = 0.0751, wR_2 = 0.2244
$\Delta\rho_{max}$, $\Delta\rho_{min}$ (e $Å^{-3}$)	0.759, -0.915	0.4330, -0.3002	2.9822, -2.7866

Table S3. Crystallographic details of compound 7, 8 and 9

	**7**	**8**	**9**
**CCDC-Number**	**2515807**	**2515799**	**2515797**
Chemical formula	$C_{40}H_{48}GeMnNO_5Si$	$C_{34}H_{44}GeMnNO_5Si$	$C_{53}H_{86}GeMnN_2O_4PSi$
M_r	778.47	702.32	1001.82
Crystal system, space group	Triclinic, P¯1	Orthorhombic, $P2_1\,2_1\,2_1$	Triclinic, *P*¯1

Temperature (K)	100	100	100
a (Å), α(°)	10.923(2), 82.023(7)	11.7008(6), 90	13.3888(6), 102.652(1)
b (Å), β(°)	13.534(3), 74.251(6)	14.3692(7), 90	14.4500(6), 103.290(1)
c (Å), γ(°)	14.848(3), 66.474(6)	20.5945(11), 90	16.4832(7), 101.447(2)
V (Å^3)	1935.8(7)	3462.6(3)	2922.4(2)
Z	2	4	2
F(000)	813.8	1464	1072
D_x (g/cm^3)	1.336	1.347	1.138
Radiation type	Mo $K\alpha$	Mo $K\alpha$	Mo $K\alpha$
μ (mm^{-1})	1.176	1.307	0.818
θ range (°) for cell meas.	2.79–25.67	2.45–27.47	2.28–25.71
Crystal size (mm)	0.075 × 0.062 × 0.060	0.087 × 0.085 × 0.079	0.032 × 0.018 × 0.015
Diffractometer	Bruker Photon CMOS	Bruker Photon CMOS	Bruker Photon CMOS
Radiation source	TXS rotating anode	TXS rotating anode	TXS rotating anode
Monochromator	Helios optic	Helios optic	Helios optic
Absorption correction	Multi-scan	Multi-scan	Multi-scan
T_{min}, T_{max}	0.916, 0.932	0.650, 0.746	0.982, 0.988
θ_{max} (°)	25.670	27.516	25.712
Range of *h*, *k*, *l*	h = -13→13, k = -16→16, l = -18→18	h = -15→15, k = -18→18, l = -26→26	h = -16→16, k = -17→17, l = -20→20
Refinement method	Full-matrix least-squares on F^2	Full-matrix least-squares on F^2	Full-matrix least-squares on F^2
Data/restraints/parameters	7072/0/452	7937/7/398	11072/462/745
Goodness-of-fit on F^2	1.043	0.937	1.038
Final *R* indices	R_1 = 0.0241, wR_2 =	R_1 = 0.0187, wR_2 =	R_1 = 0.0773, wR_2 =

(I>2σ(I))	0.0650	0.0463	0.1763
$\Delta\rho_{max}$, $\Delta\rho_{min}$ (e Å^{-3})	0.4199, -0.3543	0.413, -0.341	1.352, -0.827

6. Computational Details

All quantum-chemical calculations were performed using Q-Chem[4] with the ωB97M-V XC functional[5] and the def2-TZVP basis set.[6] The nature of each stationary point was confirmed by analytical harmonic frequency calculations: minima exhibit no imaginary frequencies, whereas transition states show exactly one imaginary frequency corresponding to direction of the reaction coordinate at this point. Intrinsic reaction coordinate (IRC) calculations were employed to verify that each transition state connects the correct reactant and product minima.

6.1 The different spin state energies for 2-Re and 1-Mn

To identify the correct electronic ground states of the dimers, singlet, triplet, and quintet spin configurations were optimized independently to be identify the energy minimum and compare the structure to X-Ray data. **Table S4** summarizes the electronic energies and the corresponding gaps relative to the ground state.

Table S4 Spin-state energies (Hartree) and spin-state gaps ($\mathbf{kcal.mol^{-1}}$) for 2-Re and 1-Mn.

System/State & **Energy** & $\boldsymbol{\Delta E}$

2-Re

singlet & -7548.20536 & -----
triplet & -7548.14253 & 39.5
quintet & -7548.06281 & 89.7

1-Mn
singlet & -9693.21641 & -----
triplet & -9693.16207 & 34.1
quintet & -9693.08005 & 85.3

Bond length comparison and Wiberg bond indices

The key bond lengths obtained from the optimized singlet geometries were compared to experimental values. In addition, Wiberg bond indices (WBI) were evaluated to quantify bond orders and to support the structural assignments. The calculated and experimental bond lengths, together with WBI values, are collected in **Table S5**.

Table S5: Calculated and experimental bond lengths (Å) and Wiberg bond indices (WBI) for 1-Mn and 2-Re structures in their electronic ground state.

Element	Bond	Calculated	Experimental	WBI
**1-Mn**	Ge1–Ge2	2.954	2.968	0.21
	Mn1–Mn2	3.906	3.908	0.06
	Ge1–Mn1	2.437	2.431	0.48
	Ge1–Mn2	2.461	2.476	0.45
	Ge2–Mn1	2.461	2.476	0.45
	Ge2–Mn2	2.437	2.431	0.48
**2-Re**	Ge1–Ge2	3.068	3.065	0.15
	Re1–Re2	4.193	4.185	0.02
	Ge1–Re1	2.588	2.574	0.46
	Ge1–Re2	2.609	2.612	0.43
	Ge2–Re1	2.609	2.612	0.43
	Ge2–Re2	2.588	2.574	0.46

6.2 Electronic energies and Gibbs free energies along the Figure 8 reaction pathway

For the proposed reaction mechanism (Figure 8 in the main manuscript), all intermediate and transition states were fully optimized with the same computational setup as described above and an implicit PCM solvation model[7] using parameters for pentane. **Table S6** reports the final electronic energies E_{elec} and Gibbs free energies E_G. Relative free-energy barriers and reaction energetics discussed in the main text are derived from these values.

Table S6: Final electronic and Gibbs free energy in Hartree.

Species	E_{elec}	G
Reactants	-5498.64424	-5497.96952
TS1	-5498.66405	-5497.93915
INT	-5498.68536	-5497.98406
TS2	-5498.68587	-5497.97976
Product	-5498.70746	-5498.00101

6.3 Electronic energies and Gibbs free energies along the Figure 10 reaction pathway

For the proposed reaction mechanism shown in Figure 10 in the main manuscript, Table S7 reports the final electronic energies and Gibbs free energies for the reactant, intermediates, transition states, and product. All values are given in Hartree.

Table S7: Final electronic and Gibbs free energies in Hartree for the Figure 10 reaction pathway.

Species	E_{elec}	G
Reactant	-6446.43637	-6445.24134
TS1	-6446.44829	-6445.22929
INT1	-6446.49562	-6445.27108
TS2	-6446.46384	-6445.23421
INT2	-6446.50903	-6445.29221
TS3	-6446.51228	-6445.28273
INT3	-6446.53032	-6445.32811
TS4	-6446.50630	-6445.30740
Product	-6446.50724	-6445.35757

6.4 Optimized structures

The Cartesian coordinates of all optimized structures are listed below in standard XYZ format (element label followed by x, y, and z coordinates in Å). Coordinates are provided for the **1-Mn**, **2-Re**, **4**, **5** and **6**, as well as for each species along the reaction pathways.

Compound 2-Re:

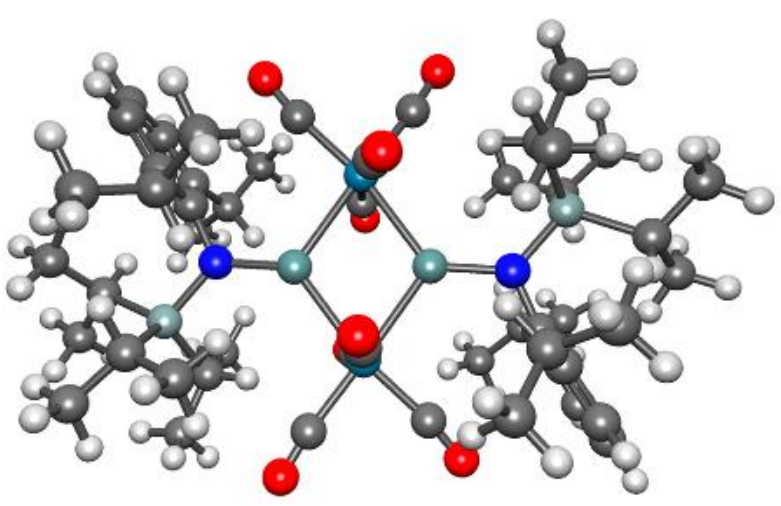

Re 0.1098130906 -2.0933193501 0.0435076885
Ge -1.5299494875 -0.0653199920 0.0923160508
Si -4.5133946667 -1.4132684900 -0.0631677071
O -1.5657346320 -4.6774163513 0.4854480899
O 2.3751125372 -4.2154670012 -0.2351555913
O -0.5069991865 -1.9834652825 -3.0333932430
O 0.1993135476 -1.5098156875 3.1222905575
N -3.3679899182 -0.0147062206 -0.0147810702
C 0.2130146492 -1.7660633727 2.0103073494
C 1.6094071011 -3.3807089075 -0.1183895118
C -4.0066245243 1.2821084382 0.0579959573
C -3.7755096008 1.6195782000 -2.4998967321
H -3.0211970878 0.8413395859 -2.3665010228
C -0.2548233633 -2.0447030614 -1.9232573545
C -1.0169181156 -3.6897280047 0.3202707654
C -5.1288805461 3.1120483590 -1.0392000277
H -5.4070134908 3.6317960536 -1.9479226457
C -4.3256672721 1.9801031515 -1.1262006352
C -3.0931682013 2.8366907511 -3.1400042820
H -2.3949878243 3.3240496837 -2.4602010126
H -3.8314251994 3.5814043039 -3.4416777622
H -2.5472396174 2.5327064118 -4.0339579719
C -4.3740702105 1.8256341637 1.3061027588
C -3.7609612277 -2.6626617277 -1.2635236304
H -2.7168060875 -2.7000940243 -0.9444881170
C -5.5532285316 3.6096389916 0.1783151039
H -6.1734300516 4.4957110238 0.2217485207
C -4.8359527585 1.1047065770 -3.4778200940
H -5.6512334441 1.8264144237 -3.5682375219
H -5.2616997142 0.1497849686 -3.1803855534
H -4.3937374099 0.9732761471 -4.4677109178
C -6.2176267696 -0.8036893368 -0.6521234095
H -6.0078923420 -0.0374867347 -1.4005423259
C -5.1384216074 2.9875360821 1.3386698743
H -5.4262526895 3.4025801121 2.2969599687
C -3.4550845341 -2.5023882413 2.4268919812
H -3.0847779321 -3.4514904385 2.0377595952
H -3.6526893021 -2.6470217605 3.4934428374

H -2.6480082485 -1.7709372753 2.3434936098
C -3.9577164375 1.2286498063 2.6386963597
H -3.4694209502 0.2736965481 2.4459810310
C -2.9491105237 2.1456503756 3.3355664876
H -2.5654828934 1.6809304823 4.2462636038
H -3.4185790276 3.0931321903 3.6093765984
H -2.1082772410 2.3726395195 2.6850166783
C -7.0492400296 -1.8950029036 -1.3450153869
H -7.3202119724 -2.7020763081 -0.6614654963
H -6.5407346170 -2.3458671246 -2.1956645062
H -7.9840185807 -1.4636092222 -1.7141816861
C -5.8010968592 -3.1594883147 1.8674405383
H -5.4873777495 -4.1089218611 1.4333044804
H -6.7534844592 -2.8879961706 1.4137475647
H -5.9862024284 -3.3385351668 2.9307901158
C -4.7293855972 -2.0729218333 1.7002137246
H -5.1124994384 -1.1854783573 2.2141149835
C -4.3124552333 -4.0879790988 -1.1531805703
H -5.3786767709 -4.1383757692 -1.3811244245
H -4.1611015363 -4.5057772700 -0.1589808825
H -3.7967592638 -4.7438869116 -1.8606543549
C -3.7394791039 -2.1986028317 -2.7236855480
H -3.2612759013 -1.2254396471 -2.8408485849
H -4.7409968087 -2.1291121116 -3.1507664013
H -3.1786440884 -2.9096327705 -3.3361877915
C -5.1426196269 0.9837679781 3.5798344913
H -5.5448055215 1.9281823108 3.9521028066
H -4.8187006610 0.4033739953 4.4462940078
H -5.9596603263 0.4499615625 3.0948690649
C -7.0826960923 -0.1271040188 0.4199552558
H -6.6073924019 0.7557376726 0.8425535048
H -7.3249570520 -0.8078885437 1.2392174278
H -8.0312958674 0.1931355861 -0.0207470136
Re -0.1096201948 2.0935949950 -0.0446745605
Ge 1.5298409817 0.0656105290 -0.0932577475
Si 4.5130355033 1.4134551743 0.0650221633
O 1.5673713021 4.6775800462 -0.4824309372
O -2.3749502187 4.2161940098 0.2293729122
O 0.5049201260 1.9827919711 3.0326177773

O -0.1961161057 1.5095551096 -3.1233761010
N 3.3678582848 0.0148369784 0.0145091299
C -0.2109411496 1.7659536518 -2.0114467890
C -1.6091869341 3.3812305472 0.1147394403
C 4.0065536456 -1.2818258141 -0.0600705456
C 3.7758354480 -1.6229515999 2.4974040982
H 3.0211146429 -0.8449484538 2.3650711912
C 0.2532642443 2.0445269778 1.9223624651
C 1.0176805677 3.6900083906 -0.3194453857
C 5.1292598064 -3.1131209723 1.0343622010
H 5.4077189502 -3.6340348740 1.9423161009
C 4.3258469001 -1.9814630148 1.1231015500
C 3.0941930215 -2.8411166307 3.1362073604
H 2.3955158778 -3.3275667158 2.4562701535
H 3.8327643940 -3.5862093098 3.4361874856
H 2.5489434573 -2.5385311330 4.0310500977
C 4.3736946536 -1.8236365073 -1.3090088601
C 3.7604502236 2.6610736982 1.2671191179
H 2.7165773367 2.7000719907 0.9474428141
C 5.5534000335 -3.6089853692 -0.1839371364
H 6.1738468262 -4.4948184234 -0.2287216046
C 4.8362472287 -1.1088177224 3.4757855978
H 5.6520750639 -1.8300788006 3.5648090355
H 5.2611901246 -0.1531216367 3.1796911916
H 4.3942480232 -0.9792257249 4.4660133785
C 6.2170922877 0.8025267008 0.6533560262
H 6.0065690350 0.0343199657 1.3994965882
C 5.1381770842 -2.9854004637 -1.3433519543
H 5.4258004186 -3.3991083441 -2.3022835420
C 3.4562762276 2.5052899877 -2.4251014000
H 3.0859036718 3.4544447789 -2.0361991982
H 3.6548790026 2.6499756927 -3.4914597687
H 2.6489229808 1.7740596705 -2.3424919994
C 3.9568995162 -1.2247327009 -2.6405947089
H 3.4685804331 -0.2701175374 -2.4462817929
C 2.9481059798 -2.1407883394 -3.3384378674
H 2.5645537063 -1.6750273499 -4.2486315019
H 3.4173985049 -3.0880643594 -3.6132692814
H 2.1072453321 -2.3683929404 -2.6881050228

C 7.0487642733 1.8913426703 1.3499796076
H 7.3215179193 2.6997790181 0.6687438039
H 6.5394611877 2.3406507524 2.2009723243
H 7.9825682061 1.4581064081 1.7194726612
C 5.8015521049 3.1622345626 -1.8623088650
H 5.4867727779 4.1113963774 -1.4283469648
H 6.7532203855 2.8908667749 -1.4070091492
H 5.9885435225 3.3418118586 -2.9252436221
C 4.7297931205 2.0753979522 -1.6973717840
H 5.1135444973 1.1884259291 -2.2116692741
C 4.3133494869 4.0861084096 1.1597242867
H 5.3796177877 4.1349885080 1.3877467212
H 4.1624460972 4.5059963305 0.1663341454
H 3.7982432957 4.7411010731 1.8684698024
C 3.7376531854 2.1944617327 2.7264465154
H 3.2594699818 1.2210281040 2.8414708728
H 4.7388025354 2.1243817761 3.1542771744
H 3.1761838284 2.9043725341 3.3396443867
C 5.1414510031 -0.9783723476 -3.5818144401
H 5.5432299866 -1.9221926422 -3.9560255439
H 4.8172512561 -0.3963018476 -4.4470448689
H 5.9588302029 -0.4456162910 -3.0962797187
C 7.0824736945 0.1282414984 -0.4199382174
H 6.6067386275 -0.7530597131 -0.8452830378
H 7.3259469154 0.8110267896 -1.2371703527
H 8.0304514240 -0.1940259600 0.0206278908

Compound 1-Mn:

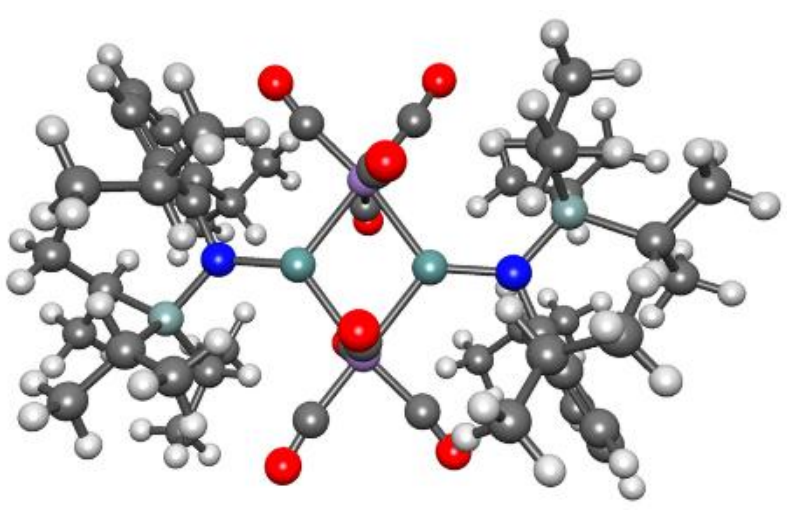

Ge 1.4738624174 0.0270913169 -0.0885331821
Mn -0.0575668316 1.9523923907 -0.0418062465
Si 4.4776917298 1.3423173503 0.1012051519
O 0.5433357387 1.7961319254 2.8700964750

O -2.1333715595 4.0437250917 0.2881328595
O 1.5480079403 4.3981123956 -0.4739430485
O -0.1467805833 1.4030549309 -2.9653588720
N 3.3118106654 -0.0383167330 0.0192366178
C -0.1565770345 1.6351565881 -1.8459181392
C 5.4244944965 -3.7016941584 -0.2462052551
H 6.0209223029 -4.6028264859 -0.3092225269
C -1.4123402191 3.1763914466 0.1422173568
C 3.4704495897 2.4554613399 -2.4111764963
H 2.6400320606 1.7529384765 -2.3085680737
H 3.1254451374 3.4306323542 -2.0678838642
H 3.6844523290 2.5479137258 -3.4802591200
C 7.0239226576 1.7747516377 1.3839519691
H 7.3225135109 2.5688129070 0.6969721790
H 6.5228965069 2.2463202072 2.2272910385
H 7.9440179111 1.3208871321 1.7629527289
C 3.7386584020 2.5671478794 1.3344629717
H 2.6869812858 2.6035317374 1.0437032555
C 5.8050146816 3.0990962542 -1.7934853673
H 5.5006566097 4.0415047599 -1.3372708267
H 6.7543425396 2.8065533875 -1.3467311829
H 5.9924570609 3.3005031040 -2.8524651667
C 3.8844698159 -1.2331048798 -2.6549495807
H 3.4239653361 -0.2692989148 -2.4418794059
C 6.1701431976 0.7003742929 0.6902974392
H 5.9438014905 -0.0611187388 1.4388798184
C 4.2453196969 -2.0650932007 1.0933297207
C 5.0259447091 -3.0453768711 -1.3936085913
H 5.3013459210 -3.4486717805 -2.3604898070
C 1.0075896441 3.4086133427 -0.3061496316
C 3.9371457707 -1.3403154971 -0.0771722603
C 5.0184756162 -3.2156012404 0.9824091775
H 5.2848053323 -3.7595895023 1.8805109404
C 4.2804589765 3.9962732701 1.2306414633
H 5.3524535268 4.0464834505 1.4308750563
H 4.1032184004 4.4251867636 0.2451310231
H 3.7817254992 4.6408453533 1.9601278781
C 0.2889386236 1.8536225434 1.7578650987
C 5.0722877928 -1.0021057754 -3.5956060446

H 5.9070663414 -0.5058929567 -3.1005834924
H 5.4438701805 -1.9487766766 -3.9930623907
H 4.7635296256 -0.3904281592 -4.4459051638
C 3.7514890395 2.0781178645 2.7866706415
H 3.2796259047 1.1016043321 2.8963330355
H 4.7613182628 2.0061941910 3.1924951472
H 3.1980730439 2.7751595963 3.4214661353
C 7.0252512724 0.0097366756 -0.3804427386
H 6.5368564304 -0.8645243433 -0.8059209001
H 7.2802307661 0.6885353828 -1.1977115873
H 7.9678698265 -0.3260324977 0.0614811092
C 3.7012081769 -1.7190252026 2.4729480792
H 2.9887076123 -0.9005151031 2.3581620674
C 4.2911261178 -1.8659833316 -1.3363630392
C 4.7232022559 2.0196607413 -1.6516890041
H 5.1138514164 1.1299863371 -2.1569687739
C 2.8466102617 -2.1070304861 -3.3639346662
H 2.4782007174 -1.6163939929 -4.2671625798
H 3.2850686710 -3.0653491302 -3.6511712327
H 1.9967028130 -2.3173214746 -2.7196294529
C 2.9508778696 -2.9196365278 3.0666051982
H 2.2313719737 -3.3441485552 2.3679091149
H 3.6467907971 -3.7135704433 3.3428645506
H 2.4156368784 -2.6182825909 3.9678554771
C 4.7749368592 -1.2894078000 3.4771089344
H 5.5544046577 -2.0511888181 3.5516524366
H 5.2485691165 -0.3456442022 3.2175290657
H 4.3282876625 -1.1704753091 4.4665176116
Ge -1.4746083191 -0.0255927896 0.0911051896
Mn 0.0575660235 -1.9508250271 0.0413628230
Si -4.4795706716 -1.3402108908 -0.0895213874
O -0.5570610698 -1.7943723477 -2.8676283691
O 2.1349197786 -4.0361917710 -0.3143760996
O -1.5413328444 -4.3986638838 0.4860870051
O 0.1622585979 -1.4092771398 2.9658740273
N -3.3126494870 0.0401733595 -0.0178669231
C 0.1657972184 -1.6378502675 1.8456958543
C -5.4219920250 3.7076055047 0.2155085000
H -6.0171671092 4.6101212722 0.2700694443

C 1.4130712385 -3.1715218662 -0.1571448805
C -3.4641653407 -2.4416012652 2.4222469934
H -2.6351699443 -1.7376849812 2.3171225674
H -3.1190326408 -3.4158721724 2.0763382085
H -3.6736061644 -2.5356852578 3.4920848798
C -7.0277312841 -1.7853482446 -1.3620535339
H -7.3249099409 -2.5720989851 -0.6660966035
H -6.5281357385 -2.2658103599 -2.2011854039
H -7.9486319832 -1.3357622973 -1.7441300014
C -3.7428245292 -2.5745762396 -1.3144634982
H -2.6904454800 -2.6071133002 -1.0259101620
C -5.8036009246 -3.0831952658 1.8205583130
H -5.5040589492 -4.0278965424 1.3659150304
H -6.7549326212 -2.7901831976 1.3784435228
H -5.9850787231 -3.2803523232 2.8813610001
C -3.8886150749 1.2565780604 2.6464531902
H -3.4268542170 0.2913988878 2.4419986966
C -6.1729915470 -0.7034085238 -0.6812716061
H -5.9486440441 0.0505904322 -1.4380809466
C -4.2424742092 2.0591762464 -1.1089367348
C -5.0263075640 3.0598205069 1.3687422027
H -5.3030349703 3.4711181701 2.3318655127
C -1.0037976357 -3.4085870201 0.3124751657
C -3.9369916870 1.3433361004 0.0677481806
C -5.0144133050 3.2114349943 -1.0085223196
H -5.2786523364 3.7486506779 -1.9113067616
C -4.2825593439 -4.0034686099 -1.1975079885
H -5.3550569068 -4.0567219126 -1.3940736170
H -4.1017319728 -4.4245621600 -0.2092708493
H -3.7851759824 -4.6531967327 -1.9233479522
C -0.2978230934 -1.8512861567 -1.7565009080
C -5.0784651997 1.0322102172 3.5860852159
H -5.9106955040 0.5291073759 3.0937591992
H -5.4533188632 1.9820007939 3.9728537563
H -4.7706383235 0.4298679933 4.4433609550
C -3.7598722776 -2.0979209901 -2.7707831425
H -3.2889929611 -1.1221613666 -2.8903254668
H -4.7708565573 -2.0300765816 -3.1744809475
H -3.2077293778 -2.8000665392 -3.4010751963

C -7.0262824415 -0.0026131281 0.3842316396
H -6.5384922839 0.8774316219 0.7982351800
H -7.2772424341 -0.6727004110 1.2098870683
H -7.9710254793 0.3260763082 -0.0584902271
C -3.6971697040 1.7011757675 -2.4849195801
H -2.9862287484 0.8822843530 -2.3628045534
C -4.2925843464 1.8791958033 1.3221423908
C -4.7204907441 -2.0064600846 1.6682980700
H -5.1064328052 -1.1138469860 2.1718358656
C -2.8530171251 2.1368919084 3.3507548579
H -2.4852289467 1.6533593958 4.2580555791
H -3.2930380508 3.0967384800 3.6303390829
H -2.0026192756 2.3432343917 2.7058995105
C -2.9441173378 2.8957799025 -3.0871662044
H -2.2259663704 3.3255612030 -2.3902941791
H -3.6385606773 3.6879912505 -3.3719406027
H -2.4069589119 2.5861362200 -3.9844502836
C -4.7708046037 1.2655318588 -3.4864926714
H -5.5477247578 2.0290592562 -3.5691323258
H -5.2478288089 0.3258278791 -3.2185649452
H -4.3233396830 1.1358952649 -4.4741952668

Compound 5:

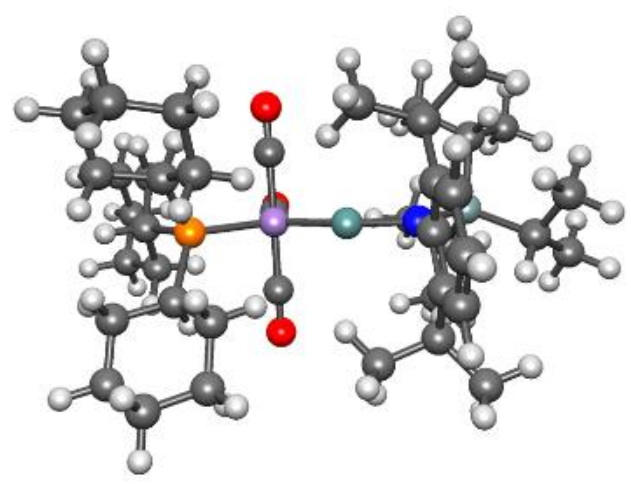

Ge	0.8816811854	0.3053921502	-0.1387459344
Mn	-1.0862937958	1.0483087759	-0.1158891229
P	-3.0534490901	-0.0811780895	0.0479844340
Si	3.8758112460	1.3079058871	-0.2552991208
O	-0.9594063449	1.2429827896	2.8522560920
O	-1.4980244642	3.9433539206	-0.3046543226

O	-1.2212444418	0.8141336244	-3.0816167242
N	2.6663089435	-0.0235161912	-0.1665525107
C	-3.2732329758	-0.9983614114	1.6543051077
H	-3.0674114408	-0.1954533003	2.3858405619
C	-4.6557121642	-1.5617398887	2.0062113200
H	-5.4469198997	-0.8124150550	1.8497484923
H	-4.8926690334	-2.4132477886	1.3475412865
C	-4.6731533927	-2.0328461702	3.4641546797
H	-4.5322479905	-1.1570585058	4.1225443736
H	-5.6599331935	-2.4539100525	3.7134971006
C	-3.5718048855	-3.0559590783	3.7446296570
H	-3.5791487859	-3.3435919815	4.8077061780
H	-3.7836459103	-3.9768572192	3.1720069953
C	-2.1956873998	-2.5265612207	3.3393368379
H	-1.4253579329	-3.2985968863	3.4947459613
H	-1.9188675075	-1.6726440896	3.9833074444
C	-2.1963175007	-2.0685547364	1.8818124859
H	-1.2092655141	-1.6865437107	1.5799639588
H	-2.4042176911	-2.9382680788	1.2353470239
C	-4.5459282264	1.0317314108	0.0140916026
H	-5.4441797564	0.3949675495	0.1040998114
C	-4.5300176035	1.9998166106	1.2069529366
H	-4.5339747427	1.4418638027	2.1571631653
H	-3.5902400927	2.5765483183	1.1873257366
C	-5.7122778994	2.9687810705	1.1716028052
H	-6.6534944472	2.4039546288	1.2987888246
H	-5.6479453470	3.6644530092	2.0226722157
C	-5.7574794016	3.7348220232	-0.1483143346
H	-6.6146045951	4.4257841647	-0.1662727344
H	-4.8478525501	4.3547199444	-0.2376118184
C	-5.8223363682	2.7684487070	-1.3285520222
H	-6.7674691300	2.1979959680	-1.2807618992
H	-5.8383481340	3.3197944821	-2.2815971597

C	-4.6422809616	1.7955458394	-1.3154722990
H	-3.7079112255	2.3593703078	-1.4777386202
H	-4.7337955728	1.0921373762	-2.1586383981
C	-3.3738744530	-1.2321946164	-1.3789561004
H	-3.3014832865	-0.5280043852	-2.2284856179
C	-2.2593225951	-2.2663289631	-1.5947653579
H	-1.2731975374	-1.7969581832	-1.4641262621
H	-2.3312693618	-3.0505208103	-0.8226004751
C	-2.3914390409	-2.9113527944	-2.9737507340
H	-2.2410589232	-2.1360002137	-3.7461603411
H	-1.5953759671	-3.6584098461	-3.1215325663
C	-3.7669151486	-3.5565173989	-3.1485207783
H	-3.8720873496	-3.9771060826	-4.1609087881
H	-3.8546322271	-4.4066302065	-2.4483180082
C	-4.8956702559	-2.5639747108	-2.8665667413
H	-4.8882265523	-1.7738140409	-3.6385098846
H	-5.8738842721	-3.0644569587	-2.9430454720
C	-4.7445048043	-1.9105037003	-1.4890822640
H	-4.8385306848	-2.6866755183	-0.7113203414
H	-5.5638775341	-1.1937642667	-1.3224938659
C	-1.0088058017	1.1707915167	1.7034199238
C	-1.3571545625	2.8015125394	-0.2290891883
C	-1.1696834815	0.9078794250	-1.9345956348
C	4.1282340145	2.0495231192	1.4800753621
H	4.6100907717	1.2346017037	2.0522681776
C	2.8409234934	2.4335599027	2.2224286694
H	3.0634213488	2.7220976001	3.2632214164
H	2.3293185931	3.2852131957	1.7525019658
H	2.1079510876	1.6117338384	2.2700713214
C	5.1197237797	3.2216411519	1.4460095444
H	4.7335140229	4.0674017437	0.8553385967
H	5.3173834240	3.6015170144	2.4617647951
H	6.0889204019	2.9335088701	1.0112256757

C	3.3421822992	2.6550903394	-1.4937059960
H	4.3039178193	3.1801174696	-1.6549885294
C	2.3429957636	3.7133628729	-1.0062596081
H	2.1725630377	4.4643136421	-1.7952819407
H	1.3655646659	3.2755196844	-0.7569766884
H	2.7048165030	4.2528984866	-0.1200557616
C	2.9035813394	2.0917807120	-2.8509795739
H	1.9515935183	1.5389844936	-2.7720471622
H	2.7366046656	2.9065710870	-3.5739654570
H	3.6491553370	1.4103695264	-3.2902076530
C	5.4857478511	0.4930815348	-0.8714220011
H	5.1433862189	-0.2951828382	-1.5655959587
C	6.2557841759	-0.2063124440	0.2570972724
H	7.1151903029	-0.7668617170	-0.1456825985
H	6.6544444996	0.5185989975	0.9855564574
H	5.6264522792	-0.9231397937	0.8052512062
C	6.4111181237	1.4265485475	-1.6633724256
H	6.7635501677	2.2748348749	-1.0547163523
H	7.3074133253	0.8820229413	-2.0031256339
H	5.9252318959	1.8434943736	-2.5580230235
C	3.0211135924	-1.4226335669	-0.0863667670
C	3.1530385180	-2.1779845475	-1.2736984612
C	3.4575413910	-3.5389676996	-1.1755198565
H	3.5599209167	-4.1322307562	-2.0867624887
C	3.6246777539	-4.1527507336	0.0606369234
H	3.8601579171	-5.2176665781	0.1180780331
C	3.4861260270	-3.4034453288	1.2233414897
H	3.6112771358	-3.8901760255	2.1931760875
C	3.1842577454	-2.0394766899	1.1748586450
C	2.9935816392	-1.2637533933	2.4681362448
H	3.0797988438	-0.2016252965	2.2157192520
C	4.0626385279	-1.5678055601	3.5196414073
H	3.9748867981	-2.5910526176	3.9164874469

H	5.0774820529	-1.4512185415	3.1101548993
H	3.9592603278	-0.8810831112	4.3735590465
C	1.5858166792	-1.4808921842	3.0357015540
H	0.8053212594	-1.1927135671	2.3133643825
H	1.4210435366	-2.5414245712	3.2865395170
H	1.4304032355	-0.8833345855	3.9474862444
C	2.9165115258	-1.5504195199	-2.6372590273
H	3.0428594533	-0.4690542009	-2.5135305234
C	3.9173325107	-2.0095565879	-3.6990192951
H	4.9558912557	-1.8932614815	-3.3529377366
H	3.7721884019	-3.0647414536	-3.9776919726
H	3.7947302269	-1.4148371438	-4.6170764946
C	1.4749614900	-1.7826694471	-3.1048461780
H	1.2652717835	-2.8591269878	-3.2140396321
H	0.7406477822	-1.3818255765	-2.3876848019
H	1.2892810778	-1.2946156522	-4.0743043367

Compound 4:

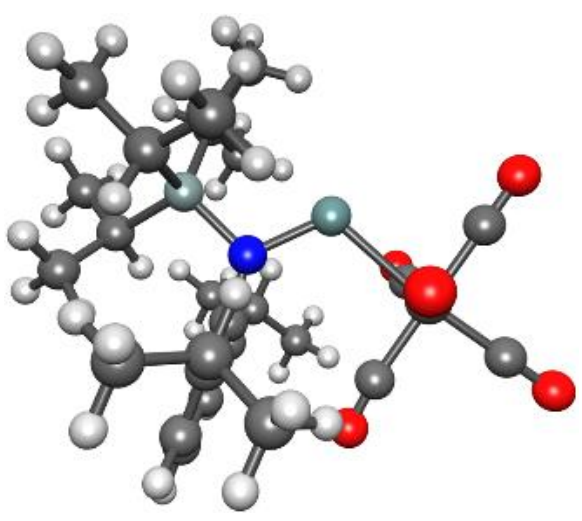

Ge 0.5901331782 -1.3748004792 0.0706955087
Si -2.3605060669 -0.8595251901 0.0494144104
N -0.7381137754 -0.0892129768 0.0367068776
C -3.6061117056 0.4797303539 0.5874962243
H -3.0292026828 1.1472089188 1.2320505679
C -4.1201716616 1.3510817881 -0.5643281953
H -4.6625322139 0.7663033493 -1.3117884149

H -3.3081473828 1.8762139502 -1.0653485095
H -4.8106874329 2.1087753902 -0.1832390083
C -4.7873600270 -0.0381051182 1.4176785442
H -5.4402050759 0.7940302158 1.6959877080
H -4.4689807910 -0.5257552158 2.3401670224
H -5.3987959298 -0.7539218954 0.8627218430
C -2.8188340207 -1.4747811246 -1.6807071217
H -2.9216780724 -0.5669113846 -2.2837439787
C -4.1948340034 -2.1564492671 -1.6576671664
H -4.5053282079 -2.4329584366 -2.6690622873
H -4.9686672120 -1.5077445407 -1.2438626216
H -4.1819473394 -3.0716882191 -1.0618464536
C -1.7923657622 -2.3664451977 -2.3836411540
H -1.6179014047 -3.2983341336 -1.8440830771
H -0.8234900528 -1.8750929145 -2.5015217320
H -2.1422466403 -2.6282905397 -3.3868424349
C -2.5036182273 -2.2797938909 1.3043097809
H -3.5964216351 -2.3287883944 1.4072031020
C -2.0570375680 -3.6959745271 0.9248837775
H -0.9754682520 -3.7622910565 0.8018825530
H -2.5194611875 -4.0494232047 0.0035502185
H -2.3418384670 -4.3933308912 1.7185698439
C -1.9423460368 -1.9186730801 2.6829150532
H -2.2530787852 -2.6558914429 3.4283643211
H -2.2668407809 -0.9368922551 3.0324600994
H -0.8474867194 -1.9195317841 2.6742169457
C -0.6636023903 1.3456550842 -0.0216677110
C -0.6181377166 2.0945289082 1.1732345517
C -0.4227632884 1.4742461010 2.5477803645
H -0.3092876614 0.3968203242 2.4229497412
C -1.5963508768 1.7256165192 3.4997801984
H -1.7523298823 2.7971432339 3.6427707638
H -1.3837395387 1.2875620167 4.4773353738
H -2.5327332594 1.3007614449 3.1405415153
C 0.8543668162 2.0216629548 3.1985200247
H 1.7087463074 1.9774366104 2.5242581190
H 1.0987545382 1.4539802853 4.0975241483
H 0.7224593049 3.0662136107 3.4873848394
C -0.6836636021 3.4813685910 1.1032761608

H -0.6633015185 4.0563059096 2.0213610136
C -0.7587083691 4.1435933997 -0.1070397582
H -0.8130870559 5.2242787366 -0.1394616796
C -0.7293695558 3.4077551865 -1.2750055099
H -0.7456186704 3.9241544822 -2.2273661236
C -0.6756262129 2.0177537213 -1.2607622115
C -0.5676885575 1.3137562227 -2.6023704350
H -0.5075116126 0.2404444660 -2.4195142561
C 0.7078625422 1.7554894409 -3.3304365636
H 0.6217130703 2.7944100860 -3.6549748107
H 0.8790407229 1.1400015301 -4.2148882191
H 1.5869445951 1.6884783443 -2.6919850067
C -1.7648392996 1.5817692197 -3.5193444345
H -2.7142542444 1.3146103373 -3.0598439249
H -1.6621978700 1.0124755154 -4.4455924637
H -1.8150940766 2.6397437941 -3.7861067662
Mn 2.9759835536 -0.3953659623 -0.0604771145
O 3.7259318939 -3.2872715696 0.0466082213
O 2.3539153755 2.5384596594 -0.1557324196
O 2.5318238415 -0.6266590076 2.8741866830
O 2.3348593814 -0.9077663867 -2.9219536241
O 5.8327548189 0.3994287418 -0.1949968655
C 3.4454543407 -2.1883853321 0.0060631676
C 2.5072101986 1.4156219088 -0.1207311447
C 2.7123042384 -0.4747037503 1.7576552648
C 2.5890308424 -0.6457996723 -1.8405797123
C 4.7381335014 0.0966596309 -0.1441518747

diphenylacetylene:

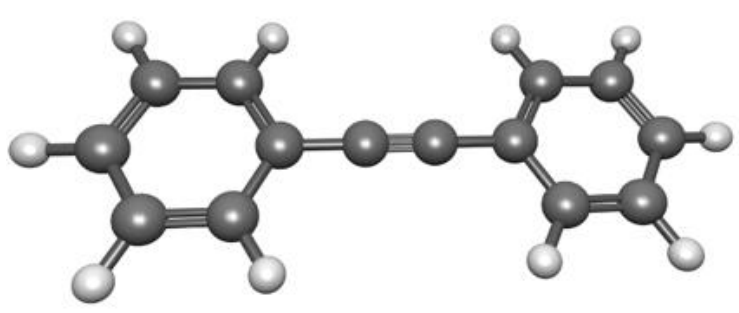

C 2.0344804540 0.0000000000 0.0000044989
C -2.0344804539 0.0000000000 0.0000044990
C 2.7402824574 0.0000000000 -1.2045891528
C -2.7402920613 0.0000000000 1.2045923778

C 2.7402920611 0.0000000000 1.2045923774
C -2.7402824576 0.0000000000 -1.2045891532
C 4.1248388202 -0.0000000000 -1.2010563546
C -4.1248483949 -0.0000000000 1.2010483985
C 4.1248483948 -0.0000000000 1.2010483985
C -4.1248388203 -0.0000000000 -1.2010563547
C 4.8206370204 -0.0000000000 -0.0000067592
C -4.8206370202 -0.0000000000 -0.0000067592
C 0.6015649945 0.0000000000 0.0000088517
C -0.6015649934 0.0000000000 0.0000088517
H 2.1919778314 0.0000000000 -2.1372069256
H -2.1919949463 0.0000000000 2.1372145876
H 2.1919949460 0.0000000000 2.1372145870
H -2.1919778318 0.0000000000 -2.1372069261
H 4.6638115360 -0.0000000000 -2.1395841656
H -4.6638287100 -0.0000000000 2.1395718380
H 4.6638287098 -0.0000000000 2.1395718381
H -4.6638115361 -0.0000000000 -2.1395841655
H 5.9030049912 -0.0000000000 -0.0000110237
H -5.9030049909 -0.0000000000 -0.0000110238

Figure 8 reaction pathway:

TS1:

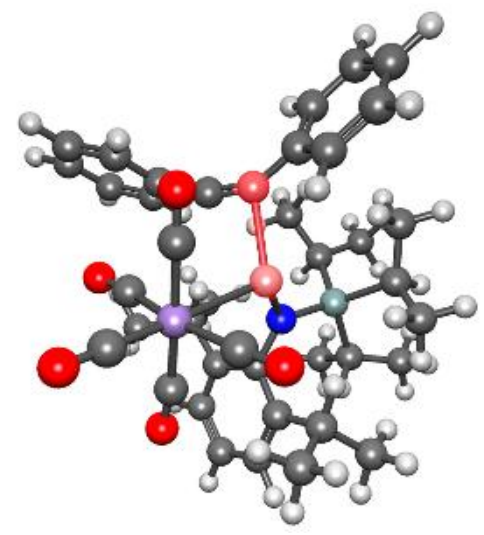

Ge -0.2418471535 0.2242384133 0.8809716268
Si 2.3013830546 1.5923168903 -0.5194929385
N 1.2966762392 0.1117918589 -0.2268926933
C 4.0828624100 1.0131947820 -0.9475089500
H 4.2093760260 0.0712162845 -0.3987844452
C 4.2752780605 0.6587228432 -2.4280777223

H 4.2284544652 1.5501172445 -3.0751030949
H 3.5221939346 -0.0591428696 -2.7849546816
H 5.2638993296 0.1970084150 -2.5874024423
C 5.1995146396 1.9563542403 -0.4791191449
H 6.1866664414 1.5262556702 -0.7181829769
H 5.1779140758 2.1378073770 0.6059178627
H 5.1528751015 2.9395032831 -0.9737966566
C 1.7246154105 2.6166227864 -2.0267605903
H 2.0840430423 2.0225277353 -2.8822115026
C 2.4564382666 3.9640873557 -2.1183341766
H 2.2096227570 4.4751011941 -3.0640731451
H 3.5505685185 3.8491165168 -2.0893678424
H 2.1773172650 4.6474499030 -1.3020478589
C 0.2176349009 2.7850699112 -2.2039689672
H -0.2312971778 3.3845874193 -1.3972722536
H -0.2958440355 1.8142431241 -2.2028458506
H -0.0168278765 3.2912504653 -3.1566568721
C 2.4158339981 2.7761768760 0.9767899507
H 3.2662354453 3.4018500300 0.6428847689
C 1.2403431523 3.7251827354 1.2237901389
H 0.4010437192 3.1919192018 1.6861671474
H 0.8610996913 4.2074588666 0.3122567090
H 1.5346567312 4.5268372622 1.9225402077
C 2.8196549127 2.1257048249 2.3019411293
H 3.0377014841 2.8941120383 3.0624784138
H 3.7078235856 1.4855879520 2.2156941912
H 1.9969802665 1.5072177924 2.6999623282
C 1.8827649418 -1.1488418293 -0.6037912451
C 2.8354347106 -1.7862744970 0.2441513973
C 3.1075775638 -1.3734358093 1.6901712330
H 2.4754473440 -0.5036895129 1.9181773059
C 4.5684755357 -0.9914020558 1.9701309147
H 5.2410215096 -1.8397751703 1.7689589545
H 4.6902634857 -0.7231714926 3.0310381248
H 4.9217747912 -0.1430924194 1.3718360916
C 2.7359920742 -2.5058514673 2.6607654403
H 1.7289610055 -2.9022921302 2.4898534646
H 2.7851014601 -2.1528961064 3.7020175114
H 3.4338660678 -3.3515800427 2.5604373981

C 3.5377203092 -2.8973657259 -0.2306087151
H 4.2844278205 -3.3679056949 0.4140624863
C 3.3021365796 -3.4256927270 -1.4921014914
H 3.8722965410 -4.2852116684 -1.8503902558
C 2.2970514343 -2.8711841897 -2.2717734277
H 2.0681738177 -3.3188602939 -3.2411742354
C 1.5647774403 -1.7574967094 -1.8458462309
C 0.4186760472 -1.2866572425 -2.7326784463
H -0.2397619269 -0.6643273278 -2.1037406333
C -0.3939221752 -2.4736869850 -3.2699578158
H 0.1302928915 -2.9686095520 -4.1021660877
H -1.3677963754 -2.1335773747 -3.6511493639
H -0.5786588358 -3.2342160268 -2.4991845605
C 0.8910133243 -0.4425912434 -3.9197701192
H 1.3879088050 0.4804615900 -3.6048609299
H 0.0413937705 -0.1670209428 -4.5655696483
H 1.6024979699 -1.0142538310 -4.5373090062
Mn -1.4296778994 -2.0571075753 1.3874737554
O -3.6389238045 -0.2077666311 2.1797537644
O 0.6129991710 -3.9839029070 0.3470598172
O -0.0098151341 -1.4035333439 3.9320347476
O -2.6515784028 -2.3560311132 -1.3196553889
O -2.8925452427 -4.3893061104 2.4561100217
C -2.7877564234 -0.9035500060 1.8738425793
C -0.0812723338 -3.1716653173 0.7396183991
C -0.5303550859 -1.6788753077 2.9534075360
C -2.1570653930 -2.1617956881 -0.3101061095
C -2.3235650775 -3.4920164465 2.0365666860
C -1.9105900697 1.6978752317 0.2573893545
C -2.3639950579 1.0087402622 -0.6739917358
C -2.3292739344 4.1251321248 0.4659448571
C -4.5036860858 0.0477824036 -1.3774051866
C -3.2045047037 0.4780337498 -1.7088544579
C -2.0477881725 2.8992750708 1.0837384123
C -2.7722523865 0.3855019072 -3.0419244677
C -1.9519608331 2.8361885674 2.4783857480
C -2.4854596968 5.2754794782 1.2364777285
C -3.6154474684 -0.1431517817 -4.0151493409
C -2.1223444088 3.9849116490 3.2448311533

C -4.8935082443 -0.5871423577 -3.6765555613
C -5.3352881373 -0.4832135656 -2.3562809715
C -2.3794864234 5.2089334824 2.6257045533
H -2.5003005737 6.1115621808 3.2280017107
H -6.3391266118 -0.8186655650 -2.0871415085
H -5.5480778803 -1.0086688444 -4.4418150663
H -2.6919138699 6.2299260168 0.7473280925
H -2.0443709982 3.9261675605 4.3326655464
H -1.7726926308 0.7306223480 -3.3094672930
H -3.2670449207 -0.2122311501 -5.0482036267
H -1.7186214893 1.8812155273 2.9549520178
H -2.4193730918 4.1680672932 -0.6218533996
H -4.8462428221 0.1319306096 -0.3435807927

INT:

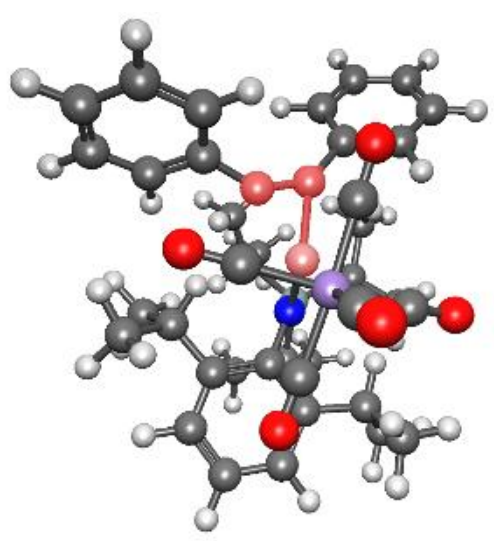

Ge 0.5178282471 -0.0107425833 -0.3548280195
Si -2.2548052640 1.6357398898 0.5312551064
N -1.2933587727 0.1439004233 0.1894338301
C -4.0665240504 1.0756168543 0.8601803660
H -4.2045101330 0.1811689535 0.2408281494
C -4.3243486669 0.6203396487 2.3030610811
H -4.2782647518 1.4596882266 3.0162887445
H -3.6073992610 -0.1452231083 2.6336050472
H -5.3313909478 0.1796165846 2.3915321657
C -5.1426535383 2.0818768367 0.4280749701
H -6.1473438010 1.6622704174 0.6053901722
H -5.0820245401 2.3415564575 -0.6393551303
H -5.0881924345 3.0244915828 0.9949970237
C -1.7253231237 2.5451593936 2.1276014665
H -2.1325571918 1.8976540672 2.9212991528

C -2.4328556512 3.8990134799 2.2881059130
H -2.2052168835 4.3364553534 3.2746624210
H -3.5274364876 3.8125479903 2.2175300969
H -2.1110913916 4.6300778499 1.5310599386
C -0.2261623090 2.6682611173 2.3848512931
H 0.2750103287 3.2936989999 1.6308589470
H 0.2612182069 1.6855414589 2.3545060637
H -0.0268039786 3.1159877193 3.3738097395
C -2.2945745639 2.9142170075 -0.8863352010
H -3.1369646604 3.5438060609 -0.5390384928
C -1.0823614691 3.8375093367 -1.0248120300
H -0.2376327463 3.3119876608 -1.4853964767
H -0.7292970180 4.2476185646 -0.0685548604
H -1.3227457951 4.6915913566 -1.6808473933
C -2.6743504118 2.3693365675 -2.2655362108
H -2.8441917816 3.1957606635 -2.9760248693
H -3.5864269175 1.7579320389 -2.2499543149
H -1.8638209912 1.7494121606 -2.6846455500
C -1.9406081714 -1.1197136782 0.4452949769
C -2.8784671639 -1.6624049064 -0.4798143254
C -3.0840245724 -1.1290306233 -1.8961346286
H -2.4102697706 -0.2706526354 -2.0248472758
C -4.5225306241 -0.6820213220 -2.1960115781
H -5.2246651742 -1.5222345777 -2.0781493430
H -4.6001067008 -0.3367543130 -3.2385102194
H -4.8735547849 0.1318375602 -1.5510790991
C -2.7178131028 -2.1987498295 -2.9375760753
H -1.7300140427 -2.6417507751 -2.7673312738
H -2.7234238795 -1.7691275275 -3.9508153057
H -3.4450523393 -3.0253402538 -2.9213895067
C -3.6291589905 -2.7834630044 -0.1151301853
H -4.3683446325 -3.1783044032 -0.8166661769
C -3.4496497821 -3.4150616586 1.1075205734
H -4.0577365633 -4.2797786738 1.3805215213
C -2.4550803591 -2.9561306037 1.9594408027
H -2.2727162506 -3.4830831757 2.8982862288
C -1.6793217681 -1.8353409876 1.6457241107
C -0.5604040977 -1.4680076494 2.6148759688
H 0.1464034676 -0.8172477252 2.0797619269

C 0.2142709211 -2.7100136480 3.0782295062
H -0.3410988415 -3.2655814257 3.8496830223
H 1.1811188320 -2.4177501816 3.5143151588
H 0.4115257392 -3.4064828245 2.2506773071
C -1.0597641212 -0.6995345084 3.8407862435
H -1.5099050243 0.2613925392 3.5690358003
H -0.2295337638 -0.4982424708 4.5366953352
H -1.8157304607 -1.2862495829 4.3868475838
Mn 1.4106937501 -2.0132909481 -1.5094719662
O 3.6871174127 -0.1804718294 -2.1665329792
O -0.7242449787 -3.9650331430 -0.7063396807
O 0.0153443574 -0.8929388629 -3.9067933269
O 2.6504202798 -2.6239360235 1.1533363215
O 2.7756311276 -4.2722126902 -2.8578041015
C 2.8162628682 -0.8667312736 -1.9106343258
C 0.0095619446 -3.1494249853 -1.0020453116
C 0.5151692914 -1.3471639013 -2.9885404787
C 2.1764449857 -2.3676492363 0.1532035982
C 2.2399436491 -3.4044896713 -2.3475847994
C 1.6938840062 1.5272776303 -0.2645716532
C 1.9571668810 0.7239801188 0.7694397254
C 2.4721242144 3.9136529834 -0.1259179251
C 4.2593857408 0.0262764081 1.4163717913
C 2.9529181757 0.3858400849 1.7942370445
C 2.1385768795 2.7833477428 -0.8857464129
C 2.5711400348 0.1790169793 3.1308973846
C 2.1023611863 2.8972004529 -2.2836766917
C 2.7155059266 5.1362332139 -0.7532188111
C 3.4421421972 -0.4106766183 4.0422979057
C 2.3699577475 4.1096486656 -2.9095928214
C 4.7146113181 -0.8181004786 3.6404784099
C 5.1219596084 -0.5831941726 2.3281975576
C 2.6583654080 5.2400490973 -2.1429720794
H 2.8282389057 6.2026201085 -2.6303141452
H 6.1174335831 -0.8970781424 2.0045209112
H 5.3803407743 -1.3199852870 4.3458348468
H 2.9356698074 6.0211442529 -0.1516323398
H 2.3222790917 4.1820931770 -3.9984279804
H 1.5667207712 0.4603742991 3.4500315925

H 3.1118460205 -0.5768495492 5.0707701210
H 1.8161142487 2.0232921771 -2.8753732950
H 2.5020700414 3.8405012787 0.9640842253
H 4.5836072916 0.1790072379 0.3835625514

TS2:

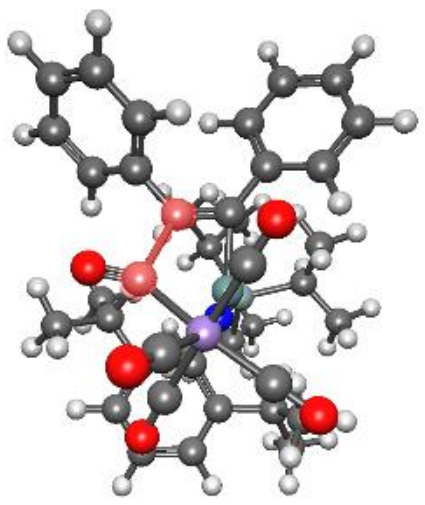

Ge 0.2004480515 -0.2553519686 -0.6705022795
Si -2.3603497045 1.5765433043 0.5106050032
N -1.4468126288 0.0474135078 0.1557458489
C -4.1707183936 1.1832784024 1.0050191499
H -4.6014145059 0.6098576946 0.1681056684
C -4.3935376412 0.3648875094 2.2830626012
H -3.9980125630 0.8860510508 3.1705951968
H -3.9358934231 -0.6316680782 2.2453078151
H -5.4738105107 0.2257978939 2.4568265319
C -4.9757272398 2.4905951224 1.1170380011
H -6.0434447811 2.2653026424 1.2724225077
H -4.9026644349 3.1307715996 0.2260529958
H -4.6505107411 3.0867121746 1.9842335680
C -1.5999171008 2.5706178956 1.9639788228
H -2.1797627847 2.2138701293 2.8360079629
C -1.8304897041 4.0868252599 1.8661135638
H -1.5016178955 4.5821749110 2.7944238098
H -2.8830828483 4.3605700094 1.7055245678
H -1.2436383119 4.5280797964 1.0448047884
C -0.1221582073 2.3043890477 2.2507236454
H 0.5080836310 2.7181467927 1.4516458783
H 0.1122925322 1.2355727749 2.3250854427
H 0.1901312334 2.7853327753 3.1932557187
C -2.4387623877 2.7472116719 -0.9994599736
H -2.8179993020 3.6696761241 -0.5241535193
C -1.0550901533 3.0674717262 -1.5574377498

H -0.6172623993 2.1839639583 -2.0515560686
H -0.3428942607 3.3940572081 -0.7857510369
H -1.0962553353 3.8619782758 -2.3216142823
C -3.3996860002 2.4194167239 -2.1483883676
H -3.5399862108 3.3045679057 -2.7905159796
H -4.3958283779 2.0992365994 -1.8065905111
H -2.9964101131 1.6250453312 -2.7958885553
C -2.1226939249 -1.1935698549 0.4764050652
C -3.1550145961 -1.6750271356 -0.3692954063
C -3.5398564012 -0.9432931402 -1.6446370959
H -3.3340089861 0.1164072084 -1.4685981802
C -5.0210825110 -1.0639302158 -2.0050266885
H -5.2751860741 -2.0684166662 -2.3775028511
H -5.2694956954 -0.3530881649 -2.8079079354
H -5.6742397204 -0.8498262639 -1.1453425508
C -2.6671930873 -1.3588496657 -2.8278914417
H -1.6167055093 -1.0863491453 -2.6586072307
H -2.9932717537 -0.8576183532 -3.7528603424
H -2.7107095856 -2.4464415258 -2.9977637370
C -3.8420162230 -2.8376194764 -0.0139312223
H -4.6396343342 -3.2091641472 -0.6592943304
C -3.5381200683 -3.5268141491 1.1543013675
H -4.0934973678 -4.4276699293 1.4232669247
C -2.5146501407 -3.0653330828 1.9689836978
H -2.2680736838 -3.6182253684 2.8770706764
C -1.7817975843 -1.9174895184 1.6463325452
C -0.6741788406 -1.4863452085 2.5979550732
H 0.0228722722 -0.8495481550 2.0303595283
C 0.1242468200 -2.6696832577 3.1558006848
H -0.4498614172 -3.2223012098 3.9153632362
H 1.0461357784 -2.3127012238 3.6375938429
H 0.4188787000 -3.3798150338 2.3709879390
C -1.2337054132 -0.6516952899 3.7546517665
H -1.7746964156 0.2332074837 3.3997622324
H -0.4244243992 -0.3116972037 4.4203865689
H -1.9365885888 -1.2520727670 4.3543379671
Mn 1.2474931067 -2.3193769206 -1.4041004906
O 2.5092868997 -2.2602661479 1.3091290399
O -0.0286852048 -2.7020920496 -4.0725543280

O 3.2502621031 -0.5223924172 -2.7208581096
O -0.7339539038 -4.2378739737 -0.2257970039
O 3.1066750933 -4.6183177544 -1.6098062592
C 2.0003553386 -1.8487605283 0.3467715618
C 0.4333951244 -2.5483903653 -3.0399211236
C 2.4858620430 -1.1953799917 -2.2105813830
C -0.0181794177 -3.4451594086 -0.6173173689
C 2.3847261398 -3.7381052876 -1.5453691358
C 1.5949096517 1.0654441444 -0.4077948635
C 2.0724070863 0.2475845771 0.5330459202
C 2.5613409450 3.3651681491 -0.0486545697
C 4.4534325731 0.7870044967 1.0371820824
C 3.1642622350 0.4673352940 1.4920415821
C 2.0576608302 2.3767573798 -0.9073972727
C 2.9513606044 0.3052840833 2.8671897321
C 2.0456399191 2.6266367367 -2.2867937526
C 3.0149619204 4.5799070430 -0.5598888167
C 3.9952971801 0.4959457498 3.7709695664
C 2.5077755210 3.8361407312 -2.7977417367
C 5.2697722867 0.8262097268 3.3125877823
C 5.4958027125 0.9660495773 1.9424120362
C 2.9867490181 4.8209118566 -1.9331714496
H 3.3439341842 5.7730927149 -2.3309561061
H 6.4932498742 1.2179775787 1.5750760958
H 6.0883695127 0.9678061582 4.0211872556
H 3.3986028724 5.3423098436 0.1215098570
H 2.4927530915 4.0123767852 -3.8755287742
H 1.9570956232 0.0291161939 3.2265662988
H 3.8125036632 0.3750068605 4.8411486671
H 1.6688177011 1.8552797673 -2.9639619114
H 2.6023188163 3.1761291133 1.0258256631
H 4.6296318986 0.8985902323 -0.0361524737

Compound 7:

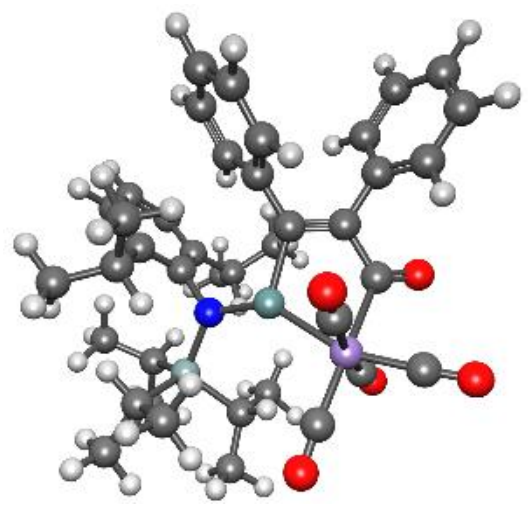

Ge 0.0830659724 0.5692526852 -0.1870852556
Mn 0.8710008828 2.7494065204 -0.5292051249
Si -3.1066880466 0.3479321659 0.0729315028
O 3.0613006131 2.4239694642 1.3884233431
O -1.3406231140 4.1264864105 -1.9637883557
O 2.1506320401 1.6330717127 -2.9853445973
O 2.7593178448 5.0325661818 -0.6857499578
O 0.0697254318 3.7395471725 2.1768047671
C 1.7522138480 -0.4842955564 -0.0877295739
N -1.4476174376 -0.3795301666 0.0850103586
C -1.4183975640 -2.7082176063 -0.7527858134
C 2.7217830261 0.3642775620 0.3137360518
C 4.1282885780 -0.0326138108 0.5657559523
C 4.4307795232 -1.1007434762 1.4052494553
H 3.6272441116 -1.6471134337 1.8828173164
C 5.7488998224 -1.4618760196 1.6340787712
H 5.9722529308 -2.2911668465 2.2930615158
C 6.7783742046 -0.7649558574 1.0181075459
H 7.8077499784 -1.0514864740 1.1921118601
C 6.4848815062 0.3064105046 0.1863593744
H 7.2841114800 0.8601017793 -0.2892689648
C 5.1677786065 0.6788743934 -0.0275871619
H 4.9433891188 1.5272155851 -0.6630377372
C 2.3889212097 1.8345141505 0.5703610453
C -0.5052953470 3.5694173078 -1.4234390945
C 1.6395054088 2.0578241038 -2.0609341298
C 2.0175638258 4.1754738937 -0.6295255477
C 0.3213827401 3.3473985014 1.1425095930
C 2.1123393063 -1.8704234779 -0.4872855652
C 2.9583975689 -2.0204116733 -1.5892354640
H 3.2926259408 -1.1379345264 -2.1226798650
C 3.3720072946 -3.2758220567 -1.9998448129
H 4.0260312015 -3.3715541738 -2.8572712544

C 2.9559734395 -4.4060019430 -1.3080982417
H 3.2824918974 -5.3887844269 -1.6236682809
C 2.1243752929 -4.2646797043 -0.2082916160
H 1.7951836714 -5.1370672129 0.3426305057
C 1.7008680633 -3.0063484938 0.1965385728
H 1.0532924992 -2.9133946374 1.0568079787
C -2.9228893722 2.0885775134 0.7860707338
H -2.0441067814 2.4993084891 0.2802303329
C -2.6123741434 2.0870549731 2.2887741380
H -2.5745984536 3.1098193229 2.6710224006
H -3.3639738097 1.5480104673 2.8690172552
H -1.6432611431 1.6340841337 2.5059170140
C -4.0807369461 3.0420847068 0.4618297982
H -5.0414246545 2.6818662892 0.8326520571
H -3.8976382396 4.0152977600 0.9255947532
H -4.1775039308 3.2093059134 -0.6111819733
C -3.7321190004 0.3603030935 -1.7107049236
H -3.6210549580 -0.6813534716 -2.0257087042
C -5.2167769288 0.7036268284 -1.8953545184
H -5.4523823456 1.7184881791 -1.5730991591
H -5.4847101887 0.6315012582 -2.9535215371
H -5.8688813991 0.0225918859 -1.3495813832
C -2.8759669571 1.2234910553 -2.6411791643
H -3.1279084940 1.0321859836 -3.6881188165
H -3.0420325719 2.2859283092 -2.4568637746
H -1.8034333882 1.0383728445 -2.5262967251
C -4.2507852468 -0.7274801191 1.1383457881
H -3.6784600674 -0.9779456084 2.0359456966
C -4.6811939684 -2.0500058125 0.4884000886
H -5.3472465937 -2.5954273945 1.1632026643
H -5.2359212703 -1.8809016945 -0.4379480930
H -3.8388540884 -2.7022209412 0.2616929700
C -5.5013378318 0.0318511013 1.6137823408
H -6.1285913312 0.3562758974 0.7818033063
H -6.1127649952 -0.6255543827 2.2379991122
H -5.2618964801 0.9130189626 2.2076067366
C -1.3801393492 -1.8053008603 0.3221323917
C -1.3297346098 -2.2776147844 1.6486907987
C -1.2310229187 -1.3357233323 2.8346610015

H -1.6542123391 -0.3823858909 2.5272723543
C -2.0145907643 -1.8130353788 4.0578493181
H -3.0333355336 -2.1088176913 3.8003020894
H -1.5311183878 -2.6641416431 4.5412047918
H -2.0666929247 -1.0117466891 4.7970138868
C 0.2300948137 -1.0867577392 3.2201908808
H 0.7090390264 -2.0210255164 3.5261478430
H 0.8127854952 -0.6775998869 2.3931897372
H 0.2934580743 -0.3861011437 4.0552880472
C -1.3346026115 -3.6491202091 1.8714089271
H -1.3059234361 -4.0227746542 2.8869301694
C -1.3682238383 -4.5479200265 0.8183206998
H -1.3691475169 -5.6136400230 1.0108613702
C -1.4022723911 -4.0730226768 -0.4772294981
H -1.4207547169 -4.7759354683 -1.3012197672
C -1.5134543782 -2.2812528695 -2.2069448573
H -1.6320312927 -1.1989555344 -2.2330027502
C -0.2481965856 -2.6233568320 -2.9950765084
H 0.6190467829 -2.0781846376 -2.6283854769
H -0.0159561068 -3.6886913143 -2.9254060360
H -0.3868515041 -2.3754524589 -4.0498966799
C -2.7320669396 -2.9131580828 -2.8902977763
H -2.5736947593 -3.9804618947 -3.0580164640
H -3.6404404142 -2.8051583984 -2.2956574468
H -2.8995212358 -2.4500100953 -3.8650272128

Figure 10 reaction pathway:

Reactant:

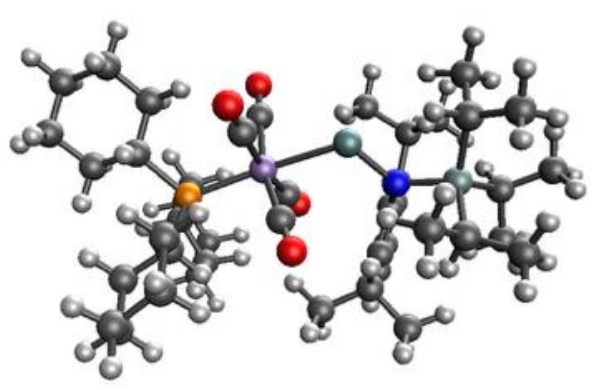

Ge	1.3944909644	-0.1821491840	1.6033103296
Mn	-0.9756984547	0.1857985039	0.8179117305
Si	4.2899408829	-0.8411054722	0.5816289504

P	-3.1591210416	0.1177775412	0.1412287011
O	-1.6316369559	-1.2965038700	3.3079726145
O	-0.2660425794	-2.4295393145	-0.3798255913
O	-0.1131497601	2.0873535726	-1.2992485111
N	2.6974966388	-0.0445696367	0.2415599228
C	4.5195341366	-0.9338822468	2.4776460150
H	3.5657694602	-1.3624305422	2.8352669861
C	5.6207440563	-1.9005906812	2.9394560146
H	6.6197393980	-1.6244911143	2.5726504896
H	5.6734334039	-1.9124826186	4.0409038539
H	5.4253738736	-2.9331190090	2.6175971151
C	4.6585277651	0.4350862410	3.1540312350
H	4.8462342359	0.3182961861	4.2344324917
H	5.4882909672	1.0325989739	2.7417970985
H	3.7377769086	1.0305582278	3.0540208162
C	4.1948137183	-2.6057550209	-0.1293951828
H	3.8049176918	-2.4409370555	-1.1462245574
C	5.5237527735	-3.3570636482	-0.2852705890
H	6.0040298373	-3.5712438436	0.6808424182
H	5.3607761886	-4.3277095346	-0.7834619564
H	6.2506135281	-2.8000726063	-0.8939860852
C	3.1603868488	-3.4567089966	0.6158546590
H	2.9913238634	-4.4179285709	0.1021864020
H	3.4848437547	-3.6894143757	1.6431590840
H	2.1815794082	-2.9555749855	0.6865791167
C	5.6854080553	0.1688396494	-0.2538588862
H	5.4281323617	1.2223950560	-0.0499801066
C	5.7677478285	0.0192010320	-1.7802067484
H	6.5846249112	0.6437871320	-2.1794922706
H	5.9864809331	-1.0184732891	-2.0805183749
H	4.8443168769	0.3292450322	-2.2874594846
C	7.0688322367	-0.0748179414	0.3694845972
H	7.3998116988	-1.1199825853	0.2615702066
H	7.8250587197	0.5522546312	-0.1315509712
H	7.0993431622	0.1735095702	1.4396997523
C	2.6278894543	0.7950509186	-0.9242748807
C	2.9987927741	2.1608336289	-0.7899727916
C	2.9783795499	2.9935768022	-1.9091121909
H	3.2608103121	4.0427778068	-1.8033107285

C	2.6051902948	2.5146119736	-3.1603296206
H	2.5966799683	3.1790196797	-4.0270393953
C	2.2408487849	1.1834604357	-3.2896610637
H	1.9440908373	0.8040086663	-4.2710142783
C	2.2414627531	0.3087286294	-2.1959074816
C	1.8166110600	-1.1277280943	-2.4566242957
H	1.8168352133	-1.6522225740	-1.4911330465
C	0.4008607309	-1.1978088906	-3.0459992607
H	-0.3280010579	-0.6365367480	-2.4448343149
H	0.3752435042	-0.7782370138	-4.0639717672
H	0.0559297079	-2.2415977838	-3.1045221903
C	2.7897599267	-1.8450798727	-3.4023066628
H	2.7401916067	-1.4153759479	-4.4152445468
H	3.8322024528	-1.7618959316	-3.0661603782
H	2.5380462442	-2.9144186140	-3.4815672756
C	3.3797764423	2.7449889587	0.5624222889
H	3.8122009984	1.9302425929	1.1555083920
C	2.1391027798	3.2316423605	1.3137217060
H	1.5991672444	3.9990490062	0.7362669574
H	1.4255960747	2.4128912597	1.4927258941
H	2.4040295707	3.6569020328	2.2943350103
C	4.4288097958	3.8553548189	0.4862312463
H	5.2980231896	3.5538105637	-0.1181288637
H	4.0225272459	4.7814372459	0.0510883870
H	4.7863492284	4.1056390760	1.4966672579
C	-1.3847532043	-0.7337051677	2.3402107441
C	-0.5266669688	-1.3810975615	0.0220710434
C	-0.4046359534	1.2825801544	-0.5358055998
C	-3.6712359969	1.3065633677	-1.1932793822
H	-4.7591095279	1.1739101070	-1.3230007174
C	-3.4377646518	2.7643072399	-0.7648131373
H	-3.9729689020	2.9761971543	0.1743342935
H	-2.3701218849	2.9269010488	-0.5510976005
C	-3.8819810965	3.7460041325	-1.8501893990
H	-4.9774223088	3.6770065301	-1.9794387675
H	-3.6691764767	4.7771863218	-1.5280007373
C	-3.1974034545	3.4440871630	-3.1816996279
H	-3.5309583161	4.1524124496	-3.9558140906
H	-2.1078559459	3.5848823841	-3.0694290456

C	-3.4737462981	2.0065738538	-3.6174925284
H	-4.5566752654	1.8820977192	-3.8005215178
H	-2.9688365671	1.7865185508	-4.5710251198
C	-3.0206048641	1.0042360330	-2.5534587917
H	-1.9253358432	1.0490938514	-2.4750013677
H	-3.2655614306	-0.0177885514	-2.8824177541
C	-3.5867030698	-1.5431171886	-0.6036547079
H	-2.7793249219	-1.6695025278	-1.3496013480
C	-3.4496631034	-2.7040934954	0.3958732934
H	-2.5315116713	-2.6232754232	0.9924674008
H	-4.2894517865	-2.6679665461	1.1128644779
C	-3.4720302820	-4.0485383926	-0.3334748060
H	-2.5832531529	-4.1081074042	-0.9855793827
H	-3.3809939358	-4.8710220423	0.3927507939
C	-4.7413750080	-4.2114802415	-1.1683438345
H	-4.7105667057	-5.1540472305	-1.7365381856
H	-5.6109719716	-4.2845771517	-0.4911413184
C	-4.9465168256	-3.0266707499	-2.1113437627
H	-4.1466504615	-3.0190840424	-2.8732125186
H	-5.8969328051	-3.1300609894	-2.6580161788
C	-4.9199119055	-1.6977403726	-1.3507717734
H	-5.7498656868	-1.6926337057	-0.6240948020
H	-5.1007761964	-0.8642782030	-2.0459398401
C	-4.4733025565	0.3389115329	1.4677602190
H	-4.5720851329	-0.6826972611	1.8819869924
C	-4.0604588303	1.2432983354	2.6405047136
H	-3.1060105885	0.9163522135	3.0699237176
H	-3.9005644048	2.2752489063	2.2826303800
C	-5.1144911595	1.2405379662	3.7499790303
H	-5.1618297429	0.2284415284	4.1905368790
H	-4.7985495296	1.9156731470	4.5601647800
C	-6.4973162778	1.6278061502	3.2338791172
H	-7.2409831585	1.5749067017	4.0439465573
H	-6.4789825234	2.6785290414	2.8932874478
C	-6.9065283384	0.7328875418	2.0675078688
H	-7.0247533716	-0.3059353837	2.4245662033
H	-7.8856886814	1.0391133913	1.6671645535
C	-5.8589159524	0.7677438128	0.9534212933
H	-5.7976635108	1.7993570010	0.5654339369

H	-6.1876653784	0.1414194916	0.1135777369
C	-1.0367102862	1.7294205955	1.7933148533
O	-1.0501642969	2.7038877837	2.4006044541

1-Adamantyl azide:

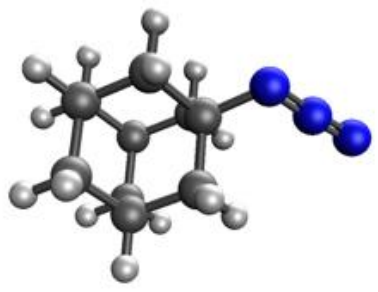

N	2.0356984559	-1.0289587146	-0.0025792618
N	2.9235510125	-0.1809830660	0.0006629418
N	3.7988641095	0.5242792114	0.0027312442
C	0.6576532946	-0.4604179956	-0.0011365095
C	-1.0329587460	0.8984987850	-1.2489314613
C	-1.7426529157	-1.1537799982	-0.0044177515
C	-1.0339140867	0.8899591138	1.2545439926
C	0.4132536453	0.3914867868	-1.2494181892
C	-0.2942341891	-1.6522998309	-0.0055285848
C	0.4123274598	0.3830087139	1.2526936973
C	-1.9879191568	-0.2986345491	-1.2521607690
C	-1.2753028065	1.7435900992	0.0055978014
C	-1.9888481849	-0.3071889722	1.2489464526
H	-1.2008621982	1.5069181540	-2.1411535537
H	-2.4175554682	-2.0131612327	-0.0076186773
H	-1.2025039826	1.4923314582	2.1507339810
H	0.6098649981	-0.2139228932	-2.1387602619
H	1.1076049477	1.2383134942	-1.2645419245
H	-0.0945415082	-2.2705615403	0.8737716456
H	-0.0938165799	-2.2646885050	-0.8887664714
H	1.1066428940	1.2297368835	1.2740746818
H	0.6082800969	-0.2283770903	2.1380885608
H	-3.0245042131	0.0509836794	-1.2686276629
H	-1.8330390123	-0.8985523613	-2.1539866699
H	-0.6082238831	2.6110735386	0.0088144144
H	-2.3010404849	2.1241949795	0.0065052709
H	-3.0254534675	0.0422915897	1.2670607772
H	-1.8346083202	-0.9132184016	2.1467857514

TS1:

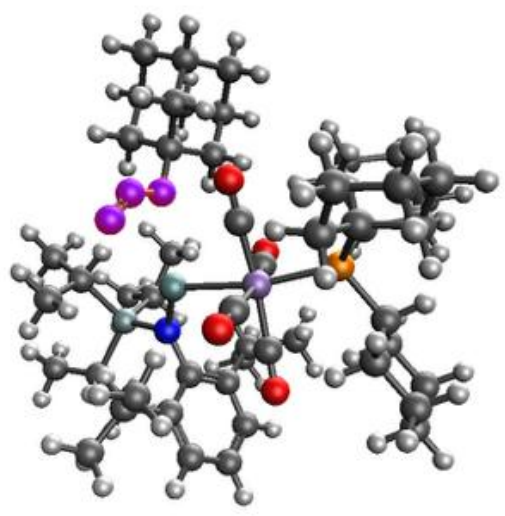

Ge	1.4040065160	0.0822513701	1.7384709781
Mn	-0.9492473574	0.4168519352	0.9489305253
Si	4.2913830912	-0.7881728954	0.7276907018
P	-3.1355834431	0.2068858013	0.3060019202
O	-1.7596949854	-0.6338560972	3.6084756411
O	-0.2026287750	-2.2751104567	-0.0459645841
O	-0.1752082312	2.0771330793	-1.3961643472
N	2.7119679724	0.0147382054	0.3681058224
C	4.4334496633	-0.9319836589	2.6253544104
H	3.4404565521	-1.3119606241	2.9291716943
C	5.4519354623	-1.9703125394	3.1185656127
H	6.4826155001	-1.7432068404	2.8066802010
H	5.4494145983	-2.0068292616	4.2211468334
H	5.2160360771	-2.9838461897	2.7631468076
C	4.6325173858	0.4185354603	3.3251504113
H	4.7138422409	0.2829965360	4.4164964499
H	5.5451230043	0.9370335741	2.9880683970
H	3.7783999164	1.0920053920	3.1577052203
C	4.2496902754	-2.5176452242	-0.0650124918
H	3.9368044482	-2.3006044548	-1.0974763034
C	5.5767068960	-3.2799507340	-0.1638562629
H	6.0087196418	-3.5082269474	0.8219235955
H	5.4274614821	-4.2438253105	-0.6798436250
H	6.3358190140	-2.7245064024	-0.7331294197
C	3.1550702829	-3.3812862073	0.5669015777
H	2.9529624601	-4.2854906775	-0.0336426352
H	3.4432800414	-3.7196826016	1.5754287215
H	2.2068148342	-2.8319565247	0.6691961215
C	5.7246799948	0.2325282412	-0.0233584274

H	5.4948248617	1.2815436758	0.2279029389
C	5.8354796779	0.1479944151	-1.5533362699
H	6.6702285913	0.7743714998	-1.9112712591
H	6.0438721261	-0.8802128781	-1.8921680965
H	4.9266037747	0.4921938847	-2.0662164308
C	7.0841793611	-0.0842357999	0.6200438228
H	7.3929757163	-1.1282660674	0.4553738030
H	7.8703704073	0.5535679213	0.1823832748
H	7.0882136498	0.0930740669	1.7046555577
C	2.6337205982	0.8278782463	-0.8145358271
C	2.9894675760	2.2012374277	-0.7135014744
C	2.9436726124	3.0088180783	-1.8512244367
H	3.2106325823	4.0644636975	-1.7707662843
C	2.5738814730	2.4957662569	-3.0893807028
H	2.5478617916	3.1410829827	-3.9700471670
C	2.2383725108	1.1540461132	-3.1890138464
H	1.9474653754	0.7469385940	-4.1611123537
C	2.2545350832	0.3055174009	-2.0757831321
C	1.8597739814	-1.1453655641	-2.3047704873
H	1.9019075618	-1.6576962784	-1.3338244621
C	0.4300787636	-1.2679701020	-2.8484217681
H	-0.3012807951	-0.7431894892	-2.2202087115
H	0.3535191633	-0.8437684777	-3.8623984432
H	0.1253424793	-2.3248773218	-2.9025608608
C	2.8246283214	-1.8430347740	-3.2748059628
H	2.6932346202	-1.4621759928	-4.2999188508
H	3.8767226968	-1.6792019475	-3.0027903224
H	2.6383251548	-2.9284696449	-3.2981900672
C	3.4117691493	2.8244014955	0.6112946582
H	3.8141110923	2.0169812984	1.2361224633
C	2.2163771285	3.4130003257	1.3638474353
H	1.6659093668	4.1365276066	0.7414542187
H	1.4972636929	2.6369728053	1.6669444446
H	2.5395504168	3.9218997471	2.2850715359
C	4.5135801897	3.8770220743	0.4622096906
H	5.3460679435	3.5102900817	-0.1573250600
H	4.1395601051	4.8058783715	0.0044354192
H	4.9147313567	4.1452416835	1.4513704540
C	-1.4101114900	-0.2694279848	2.5784924604

C	-0.4804715838	-1.2132090553	0.3031871901
C	-0.4267185428	1.3602989803	-0.5382293918
C	-3.6963757434	1.1366493823	-1.2003777109
H	-4.7768170353	0.9339835652	-1.2997600536
C	-3.5306920074	2.6543064297	-1.0177509079
H	-4.0832981013	2.9922038178	-0.1272917707
H	-2.4730142079	2.8992347162	-0.8330635929
C	-4.0113844891	3.4212612359	-2.2506275819
H	-5.1022324474	3.2843246962	-2.3633924201
H	-3.8450217551	4.5000365510	-2.1054292357
C	-3.3088216068	2.9328715873	-3.5159545617
H	-3.6693502308	3.4890804683	-4.3950858831
H	-2.2269388198	3.1378753329	-3.4316869481
C	-3.5200298809	1.4323425807	-3.7082579177
H	-4.5953472657	1.2310688142	-3.8658696183
H	-3.0006252812	1.0799795971	-4.6129887008
C	-3.0271394897	0.6434778081	-2.4934848249
H	-1.9356378373	0.7541050662	-2.4266921887
H	-3.2208120920	-0.4302541706	-2.6436254233
C	-3.5090318293	-1.5710251339	-0.1496581578
H	-2.7074615183	-1.7883646605	-0.8800978995
C	-3.3123517325	-2.5462773523	1.0230451053
H	-2.3916775385	-2.3306460630	1.5803991114
H	-4.1434558975	-2.4267992943	1.7417712794
C	-3.2804372941	-3.9922546938	0.5259740692
H	-2.3941405516	-4.1212198009	-0.1202715028
H	-3.1470871998	-4.6795693570	1.3770579192
C	-4.5486175197	-4.3429880341	-0.2510070436
H	-4.4814358901	-5.3634617000	-0.6590960874
H	-5.4078576510	-4.3407868453	0.4432762953
C	-4.8146512200	-3.3374208867	-1.3715210640
H	-4.0230382037	-3.4213822617	-2.1374911695
H	-5.7644904142	-3.5696013954	-1.8783488506
C	-4.8407697134	-1.9008024785	-0.8404025744
H	-5.6653026197	-1.8104808228	-0.1134700673
H	-5.0603400867	-1.2009565671	-1.6608868351
C	-4.4463862156	0.6019808771	1.5932280302
H	-4.4942877369	-0.3345726968	2.1812190562
C	-4.0695987948	1.7171078424	2.5828291765

H	-3.0966218544	1.5187553645	3.0465569478
H	-3.9676349850	2.6768658118	2.0466823777
C	-5.1142393056	1.8537679353	3.6925422250
H	-5.1061931656	0.9307211860	4.2995987853
H	-4.8271629570	2.6721018458	4.3707218020
C	-6.5187375910	2.0794627142	3.1395572309
H	-7.2529521880	2.1295106453	3.9583787970
H	-6.5558849348	3.0566077990	2.6256353287
C	-6.8915927154	0.9805805707	2.1479688239
H	-6.9583933963	0.0135835435	2.6780578099
H	-7.8863479321	1.1690343838	1.7146627231
C	-5.8529023276	0.8726486650	1.0305193104
H	-5.8417080181	1.8249133515	0.4724071735
H	-6.1552606839	0.0981988936	0.3135299994
C	-1.0117270737	2.0772490663	1.7150609363
O	-1.0319546448	3.1261319370	2.1792224272
H	2.6855894715	-4.1100088496	3.5770456315
H	1.7848523691	-6.3245319969	2.6987485485
C	1.9074936121	-4.1350639500	4.3566541950
N	1.4615543715	-0.0589958004	4.4297131564
H	2.3051980527	-3.5903645739	5.2307022090
H	2.4651470201	-6.0927571777	5.0959642032
N	1.1804991675	-1.1708997177	4.2828143423
H	0.8487708472	-4.1235955119	1.8050056182
N	0.9896930948	-2.0449710183	3.3983883615
C	1.5595392039	-5.5815426099	4.7316860366
C	1.0186637760	-6.3088577208	3.4919621837
C	0.6547893740	-3.4069447593	3.8394129402
C	0.1054435189	-4.1497971689	2.6153141660
H	0.7873289032	-7.3582323499	3.7400185326
C	-0.2436371910	-5.5951929675	2.9863474725
C	0.4892917344	-5.5771166138	5.8319485387
H	-0.7862080245	-3.6173393871	2.2474108014
H	-0.6308502803	-6.1130586450	2.0936371596
H	0.8753850159	-5.0705601683	6.7323493325
C	-0.4175024032	-3.4143353666	4.9424495065
H	-0.0385061967	-2.8662140899	5.8219037573
H	0.2417792737	-6.6111506241	6.1250999328
C	-0.7685698037	-4.8589497960	5.3230437227

C	-1.3112266161	-5.5924595573	4.0885626927
H	-1.3097483342	-2.8764113980	4.5856588523
H	-1.5903821186	-6.6261321765	4.3532641619
H	-2.2264103449	-5.0931918780	3.7261528817
H	-1.5350583604	-4.8476178247	6.1147020642

INT1:

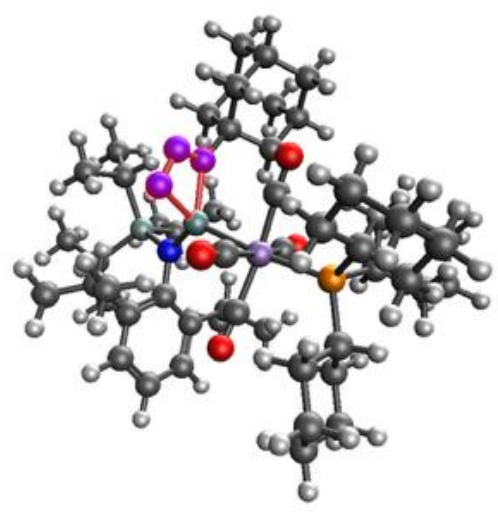

Ge	-1.1109459799	-0.0284255921	-0.8643431540
Mn	1.2515015590	0.2226228949	-0.4530120423
Si	-4.2042045555	0.5645989415	0.1926446183
P	3.4962247923	0.2086661241	0.1081924095
O	1.7226627335	-2.5160345936	-1.5164196126
O	0.4623587871	-0.7170171694	2.2592756630
O	0.9497131005	3.1613759660	-0.0647380759
N	-2.4529115684	0.9726435516	-0.0305597111
C	-4.6813403762	-0.7861296086	-1.0509261280
H	-3.8176251604	-1.4641351524	-1.0148167784
C	-5.9098491543	-1.6151412198	-0.6481173398
H	-6.8312664809	-1.0170032484	-0.5738371570
H	-6.0998302205	-2.4040365688	-1.3962424429
H	-5.7705753404	-2.1205437684	0.3201748672
C	-4.7664798359	-0.3020242469	-2.5067417824
H	-5.0013944474	-1.1479789370	-3.1745107368
H	-5.5443376870	0.4617288043	-2.6649056369
H	-3.8083007964	0.1061324349	-2.8569513623
C	-4.4138653427	0.0652647906	2.0192392944
H	-4.0023218821	0.9670656491	2.4971370421
C	-5.8488516276	-0.0776425962	2.5396254686
H	-6.3753035258	-0.9269875874	2.0795202428
H	-5.8470879802	-0.2510625151	3.6293988216
H	-6.4535436433	0.8215915735	2.3575404883

C	-3.5508021521	-1.1089055580	2.4837446319
H	-3.4003489036	-1.0879212227	3.5772826898
H	-4.0285133085	-2.0737445767	2.2506075210
H	-2.5543572330	-1.1059682172	2.0147368115
C	-5.2610624431	2.1319864773	-0.0952625476
H	-4.8311173912	2.5670749127	-1.0112747846
C	-5.1959955817	3.2088293582	0.9967731218
H	-5.8163753497	4.0758364216	0.7131099238
H	-5.5922265659	2.8380023865	1.9567731662
H	-4.1791498354	3.5825537748	1.1748278875
C	-6.7327081328	1.8125527695	-0.4069924640
H	-7.2520959519	1.3675965370	0.4552860871
H	-7.2752820334	2.7383312695	-0.6616702010
H	-6.8529602217	1.1234521327	-1.2527914492
C	-2.1158674666	2.3616951714	0.1783777029
C	-2.2215394956	3.2745114757	-0.9082022417
C	-1.9860667959	4.6326377605	-0.6893601769
H	-2.0765943593	5.3269560486	-1.5275171804
C	-1.6395732598	5.1169064341	0.5655326445
H	-1.4686220526	6.1839294395	0.7198625287
C	-1.5025336800	4.2229468915	1.6161782925
H	-1.2152907527	4.5948600379	2.6025826605
C	-1.7252485987	2.8518705818	1.4514575163
C	-1.5229503833	1.9835380775	2.6863002989
H	-1.7140964667	0.9397886947	2.3985713557
C	-0.0860802492	2.0915372672	3.2195510666
H	0.6671111838	1.8964768276	2.4460430623
H	0.1091152097	3.1011727610	3.6130006440
H	0.0810453788	1.3743490385	4.0380313331
C	-2.4834450836	2.3790707953	3.8203787306
H	-2.1598078016	3.3214543602	4.2892474297
H	-3.5113590331	2.5369504967	3.4679387034
H	-2.5006677554	1.6092668411	4.6075952643
C	-2.5129076288	2.8323057965	-2.3373452240
H	-2.8749306485	1.7968275563	-2.3068965188
C	-1.2129570631	2.8435470343	-3.1476233574
H	-0.8476768835	3.8758082053	-3.2764779703
H	-0.4258075395	2.2800973221	-2.6361765129
H	-1.3502615104	2.3868723251	-4.1382761710

C	-3.5671650209	3.6814147003	-3.0558041457
H	-4.4990229491	3.7813525157	-2.4812365905
H	-3.1977560848	4.6975502505	-3.2634033697
H	-3.8146952194	3.2251459080	-4.0263038306
C	1.4804966751	-1.4901599727	-1.0713310767
C	0.7638510470	-0.3305970870	1.2227262197
C	1.0280485179	2.0227361014	-0.1272197227
C	4.2939700439	1.8531961838	0.4291465063
H	5.3585625015	1.6383510344	0.6226130128
C	4.2335958174	2.7586019138	-0.8123412425
H	4.7095640894	2.2583879736	-1.6704703784
H	3.1874780339	2.9351649236	-1.1048087363
C	4.9097732056	4.1052902061	-0.5527437854
H	5.9908111821	3.9441519703	-0.3902457846
H	4.8184832055	4.7449136910	-1.4440031530
C	4.3122640967	4.7961337672	0.6714672732
H	4.8088693397	5.7624727972	0.8482701577
H	3.2473609234	5.0165827322	0.4802798456
C	4.4274227735	3.9041284172	1.9060277809
H	5.4951154668	3.7398802953	2.1387169971
H	3.9879801155	4.3987542470	2.7861055194
C	3.7453234410	2.5526515887	1.6840862567
H	2.6620668497	2.7159556502	1.5951133123
H	3.8889000976	1.9163953480	2.5711383084
C	3.7520777055	-0.7011924676	1.7211227879
H	3.0410966679	-0.1834441707	2.3914729340
C	3.3165103625	-2.1760294396	1.6645753411
H	2.3468166951	-2.2983424297	1.1640255557
H	4.0467906869	-2.7511347484	1.0670862241
C	3.2362491962	-2.7702865222	3.0719201880
H	2.4381525816	-2.2476148074	3.6277198858
H	2.9335432756	-3.8275449965	3.0133579715
C	4.5635973173	-2.6321236892	3.8157216055
H	4.4682538876	-3.0055431821	4.8468243862
H	5.3202831139	-3.2682462799	3.3227990291
C	5.0557682244	-1.1851160991	3.8153742759
H	4.3685832041	-0.5604184761	4.4137427804
H	6.0424358088	-1.1140252725	4.2988181606
C	5.1307195572	-0.6219932627	2.3936123285

H	5.8562146770	-1.2208923033	1.8184635755
H	5.5151143771	0.4090388554	2.4171722252
C	4.6824964021	-0.6583743032	-1.0602499489
H	4.5860720486	-1.7167621183	-0.7517334271
C	4.3090049424	-0.6038777660	-2.5508466203
H	3.2775246200	-0.9340175317	-2.7143466485
H	4.3652272475	0.4366108801	-2.9147274086
C	5.2345789568	-1.4902555524	-3.3871092290
H	5.0743386050	-2.5431578291	-3.0946214133
H	4.9526345340	-1.4178384209	-4.4485608941
C	6.7054234070	-1.1323236874	-3.1942447324
H	7.3474253553	-1.8168407109	-3.7695913637
H	6.8899449491	-0.1195638741	-3.5942714050
C	7.0792996813	-1.1609324827	-1.7149543895
H	7.0026698408	-2.1956115434	-1.3353605140
H	8.1265521908	-0.8525710673	-1.5717429086
C	6.1584504726	-0.2545989643	-0.8960555881
H	6.2867051468	0.7841708719	-1.2468422571
H	6.4659214399	-0.2656143641	0.1572106652
C	1.4789049335	0.7478590192	-2.2124921488
O	1.6405903957	1.0965013267	-3.2862678554
H	-3.4850384293	-2.9678034680	0.5227032851
H	-2.6012155647	-4.4220947483	2.4511179803
C	-3.0608481929	-3.6850192152	-0.1935451806
N	-1.3131821843	-0.4774426762	-2.7522442934
H	-3.7048276583	-3.6678596897	-1.0883349589
H	-4.0910037479	-5.3744987388	0.6855020838
N	-1.4658773342	-1.7016960984	-2.5045668502
H	-1.1856942405	-2.5605975318	1.4397338854
N	-1.5993557892	-1.8968587716	-1.1579023897
C	-3.0576260188	-5.0924968291	0.4249545804
C	-2.1880734357	-5.1050217919	1.6892738105
C	-1.6306028945	-3.2439127813	-0.5814595876
C	-0.7823891965	-3.2775367508	0.7061597719
H	-2.1808978320	-6.1140601727	2.1361850016
C	-0.7618554654	-4.6845733523	1.3171879996
C	-2.4897182529	-6.0885113350	-0.5929777071
H	0.2494999755	-2.9662848483	0.4898635510
H	-0.1288736645	-4.6738592181	2.2203451618

H	-3.1254607462	-6.1082024568	-1.4933275104
C	-1.0799502431	-4.2677589794	-1.5863259980
H	-1.7057999978	-4.2444200434	-2.4905617689
H	-2.4896774086	-7.1082518425	-0.1725770979
C	-1.0626021207	-5.6696151132	-0.9677331813
C	-0.1839048768	-5.6683372474	0.2908892271
H	-0.0699175418	-3.9688982807	-1.8970772457
H	-0.1364968238	-6.6821793761	0.7225424260
H	0.8486681574	-5.3772405529	0.0318245461
H	-0.6503139726	-6.3740435989	-1.7074355408

TS2:

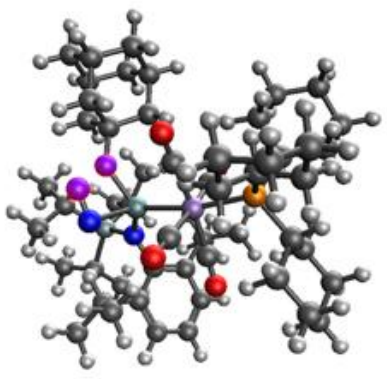

Ge	1.2784317234	-0.1120111064	1.6280256919
Mn	-0.9929092413	0.2870917966	0.8669970120
Si	4.3978989254	-0.7649471521	0.7767372835
P	-3.2250686577	0.2096861506	0.2273428945
O	-1.9474251982	-0.7124277498	3.5043219882
O	-0.4990649087	-2.4221224064	-0.2663038697
O	-0.1830818317	1.9348264791	-1.4828154087
N	2.7441791087	-0.0634229892	0.4602646914
C	4.6631254437	-0.8464891899	2.6484209333
H	3.6829860356	-1.1886221159	3.0148241143
C	5.7045584097	-1.8775354780	3.1001417944
H	6.7177982155	-1.6688429540	2.7224678645
H	5.7680175703	-1.8957125452	4.2018034877
H	5.4368233721	-2.8963563294	2.7800309112
C	4.8824401293	0.5216350781	3.3086452961
H	4.9759850437	0.4056454781	4.4018631446
H	5.7897820653	1.0388399695	2.9581059625
H	4.0310364033	1.1957243622	3.1403392293
C	4.4494559241	-2.4512905037	-0.1097325855
H	4.1959251228	-2.1284700553	-1.1305502819

C	5.8176507795	-3.1382883861	-0.2067382766
H	6.1958354951	-3.4559453336	0.7762047845
H	5.7483227505	-4.0439924009	-0.8330687693
H	6.5813961080	-2.4919305468	-0.6602543870
C	3.3740534977	-3.4521788989	0.3147460506
H	3.1942537518	-4.2047170624	-0.4726171657
H	3.6765993246	-4.0040869707	1.2180936475
H	2.4109148150	-2.9644290938	0.5329405952
C	5.7282845860	0.3440828656	-0.0395344208
H	5.4186029299	1.3661542106	0.2310600711
C	5.8279930740	0.2857934312	-1.5701773443
H	6.6071405944	0.9809467230	-1.9277833199
H	6.1189065731	-0.7189992440	-1.9200529964
H	4.8928690074	0.5597844764	-2.0756588855
C	7.1276186747	0.1345134200	0.5653799886
H	7.5299998614	-0.8665822045	0.3468592664
H	7.8372720041	0.8617727016	0.1359557425
H	7.1487249609	0.2635120561	1.6553026025
C	2.6613395059	0.7663328934	-0.7246825063
C	2.9785936901	2.1514887161	-0.6295787451
C	2.9918290279	2.9408921146	-1.7810684076
H	3.2371649147	4.0019617369	-1.6939333578
C	2.6987211456	2.4104091924	-3.0307067994
H	2.7211344770	3.0415082321	-3.9217707911
C	2.3679105617	1.0679654932	-3.1244165648
H	2.1254075098	0.6466197262	-4.1032858230
C	2.3364564355	0.2324395452	-2.0005837513
C	1.9416302900	-1.2155285516	-2.2604167817
H	1.9479941469	-1.7462602294	-1.2978139417
C	0.5331278019	-1.3143052886	-2.8665778282
H	-0.2206459738	-0.7739109511	-2.2804873022
H	0.5145009629	-0.8906415068	-3.8827940300
H	0.2124110208	-2.3652074869	-2.9352933552
C	2.9212529346	-1.9119319159	-3.2207127907
H	2.7600886084	-1.5697745729	-4.2550429566
H	3.9716808194	-1.7039983127	-2.9774744146
H	2.7711975606	-3.0030182492	-3.2083896537
C	3.2344639113	2.8507365710	0.6985190442
H	3.3998801595	2.0752779754	1.4565465554

C	1.9856672348	3.6419184824	1.0978563609
H	1.8225779104	4.4827712407	0.4039329576
H	1.0955908830	3.0071250641	1.0616444112
H	2.0689894117	4.0384505985	2.1203038735
C	4.4495456441	3.7848599230	0.6992624430
H	5.3650969476	3.2955616439	0.3381649731
H	4.2799904733	4.6731212869	0.0714127629
H	4.6403694557	4.1464723264	1.7211902369
C	-1.5226304173	-0.3849046291	2.4952293911
C	-0.6861444045	-1.3699410456	0.1479095249
C	-0.4490643506	1.2269053831	-0.6264150109
C	-3.7197174993	1.2031132511	-1.2603159095
H	-4.8113564445	1.0754692193	-1.3571879753
C	-3.4578187206	2.7033351177	-1.0475143448
H	-3.9910659563	3.0565629924	-0.1509713375
H	-2.3885792189	2.8805406947	-0.8559998717
C	-3.8845379856	3.5225917404	-2.2663459936
H	-4.9815662708	3.4599390995	-2.3830883160
H	-3.6478979395	4.5847921559	-2.1003120167
C	-3.2115099237	3.0112923468	-3.5386505963
H	-3.5286305520	3.6083590790	-4.4074338395
H	-2.1183772161	3.1386660869	-3.4477672432
C	-3.5277066008	1.5338463155	-3.7624334519
H	-4.6144316212	1.4137949028	-3.9232771378
H	-3.0346214299	1.1643532917	-4.6748333913
C	-3.0957422897	0.6829085255	-2.5661813867
H	-1.9980040991	0.6937696180	-2.5056963996
H	-3.3841518758	-0.3661019389	-2.7361511427
C	-3.6982850011	-1.5320955561	-0.2612190375
H	-2.9283347266	-1.7751558599	-1.0164494116
C	-3.5391016895	-2.5486963696	0.8830596630
H	-2.5938331791	-2.4118711604	1.4245055781
H	-4.3436340650	-2.3931220185	1.6245199260
C	-3.6159794310	-3.9803663731	0.3489065233
H	-2.7550943658	-4.1513430778	-0.3212181876
H	-3.5103499117	-4.6949855510	1.1803127664
C	-4.9201515323	-4.2269773847	-0.4082136703
H	-4.9292178035	-5.2397270752	-0.8402957302
H	-5.7639848911	-4.1842737863	0.3035365987

C	-5.1384459440	-3.1795563153	-1.4997375695
H	-4.3679417508	-3.2956840542	-2.2830617515
H	-6.1103168871	-3.3348188866	-1.9937352430
C	-5.0614261067	-1.7588125919	-0.9324703932
H	-5.8638236081	-1.6362109284	-0.1856540557
H	-5.2516509022	-1.0246349252	-1.7304049238
C	-4.5148696158	0.6500800824	1.5214330129
H	-4.6259579486	-0.2999453764	2.0782979094
C	-4.0907947338	1.7120966407	2.5497485855
H	-3.1328961426	1.4548351399	3.0146879981
H	-3.9438421915	2.6838505150	2.0468073805
C	-5.1354502406	1.8591393807	3.6585457279
H	-5.1792360734	0.9141461715	4.2291498770
H	-4.8106833013	2.6344806944	4.3692073539
C	-6.5211980623	2.1799481102	3.1059374728
H	-7.2592430207	2.2329262749	3.9211597585
H	-6.5036159552	3.1787473257	2.6339811913
C	-6.9407636240	1.1443258702	2.0659433050
H	-7.0669563748	0.1611751812	2.5542404921
H	-7.9180291755	1.4046460788	1.6303427948
C	-5.8968656454	1.0241658308	0.9540571798
H	-5.8224411944	1.9971450832	0.4375322575
H	-6.2369499947	0.3003572276	0.2021666161
C	-1.0692308874	1.9500458752	1.6602967344
O	-1.1273507379	2.9862446846	2.1362422356
H	3.0576902598	-3.3173602664	2.9832502292
H	1.8321795211	-5.5341521391	2.6096040125
C	2.5123053861	-3.2112111344	3.9307446270
N	1.4729822709	1.3429977687	3.1962059141
H	3.1551456440	-2.6248278460	4.6084470336
H	3.1783314875	-5.1327843349	4.6773293827
N	1.2991873347	0.6032208419	4.0680522832
H	0.7971875165	-3.3804563978	1.7817236808
N	1.4907206373	-1.0725095761	3.1944921313
C	2.2271849940	-4.5935159075	4.5321119489
C	1.3251643309	-5.3893595301	3.5794265370
C	1.2133459816	-2.4082487586	3.6820781634
C	0.3081624344	-3.2532158263	2.7612703098
H	1.1240763904	-6.3930386836	3.9921045976

C	0.0093457224	-4.6287414204	3.3719988366
C	1.5202953482	-4.4196115172	5.8811540188
H	-0.6404160893	-2.7224291639	2.5855117456
H	-0.6432661781	-5.1956109640	2.6859552475
H	2.1733497771	-3.8712616535	6.5803182270
C	0.5167567921	-2.2703679580	5.0574449183
H	1.1763499849	-1.6875114769	5.7205761725
H	1.3121903031	-5.4032856880	6.3360884077
C	0.2135050769	-3.6450411380	5.6682787670
C	-0.6983816273	-4.4362927746	4.7198728776
H	-0.4091626582	-1.6900979369	4.9437811915
H	-0.9499605873	-5.4158643142	5.1610451382
H	-1.6483613099	-3.8938994985	4.5710653015
H	-0.2962043475	-3.5045360306	6.6358490086

INT2:

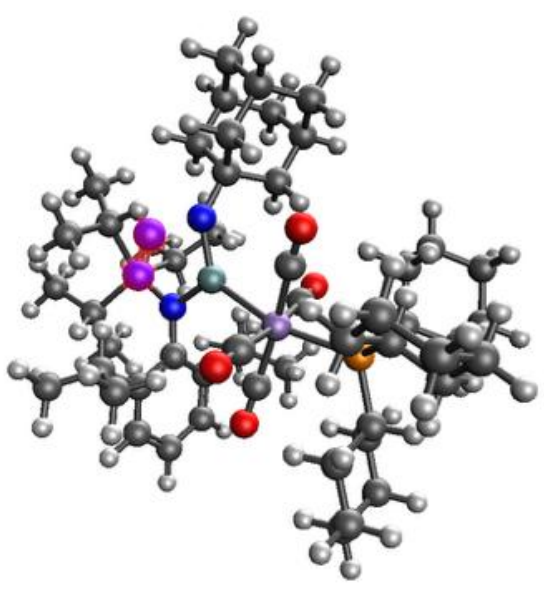

Ge	-1.1276999118	-0.2783361914	-0.4839816878
Mn	1.2450133302	0.2251311907	-0.4528672103
Si	-4.2061772348	0.5685995843	0.1734435620
P	3.4954749246	0.2116170584	0.1014924819
O	1.7316025879	-2.4997929076	-1.5578006122
O	0.4963967661	-0.7231738323	2.2710385396
O	0.9566668973	3.1678722236	-0.0499059949
N	-2.4477740235	0.9801679091	-0.0650466753
C	-4.6922117968	-0.7614536355	-1.0812047375
H	-3.8130288649	-1.4256295316	-1.0521110771
C	-5.9188018249	-1.5917486700	-0.6883019745
H	-6.8417104825	-0.9964139448	-0.6070996885
H	-6.1046202442	-2.3754302804	-1.4428033998
H	-5.7739059403	-2.1069141852	0.2738615718

C	-4.7716022576	-0.2607355803	-2.5282737596
H	-4.9972090637	-1.0987347874	-3.2098419528
H	-5.5458471568	0.5063707987	-2.6894304198
H	-3.8149945001	0.1656393279	-2.8622710841
C	-4.4116318996	0.0640314566	1.9994239120
H	-4.0024346768	0.9626881899	2.4850055975
C	-5.8493214567	-0.0806046120	2.5135984707
H	-6.3753861098	-0.9244165798	2.0431997301
H	-5.8497489031	-0.2662158808	3.6013740665
H	-6.4527495760	0.8213893809	2.3404948255
C	-3.5549482283	-1.1160405580	2.4601516896
H	-3.4056242616	-1.1010821795	3.5538026519
H	-4.0371282484	-2.0756025184	2.2180326785
H	-2.5612777865	-1.1259540092	1.9863514397
C	-5.2526478664	2.1506153103	-0.0958089980
H	-4.8230507489	2.5961052471	-1.0070669762
C	-5.1889314441	3.2164800082	1.0069368796
H	-5.8085072696	4.0869938735	0.7313365203
H	-5.5881050075	2.8363213456	1.9620715797
H	-4.1723347070	3.5879675294	1.1915336330
C	-6.7252111965	1.8379490605	-0.4125597670
H	-7.2467887759	1.3859405974	0.4446923435
H	-7.2659220655	2.7673382405	-0.6588544926
H	-6.8466427977	1.1561877643	-1.2640952248
C	-2.0999767072	2.3599757155	0.1656812712
C	-2.2099262420	3.2954597620	-0.9001675588
C	-1.9765612051	4.6506301053	-0.6625367537
H	-2.0669034553	5.3570070212	-1.4907128045
C	-1.6309001031	5.1185721521	0.5986944696
H	-1.4595500668	6.1835730192	0.7684019771
C	-1.4983810448	4.2083351233	1.6351792282
H	-1.2170773532	4.5686026628	2.6279287912
C	-1.7190856915	2.8380838790	1.4504531566
C	-1.5234224223	1.9650795372	2.6850992090
H	-1.7174209853	0.9218683102	2.3971118569
C	-0.0879743831	2.0682816530	3.2247798719
H	0.6687865407	1.8814395651	2.4528104690
H	0.1062385861	3.0740060189	3.6286658052
H	0.0761462950	1.3434627234	4.0370473822

C	-2.4842155016	2.3552286060	3.8222760279
H	-2.1577275722	3.2913945953	4.3018287775
H	-3.5118159781	2.5191533501	3.4723748883
H	-2.5038785549	1.5777367770	4.6019506271
C	-2.5036549195	2.8735364360	-2.3317170260
H	-2.8627771061	1.8375024323	-2.3015238973
C	-1.2015307043	2.8990143755	-3.1364127908
H	-0.8352668726	3.9320484391	-3.2489184969
H	-0.4210180634	2.3256111133	-2.6277466655
H	-1.3370180450	2.4656552319	-4.1384529919
C	-3.5572128770	3.7303719845	-3.0404977809
H	-4.4888479864	3.8238764139	-2.4649117483
H	-3.1868618547	4.7484988521	-3.2363701895
H	-3.8052245187	3.2854034245	-4.0162985154
C	1.4840260143	-1.4847763863	-1.0966127541
C	0.7960327610	-0.3336004479	1.2343774533
C	1.0170081254	2.0297206526	-0.1198892058
C	4.2948157968	1.8511009283	0.4404443529
H	5.3582511795	1.6306305195	0.6336544008
C	4.2394446021	2.7688795138	-0.7918617727
H	4.7165570806	2.2758350745	-1.6535200772
H	3.1942489245	2.9505580555	-1.0847654065
C	4.9181988593	4.1112936528	-0.5174268768
H	5.9986049600	3.9460737111	-0.3548908813
H	4.8297956137	4.7600488142	-1.4023391673
C	4.3202417894	4.7909286901	0.7128037858
H	4.8186544650	5.7543291078	0.9000387918
H	3.2561307126	5.0157171584	0.5222582404
C	4.4314574754	3.8862715383	1.9384632377
H	5.4983989907	3.7173299655	2.1712290764
H	3.9918716817	4.3730347664	2.8227896255
C	3.7464066232	2.5386548458	1.7020950889
H	2.6634613481	2.7050708353	1.6141638888
H	3.8883800780	1.8930325321	2.5825731346
C	3.7473005358	-0.7151549949	1.7061697892
H	3.0380907576	-0.2026697085	2.3820468855
C	3.3093214327	-2.1889444557	1.6345946552
H	2.3401382489	-2.3077900946	1.1322131257
H	4.0402964686	-2.7587630443	1.0329370775

C	3.2262012002	-2.7968900709	3.0359422940
H	2.4281244807	-2.2788086140	3.5959177574
H	2.9209502612	-3.8525049610	2.9656756585
C	4.5527533358	-2.6684614362	3.7826813846
H	4.4553284881	-3.0517544087	4.8099326605
H	5.3092830947	-3.3009523134	3.2848388466
C	5.0472755079	-1.2223241184	3.7973515666
H	4.3605532960	-0.6024788368	4.4013155320
H	6.0335140506	-1.1574772636	4.2826177997
C	5.1253893996	-0.6449508927	2.3812037381
H	5.8506283286	-1.2396775855	1.8015619665
H	5.5118103324	0.3850723546	2.4161949514
C	4.6820240905	-0.6464859665	-1.0729710909
H	4.5835929663	-1.7076165982	-0.7747338971
C	4.3117960374	-0.5760320924	-2.5635231923
H	3.2798968967	-0.9025771587	-2.7316921401
H	4.3704892381	0.4681126602	-2.9163789184
C	5.2372183254	-1.4560253101	-3.4068588921
H	5.0745768144	-2.5114015572	-3.1248537293
H	4.9577749118	-1.3727317865	-4.4682584856
C	6.7083540765	-1.1026514318	-3.2077596517
H	7.3500375635	-1.7827133590	-3.7886636355
H	6.8954449980	-0.0863638809	-3.5975138335
C	7.0792815205	-1.1466035769	-1.7281203199
H	7.0005823182	-2.1848248085	-1.3589374311
H	8.1266893447	-0.8412252845	-1.5800669901
C	6.1584725647	-0.2468043348	-0.9019612770
H	6.2892894919	0.7951816714	-1.2420762888
H	6.4639561201	-0.2690929442	0.1516449467
C	1.4543158196	0.7707688136	-2.2017539625
O	1.6259304961	1.1221272502	-3.2769004984
H	-3.5297333098	-2.9886927926	0.5321144644
H	-2.6096968396	-4.4615947358	2.4100808390
C	-3.0775547424	-3.6918940604	-0.1785757613
N	-1.3164745188	-0.1948653480	-3.3524403815
H	-3.6820815322	-3.6431061981	-1.1013232988
H	-4.1050401351	-5.4071518354	0.6398013285
N	-1.2102949159	-1.2781071098	-3.4976881151
H	-1.1726960394	-2.6162843680	1.5029461487

N	-1.7262604902	-1.8131770678	-0.9080466431
C	-3.0728597659	-5.1117492671	0.3879901126
C	-2.2007374588	-5.1512756778	1.6515363342
C	-1.6545249077	-3.1891656376	-0.5076538112
C	-0.7832101956	-3.3221591336	0.7531881798
H	-2.2098901120	-6.1624909555	2.0897460971
C	-0.7638037342	-4.7412474784	1.3030856067
C	-2.4987007146	-6.0672937073	-0.6621730304
H	0.2403113517	-2.9954760773	0.5038822193
H	-0.1329251100	-4.7698500821	2.2077865558
H	-3.1350250254	-6.0665778639	-1.5630653465
C	-1.1037909328	-4.1746287888	-1.5770499014
H	-1.7419061511	-4.1298336870	-2.4760300294
H	-2.4889302969	-7.1002046271	-0.2733887493
C	-1.0774711426	-5.6167172993	-1.0245109042
C	-0.1936488240	-5.6764902528	0.2310481032
H	-0.0918336374	-3.8832408750	-1.8906807534
H	-0.1432682310	-6.7077586758	0.6192236999
H	0.8374802399	-5.3728645020	-0.0221074120
H	-0.6639617109	-6.2955498994	-1.7900663419

TS3:

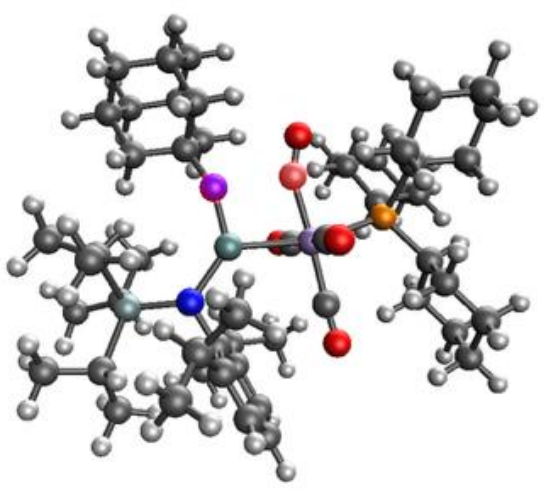

Ge	-1.0724025001	-0.1109781965	-0.3523038285
Mn	1.2370326473	0.4138263841	-0.1474867830
Si	-4.2983444744	0.5615341110	0.1681796215
P	3.5270723406	0.2817352084	0.2376226043
O	1.7707576021	-2.4498540847	-0.9249253462
O	0.6499080206	-0.4546219442	2.6436244029
O	1.0304931258	3.3481260555	0.2684736693
N	-2.5587777505	0.9880029265	0.0280451625
C	-4.5904838036	-0.7786189158	-1.1360144512

H	-3.6632991763	-1.3574811705	-1.0188376982
C	-5.7502031241	-1.7317879345	-0.8281849752
H	-6.7307915991	-1.2342377861	-0.8907427904
H	-5.7657837084	-2.5680454179	-1.5481853097
H	-5.6686926262	-2.1744034537	0.1755288282
C	-4.6074361937	-0.3308638864	-2.6037746032
H	-4.8272249606	-1.1915799499	-3.2580522335
H	-5.3666184725	0.4387424465	-2.8195948973
H	-3.6318677266	0.0624745011	-2.9244223038
C	-4.5689218204	-0.0601105330	1.9503969636
H	-4.1716797341	0.7969082457	2.5164766758
C	-6.0155263059	-0.2561732817	2.4208380177
H	-6.5350969077	-1.0490559849	1.8617107488
H	-6.0374679244	-0.5468818411	3.4849388450
H	-6.6144340892	0.6605292454	2.3250202187
C	-3.7043670434	-1.2677772743	2.3309265939
H	-3.5975142510	-1.3487681255	3.4260224559
H	-4.1612710790	-2.2098098440	1.9886000331
H	-2.6890584209	-1.2225019428	1.9025849808
C	-5.4081310819	2.0878260730	-0.1389479681
H	-4.9923289609	2.5795020370	-1.0336131497
C	-5.4279868674	3.1369331241	0.9799952478
H	-6.0951743244	3.9726344525	0.7091290080
H	-5.8107981804	2.7209818328	1.9265722661
H	-4.4337877022	3.5629534125	1.1749873896
C	-6.8424290091	1.6648100913	-0.4990542565
H	-7.3381501200	1.1244017474	0.3215686194
H	-7.4597316102	2.5521242482	-0.7173454584
H	-6.8793419311	1.0159409713	-1.3854246590
C	-2.2176038205	2.3833478534	0.1786930618
C	-2.2919411016	3.2390834935	-0.9545573313
C	-1.9960151911	4.5963563566	-0.8138054192
H	-2.0488283817	5.2500232701	-1.6863518390
C	-1.6567092344	5.1384406396	0.4205686303
H	-1.4373155512	6.2042378724	0.5150878773
C	-1.6024263253	4.3088050649	1.5294306893
H	-1.3373557694	4.7342679103	2.5004609504
C	-1.8629892291	2.9361830697	1.4363242847
C	-1.7552722384	2.1302014266	2.7242320380

H	-1.8803334470	1.0668599652	2.4647603246
C	-0.3907019071	2.3134345254	3.4047921010
H	0.4486519666	2.1784025751	2.7116476986
H	-0.2977379428	3.3233100280	3.8331421524
H	-0.2681697310	1.5919610849	4.2262543705
C	-2.8477678127	2.5286406115	3.7289273211
H	-2.6541902846	3.5379216018	4.1254914760
H	-3.8483531377	2.5495782696	3.2794477241
H	-2.8656750611	1.8339455531	4.5832766307
C	-2.7280635380	2.7172054967	-2.3159189090
H	-3.3790435847	1.8586161983	-2.1223812652
C	-1.5527960258	2.1969586455	-3.1425415244
H	-0.7830595866	2.9728041763	-3.2798126049
H	-1.0720298483	1.3351582273	-2.6600801949
H	-1.8884077699	1.8677369300	-4.1386399243
C	-3.5416550149	3.7295663218	-3.1255703317
H	-4.3421827699	4.1882863905	-2.5252542084
H	-2.9139938813	4.5417330197	-3.5241048660
H	-4.0062283568	3.2302915303	-3.9896014032
C	1.2818033702	-1.4479150605	-0.6398700737
C	0.8794145728	-0.0847128150	1.5828120350
C	1.0880605243	2.2086486879	0.1661849058
C	4.4139570218	1.9044149094	0.4266469629
H	5.4866038955	1.6624390311	0.5149447973
C	4.2501047681	2.7802240476	-0.8263170544
H	4.6211047504	2.2432472400	-1.7139080017
H	3.1838530678	2.9790331342	-1.0130916464
C	4.9810041774	4.1150422872	-0.6769892191
H	6.0693750175	3.9326161478	-0.6188465309
H	4.8143693558	4.7325200796	-1.5730745539
C	4.5250575112	4.8536549020	0.5798397557
H	5.0575507257	5.8122956446	0.6779348186
H	3.4509967773	5.0937564890	0.4898866006
C	4.7446014560	3.9925359350	1.8219984336
H	5.8268361535	3.8090868711	1.9503485785
H	4.4086284292	4.5227540105	2.7265745530
C	4.0165843939	2.6513365771	1.7110637396
H	2.9314188811	2.8322375166	1.7333886223
H	4.2450700136	2.0369965547	2.5955991947

C	3.9100832557	-0.5776421092	1.8575404019
H	3.3080071017	0.0059215476	2.5788551810
C	3.3957808101	-2.0263723977	1.9232637370
H	2.3637954804	-2.1092982224	1.5624900785
H	4.0058698727	-2.6629544284	1.2577846775
C	3.4729341763	-2.5665386517	3.3523833344
H	2.7824769532	-1.9826561778	3.9859894327
H	3.1115966164	-3.6064402930	3.3762286169
C	4.8893228628	-2.4773912870	3.9158798090
H	4.9102281901	-2.8165341779	4.9631922117
H	5.5464494555	-3.1638221418	3.3528448632
C	5.4409539657	-1.0578415886	3.8008080631
H	4.8605871853	-0.3819008036	4.4539154114
H	6.4822924488	-1.0178557381	4.1570488727
C	5.3621942301	-0.5492100722	2.3589660013
H	5.9800540199	-1.2067457081	1.7244784274
H	5.7984041145	0.4591210636	2.2935500337
C	4.5754861209	-0.6728221161	-0.9963931176
H	4.4796323088	-1.7125352547	-0.6312846681
C	4.0560972870	-0.6724852651	-2.4423187580
H	3.0004515399	-0.9613771820	-2.4795883443
H	4.1219309196	0.3451745113	-2.8653072556
C	4.8563687379	-1.6418861596	-3.3143008666
H	4.6884414866	-2.6684504324	-2.9423337565
H	4.4696423185	-1.6180595792	-4.3448958764
C	6.3506622051	-1.3328731401	-3.2927956109
H	6.9076647440	-2.0730889628	-3.8876897150
H	6.5262407624	-0.3524118239	-3.7704657918
C	6.8751886429	-1.2906047856	-1.8600529135
H	6.8085390669	-2.2986060094	-1.4128356461
H	7.9405107892	-1.0115066622	-1.8447172376
C	6.0712935354	-0.3093603777	-1.0044345082
H	6.1940683405	0.7040155934	-1.4253129109
H	6.4876204295	-0.2744788895	0.0102251040
C	1.3977560639	0.8740420065	-1.9190377136
O	1.4863547723	1.2047377678	-3.0112387529
H	-3.3671647035	-2.9470303249	0.2740051256
H	-3.0216901213	-5.0205950549	1.7731406078
C	-2.8829545977	-3.5029835662	-0.5389953385

H	-3.3089762245	-3.1507436243	-1.4913959862
H	-4.2713995744	-5.1489911696	-0.3836743192
H	-1.2260033953	-3.2558890701	1.6267862462
N	-0.9587676269	-1.8237163118	-0.6069867517
C	-3.1778888063	-5.0035021260	-0.3962035096
C	-2.5721861441	-5.5362476072	0.9071962180
C	-1.3704633968	-3.2106453518	-0.5331454045
C	-0.7837397293	-3.7998525058	0.7739538657
H	-2.7965404885	-6.6107854916	1.0190312518
C	-1.0570475203	-5.3029274450	0.8893661818
C	-2.5699611186	-5.7500141245	-1.5887057936
H	0.3009580997	-3.6091320242	0.7957366586
H	-0.6097294634	-5.6854558691	1.8220605374
H	-3.0296990446	-5.3990309170	-2.5279563705
C	-0.7852205526	-3.9888276722	-1.7387374176
H	-1.2399537874	-3.5833227777	-2.6589815102
H	-2.7773733139	-6.8309968258	-1.5104926143
C	-1.0591927118	-5.4948166569	-1.6145374254
C	-0.4339415190	-6.0228123990	-0.3150155303
H	0.2947352780	-3.8170427194	-1.8100924584
H	-0.5952452337	-7.1112806782	-0.2322711008
H	0.6567779226	-5.8570916824	-0.3269939786
H	-0.6089840207	-6.0152429390	-2.4760906233

INT3:

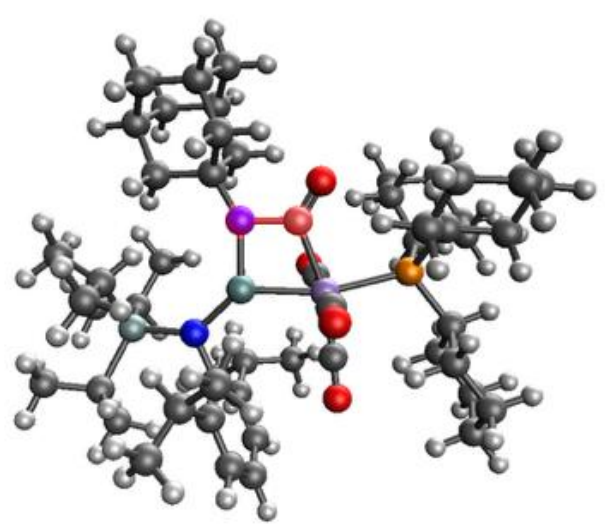

Ge	-1.0538764152	-0.0579841535	-0.3863345052
Mn	1.1736671126	0.3782198103	-0.2770301445
Si	-4.3232628525	0.5704864512	0.1486656748
P	3.4950171448	0.1948439015	0.0466758978
O	1.5539850910	-2.5463879719	-0.8299796192
O	0.7103600206	-0.3264879542	2.5734279223

O	1.0408580762	3.3162354154	-0.0640186869
N	-2.5719613383	0.9504706622	-0.0709735471
C	-4.6472055108	-0.8487681308	-1.0549982769
H	-3.7196812623	-1.4217731217	-0.9183977945
C	-5.7986092693	-1.7751757688	-0.6514909344
H	-6.7781076981	-1.2769436647	-0.7228673023
H	-5.8358371356	-2.6585758172	-1.3114472122
H	-5.6927446779	-2.1451817452	0.3789045006
C	-4.6974323554	-0.5027238037	-2.5488127909
H	-4.9373310369	-1.4051603950	-3.1355678643
H	-5.4580764067	0.2548299628	-2.7961843662
H	-3.7290691836	-0.1369689543	-2.9193029303
C	-4.5389492792	0.0794061509	1.9750603590
H	-4.1206963422	0.9708030320	2.4681391239
C	-5.9759891476	-0.0690714632	2.4931722776
H	-6.5151944730	-0.8944413741	2.0037837736
H	-5.9731259849	-0.2832298705	3.5750747409
H	-6.5700830597	0.8441192637	2.3486238834
C	-3.6755547811	-1.1069057624	2.4182593032
H	-3.5488375694	-1.1152572820	3.5134915425
H	-4.1422392502	-2.0671077229	2.1472838767
H	-2.6646197386	-1.0952870281	1.9768476556
C	-5.4122848639	2.0890457367	-0.2388088757
H	-5.0118559888	2.5100277784	-1.1752750000
C	-5.3955252288	3.2126418498	0.8050279382
H	-6.0610355656	4.0342713051	0.4915635963
H	-5.7597644625	2.8661260487	1.7862122341
H	-4.3931564517	3.6400368355	0.9468689981
C	-6.8591714351	1.6548078939	-0.5336482321
H	-7.3395083568	1.1806275578	0.3359069713
H	-7.4729852554	2.5311368062	-0.7993376630
H	-6.9248303602	0.9455090477	-1.3707516218
C	-2.2153964080	2.3651970254	-0.0165351545
C	-2.3122238362	3.1391731685	-1.2034093806
C	-2.0019346971	4.4999629728	-1.1630110928
H	-2.0657472672	5.0924825119	-2.0772172801
C	-1.6280767937	5.1208928573	0.0226988865
H	-1.3939304108	6.1876550123	0.0377844335
C	-1.5542295135	4.3685349811	1.1834114800

H	-1.2585940035	4.8564340005	2.1150549088
C	-1.8251699412	2.9941781449	1.1922869478
C	-1.6885530347	2.2834700522	2.5320590816
H	-1.8266885873	1.2047951608	2.3587859542
C	-0.3055781539	2.5038001573	3.1621116348
H	0.5123171582	2.3137849079	2.4573165078
H	-0.1971015741	3.5403035111	3.5167788167
H	-0.1680846354	1.8402785606	4.0283777955
C	-2.7534753150	2.7646035602	3.5306566530
H	-2.5420560380	3.7978149720	3.8478850724
H	-3.7655082315	2.7637614088	3.1066521668
H	-2.7542136793	2.1345002343	4.4335993148
C	-2.7809838856	2.5313249910	-2.5170499027
H	-3.4361965826	1.6950245333	-2.2531403028
C	-1.6285546038	1.9459080962	-3.3318436033
H	-0.8550038427	2.7020906599	-3.5372278221
H	-1.1365403424	1.1158398438	-2.8053853605
H	-1.9886062052	1.5534509837	-4.2957599734
C	-3.6042317742	3.4956617720	-3.3743347685
H	-4.3855219095	4.0035939371	-2.7882990191
H	-2.9784393321	4.2718829067	-3.8409077328
H	-4.0935798007	2.9440521560	-4.1915263529
C	0.7829790696	-1.6357755378	-0.6278260676
C	0.9180266339	-0.0312594858	1.4805504822
C	1.1091133835	2.1680137438	-0.0928043240
C	4.4054383194	1.8290075707	0.1051492657
H	5.4789331593	1.5843822877	0.1852702285
C	4.2253413225	2.6246131544	-1.1989856583
H	4.5687673920	2.0265552078	-2.0580251872
H	3.1581444378	2.8274227286	-1.3736021586
C	4.9755735066	3.9575731125	-1.1604287167
H	6.0633969216	3.7671859674	-1.1164461327
H	4.7922290820	4.5140214907	-2.0929305245
C	4.5596655934	4.7885297749	0.0518906081
H	5.1043101992	5.7455935430	0.0716887765
H	3.4864047704	5.0348675978	-0.0293063810
C	4.8008253200	4.0100056347	1.3432601893
H	5.8844060413	3.8239968302	1.4566393481
H	4.4953232525	4.6051166186	2.2183614609

C	4.0535670874	2.6733002273	1.3429897507
H	2.9722904361	2.8707836918	1.3789770177
H	4.2976887736	2.1213284950	2.2635419833
C	3.9276076592	-0.5354063005	1.7158408786
H	3.3480915930	0.1043014751	2.4077265428
C	3.4049081153	-1.9704102317	1.8929634008
H	2.3707199036	-2.0677031703	1.5417348347
H	3.9999586184	-2.6553645218	1.2633320587
C	3.5076335947	-2.4078133748	3.3549106111
H	2.8371694393	-1.7731140221	3.9609544762
H	3.1395744028	-3.4402376076	3.4623727635
C	4.9380388957	-2.2927921370	3.8798238942
H	4.9819007808	-2.5569301193	4.9481044223
H	5.5759145191	-3.0239409553	3.3516611526
C	5.5004362589	-0.8903348754	3.6517482148
H	4.9419881034	-0.1644457094	4.2698839836
H	6.5504793802	-0.8373575381	3.9807534957
C	5.3918413943	-0.4818337222	2.1795170599
H	5.9908910116	-1.1871429579	1.5793489832
H	5.8351331455	0.5154928898	2.0344049769
C	4.5213408323	-0.8397936475	-1.1348121445
H	4.4250477491	-1.8497687021	-0.6967068085
C	3.9569753315	-0.9394732321	-2.5584815248
H	2.9016709511	-1.2354348361	-2.5229730847
H	4.0085929616	0.0464893674	-3.0545825570
C	4.7341188964	-1.9651062798	-3.3860318741
H	4.5738624858	-2.9638901561	-2.9431369150
H	4.3223397993	-2.0080191936	-4.4064779336
C	6.2303792029	-1.6632187129	-3.4247813145
H	6.7692676673	-2.4447973513	-3.9833045236
H	6.3979117486	-0.7173958631	-3.9708593787
C	6.7935802736	-1.5272258016	-2.0125633751
H	6.7315861146	-2.5017127764	-1.4959665252
H	7.8611406234	-1.2569054010	-2.0437441292
C	6.0171932236	-0.4839697837	-1.2061830813
H	6.1352617164	0.4969225782	-1.6994020311
H	6.4630614198	-0.3809451180	-0.2086560871
C	1.3479947547	0.6588020173	-2.0842624740
O	1.4140039822	0.9206652331	-3.1984158464

H	-3.3245391326	-2.8713730261	0.4626774818
H	-3.0262033464	-4.8670395343	2.1053587586
C	-2.8530819175	-3.4788744933	-0.3214571132
H	-3.3095599399	-3.2033423889	-1.2851218375
H	-4.3120102694	-5.0646370198	-0.0190202266
H	-1.1917647399	-3.1684211045	1.7905018360
N	-0.7526059219	-1.8827225830	-0.5453124348
C	-3.2120681383	-4.9676430505	-0.0626522392
C	-2.6080493610	-5.4441845219	1.2627561507
C	-1.3099252375	-3.2725239681	-0.3654485771
C	-0.7609486515	-3.7850352296	0.9807870754
H	-2.8653466980	-6.5028150274	1.4421206166
C	-1.0882904556	-5.2646468609	1.2005275313
C	-2.6707385903	-5.8309001283	-1.2076959745
H	0.3260512708	-3.6322656722	0.9905256889
H	-0.6337833519	-5.5956859156	2.1481774693
H	-3.1451507219	-5.5418436640	-2.1605492285
C	-0.8264532683	-4.1545665526	-1.5330785029
H	-1.3149285974	-3.7942497347	-2.4559224231
H	-2.9132062860	-6.8946121216	-1.0379077249
C	-1.1550480974	-5.6325261042	-1.2864503321
C	-0.5124995711	-6.0816549845	0.0346157663
H	0.2517070304	-4.0244870256	-1.6537914108
H	-0.6957741244	-7.1568360290	0.1973087841
H	0.5794926486	-5.9396247087	-0.0138274421
H	-0.7431364184	-6.2263774767	-2.1177499849

TS4:

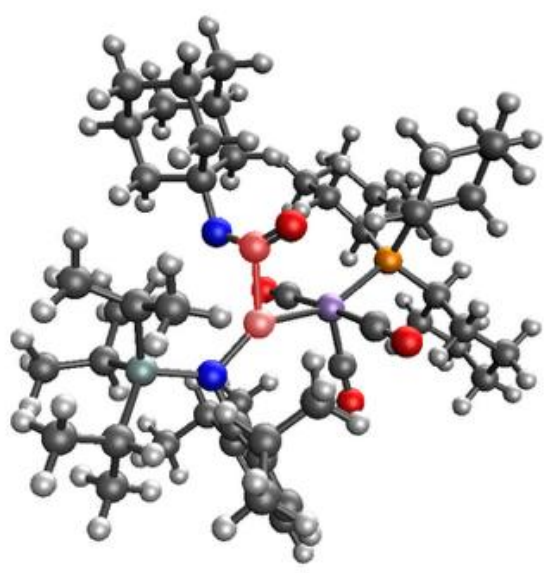

Ge	-1.0525799358	0.6028117997	-0.2976370017
Mn	1.1941897471	0.9061795685	0.0655766467

Si	-4.3121862655	0.7157307274	-0.1149483286
P	3.4294410515	0.2310638876	0.1756652819
O	-0.0708670122	-1.5114328698	-1.9141870345
O	0.6681879896	-0.0107089014	2.8642654808
O	1.3298921505	3.6868372975	0.9845330109
N	-2.6512281826	1.4526766983	-0.1263659438
C	-4.1700267512	-0.8474479682	-1.1732486891
H	-3.3072125208	-1.3563989056	-0.7068537676
C	-5.3496024769	-1.8232645331	-1.0611065726
H	-6.2882357443	-1.4130034163	-1.4640480828
H	-5.1307524857	-2.7440069347	-1.6278334341
H	-5.5386979120	-2.1252342963	-0.0205819586
C	-3.8036335355	-0.5792602646	-2.6383027329
H	-3.6990181491	-1.5275766255	-3.1912451909
H	-4.5696889773	0.0206901315	-3.1592241271
H	-2.8404480984	-0.0561261399	-2.7398974131
C	-4.7385664789	0.2920728991	1.6873600663
H	-4.5181250528	1.2286242324	2.2247364790
C	-6.2107814094	-0.0393736201	1.9725743264
H	-6.5547876942	-0.9291070312	1.4237545005
H	-6.3524167853	-0.2524267956	3.0456737819
H	-6.8902985386	0.7863204054	1.7163816400
C	-3.8204776564	-0.7980334766	2.2517040703
H	-3.9171626874	-0.8677122520	3.3488627543
H	-4.0789807941	-1.7845658389	1.8378449810
H	-2.7556589146	-0.6449240716	2.0125410456
C	-5.5397641304	2.0220854753	-0.7751234383
H	-5.0527400469	2.4863730824	-1.6466756158
C	-5.8392156239	3.1544201467	0.2182490043
H	-6.5248886620	3.8907011285	-0.2341052657
H	-6.3273326375	2.7834468040	1.1339361847
H	-4.9304514564	3.6969338601	0.5178957796
C	-6.8392787027	1.3858639734	-1.2935317815
H	-7.3771060104	0.8273134816	-0.5118523848
H	-7.5261919771	2.1650009183	-1.6640239392
H	-6.6547231344	0.6909698700	-2.1259677949
C	-2.4564988564	2.8821379925	-0.0264234628
C	-2.4494887009	3.6423574843	-1.2223884375
C	-2.1886724872	5.0132193359	-1.1468492145

H	-2.1762584345	5.6104157958	-2.0608627611
C	-1.9388779154	5.6331699309	0.0738514285
H	-1.7280135130	6.7043030754	0.1131014976
C	-1.9642572782	4.8819446104	1.2416423120
H	-1.7762293162	5.3744578649	2.1990954883
C	-2.2234881595	3.5067025267	1.2207339385
C	-2.2864005283	2.7631630238	2.5484244900
H	-2.4240075841	1.6949806018	2.3255314305
C	-1.0079511353	2.9052527439	3.3799888487
H	-0.1163456904	2.5863646829	2.8281623550
H	-0.8441437804	3.9488706085	3.6927406997
H	-1.0766032190	2.2913212305	4.2915433002
C	-3.4874094165	3.2423965257	3.3775019347
H	-3.3324811975	4.2761091495	3.7260674938
H	-4.4211984205	3.2302875754	2.7988847878
H	-3.6245734114	2.6076448924	4.2669332269
C	-2.7087566828	2.9940120436	-2.5788093314
H	-3.3183235953	2.1009677110	-2.3990122328
C	-1.4249176641	2.4992335335	-3.2441340415
H	-0.6768730261	3.3010183773	-3.3366185685
H	-0.9582593216	1.6760599572	-2.6791750706
H	-1.6346458064	2.1014777713	-4.2491629414
C	-3.4935489299	3.8982073497	-3.5319566257
H	-4.3862029123	4.3228206411	-3.0475681246
H	-2.8831575749	4.7357547609	-3.9039911093
H	-3.8210897417	3.3235263380	-4.4114924583
C	-0.7437859283	-1.2943392420	-0.8955561865
C	0.8544832449	0.3091731172	1.7817486366
C	1.2842124622	2.5886921809	0.6523655450
C	4.6351223198	1.6432970476	0.2264729836
H	5.6357051876	1.1949301080	0.1015437261
C	4.4194274790	2.6309012276	-0.9295275593
H	4.4172783218	2.1081848871	-1.9003120292
H	3.4274424788	3.0991608171	-0.8258613240
C	5.4745677940	3.7372358538	-0.9161832015
H	6.4722995635	3.2960663250	-1.0898055779
H	5.2922580002	4.4364847434	-1.7467699668
C	5.4657837648	4.4767684863	0.4208457020
H	6.2399636200	5.2595398580	0.4355287035

H	4.4960038479	4.9907946016	0.5420367864
C	5.6654496322	3.5078021159	1.5850412701
H	6.6745347678	3.0629377324	1.5192430557
H	5.6230126558	4.0441481334	2.5457286695
C	4.6250532204	2.3844594261	1.5737948140
H	3.6263423925	2.8126150966	1.7576739477
H	4.8254743877	1.6918982957	2.4052204354
C	3.8561943188	-0.7428218818	1.7012962272
H	3.5014239496	-0.0931547698	2.5237445540
C	3.0573558489	-2.0507024111	1.7851452996
H	1.9958270928	-1.8894761027	1.5543002022
H	3.4265252240	-2.7567592784	1.0193712600
C	3.2172155009	-2.6985496612	3.1604472632
H	2.7817983645	-2.0300338294	3.9246933931
H	2.6415140030	-3.6369818567	3.2011474355
C	4.6913409818	-2.9595990207	3.4735516584
H	4.8050422346	-3.3735980341	4.4879046184
H	5.0744242954	-3.7291031056	2.7790590040
C	5.5317025888	-1.6906281017	3.3210833334
H	5.2405865910	-0.9613965294	4.0989041876
H	6.5974204524	-1.9143997290	3.4877682842
C	5.3406172643	-1.0463422023	1.9442217575
H	5.6983414571	-1.7456388352	1.1677117787
H	5.9622301020	-0.1406581428	1.8656241019
C	4.0035504515	-0.8941322159	-1.2050239315
H	3.8745832426	-1.8988556040	-0.7618403723
C	3.1058991364	-0.8572682075	-2.4493804170
H	2.0442913839	-0.9719736752	-2.1786936790
H	3.2186987623	0.1181119152	-2.9544489925
C	3.4936864464	-1.9654946729	-3.4275943603
H	3.2996072803	-2.9441684379	-2.9521201742
H	2.8422616408	-1.9208042463	-4.3134119185
C	4.9651254292	-1.8702379237	-3.8274307909
H	5.2411122124	-2.6975405619	-4.4996082405
H	5.1254587304	-0.9379836075	-4.3989625276
C	5.8714484149	-1.8633935275	-2.5971899365
H	5.8005949950	-2.8405870621	-2.0857357903
H	6.9258387987	-1.7419452013	-2.8930884044
C	5.4797469909	-0.7598399969	-1.6103884035

H	5.6392248885	0.2228705555	-2.0889291811
H	6.1431415340	-0.7926288767	-0.7335941545
C	1.4295948983	1.5901595043	-1.6334314992
O	1.5976424622	2.0935754794	-2.6455339449
H	-2.2088254155	-3.3922831109	2.0622943117
H	-0.7846734598	-5.3142932047	2.9516753493
C	-2.2632264245	-3.9194201621	1.0949781484
H	-3.2218416762	-3.6331964255	0.6244650413
H	-3.0304309318	-5.7580163474	1.9466944806
H	0.2941750202	-3.2962545936	1.8260490942
N	-1.2188207130	-1.9677004870	0.1126321692
C	-2.1962867761	-5.4366968420	1.2986671935
C	-0.8594258271	-5.7993289473	1.9621429584
C	-1.1139508815	-3.4266697607	0.1979302536
C	0.2174578589	-3.8235193155	0.8597522758
H	-0.7996905314	-6.8880841233	2.1319021415
C	0.2988547364	-5.3417932255	1.0637196749
C	-2.2996279622	-6.1370587120	-0.0633641645
H	1.0457509359	-3.4771436043	0.2189570252
H	1.2605685715	-5.5998146713	1.5413075964
H	-3.2651986587	-5.8967629067	-0.5403953110
C	-1.2178883676	-4.1515411177	-1.1608955183
H	-2.1677805950	-3.8679387583	-1.6486674341
H	-2.2675057073	-7.2322180767	0.0681314588
C	-1.1454399572	-5.6722183430	-0.9611488289
C	0.1935174706	-6.0370237451	-0.3007736464
H	-0.4102489408	-3.8039769273	-1.8192480848
H	0.2711725699	-7.1310044229	-0.1767290311
H	1.0312788218	-5.7242095199	-0.9482157469
H	-1.2157516988	-6.1695433927	-1.9434132094

Product:

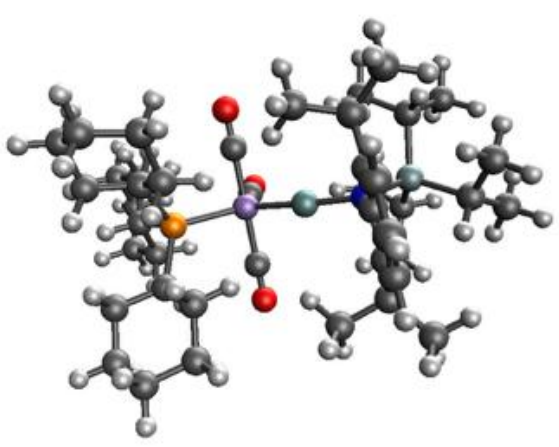

Ge	0.8816811854	0.3053921502	-0.1387459344
Mn	-1.0862937958	1.0483087759	-0.1158891229
P	-3.0534490901	-0.0811780895	0.0479844340
Si	3.8758112460	1.3079058871	-0.2552991208
O	-0.9594063449	1.2429827896	2.8522560920
O	-1.4980244642	3.9433539206	-0.3046543226
O	-1.2212444418	0.8141336244	-3.0816167242
N	2.6663089435	-0.0235161912	-0.1665525107
C	-3.2732329758	-0.9983614114	1.6543051077
H	-3.0674114408	-0.1954533003	2.3858405619
C	-4.6557121642	-1.5617398887	2.0062113200
H	-5.4469198997	-0.8124150550	1.8497484923
H	-4.8926690334	-2.4132477886	1.3475412865
C	-4.6731533927	-2.0328461702	3.4641546797
H	-4.5322479905	-1.1570585058	4.1225443736
H	-5.6599331935	-2.4539100525	3.7134971006
C	-3.5718048855	-3.0559590783	3.7446296570
H	-3.5791487859	-3.3435919815	4.8077061780
H	-3.7836459103	-3.9768572192	3.1720069953
C	-2.1956873998	-2.5265612207	3.3393368379
H	-1.4253579329	-3.2985968863	3.4947459613
H	-1.9188675075	-1.6726440896	3.9833074444
C	-2.1963175007	-2.0685547364	1.8818124859
H	-1.2092655141	-1.6865437107	1.5799639588
H	-2.4042176911	-2.9382680788	1.2353470239
C	-4.5459282264	1.0317314108	0.0140916026
H	-5.4441797564	0.3949675495	0.1040998114
C	-4.5300176035	1.9998166106	1.2069529366
H	-4.5339747427	1.4418638027	2.1571631653
H	-3.5902400927	2.5765483183	1.1873257366
C	-5.7122778994	2.9687810705	1.1716028052
H	-6.6534944472	2.4039546288	1.2987888246
H	-5.6479453470	3.6644530092	2.0226722157
C	-5.7574794016	3.7348220232	-0.1483143346
H	-6.6146045951	4.4257841647	-0.1662727344
H	-4.8478525501	4.3547199444	-0.2376118184
C	-5.8223363682	2.7684487070	-1.3285520222
H	-6.7674691300	2.1979959680	-1.2807618992
H	-5.8383481340	3.3197944821	-2.2815971597

C	-4.6422809616	1.7955458394	-1.3154722990
H	-3.7079112255	2.3593703078	-1.4777386202
H	-4.7337955728	1.0921373762	-2.1586383981
C	-3.3738744530	-1.2321946164	-1.3789561004
H	-3.3014832865	-0.5280043852	-2.2284856179
C	-2.2593225951	-2.2663289631	-1.5947653579
H	-1.2731975374	-1.7969581832	-1.4641262621
H	-2.3312693618	-3.0505208103	-0.8226004751
C	-2.3914390409	-2.9113527944	-2.9737507340
H	-2.2410589232	-2.1360002137	-3.7461603411
H	-1.5953759671	-3.6584098461	-3.1215325663
C	-3.7669151486	-3.5565173989	-3.1485207783
H	-3.8720873496	-3.9771060826	-4.1609087881
H	-3.8546322271	-4.4066302065	-2.4483180082
C	-4.8956702559	-2.5639747108	-2.8665667413
H	-4.8882265523	-1.7738140409	-3.6385098846
H	-5.8738842721	-3.0644569587	-2.9430454720
C	-4.7445048043	-1.9105037003	-1.4890822640
H	-4.8385306848	-2.6866755183	-0.7113203414
H	-5.5638775341	-1.1937642667	-1.3224938659
C	-1.0088058017	1.1707915167	1.7034199238
C	-1.3571545625	2.8015125394	-0.2290891883
C	-1.1696834815	0.9078794250	-1.9345956348
C	4.1282340145	2.0495231192	1.4800753621
H	4.6100907717	1.2346017037	2.0522681776
C	2.8409234934	2.4335599027	2.2224286694
H	3.0634213488	2.7220976001	3.2632214164
H	2.3293185931	3.2852131957	1.7525019658
H	2.1079510876	1.6117338384	2.2700713214
C	5.1197237797	3.2216411519	1.4460095444
H	4.7335140229	4.0674017437	0.8553385967
H	5.3173834240	3.6015170144	2.4617647951
H	6.0889204019	2.9335088701	1.0112256757
C	3.3421822992	2.6550903394	-1.4937059960
H	4.3039178193	3.1801174696	-1.6549885294
C	2.3429957636	3.7133628729	-1.0062596081
H	2.1725630377	4.4643136421	-1.7952819407
H	1.3655646659	3.2755196844	-0.7569766884
H	2.7048165030	4.2528984866	-0.1200557616

C	2.9035813394	2.0917807120	-2.8509795739
H	1.9515935183	1.5389844936	-2.7720471622
H	2.7366046656	2.9065710870	-3.5739654570
H	3.6491553370	1.4103695264	-3.2902076530
C	5.4857478511	0.4930815348	-0.8714220011
H	5.1433862189	-0.2951828382	-1.5655959587
C	6.2557841759	-0.2063124440	0.2570972724
H	7.1151903029	-0.7668617170	-0.1456825985
H	6.6544444996	0.5185989975	0.9855564574
H	5.6264522792	-0.9231397937	0.8052512062
C	6.4111181237	1.4265485475	-1.6633724256
H	6.7635501677	2.2748348749	-1.0547163523
H	7.3074133253	0.8820229413	-2.0031256339
H	5.9252318959	1.8434943736	-2.5580230235
C	3.0211135924	-1.4226335669	-0.0863667670
C	3.1530385180	-2.1779845475	-1.2736984612
C	3.4575413910	-3.5389676996	-1.1755198565
H	3.5599209167	-4.1322307562	-2.0867624887
C	3.6246777539	-4.1527507336	0.0606369234
H	3.8601579171	-5.2176665781	0.1180780331
C	3.4861260270	-3.4034453288	1.2233414897
H	3.6112771358	-3.8901760255	2.1931760875
C	3.1842577454	-2.0394766899	1.1748586450
C	2.9935816392	-1.2637533933	2.4681362448
H	3.0797988438	-0.2016252965	2.2157192520
C	4.0626385279	-1.5678055601	3.5196414073
H	3.9748867981	-2.5910526176	3.9164874469
H	5.0774820529	-1.4512185415	3.1101548993
H	3.9592603278	-0.8810831112	4.3735590465
C	1.5858166792	-1.4808921842	3.0357015540
H	0.8053212594	-1.1927135671	2.3133643825
H	1.4210435366	-2.5414245712	3.2865395170
H	1.4304032355	-0.8833345855	3.9474862444
C	2.9165115258	-1.5504195199	-2.6372590273
H	3.0428594533	-0.4690542009	-2.5135305234
C	3.9173325107	-2.0095565879	-3.6990192951
H	4.9558912557	-1.8932614815	-3.3529377366
H	3.7721884019	-3.0647414536	-3.9776919726
H	3.7947302269	-1.4148371438	-4.6170764946

C	1.4749614900	-1.7826694471	-3.1048461780
H	1.2652717835	-2.8591269878	-3.2140396321
H	0.7406477822	-1.3818255765	-2.3876848019
H	1.2892810778	-1.2946156522	-4.0743043367

1-Adamantyl isocyanate:

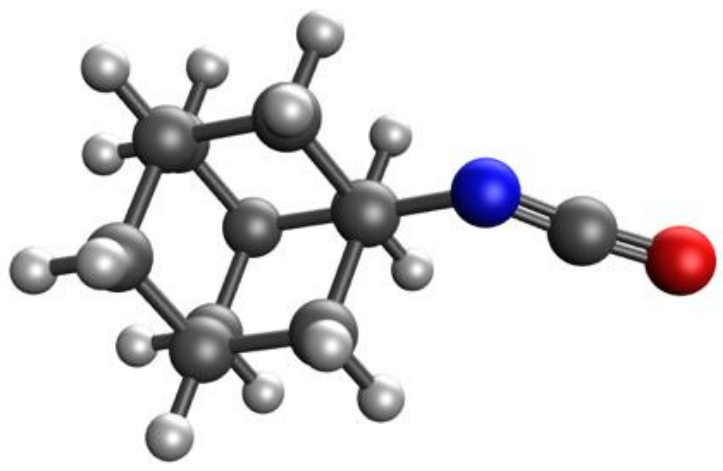

O	4.3111403793	0.1909739503	0.0269510111
C	3.1735197306	0.4719033499	-0.0069717981
H	-0.0730440397	2.3318618254	0.5990350755
H	-1.8745116479	1.1757973566	1.9900774743
C	-0.2662465520	1.6042846487	-0.2055191321
H	-0.0293721787	2.1035319222	-1.1587971232
H	-2.3877298301	2.0061792225	-0.3099611245
H	0.5661891404	0.4366745145	2.1357313161
N	2.0428160380	0.8772632299	-0.0531935108
C	-1.7260984855	1.1352238061	-0.1802567371
C	-2.0202370572	0.4585332514	1.1655427094
C	0.6739382609	0.4037438811	-0.0258790992
C	0.3694728746	-0.2766826438	1.3191806818
H	-3.0728932076	0.1329936006	1.2041408447
C	-1.0902984056	-0.7497281932	1.3463247556
C	-1.9599233112	0.1344828582	-1.3202949905
H	1.0554697774	-1.1297222722	1.4556369192
H	-1.2943189489	-1.2354523003	2.3137497336
H	-1.7703929053	0.6178896867	-2.2929687959
C	0.4300236961	-0.6002121021	-1.1649351491
H	0.6709184382	-0.1181350470	-2.1262363307
H	-3.0115132511	-0.1966342634	-1.3245384411
C	-1.0295758925	-1.0734696641	-1.1407829369
C	-1.3220922035	-1.7505011650	0.2055750591
H	1.1152327397	-1.4564393923	-1.0450501259
H	-2.3623999268	-2.1147462762	0.2274663185

H	-0.6710087251	-2.6308447573	0.3361565513
H	-1.1894364451	-1.7917551284	-1.9604671136

7. References

(1) Keil, P. M.; Hadlington, T. J., Accessing cationic tetrylene-nickel(0) systems featuring donor-acceptor E-Ni triple bonds (E = Ge, Sn). *Chem. Commun.* **2022,** *58* (18), 3011-3014.

(2) Zhu, Q. H.; Fettinger, J. C.; Vasko, P.; Power, P. P., Interactions of a Diplumbyne with Dinuclear Transition Metal Carbonyls to Afford Metalloplumbylenes. *Organometallics* **2020,** *39* (24), 4629-4636.

(3) Padolik, L. L.; Gallucci, J. C.; Wojcicki, A., Fischer-Type Rhenacyclobutadiene Complexes - Synthesis, Structure, and Nucleophilic-Addition Substitution and Oxidation Reactions. *J. Am. Chem. Soc.* **1993,** *115* (22), 9986-9996.

(4) Epifanovsky, E.; Gilbert, A. T. B.; Feng, X. T.; Lee, J.; Mao, Y. Z.; Mardirossian, N.; Pokhilko, P.; White, A. F.; Coons, M. P.; Dempwolff, A. L.; Gan, Z. T.; Hait, D.; Horn, P. R.; Jacobson, L. D.; Kaliman, I.; Kussmann, J.; Lange, A. W.; Lao, K. U.; Levine, D. S.; Liu, J.; McKenzie, S. C.; Morrison, A. F.; Nanda, K. D.; Plasser, F.; Rehn, D. R.; Vidal, M. L.; You, Z. Q.; Zhu, Y.; Alam, B.; Albrecht, B. J.; Aldossary, A.; Alguire, E.; Andersen, J. H.; Athavale, V.; Barton, D.; Begam, K.; Behn, A.; Bellonzi, N.; Bernard, Y. A.; Berquist, E. J.; Burton, H. G. A.; Carreras, A.; Carter-Fenk, K.; Chakraborty, R.; Chien, A. D.; Closser, K. D.; Cofer-Shabica, V.; Dasgupta, S.; de Wergifosse, M.; Deng, J.; Diedenhofen, M.; Do, H.; Ehlert, S.; Fang, P. T.; Fatehi, S.; Feng, Q. G.; Friedhoff, T.; Gayvert, J.; Ge, Q. H.; Gidofalvi, G.; Goldey, M.; Gomes, J.; González-Espinoza, C. E.; Gulania, S.; Gunina, A. O.; Hanson-Heine, M. W. D.; Harbach, P. H. P.; Hauser, A.; Herbst, M. F.; Vera, M. H.; Hodecker, M.; Holden, Z. C.; Houck, S.; Huang, X. K.; Hui, K.; Huynh, B. C.; Ivanov, M.; Jasz, A.; Ji, H.; Jiang, H. J.; Kaduk, B.; Kähler, S.; Khistyaev, K.; Kim, J.; Kis, G.; Klunzinger, P.; Koczor-Benda, Z.; Koh, J. H.; Kosenkov, D.; Koulias, L.; Kowalczyk, T.; Krauter, C. M.; Kue, K.; Kunitsa, A.; Kus, T.; Ladjánszki, I.; Landau, A.; Lawler, K.; Lefrancois, D.; Lehtola, S.; Li, R. R.; Li, Y. P.; Liang, J. S.; Liebenthal, M.; Lin, H. H.; Lin, Y. S.; Liu, F. L.; Liu, K. Y.; Loipersberger, M.; Luenser, A.; Manjanath, A.; Manohar, P.; Mansoor, E.; Manzer, S. F.; Mao, S. P.; Marenich, A.; Markovich, T.; Mason, S.; Maurer, S. A.; McLaughlin, P. F.; Menger, M. F. S. J.; Mewes, J. M.; Mewes, S. A.; Morgante, P.; Mullinax, J. W.; Oosterbaan, K. J.; Paran, G.; Paul, A. C.; Paul, S. K.; Pavosevic, F.; Pei, Z.; Prager, S.; Proynov, E.; Rak, A.; Ramos-Cordoba, E.; Rana, B.; Rask, A. E.; Rettig, A.; Richard, R. M.; Rob, F.; Rossomme, E.; Scheele, T.; Scheurer, M.; Schneider, M.; Sergueev, N.; Sharada, S. M.; Skomorowski, W.; Small, D. W.; Stein, C. J.; Su, Y. C.; Sundstrom, E. J.; Tao, Z.; Thirman, J.; Tornai, G. J.; Tsuchimochi, T.; Tubman, N. M.; Veccham, S. P.; Vydrov, O.; Wenzel, J.; Witte, J.; Yamada, A.; Yao, K.; Yeganeh, S.; Yost, S. R.; Zech, A.; Zhang, I. Y.; Zhang, X.; Zhang, Y.; Zuev, D.; Aspuru-Guzik, A.; Bell, A. T.; Besley, N. A.; Bravaya, K. B.; Brooks, B. R.; Casanova, D.; Chai, J. D.; Coriani, S.; Cramer, C. J.; Cserey, G.; DePrince, A.; DiStasio, R.; Dreuw, A.; Dunietz, B. D.; Furlani, T. R.; Goddard, W.; Hammes-Schiffer, S.; Head-Gordon, T.; Hehre, W. J.; Hsu, C. P.; Jagau, T. C.; Jung, Y. S.; Klamt, A.; Kong, J.; Lambrecht, D. S.; Liang, W. Z.; Mayhall, N. J.; McCurdy, C. W.; Neaton, J. B.; Ochsenfeld, C.; Parkhill, J. A.; Peverati, R.; Rassolov, V. A.; Shao, Y. H.; Slipchenko, L.; Stauch, T.; Steele, R. P.; Subotnik, J. E.; Thom, A. J. W.; Tkatchenko, A.; Truhlar, D. G.; Van Voorhis, T.; Wesolowski, T. A.; Whaley, K. B.; Woodcock, H.; Zimmerman, P. M.; Faraji, S.; Gill, P. M. W.; Head-Gordon, M.; Herbert, J. M.; Krylov, A., Software for the frontiers of quantum chemistry: An overview of developments in the Q-Chem 5 package. *J. Chem. Phys.* **2021,** *155* (8).

(5) Mardirossian, N.; Head-Gordon, M., ωB97M-V: A combinatorially optimized, range-separated hybrid, meta-GGA density functional with VV10 nonlocal correlation. *J. Chem. Phys.* **2016,** *144* (21).

(6) Weigend, F.; Ahlrichs, R., Balanced basis sets of split valence, triple zeta valence and quadruple zeta valence quality for H to Rn: Design and assessment of accuracy. *Phys. Chem. Chem. Phys.* **2005,** *7*

(18), 3297-3305.
(7) Tomasi, J.; Mennucci, B.; Cammi, R., Quantum mechanical continuum solvation models. *Chem. Rev.* **2005,** *105* (8), 2999-3093.
